# Health factors that influence sustainable behaviour in a single-player resource management game

**DOI:** 10.3758/s13423-023-02341-y

**Published:** 2023-09-15

**Authors:** Paul Rauwolf, Arlen McKinnon, Amy C. Bilderbeck, Robert D. Rogers

**Affiliations:** 1 School of Psychology and Sport Science, Bangor University, Brigantia Building, Bangor, Gwynedd UK LL57 2AS; 2 P1vital Products Ltd, Manor House, Howbery Park, Wallingford, Oxfordshire UK OX10 8BA

**Keywords:** Resource management, Delay discounting, Alcohol use, Psychological distress

## Abstract

Encouraging sustainable use of limited natural, social, and economic resources requires understanding the variety of ways in which people think about how resources work and how they adjust their behaviour (or not) as available resources fluctuate. Previous investigations which have focused on understanding how individuals navigate erodible resources, have tended to use group-based, common pool games. However, such social games make it difficult to disentangle whether resource erosion is linked to difficulty navigating the dynamics of the resource or caused by social factors. Here, in two experiments, we recruited 781 participants to play a single-player resource management game in which individuals were invited to harvest monetary rewards from a fully depletable but stochastically replenishing resource over time. We find that the ability to sustain a resource over successive harvesting opportunities (in order to maximize the total harvested rewards) is reliably worse in individuals reporting elevated psychological distress, the often cooccurring hazardous alcohol use, and elevated rates of delay discounting. The associations between resource outcomes, harmful alcohol use, and psychological distress remained substantial even once we had controlled for elevated discounting rates (as a form of impulsivity and a strong risk factor for these health challenges). By contrast, individuals who reported higher levels of financial literacy and general well-being achieved better resource outcomes. Our observations demonstrate that the capacity to respond effectively to the dynamics of a resource are compromised in individuals at risk of psychological and alcohol-related disorders.

**Supplementary Information:**

The online version contains supplementary material available at 10.3758/s13423-023-02341-y.

## Introduction

Identifying sustainable ways to use valuable but erodible resources is now an economic, environmental and public health imperative for individuals, communities, and governments (Armstrong & Kamieniecki, 2019; Kanger et al., 2020; Macke & Genari, 2019). Characterizing the variation in behaviour and outcomes of behavioural economic ‘take-some’ common-pool games (Balliet et al., 2009; Brewer & Kramer, 1986) can help us to understand the different ways in which individuals engage with dynamic but depletable resources and contribute to the development of effective interventions and policy across communities.

Surveying the literature, an extensive evidence-base indicates that behaviour and resource failures in common-pool games can involve the cumulative impacts of individuals’ attitudes, cognitions, affect, and behavioural biases (Van Lange et al., 2013). Careful harvesting behaviours can reflect individuals’ prosocial attitudes (Balliet et al., 2009; Bogaert et al., 2008; Kramer et al., 1986), environmental attitudes (Sussman et al., 2016), emotional reactivity to environmental concerns (Tarditi et al., 2018), intrinsic values and motivations (Han et al., 2018), and risk-aversion (Chermak & Krause, 2002). More broadly, there are mixed findings of differing harvesting behaviours in males compared with females, but more consistent evidence of smaller harvests (in order to sustain resources) in older compared with younger individuals (Chermak & Krause, 2002; Tisserand et al., 2022). Lastly, groups consisting of higher average cognitive ability (as ‘Spearman’s g’) and stronger appreciation of other peoples’ mental states (as ‘Theory of Mind’) tend to sustain resources for longer and (in the former case) maximize totals harvested (Freeman et al., 2020).

While these experiments suggest that sustainable behaviour and outcomes of common-pool games are moderated by individual characteristics, interpreting their findings is challenging. Behaviour in common-pool games reflects the integration of at least two sets of socio-cognitive processes: (i) individuals’ understanding and responses to the social aspects of managing a resource in a group (e.g. attributions of partners’ motivations, affective reactions to partner behaviours, and predicted harvests) and (ii) learning about the dynamics of a resource itself and the other externalities that contribute to its fluctuating value against harvesting demands. Of course, these processes are not independent but interrelate in complex ways; for example, subtle and diffuse social framing effects operate across single-player games and both formal experimental and commercial game settings (Bernold et al., 2023; Ellingsen et al., 2012; Stenros et al., 2009).

Nonetheless, navigating the dynamics of the resource is also nontrivial. Even in common-pool games, individuals often need to diagnose the properties of the resource over time (e.g. they are often not given the replenishment rate of the resource and must estimate this through trial and error). So, behaviour may be conditioned by an individual’s understanding of the resource, their estimate of the likely resource replenishment rate, or how steeply they discount the value of future benefits afforded by a still-viable resource (Hendrickx et al., 2001). Thus, investigations with single-player games offer opportunities to observe how individual characteristics moderate responses to resource dynamics, while removing the most overt social challenges inherent in multiplayer games.

A relatively small number of studies have removed the social aspects of common pool games and tested whether individuals struggle to navigate resource dynamics in isolation. The outcomes in single-player resource games, like those of common-pool games, tend to be heterogenous (Hey et al., 2009; Messick & McClelland, 1983; Schnier & Anderson, 2006). Operating by themselves, some struggle to sustain resources, eroding or exhausting them prematurely. This is true even for individuals with expertise in resource dynamics, such as experienced fishers and policy makers (Moxnes, 1998, 2000). It is also the case irrespective of whether resources are framed as natural in kind, such as fish stocks (Brechner, 1977; Knapp & Clark, 1991; Moxnes, 1998; Tice et al., 2001) and grazing land (Moxnes, 2000, 2004) or as abstract pools of nominal rewards (Messick & McClelland, 1983). Individuals can also struggle to sustain resources when replenishment rates reflect biologically informed contingencies (Moxnes, 1998, 2000) or simpler stochastic percentages (Knapp & Clark, 1991; Messick & McClelland, 1983; Moxnes, 2004). Further, single-player resource management outcomes are varied in both children (Koomen & Herrmann, 2018a) and chimpanzees (Koomen & Herrmann, 2018b)—although children and chimpanzees perform better in isolation compared with group-based settings.

Despite the fact that some individuals struggle to navigate resource dynamics in a single-player game, there has been little work which has tried to understand the individual characteristics associated with (un)sustainable behaviour in a single-player setting. However, we believe that improving our understanding of the individual characteristics associated with outcomes in single-player resource games is an important precursor to understanding the difficulties faced when individuals are placed in more complex, social resource management contexts, such as common pool games. As noted above, since common pool settings have interrelated underlying socio-cognitive processes, using single-player games can help us better isolate and investigate how individuals navigate resource dynamics. Future work on group-based resource maintenance can then amalgamate the causes underlying variation in individual resource management behaviours with the social dynamics of multipartner interactions.

In these experiments, we begin the process of investigating how individual characteristics relate to resource outcomes in a single-player game. Specifically, we evaluate whether adverse health experiences and their risk factors are associated with resource outcomes. We test whether resource outcomes vary by validated measures of psychological distress (as depressive and somatic symptoms; Goldberg et al., 1997), hazardous alcohol use (Reinert & Allen, 2002; Saunders, Aasland, Amundsen et al., 1993), and delay discounting (Koffarnus & Bickel, 2014).

There are three reasons why we expect poor sustainable behaviour to be linked to psychological distress, harmful alcohol use, and delay discounting rates. First, at an observational level, social and economic hardships are associated with high rates of depression, anxiety, and alcohol misuse (Adda et al., 2009; Bellis et al., 2016; Marmot, 1999) as well as other risky, unhealthy behaviours (Haushofer & Fehr, 2014; Huckle et al., 2010; Makela, 1999; Sze et al., 2017). In turn, the impacts of these experiences (e.g. prolonged stress) can undermine the effective use of financial, social, and clinical resources necessary for the development of resilience and/or protection against relapse (Adinoff et al., 2016; Haushofer & Fehr, 2014; Richardson et al., 2017). Thus, we hypothesize that the resource outcomes of affected individuals playing a single-player game will mirror their lived experiences of resource management and be demonstrably poorer as a function of self-reported depressive symptoms and hazardous alcohol use.

Alongside this, a large evidence-base indicates that depression, anxiety, and alcohol misuse are associated with difficulties in reward-based (‘reinforcement’) learning and decision-making under conditions of uncertainty (Bishop & Gagne, 2018; Gray & MacKillop, 2015; Hagiwara et al., 2022). This suggests that resource management in single-player games, mediated by these same cognitive functions, will be altered in individuals reporting these heath challenges. So far as we are aware, however, the only relevant evidence in the context of single-player games is two demonstrations of poor resource outcomes following transient reductions in mood in samples of broadly healthy adults (Knapp & Clark, 1991; Tice et al., 2001). At the current time, there are no published tests of how the resource outcomes in single-player games vary by validated measures of self-reported psychological distress, alcohol use or well-being. Here, we test these associations directly.

Finally, at a theoretical level, other data demonstrate that behavioural responses to social dilemmas (e.g. in common-pool games) can reflect ‘temporal traps’ where individuals may struggle to consider the importance of preserving resources for the longer term (Mannix, 1991). More broadly, however, steeper delay discounting rates (as individuals’ tendency to devalue rewards over increasing time intervals to their delivery; Ainslie, 1975; Odum, 2011; Petry, 2001; Story et al., 2014) can be associated with diminished appraisals of the seriousness of human-instigated environmental change (Farias et al., 2021) and fewer self-reported pro-environmental behaviours (Sahraeian et al., 2021). As such, variability of delay discounting is a central aspect of how we measure and understand poor resource management behaviour (Hirsh et al., 2015).

Convergently, steeper discounting rates are also a well-attested risk factor for a number of psychological disorders linked to problematic patterns of consumption, including alcohol use disorders (Case et al., 2019) and co-occurring: depression, somatic symptoms, and social withdrawal (Åhlin et al., 2015; Fields et al., 2014; Levitt et al., 2023). So, good resource outcomes in single-player games are likely to reflect individuals’ tolerance of delays to the larger rewards (offered by sustained resources) while poor outcomes are likely to reflect, at least sometimes, early aggressive harvesting mediated by higher rather than lower delay discounting rates (Odum, 2011; Petry, 2001; Story et al., 2014). Thus, examining the variability in the resource outcomes of individuals reporting harmful alcohol use, stress, and depression may offer a fresh perspective on how elevated delay discounting (which is associated with changes in other cognitive and affective processes) operates to promote maladaptive resource management behaviour in vulnerable populations. Our experiments offer the first test of these possibilities.

Here, in two separate experiments, we sought to characterize the varying behaviours and outcomes of a single-player resource management game in which individuals were invited to harvest monetary rewards from a fully depletable but replenishing resource over time. Overharvesting behaviours tended to diminish the resource, limiting future opportunities to gather rewards; while more moderate harvesting sustained the resource, preserving opportunities to gather more rewards for longer (Messick & McClelland, 1983). In Experiment 1 (*N* = 400), we tested the hypothesis that resource outcomes are poorer for individuals reporting higher levels of psychological distress (and accompanying somatic symptoms), higher delay discounting, lower well-being, and stronger patterns of hazardous drinking. We also sought to identify other factors that might *improve* the management of resources and so we tested whether resource outcomes are positively associated with the life-skill of financial literacy. In Experiment 2 (*N* = 381), we sought to replicate part of Experiment 1, and tested whether high delay discounting and strong patterns of alcohol use were associated with poorer resource outcomes.

## Experiment 1

We sought to better understand the relationship between the resource outcomes in a single-player resource management game and (i) psychological distress—using the 12-item General Health Questionnaire (GHQ-12; Goldberg et al., 1997; Hankins, 2008); (ii) hazardous alcohol use—measured by the 10-item Alcohol Use Disorders Identification Test (AUDIT; Saunders, Aasland, Amundsen et al., 1993); (iii) delay discounting rates—using the 5-item ED_50_ elicitation (Koffarnus & Bickel, 2014); (iv) financial literacy—using 9 Organisation for Economic Co-operation and Development (OECD) survey items (Čonková, 2014) and; (v) general well-being—measured by the 5-item World Health Organisation Well-Being Index (WHO-5; Topp et al., 2015).

## Method

### Participants

Ethical approval for the protocol was given by the School of Psychology Research Ethics Committee at Bangor University. Four-hundred participants took part, recruited from the online participant pool, Amazon Mechanical Turk (MTurk; http://mturk.com). The sample consisted of 178 females, 221 males, and one nonbinary participant, with a mean age of 34.4±10.2 (*SD*). The survey questions were hosted on the Qualtrics platform, and participants completed the survey through their web browser. The experiment was only made available to MTurk workers based in the United States of America with at least a 95% approval rating from previous studies. No participant was excluded.

Participants were told they would earn a minimum base pay of $1.50 for completing a 15–20 minute online survey, with a chance to earn a bonus payment of up to a value of $3, based on how well they performed in a resource game. Potential payments of $13/hour is generous compared with the average MTurk compensation (Hara et al., 2018), so we believed this would provide sufficient motivation. Of note, after the experiment, we calculated that participants earned an average bonus of $0.87±$.61 (*SD*), on top of their base pay (bonus payments ranged from $0.30 to $2.47). Using the time it took for each participant to complete the protocol, the average rate of pay was $8.00±$3.42 per hour.

### Materials

#### Resource management game

**Fig. 1:**
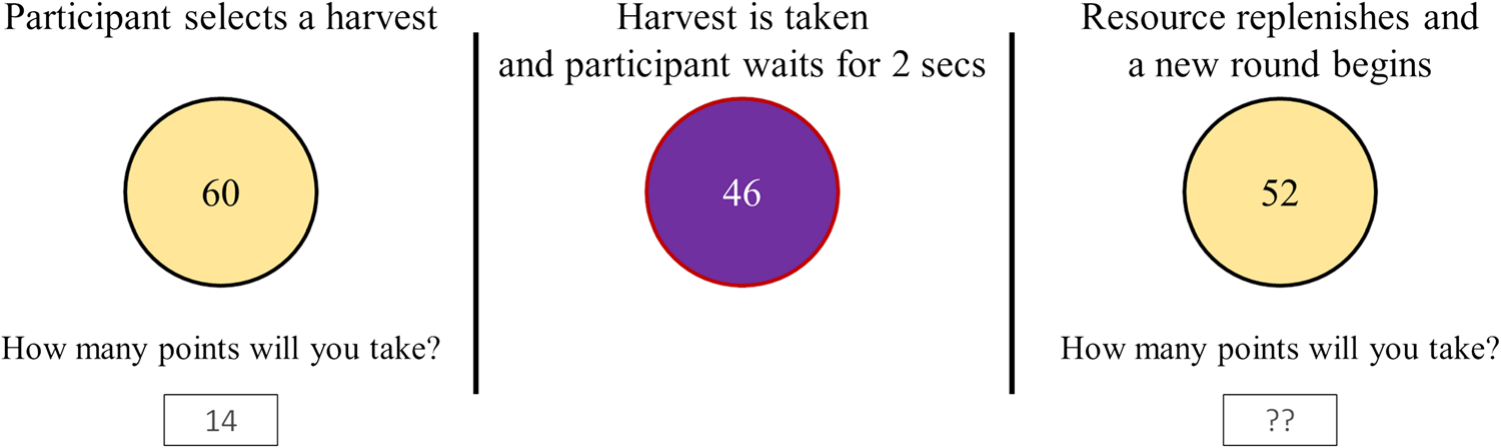
Flow chart of a round of play during the single-player resource management game. Left: First, participants see a resource with 60 points and may take any number of points. Middle: the points are then removed, and the participant waits for 2 seconds. Right: Finally, the resource replenishes, and the next harvesting phase begins

At the beginning of the protocol, participants played the resource management game. When the game began, participants saw a pool or ‘resource’ of 60 rewards—its maximum value—and were told they could earn bonus money based on the number of rewards they harvested. This bonus was capped at $3, and participants were not told the value of each harvested reward ($0.005) until after the game finished. Each harvesting opportunity, or ‘round’, was broken into three phases (see Fig. 1). First, participants selected how many rewards they wished to harvest from the resource. They could harvest any amount. Second, the value of the harvest was subtracted from the resource and added to the participant’s (unseen) total accumulated rewards. Finally, the remaining pool was replenished by a hidden replenishment function.

Behavioural models of resource management have tended to use one of two replenishment functions: (i) Gaussian (bell-shaped) functions where the optimal behaviour is to harvest half the resource (Knapp & Clark, 1991; Moxnes, 2000) or (ii) stochastic percentages of the remaining resource (Brechner, 1977; Messick & McClelland, 1983), truncating the replenished value if it grows the resource beyond a maximum bound. Here, we reasoned that a simple bell-curve replenishment function (that offers a 100% replenishment rate for harvests of half the resource) might not be sufficiently challenging to differentiate participants’ performance in our game. We wanted to evaluate behaviour on a resource that was sufficiently vulnerable to overharvesting. Therefore, we opted for a stochastic mechanism that, following each harvest, drew replenishment values from a Gaussian distribution (μ = 15%; σ = 3%). Since these replenishments were based upon the value of the remaining resource, participants could maximize their total rewards gathered by making modest (rather than aggressive) harvests (please see below for a more detailed discussion of optimal harvesting behaviour).

The game ended when participants exhausted the resource completely or after 70 rounds. Participants were not told the replenishment rate or the maximum number of rounds. To succeed, participants had to try to estimate the replenishment rate on the basis of exploring the impacts of harvesting behaviours on the available resource, all the while paying long-term costs if they harvested too much (e.g. it might take several rounds of not harvesting or minimal harvesting to restore the resource once it is severely depleted).

Before starting the game, participants were told that if they depleted the resource quickly, they would have to wait up to 5 minutes before proceeding with the rest of the protocol. Unbeknownst to participants, if they exhausted the resource completely before round 50, they had to wait 6 seconds for every round remaining between the round the resource depleted to zero and round 50 (e.g. if they depleted the resource at round 10, they would have to wait 4 minutes before proceeding; 40 × 6 = 240 seconds). In such an instance, the participant was shown a timer which counted down to zero, and the ‘Next’ button was disabled until the counter reached zero (please see Supplemental Material A for more details). This arrangement was adapted from previous experiments with delay discounting elicitations (Sonuga-Barke et al., 1992) to mitigate the impacts of participants’ general delay aversion and moderate the relative value of ‘opportunity costs’ to do other things once participants had completed the game. In other work, we have demonstrated that this incentivizing mechanism increases engagement when playing the resource management game, since it was in the participants’ best interest to learn to maintain the resource (Rauwolf & Rogers, 2023).

##### Optimal harvesting behaviour

Importantly, the game was created such that optimal play involves sustaining the resource at a high level. The optimal policy was calculated by framing the resource management game as a Markov decision process (Bellman, 1957; Sutton & Barto, 2018). Dynamic programming was then used to evaluate the best harvesting action to take at each possible value of the resource (please see Supplemental Material B for an in-depth discussion of the process; Bellman, 2010; Puterman, 1994). In this case, the optimal strategy is to harvest zero if the resource has a value of 51 or less. If the resource has a value above 51, the optimal harvesting behaviour is to harvest the resource down to a value of 51. Given that the resource replenishes more points when the resource has a larger value, but also cannot grow above 60, aiming for a resource value of 51 strikes the perfect balance between harvesting as many points as possible while still allowing the resource to replenish close to its maximum level each round. Such a sustainable strategy maximizes the long-term earnings of a player.

#### Psychometric measures

Following the game, participants completed a subset of the following self-assessments: (i) GHQ-12 (Goldberg et al., 1997), (ii) AUDIT (Reinert & Allen, 2002), (iii) ED_50_ (Koffarnus & Bickel, 2014), (iv) OECD financial literacy questionnaire (Čonková, 2014), and (v) WHO-5 (Hall et al., 2011; Topp et al., 2015).

##### Psychological distress

The General Health Questionnaire (GHQ) was originally developed to help identify psychological distress (Goldberg & Blackwell, 1970). We used the short-form, 12-item unidimensional version that captures psychiatric dysfunction in three domains: social dysfunction, anxiety, and loss of confidence (Graetz, 1991; Hankins, 2008). The measure is well-validated against longer versions of the GHQ (Goldberg et al., 1997).

##### Hazardous alcohol usage

The Alcohol Use Disorders Identification Test (AUDIT) was developed as a 10-item screening questionnaire to assess harmful alcohol usage (Saunders, Aasland, Babor et al., 1993). It has been widely used in community and clinical samples and shows good construct and criterion validity (Reinert & Allen, 2002, 2007).

##### Delay discounting

Delay discounting is the tendency to devalue rewards (i.e. discount them) the longer one has to wait for the delivery. High delay discounting (i.e. the tendency to devalue rewards steeply as a function of time) is associated with several health risk-factors and adverse health experiences (Story et al., 2014). These include hazardous patterns of alcohol use (Petry, 2001), substance misuse (Kirby et al., 1999), obesity and associated metabolic disorders (Barlow et al., 2016), and gambling (Madden et al., 2011). The ED_50_ elicitation consists of five forced-choice items asking participants whether they prefer $500 now or $1,000 at some later delay. The ED_50_ finds the delay (between 1 hour and 25 years) that devalues a reward by 50% (Koffarnus & Bickel, 2014).

##### Financial literacy

Financial literacy is identified by the International Network on Financial Education (INFE) as ‘a combination of awareness, knowledge, skills, attitude and behaviour necessary to make sound financial decisions and ultimately achieve individual financial well-being’ (OECD, 2018, p. 4). Financial literacy is known to correlate with financial well-being (Taft et al., 2013). We used a short form of the OECD financial literacy survey (Čonková, 2014).

##### Well-being

The World Health Organization Five Well-Being Index (WHO-5) is a short-report measure of subjective well-being. It has been shown to be a good screening tool for depression (Topp et al., 2015) and correlate of subjective quality of life (Hall et al., 2011).

### Procedure

Participants read a brief information page and provided informed consent (see Supplemental Material A for full details and screen displays of the protocol). Next, participants completed a couple of demographic questions (i.e. gender and age). Then, they completed a short informational session on the resource management game. Following this, participants were asked three questions to demonstrate their understanding of the game. They were not able to continue until they had answered these questions correctly, repeating the information session until they demonstrated their understanding of how to play the game (see Supplemental Material A). Participants then played the resource management game. Upon completion, participants were asked a few questions about their strategy and how they felt after playing the game (these questions were exploratory and not considered in the analysis—please see Supplemental Material A for more details).

Next, participants completed various psychometric measures. Data collection was completed in two waves. In the first wave, 200 participants completed the (i) ED_50_, (ii) GHQ-12, (iii) AUDIT, and (iv) WHO-5. In the second wave, 200 participants completed the elicitations for (i) ED_50_ and (ii) financial literacy. Finally, participants were informed of the bonus money they had earned, debriefed, and told they would be paid shortly. All data can be found online (https://osf.io/8b7av/.).

## Results

**Fig. 2:**
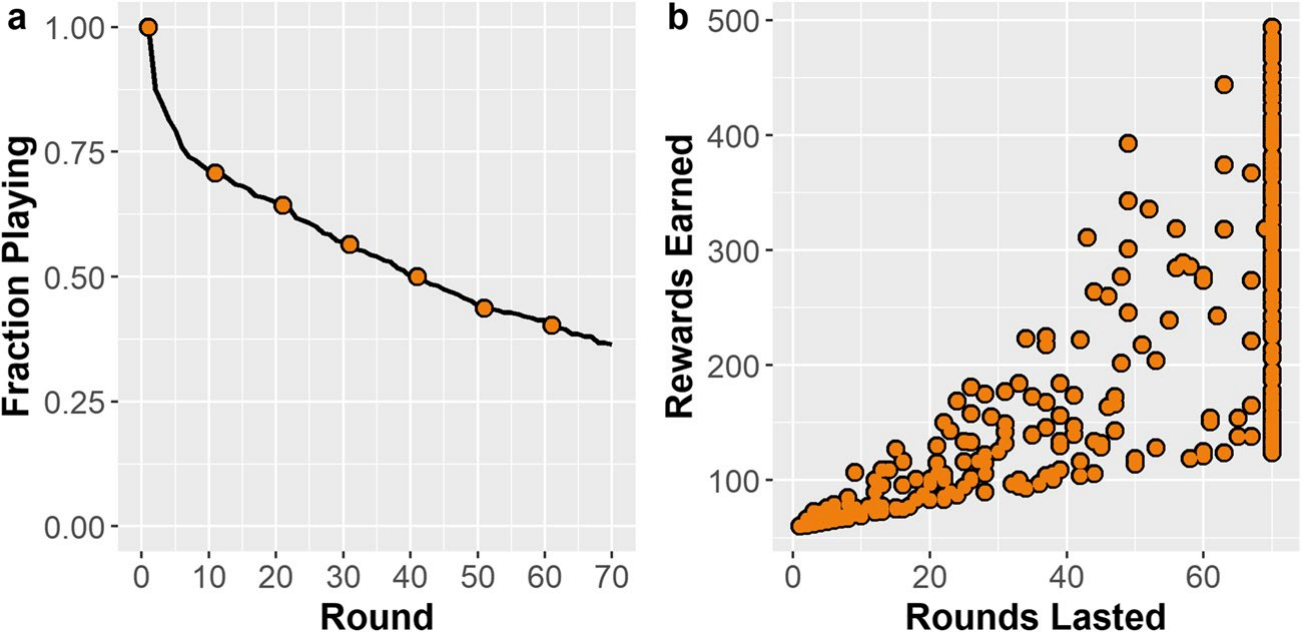
Left: Proportion of participants still playing after a given round. Right: Relationship between rounds lasted and rewards earned for each participant

Internal consistency of the questionnaire scores was generally excellent, with Cronbach’s α’s of: 0.87 (GHQ-12), 0.95 (AUDIT), and 0.92 (WHO-5). The one exception was the measure of financial literacy, but this remained within acceptable ranges: α = 0.76. We considered two outcome measures: (i) the number of rounds sustained with a positive resource value and (ii) the total rewards harvested. Figure 2 (left) shows the proportion of participants still sustaining the resource over 70 rounds of the single-player game. As expected, there was large variation in outcomes across the sample. Whilst over 25% of participants completely depleted the resource by round 10, 36% maintained the resource over all 70 rounds.

Figure 2 (right) illustrates the variation and heteroskedasticity in the total rewards collected relative to the number of rounds lasted. While total rewards were strongly associated with the number of rounds lasted (Spearman’s rank order: *r*_*s*_(398) = 0.88, *p* = 2.2×10^-16^), the Breusch–Pagan test for heteroskedasticity showed that this association weakened among individuals who sustained the resource for longer (Breusch–Pagan = 153.76*, p* = 2.2×10^-16^). So, among participants who sustained the resource for the whole game, some collected almost 500 rewards, while others harvested as few as 125 rewards (which was fewer than some participants who only lasted 15 rounds). This demonstrates that the total rewards gathered over the course of the game can dissociate from the number of rounds sustained, and that there was large variation in outcomes even amongst those who sustained the resource across all 70 rounds of the game. Thus, while many participants were able to last the maximum number of rounds, very few were able to approach the maximum number of rewards earned.

Next, we tested whether this variation in resource outcomes could be explained by health experiences. AUDIT scores indicated highly variable but overall borderline harmful alcohol use (Mean: 9.8±10.1). Similarly, GHQ-12 scores and WHO-5 scores indicated threshold psychological distress and moderate well-being (14.7±5.1 and 12.2±6.5, respectively). As expected, delay discounting rates (calculated as log of k) showed significant variability (−4.4±2.8), but financial literacy less so (34.3±5.8). The dataset did not meet several of the assumptions for parametric analysis techniques (see Supplemental Material C for an in-depth analysis and discussion). As such, we used two non-parametric techniques to evaluate whether resource outcomes from the single-player game showed significant associations with health experiences.

First, we evaluated the associations between each resource outcome and each psychometric variable using Spearman’s rank correlation. Participants who reported more hazardous alcohol use sustained the resource for fewer rounds, *r*_*s*_(198) = −0.59, *p* = 2.2×10^-16^, and harvested fewer rewards, *r*_*s*_(198) = −0.56, *p* = 2.2×10^-16^. Gignac and Szodorai (2016) recommend that individual difference researchers refer to correlations of 0.1, 0.2, and 0.3 as small, medium, and large, respectively. Therefore, since Spearman’s rank correlation (*r*_*s*_) was greater than .3, there was a large effect between hazardous alcohol use and the two resource outcomes. For brevity, scatterplots of the data can be found in Supplemental Material D.

Participants who reported recent psychological distress (as GHQ-12 scores) also sustained the resource for fewer rounds and accumulated fewer rewards over the course of the game, *r*_*s*_(198) = −0.19, *p* = .007, and *r*_*s*_(198) = −0.18, *p* = .014, respectively, demonstrating a medium-small effect. By contrast, better financial literacy was associated with sustaining resources for longer, *r*_*s*_(198) = 0.35, *p* = 3.81×10^-7^, and gathering more rewards, *r*_*s*_(198) = 0.28, *p* = 4.53×10^-5^—a large-medium effect-size. Similarly, better well-being (as WHO-5 scores) was associated with sustaining the resource longer, *r*_*s*_(198) = 0.23, *p* = .001, and gathering more rewards, *r*_*s*_(198) = 0.18, *p* = .009—a medium-small effect. Please see Supplemental Material D for visuals.

As expected, steeper discounting rates were linked to early depletion of resources, *r*_*s*_(398) = −0.31, *p* = 1.39×10^-10^, and fewer rewards gathered, *r*_*s*_(398) = −0.27, *p* = 4.07×10^-8^—a large-medium effect. Consistent with other reports (Sze et al., 2017), steeper delay discounting rates were correlated with hazardous alcohol use, *r*_*s*_(198) = 0.41, *p* = 1.48×10^-9^. However, partial Spearman correlations showed that early exhaustion of the resource (to zero) and fewer rewards gathered over the course of the game remained linked to both hazardous alcohol use, *r*_*s*_(197) = −0.38, *p* = 1.25×10^-7^; *r*_*s*_(197) = −0.41, *p* = 1.39×10^-8^ and psychological distress, *r*_*s*_(197) = −0.18, *p* = .019; *r*_*s*_(197) = −0.17, *p* = .023, even when delay discounting rates were controlled for statistically by adding it as a covariate (Liu et al., 2018). Similarly, both resource outcomes remained linked to better financial literacy, *r*_*s*_(197) = 0.26, *p* = .0006; and *r*_*s*_(197) = 0.21, *p* = .0039, when controlling for delay discounting. These observations show that variable resource outcomes amongst those vulnerable to adverse health experiences are not simply a matter of elevated impulsivity in the form of steeper delay discounting rates.

While the Spearman’s correlations indicate general associations, the relationships between resource outcomes and characteristics were nonuniform across performance. To analyze the relationships at various levels of performance, we employed quantile regression (Koenker & Bassett, 1978). Like ordinary least squares (OLS) regression, quantile regression fits a line through data points to model the relationship between independent variables and a dependent variable. However, the quantile regression line is fit differently compared with OLS regression. In quantile regression, the line minimizes the weighted distances between observed values and the values predicted by the model such that a certain proportion of the data points fall above and below the regression line. For example, a quantile regression line fit for the 0.2 quantile is fit such that 20% of the data points lie below the line, while a quantile regression line fit for the median (the 0.5 quantile) is fit such that 50% of the data lie below the regression line (Cook & Manning, 2013; Koenker & Hallock, 2001). Similar to OLS regression, equations can then be constructed for these lines, where the regression coefficient represents the predicted rate of change in the dependent variable per unit change in an independent variable for that specific quantile (Cade & Noon, 2003).

Here, for each questionnaire measure and dependent measure pair, we computed quantile regression models for the 0.05 to 0.95 quantiles, in increments of 0.05. For these models, we normalized the questionnaire scores to a mean of 0 and a standard deviation (*SD*) of 1. This allowed comparison across models involving questionnaires with different maximum scores. In the resulting models, the regression coefficients at each quantile represent the estimated change in the dependent variable (rounds lasted or total rewards collected) for an increase of 1 standard deviation in an individual characteristic.

**Fig. 3:**
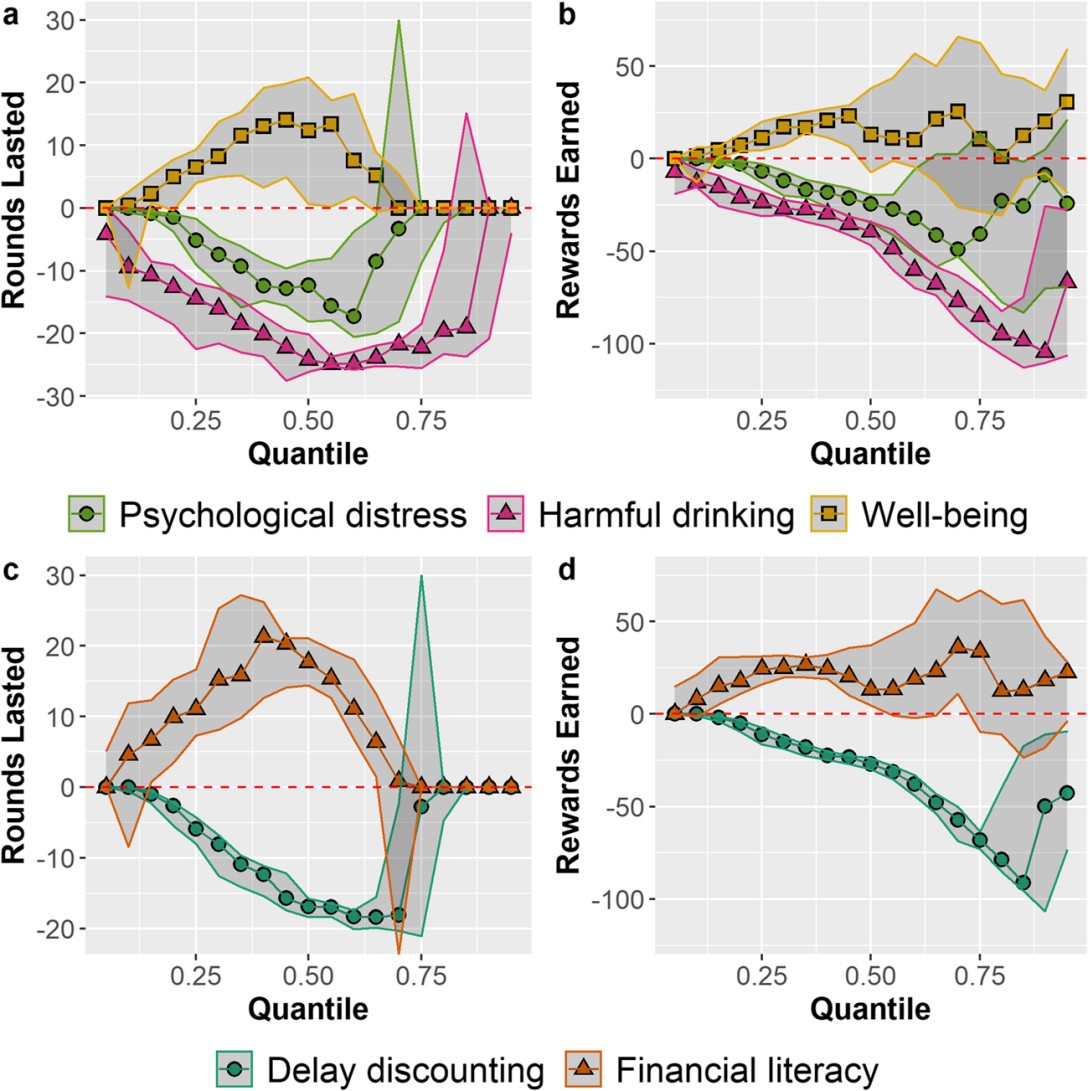
Quantile regression coefficients for normalized (mean = 0; SD = 1) harmful alcohol usage (AUDIT), psychological distress (GHQ-12), well-being (WHO-5), delay discounting (ED_50_), and OECD financial literacy scores when predicting rounds lasted **(a & c)** and rewards gathered **(b & d)** from quantiles 0.05–0.95 in increments of 0.05. Shaded areas represent 95% CIs

Figure 3 shows the associations between the individual characteristics and both rounds lasted (a and c) and total rewards collected (b and d), at different levels of performance. Each dot represents the regression coefficient of the change in rounds lasted or rewards gathered for an individual characteristic at that quantile. The translucent bands represent 95% confidence intervals for these regression coefficients at each quantile (Koenker, 1994). For example, looking at Fig. 3c, the regression coefficient for financial literacy at the 0.25 quantile was 11 rounds lasted, with a 95% CI [7, 17]. This is equivalent to saying that, at the 0.25 quantile, a participant with a financial literacy score 1 standard deviation higher than another participant would be expected to last 11 more rounds in the game. Where the interval does not cross 0 (i.e. dotted red line) in these plots, we can be 95% confident that the resource outcome at that quantile can be predicted on the basis of that characteristic.

Figure 3a and Fig. 3c show that rounds lasted was significantly associated with all the characteristics around the median; that is, between the 0.20 and 0.70 quantiles. Specifically, at the median, the number of rounds over which individuals sustained the resource was predicted to decrease by 13 rounds for every increase of 1 standard deviation of psychological distress (Fig. 3a), by 25 rounds for every increase of 1 standard deviation of harmful alcohol usage (Fig. 3a) and by 17 rounds for every increase of one standard deviation of delay discounting (Fig. 3c). By contrast, the number of rounds survived was predicted to increase by 18 rounds for those who were more financially literate (Fig. 3c) and by 12 rounds for every increase of 1 standard deviation in general well-being (Fig 3a). Most of these associations diminished amongst those who sustained the resource for the longest (>0.75 quantile) since the best performers survived all 70 rounds, regardless of their characteristics. Please see Supplemental Material D for visualizations of the best fit quantile lines in relation to the raw data.

Figure 3b and d show the associations between the total rewards collected and characteristics as a function of performance. Negative associations with psychological distress (GHQ-12) are evident among the participants between the 0.25 and 0.60 quantiles. By contrast, we found increasingly negative associations between the total rewards gathered and both increasing hazardous alcohol use and increasing delay discounting across almost all quantiles. The effect was particularly stark among the very best performers. At the 0.85 quantile, 98 rewards might be lost for every increase of one standard deviation in hazardous alcohol usage (see Fig 3b); similarly, 91 rewards might be lost for 1 standard deviation in delay discounting rate (Fig 3d). Finally, among the performers within the 0.10–0.50 quantiles, more rewards were gathered with improved well-being and financial literacy (see Supplemental Material D for more visuals).

Experiment 1 demonstrates that individuals’ outcomes (in terms of sustaining a resource to facilitate the harvesting of small monetary rewards) in a single-player resource management game vary markedly, and are worse with self-reported recent psychological distress, hazardous alcohol use, and rapid delay discounting. By contrast, the same outcomes appear to be improved among individuals with better self-reported well-being and financial literacy. Overall, the strongest relationships involved hazardous alcohol use (as measured with the AUDIT) and delay discounting (as the rates elicited by the ED_50_). Importantly, the links between resource outcomes and hazardous drinking (and indeed psychological distress) do not appear to be artefacts of covarying rapid delay discounting. This is because the former associations survived correction for participants’ discounting rates. In Experiment 2, we consolidate these findings with a direct replication.

## Experiment 2

In Experiment 2, we sought to replicate the effects of delay discounting and harmful alcohol usage found in Experiment 1. Participants played the same single-player resource management game as above. They then took two psychometric questionnaires: the 10-item Alcohol Use Disorders Identification Test (AUDIT; Saunders, Aasland, Babor, et al., 1993) and the 5-item ED_50_, delay discounting elicitation (Koffarnus & Bickel, 2014). Experiment 2 was part of a larger set of experiments, reported elsewhere, that addressed a distinct research question (McKinnon et al., 2023). However, this is the first time this dataset has been used to consider the effect of individual characteristics on resource outcomes.

## Method

### Participants

Ethical approval for this experiment was granted by the Psychology Research Ethics Committee at Bangor University. Three hundred and eighty-one participants took part, recruited from Amazon Mechanical Turk. As in Experiment 1, participants completed an online protocol hosted on Qualtrics. The sample consisted of 158 females, 222 males, and one participant who preferred not to identify their gender. Participants had a mean age of 35.3±10.7 years. No participant was excluded.

The experiment was only made available to MTurk workers based in the United States of America with at least a 95% approval rating from previous tasks. Participants were paid a base fee of $0.75 for participating and told they could earn a bonus payment up to value of $6.00. They could earn up to $3.00 depending on their performance in the single-player game, and another $3.00 for a different game, which is not described here. As in Experiment 1, participants were not told the value of each reward harvested in the game ($0.005) until they had completed the entire experiment. Of note, after the experiment, we calculated that participants earned an average bonus of $0.76 ± $.59 (*SD*) for the single-player game (bonus payments ranged from $0.30 to $2.53). Using the time it took for each participant to complete the protocol, along with all bonus payments, the average rate of pay was $7.74± $4.05 per hour.

### Materials

The materials were similar to those used in Experiment 1. Participants played a resource management game (see Experiment 1 and Fig. 1). Then, participants completed measures of harmful alcohol use (AUDIT) and delay discounting (ED_50_)—please see Experiment 1 for more details on these measures.

### Procedure

First, participants were presented with an information sheet and completed a consent form (please see Supplemental Material A for full details of the experimental procedure). Next, participants answered a couple of demographic questions (age and gender). As in Experiment 1, participants were given instructions on how to play the resource management game. Prior to playing the game, participants had to correctly answer four questions, demonstrating their understanding of the game. Participants then played the resource management game. Upon completion, if they accrued a time-penalty for depleting the resource early, they had to wait before proceeding. Then, participants were informed of how much bonus money they had earned. As this experiment was part of a multifaceted procedure, the participants then played a different game, which is not considered in this work. Afterwards, participants completed the ED_50_ followed by the AUDIT. Finally, participants were debriefed, reminded of how much bonus money they had earned, and told they would receive payment within 72 hours. All data can be found online (https://osf.io/8b7av/).

## Results

**Fig. 4:**
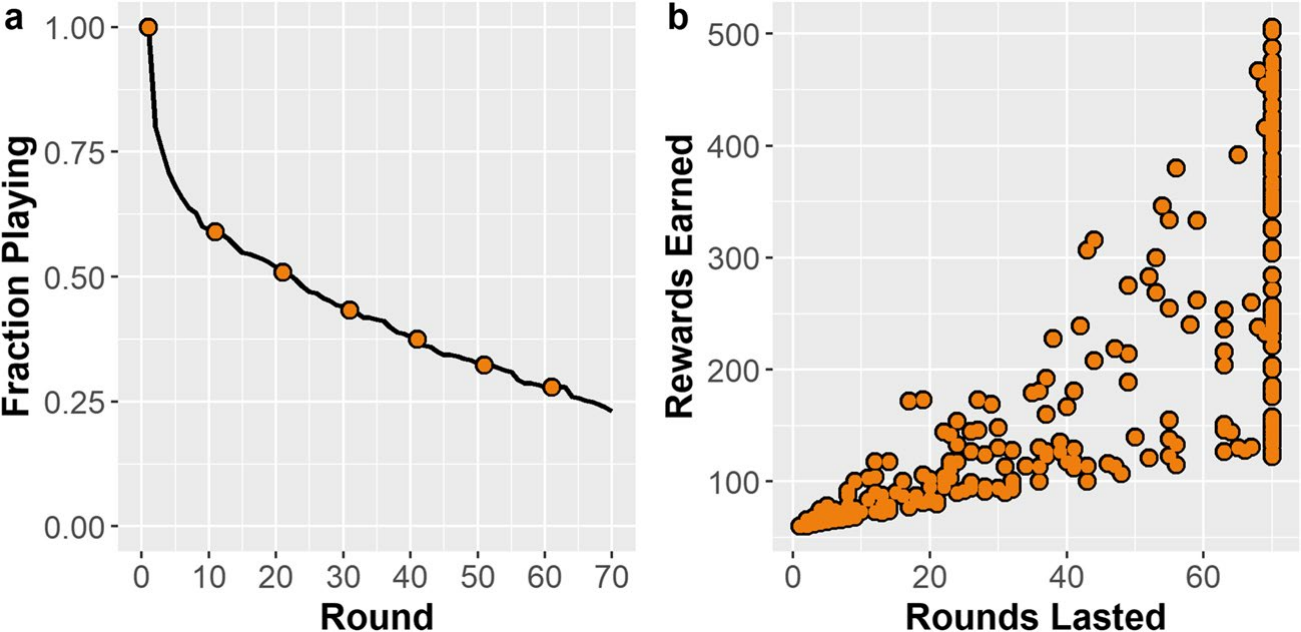
Left: Proportion of participants still playing after a given round. Right: Relationship between rounds lasted and rewards earned for each participant

The data showed significant heteroskedasticity (see Supplemental Material C), so data analysis was completed with the same statistical tests as Experiment 1. Figure 4 (left) shows the proportion of participants still sustaining the resource at each of the 70 rounds of the resource management game. Forty-one percent of participants fully exhausted the resource by round 10, while 23% maintained the resource across the maximum 70 rounds. Figure 4 (right) shows how, as in Experiment 1, the variation in rewards gathered increased with the number of rounds lasted. While many participants were able to last the maximum 70 rounds, few were able to harvest a large number of rewards.

Next, we explored the relationships between resource outcomes and (i) harmful alcohol use (*M* = 12.03, *SD* = 11.05), and (ii) delay discounting (calculated as log of k; *M* = −3.34, *SD* = 3.27). The responses to the AUDIT questionnaire showed excellent internal consistency, with a Cronbach’s α of 0.95.

Nonparametric Spearman correlations showed that participants reporting hazardous alcohol use tended to sustain the resource for fewer rounds, *r*_*s*_(379) = −0.54, *p* = 2.2×10^-16^, and gather fewer rewards, *r*_*s*_(379) = −0.52, *p* = 2.2×10^-16^. These effect sizes (*r*_s_) are very similar to Experiment 1, replicating the large effect of harmful alcohol use. Similarly, participants with steeper discounting rates also sustained the resource for fewer rounds of the game, *r*_*s*_(379) = −0.34, *p* = 9.33×10^-12^, and gathered fewer rewards, *r*_*s*_(379) = −0.31, *p* = 3.86×10^-10^. Again, these effect sizes are very comparable with Experiment 1, constituting a convincing replication. As in Experiment 1, partial Spearman correlations showed that, when controlling for delay discounting, harmful alcohol use was still significantly associated with fewer rounds lasted, *r*_*s*_(378) = −0.46, *p* = 1.61×10^-16^, and rewards earned, *r*_*s*_(378) = −0.44, *p* = 3.00×10^-18^. For brevity, scatter plots of the raw data can be found in Supplemental Material D.

**Fig. 5:**
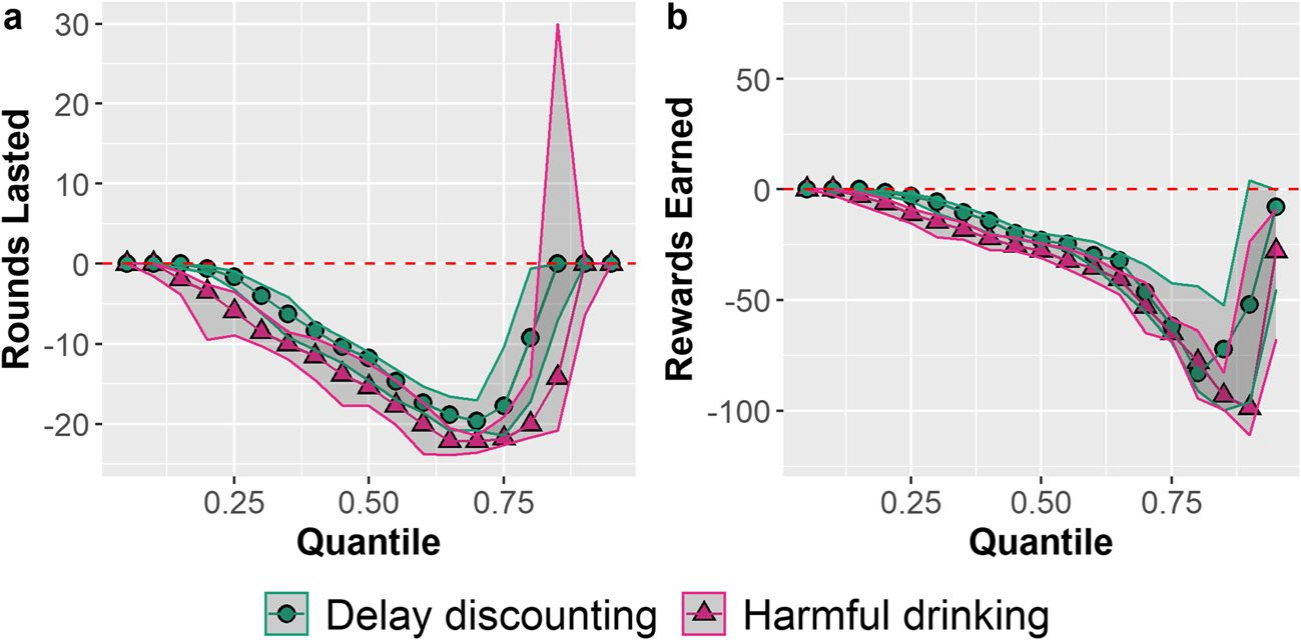
Quantile regression of rounds lasted (left) and rewards gathered (right) against normalized (mean = 0; *SD* = 1) harmful alcohol usage (AUDIT) and delay discounting (ED_50_) from quantiles 0.05–0.95 in increments of 0.05. Shaded areas represent 95% CIs

Finally, we ran quantile regressions to examine these relationships at various levels of performance. As in Experiment 1, each individual characteristic was normalized to a mean of 0 and a standard deviation of 1. Figure 5 shows the relationships between rounds lasted (Fig. 5a), and total rewards gathered (Fig. 5b) on both AUDIT scores and ED_50_ discounting rate.

Here, we found similar relationships compared with Experiment 1. Harmful alcohol use (as measured by AUDIT) was associated with fewer rounds lasted between the 0.15 and 0.80 quantiles and fewer rewards gathered in all quantiles after 0.15. Associations involving delay discounting showed similar patterns. Discounting rates (as ED_50_ scores) were associated with fewer rounds lasted between the 0.30 and 0.80 quantiles and fewer total rewards gathered in all quantiles after 0.30. Please see Supplemental Material D for visualizations of the best fit quantile lines in relation to the raw data.

Finally, across the quantiles, the effect sizes were similar to Experiment 1. For instance, the quantile regression models predict that, at the median, the number of rounds lasted would decrease by 15 for every increase of 1 standard deviation of harmful alcohol use, and by 12 for every increase of 1 standard deviation of delay discounting. Further, at the 0.85 quantile, participants would be expected to gather 93 fewer points for an increase of 1 standard deviation of harmful alcohol use and 72 fewer points for an increase of 1 standard deviation of delay discounting.

## Discussion

Encouraging sustainable use of limited natural, social, and economic resources involves understanding the variety of ways in which people think about how resources work and how they adjust their behaviour (or not) as available resources fluctuate. Interpreting the variation in individual behaviours in group-based, common-pool games (as models of resource management; Balliet, 2009; Brewer & Kramer, 1986) is complicated by the difficulty of disentangling responses to the social aspects of sharing resources with other nonsocial factors. Thus, characterizing varying behaviours and outcomes in single-player games can help us to understand more about how individuals engage with resource ‘temporal dynamics’ (Hendrickx et al., 2001).

Here, for the first time, we show that resource outcomes of a single-player game involving a dynamic resource are moderated by self-reported mental health experiences and delay-discounting rates (as a generic risk factor). In two experiments with large sample sizes, including one direct replication, we find that the ability to sustain a resource over successive harvesting opportunities is reliably worse in individuals reporting elevated psychological distress and the often cooccurring hazardous alcohol use (Lai et al., 2015). Strikingly, these associations remained substantial once we had accounted for elevated delay discounting rates (as a form of impulsivity; Ainslie, 1975), itself a strong risk factor for alcohol misuse and other health problems (Amlung et al., 2019; Fields et al., 2014; Levitt et al., 2023; Petry, 2001; Story et al., 2014). By contrast, individuals who reported higher levels of financial literacy and general well-being achieved correspondingly better resource outcomes.

Our observations suggest that the capacity to respond effectively to the dynamics of resources are compromised in individuals at risk of psychological and alcohol-related disorders. We suggest this work has important ramifications for understanding how individual characteristics moderate behaviour in more complex group settings (e.g. common pools). Managing a resource at the group level involves learning complex multipartner dynamics whilst simultaneously learning non-trivial, time-based resource dynamics (Hendrickx et al., 2001). This work demonstrates that health risk factors are associated with difficulties navigating resource dynamics in a single-player setting, where one does not navigate multipartner dynamics. Future work in understanding how individual characteristics affect group-based resource maintenance should amalgamate the causes underlying variation of individual resource management behaviours with the complex dynamics caused by multipartner interactions.

The variation in resource management behaviours reported here might reflect several—possibly overlapping—psychological mechanisms. First, consistent with evidence of aggressive harvesting behaviours when resources are independently devalued with time (Mannix, 1991), it is likely that individuals with high delay discounting rates will have harvested aggressively earlier in the game in order to secure rewards quickly at the expense of later but larger total rewards afforded by a sustained resource (cf. Bechara et al., 1994). However, other interrelated forms of impulsivity might be involved (Dalley & Robbins, 2017; Evenden, 1999). These include the tendency to act prematurely—in this case, harvesting heavily—without reflecting on the potential damage to the resource (Clark et al., 2006). It might, therefore, be helpful to explore whether interventions to promote future-oriented thinking improve resource management in single-player games in the same way as they have been found to produce transient reductions in discounting rates (Bar, 2010; Benoit et al., 2011) and moderate health-relevant behaviours (Dassen et al., 2016).

Further, the observation that resource outcomes were linked to participants’ psychological distress and alcohol misuse (even after controlling for delay discounting rates) suggests variation in broader cognitive and emotional function linked to psychopathology. Optimal play in our single-player game involves learning about the good and bad outcomes of higher versus lower harvests. However, the motivational processes mediated by this kind of reinforcement learning are likely to be impaired in individuals with anxiety and depression (Browning et al., 2015; Pizzagalli et al., 2008) and subject to broader mood-dependent biases in the processing of positive and negative information (Bradley et al., 1995; Hamilton & Gotlib, 2008; Mennen et al., 2019). Similarly, reward-based learning can also be compromised in groups with alcohol-use difficulties (Cao et al., 2021; Park et al., 2010). In the present experiments, we used validated self-report measures to identify increasing risk of psychological disorders. Further work in clinical samples with established diagnoses for mood, anxiety, or alcohol-use disorders could clarify whether difficulties engaging with resources are linked to symptom severity, persist in the euthymic or remitted state, or are linked to broader difficulties in managing financial, social, and clinical resources in patients’ lives (Adinoff et al., 2016; Haushofer & Fehr, 2014; Richardson et al., 2017). Finally, the positive links seen here between resource outcomes and financial literacy suggest that the effective use of the resource in our single-player game is linked to broader patterns of planning and cautious decision-making in household and financial contexts, as captured by the OECD measure of financial literacy (Čonková, 2014).

These experiments do have limitations. First, our findings involved a single-player game with a specific initial resource value (60 nominal rewards) and a particular replenishment value (15±3%). We cannot yet know whether the pattern of harvesting behaviours observed and their modulation by health experiences might be different with different game parameters. Future work could consider the resource parameters which constitute the boundary conditions for these patterns. Second, although our findings show that resource management outcomes are linked to participants’ delay discounting rates, the replenishment mechanism in our single-player game involved stochastic additions to the resource. Therefore, in future work, it might be helpful to explore whether harvesting behaviours are also moderated by individuals’ probability discounting rates as a distinct construct (Green & Myerson, 2013).

Third, the use of a stochastic replenishment function meant that participants experienced slightly different fluctuations in the resource. While the resource replenished at 15% on average, some participants would have seen slightly higher or lower replenishments rates compared with others. This could have influenced behaviour in the game—for example, some participants might have experienced low replenishments early while they were learning about the vulnerability of the resource. To test whether resource outcomes reflected variation in experienced replenishment rates, we calculated, for each participant in each experiment, the mean replenishment rate over the first 1, 2, 3, 5, 10, 20, 50, and 70 rounds. We then ran Spearman correlations between the mean replenishment rates across participants and both rounds lasted and total rewards gathered. None of the effects were substantial or statistically significant (see Supplemental Material E for the detailed analysis). Therefore, it seems unlikely that the participants’ behaviour in the games was linked to variance in replenishment experiences.

Fourth, in these studies, participants did not know the resource management game would end after 70 rounds. Further, previous research suggests that, in other contexts, manipulating uncertainty around the termination of games can affect risk and delay-related choices (Bigoni et al., 2015; Pietras et al., 2003; Pietras & Hackenberg, 2001). As such, it is possible that the poor sustainable behaviour observed here among individuals with health risk factors was due to poor navigation of the uncertain termination rule of the game. In other words, perhaps those with health risk factors would manage a resource better if they were certain the resource game would not end abruptly. Using previously unanalyzed data from another study, we found that this was not likely to be the case (see Supplemental Material F for complete analysis). Here, 100 participants played the same single-player resource management game but with the number of rounds remaining in the game displayed before each harvest. These participants also completed the ED_50_ measure of delay discounting. As above, the number of rounds sustained and the total rewards gathered were strongly and negatively associated with discounting rates. In fact, the strength of these correlations were larger when participants knew the termination rule of the game compared with when they did not know, as in Experiments 1 and 2. Thus, the associations involving delay discounting rates are not an artefact of participants’ lack of knowledge of the game’s termination rule.

Finally, there remain important questions about the direction of causality in the poor resource outcomes of individuals reporting adverse health experiences. Possibly, as above, the behaviours seen in the present experiments directly reflect changes in the cognitive and affective mediators of symptom severity. However, equally, social and economic difficulties are linked to elevated rates of depression, anxiety and alcohol misuse (Haushofer & Fehr, 2014; Makela, 1999; Marmot, 1999; Richardson et al., 2017; Sze et al., 2017). Prolonged, psychosocial pressures of this kind can undermine resilience, possibly promoting ‘fast-life strategies’ that, in a context of uncertainty and scarcity, prioritize the rapid consumption of resources which can further elevate stress and the likelihood of relapse or symptom severity (Pampel et al., 2010; Pepper & Nettle, 2017). As such, it may be helpful to focus future research on psychoeducation interventions to include content about resource dynamics to support individuals reporting psychological distress in the effective management of their financial, social, and clinical resources in order to protect their well-being. It may also be helpful to tailor policy interventions to encourage sustainable use of resources, allowing for the difficulties that clinical groups may experience with resource dynamics.

### Supplementary Information

Below is the link to the electronic supplementary material.

Supplementary file1 (DOCX 14.1 MB)
