# Supplementary material for: Health factors that influence sustainable behaviour in a single-player resource management game
Source: Psychon Bull Rev. 2023 Sep 15;31(2):734–49. doi: 10.3758/s13423-023-02341-y (PMC11061002; doi:10.3758/s13423-023-02341-y)
Supplement: Supplementary file 1 — Supplementary file1 (DOCX 14.1 MB) [file 13423_2023_2341_MOESM1_ESM.docx]

**Supplemental Material A– Materials**

Below is a detailed description of the experimental protocols for both experiments, including screenshots of each stage of the procedure in each experiment.

***Experiment 1***

**Participant information page and consent form:**

Prior to completing the protocol, the participant needed to consent to participate. To do this, each participant was shown the below Information Page. At the end of the page, they agreed to several bullet points (e.g., that they knew they could quit at any point by closing the experiment). The participant agreed to the consent form by clicking ‘Accept’ at the bottom of the page.


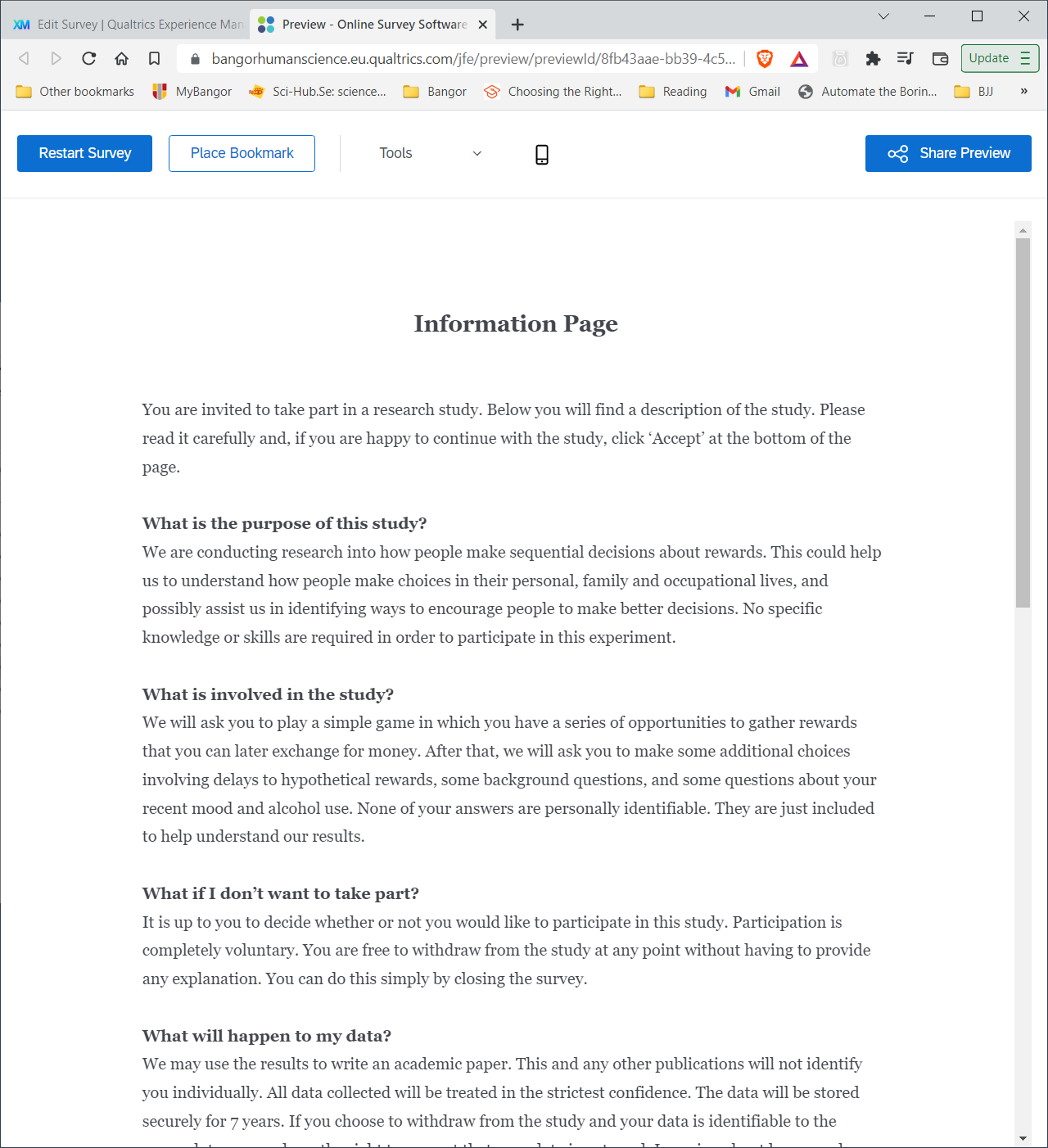


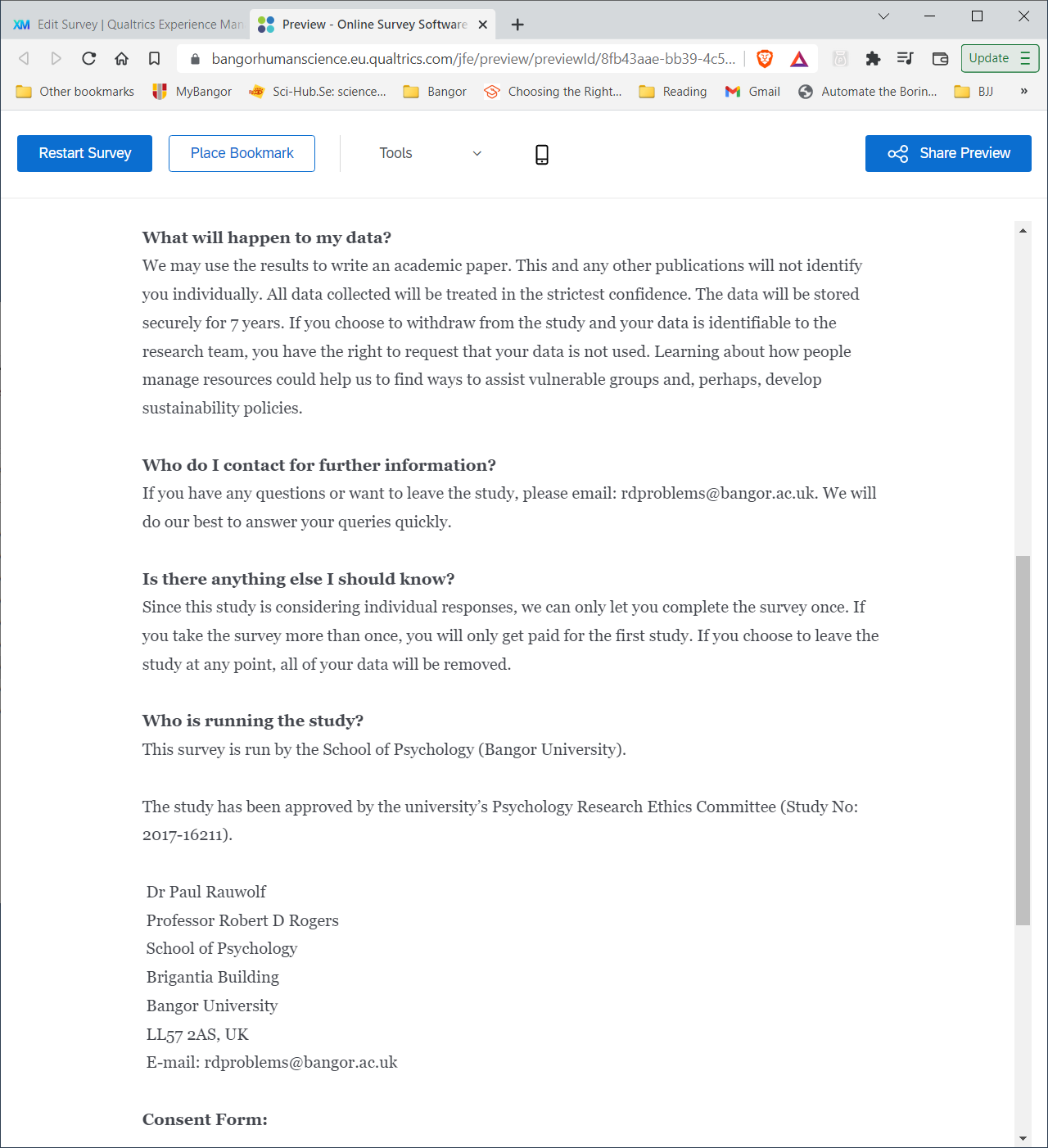

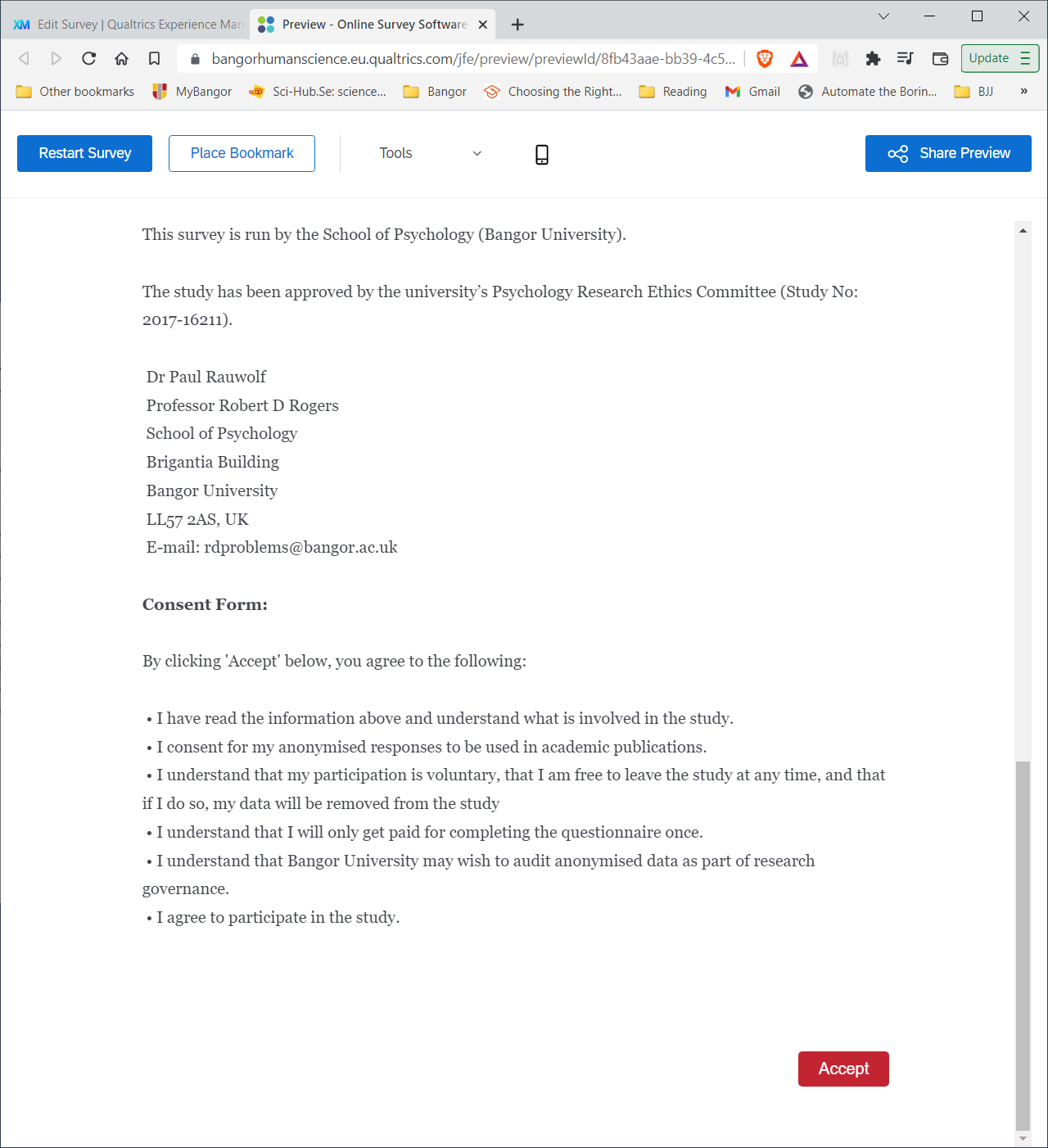


**Participant demographic information:**

Next, we asked participants some demographic questions.


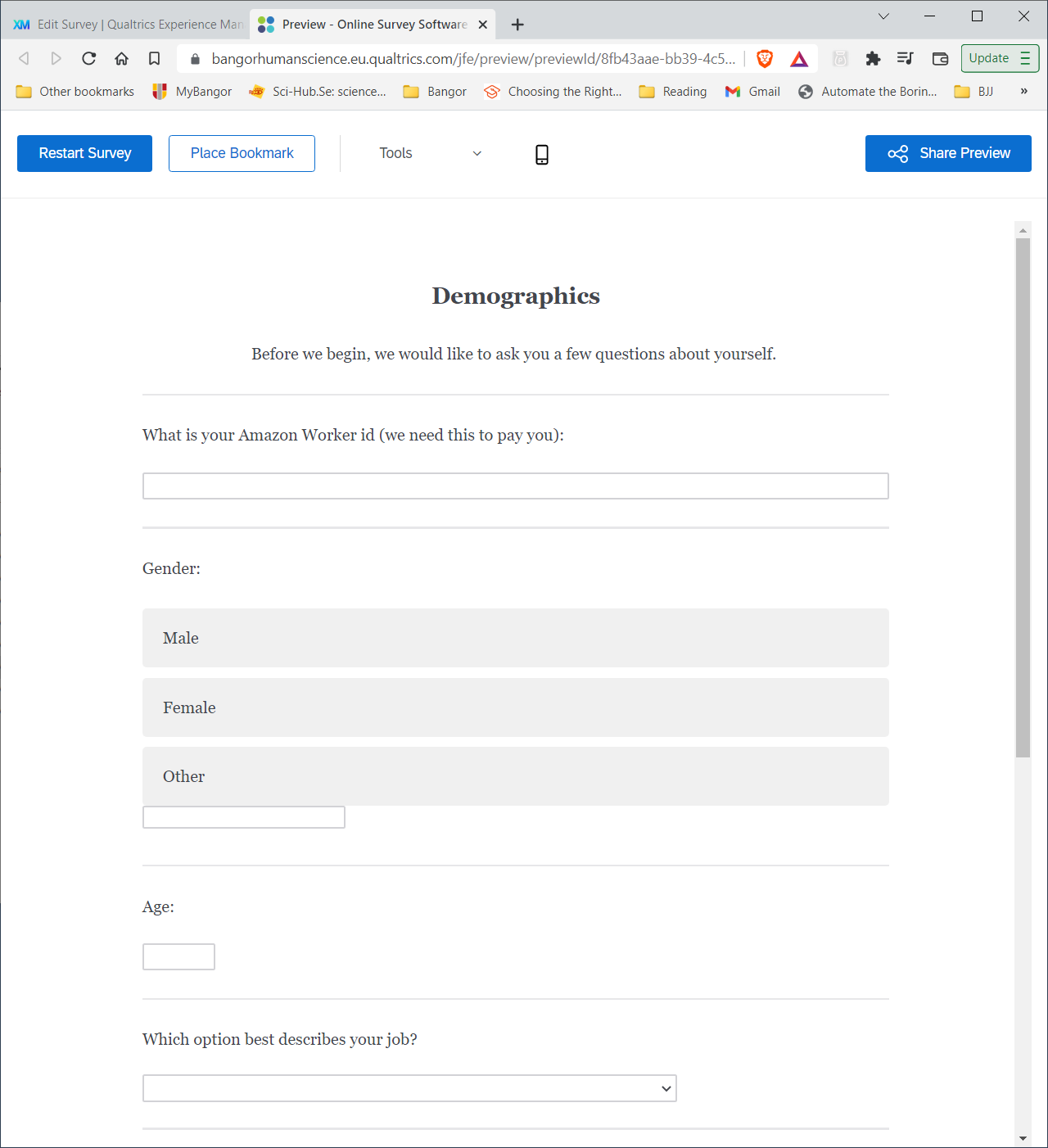


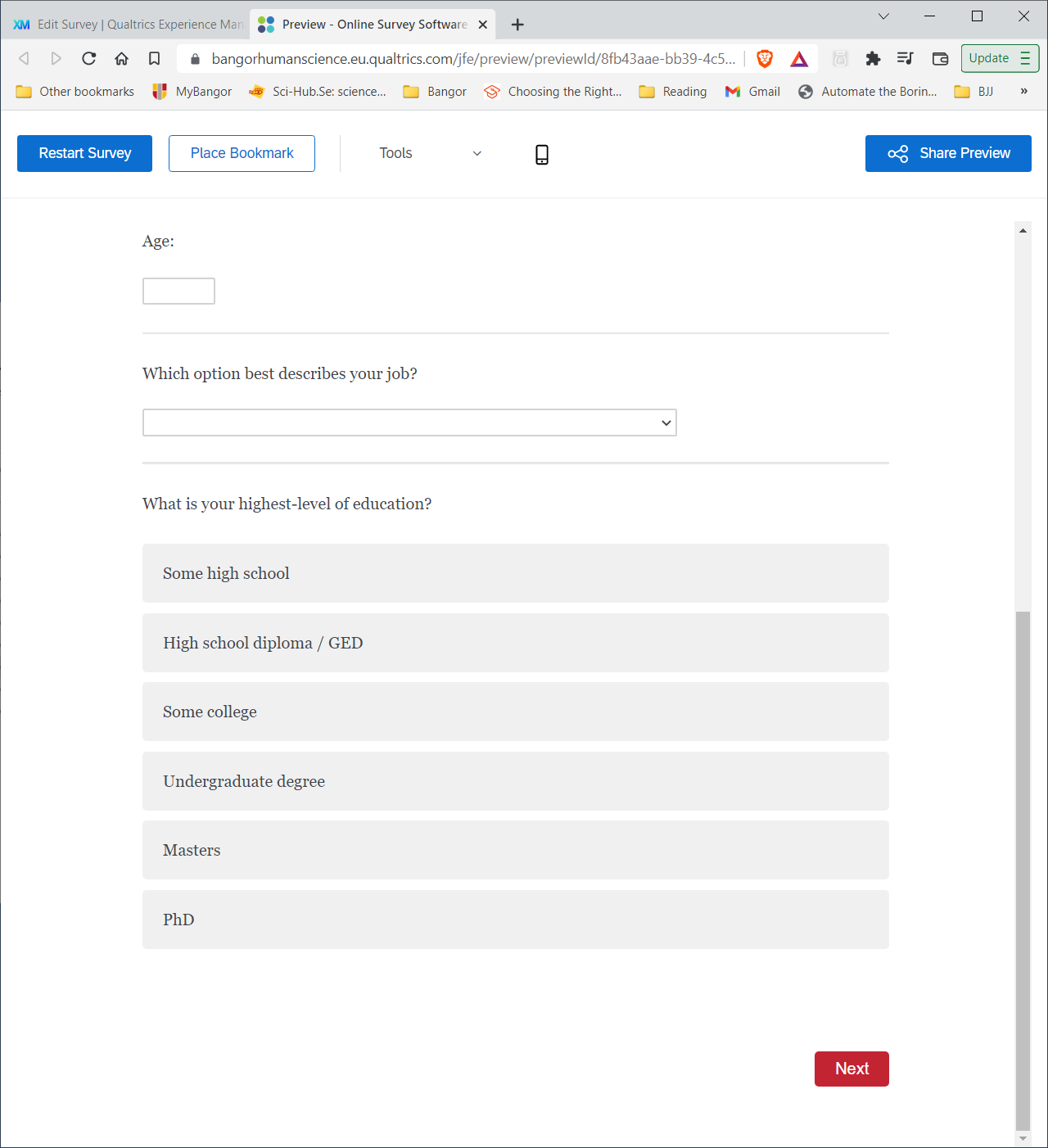


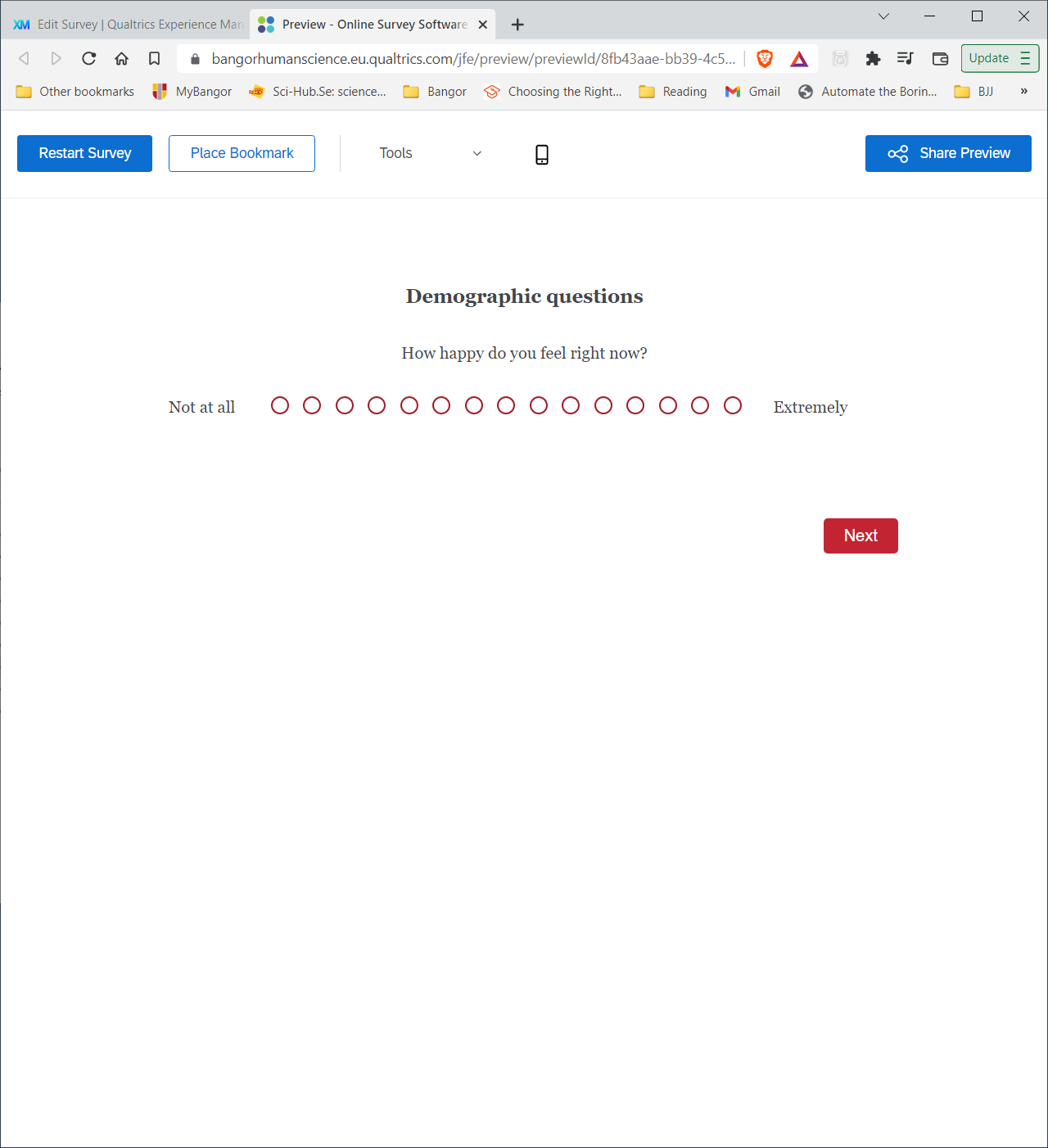


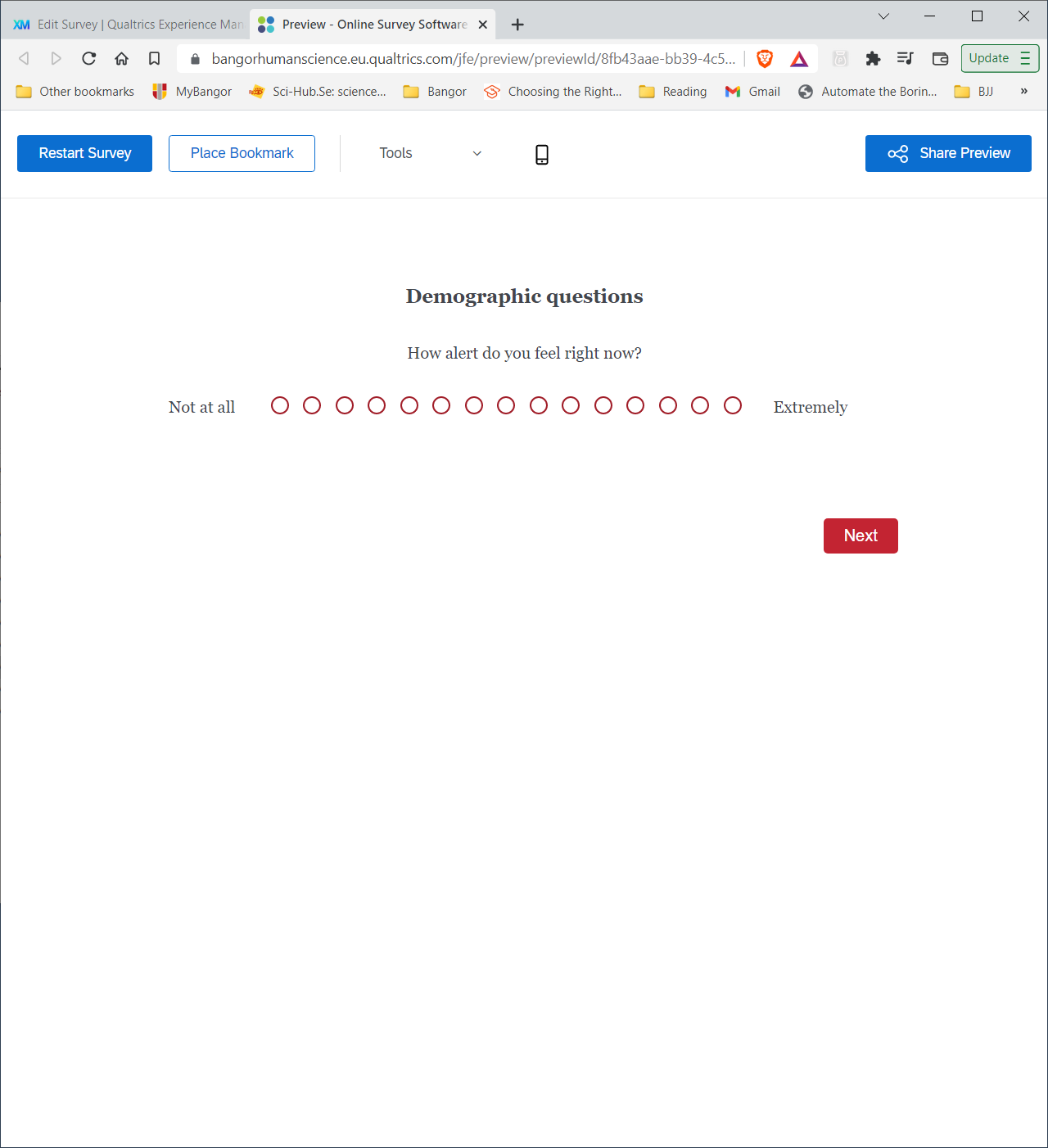


**Instructions:**

Before participants played the game, we offered a tutorial about how the game works and what they were expected to do. We did not tell the participants the replenishment rate, maximum number of rounds, or monetary value of each reward earned. Participants were only told that they could earn a maximum of $3 for their play in the game.


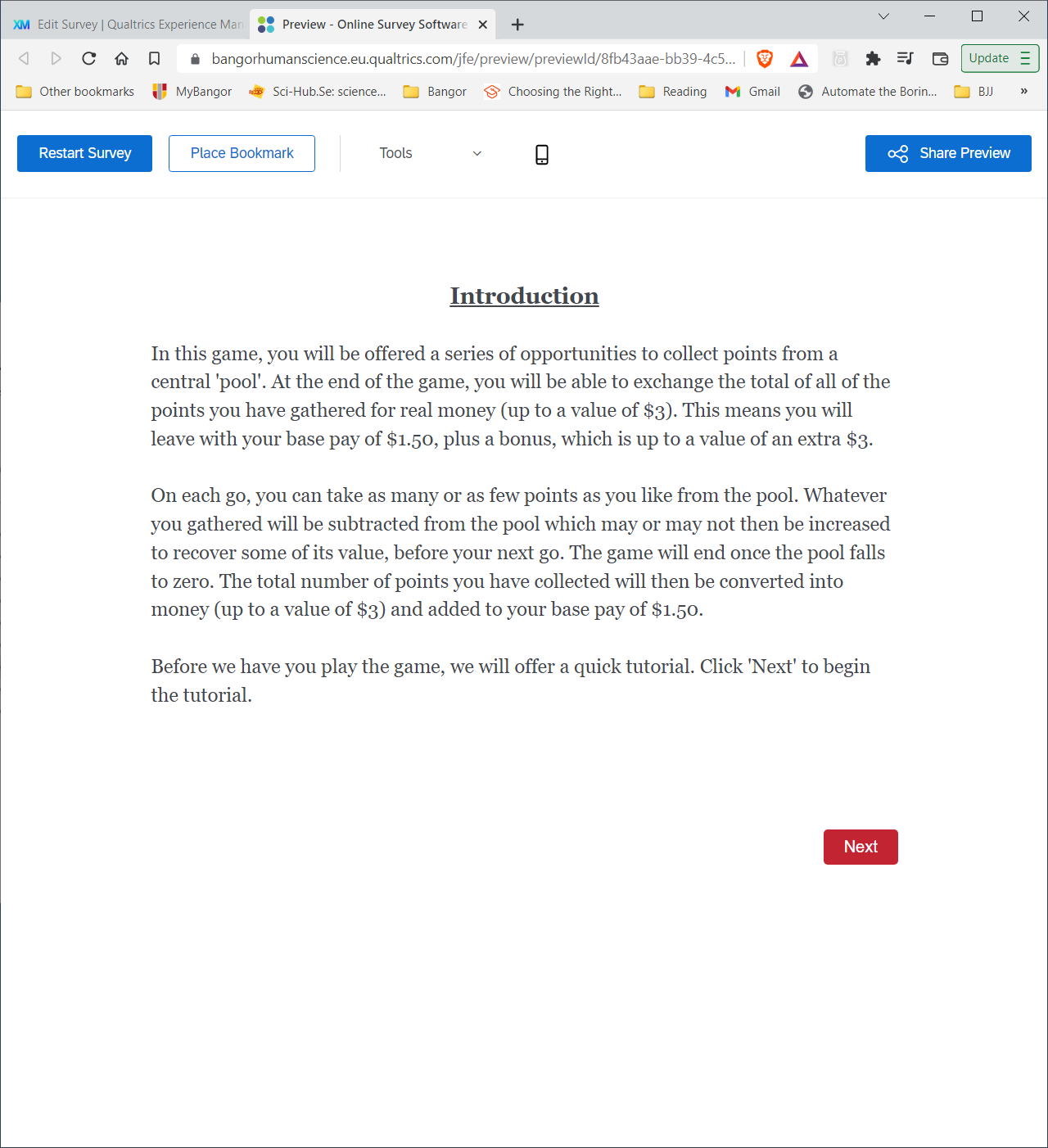


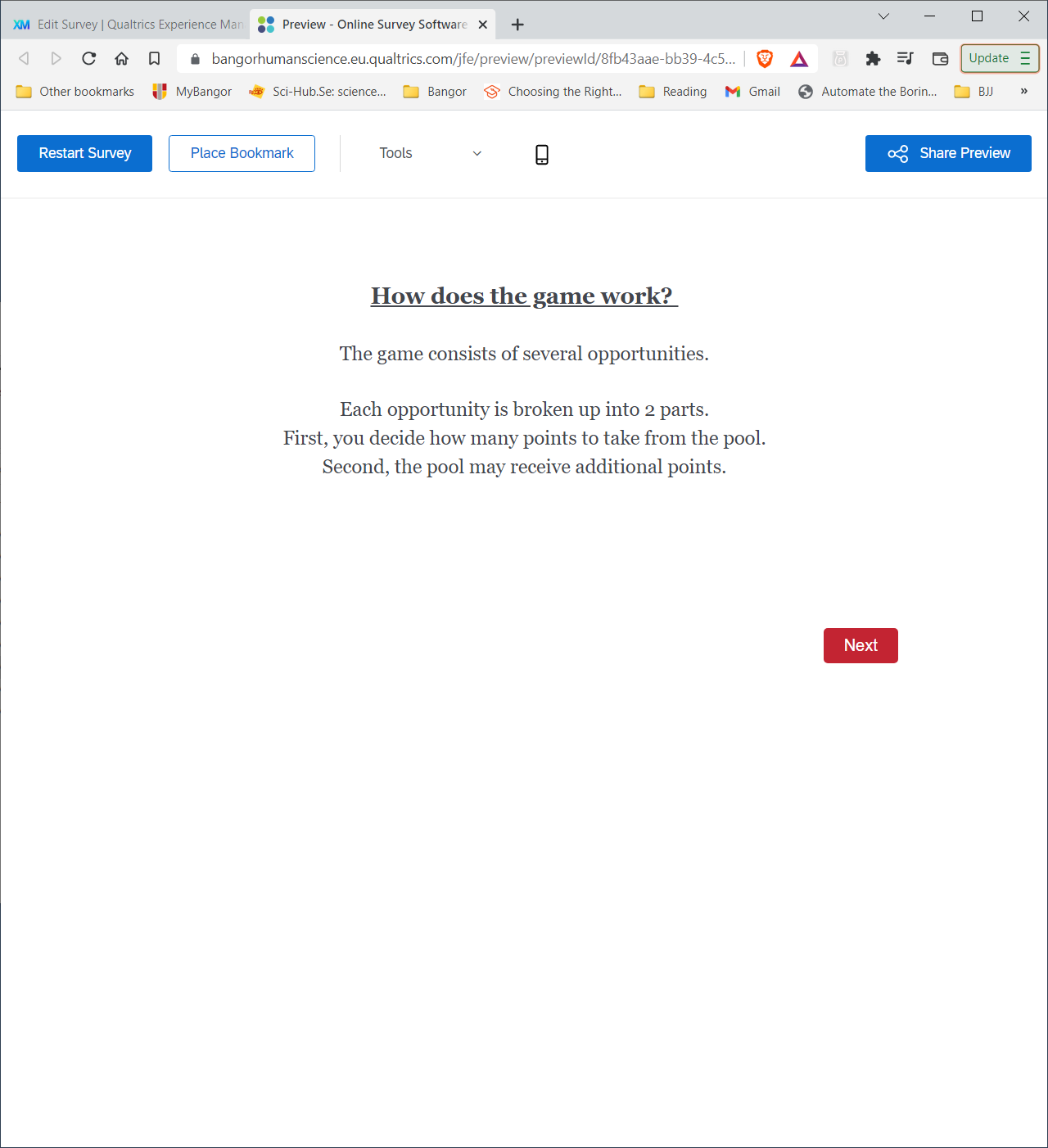


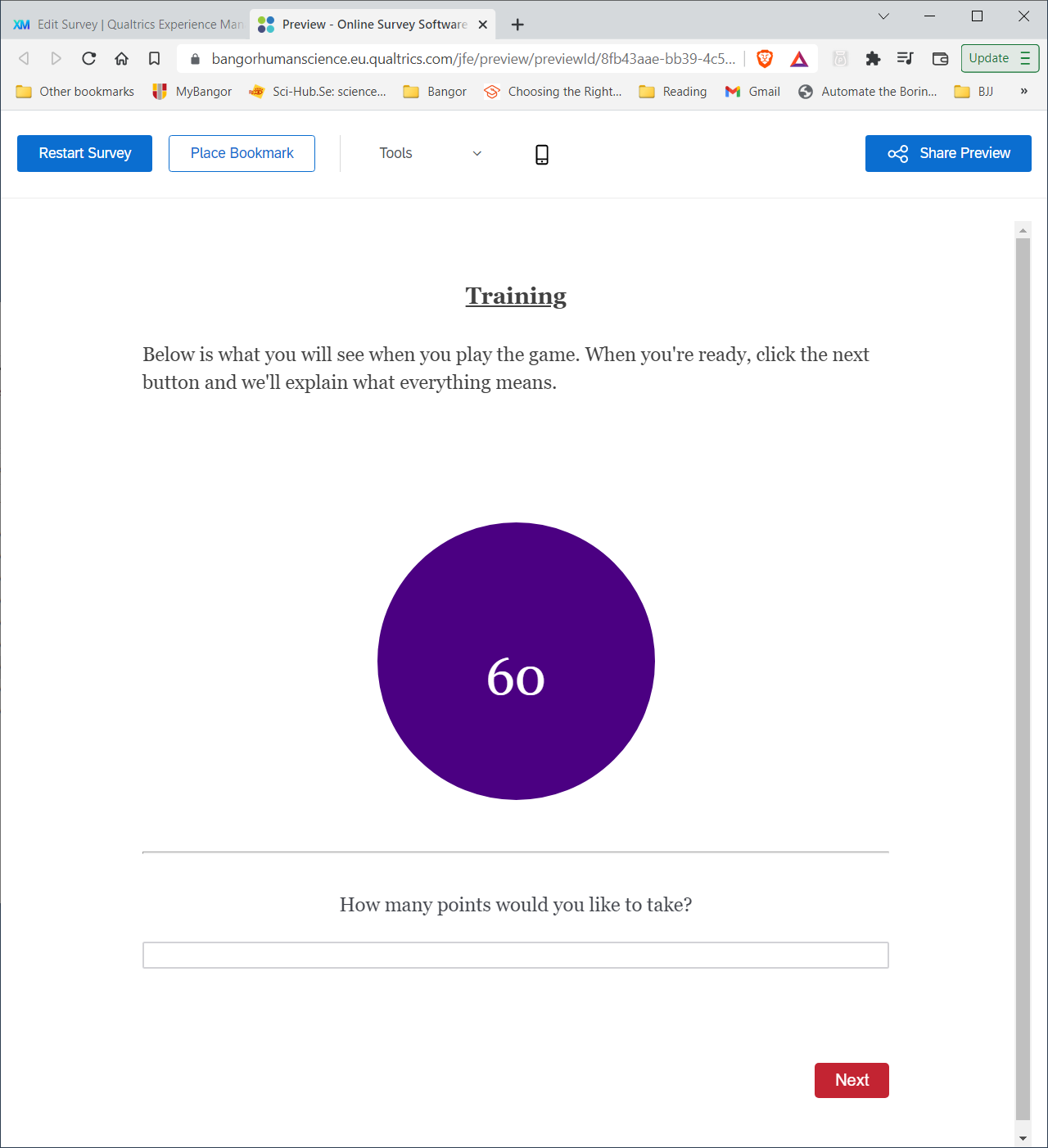


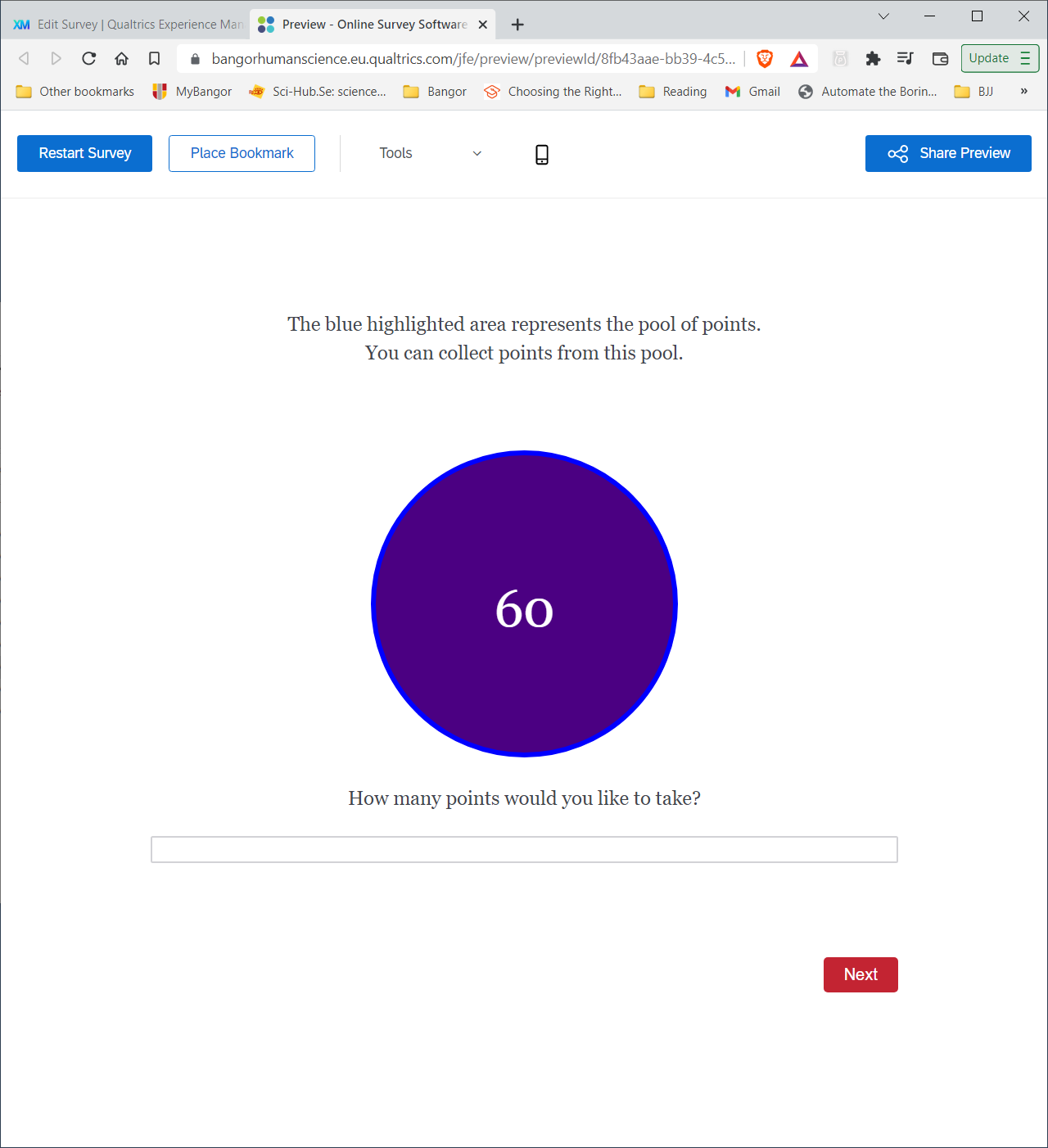

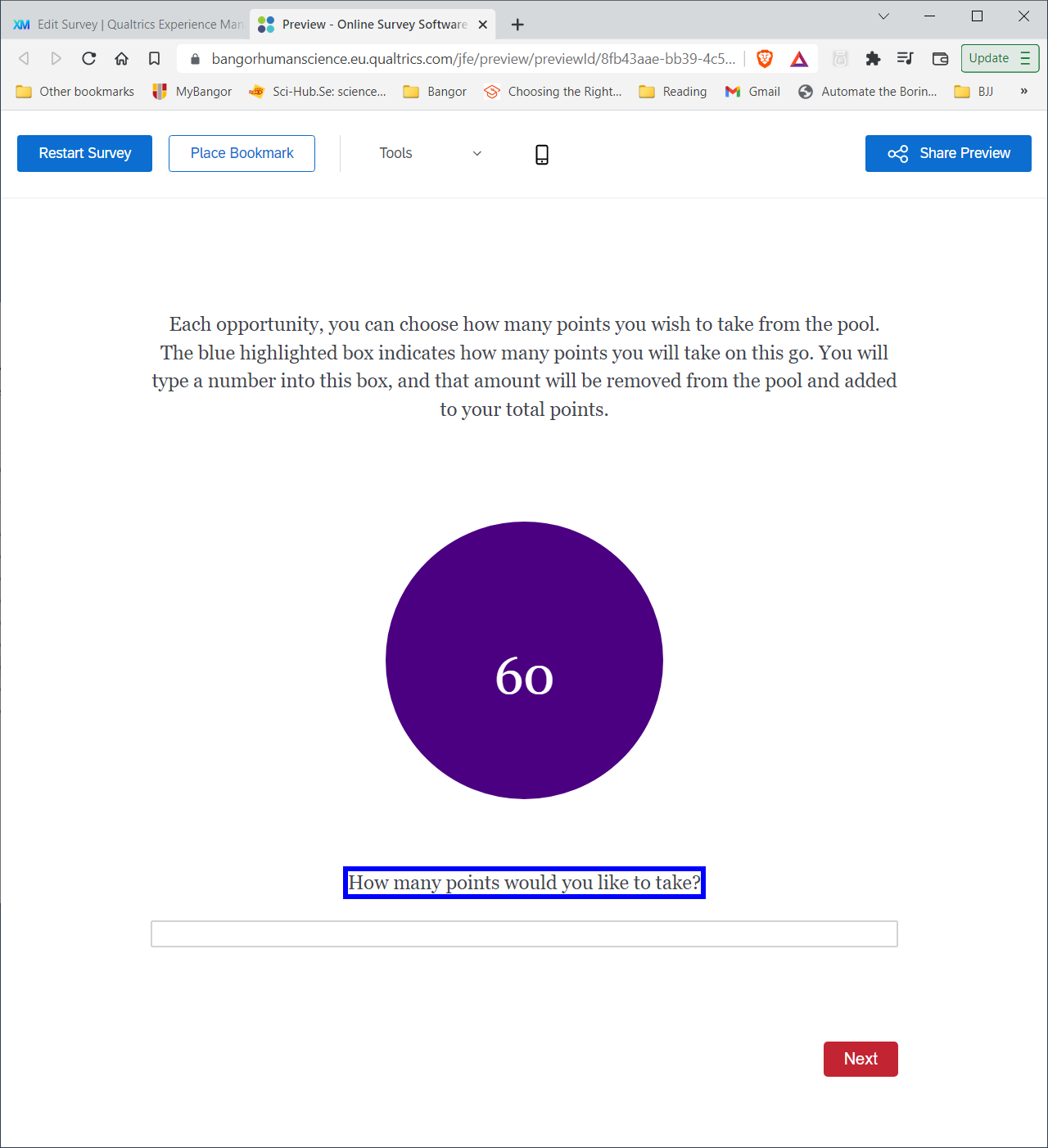

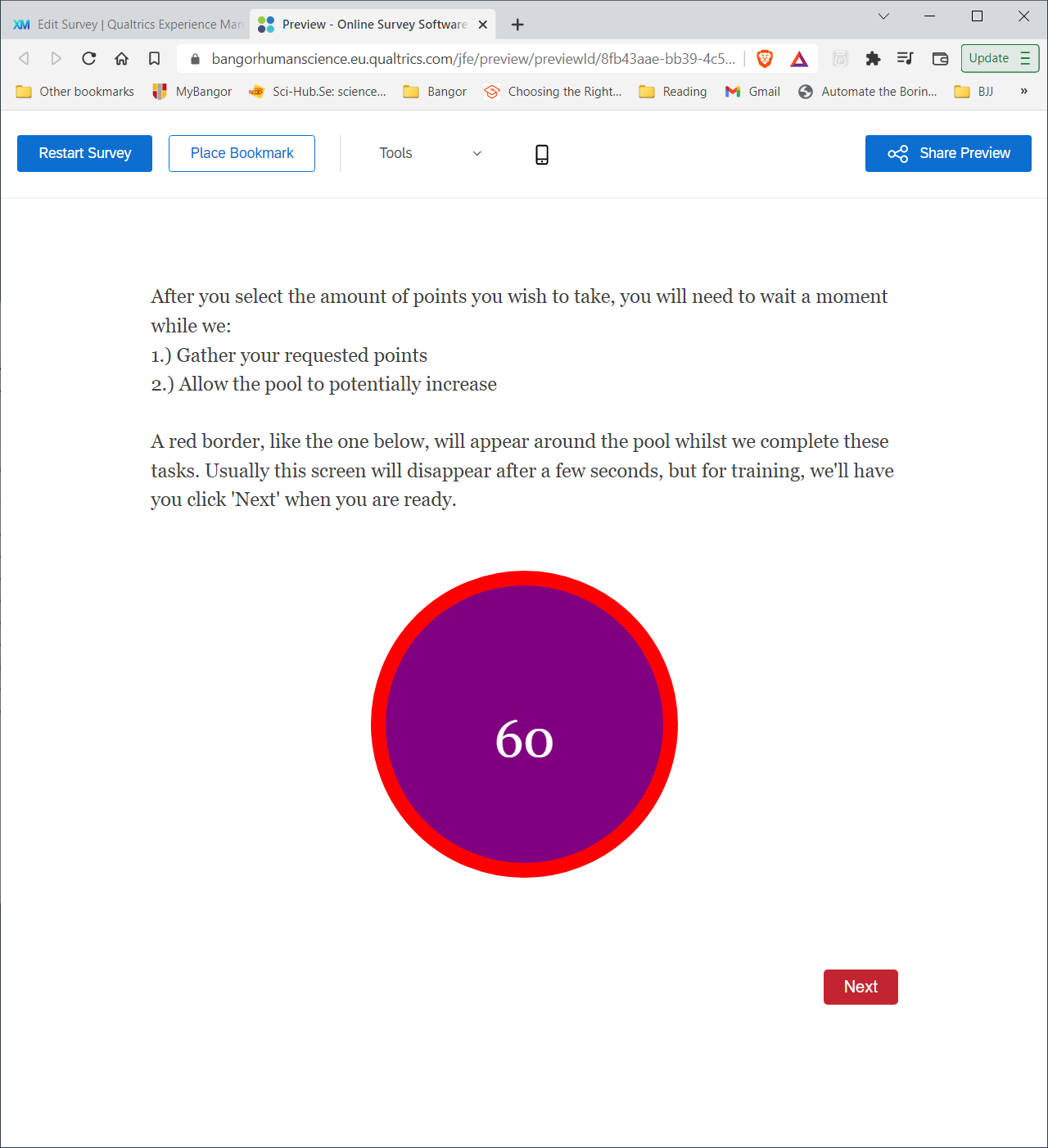


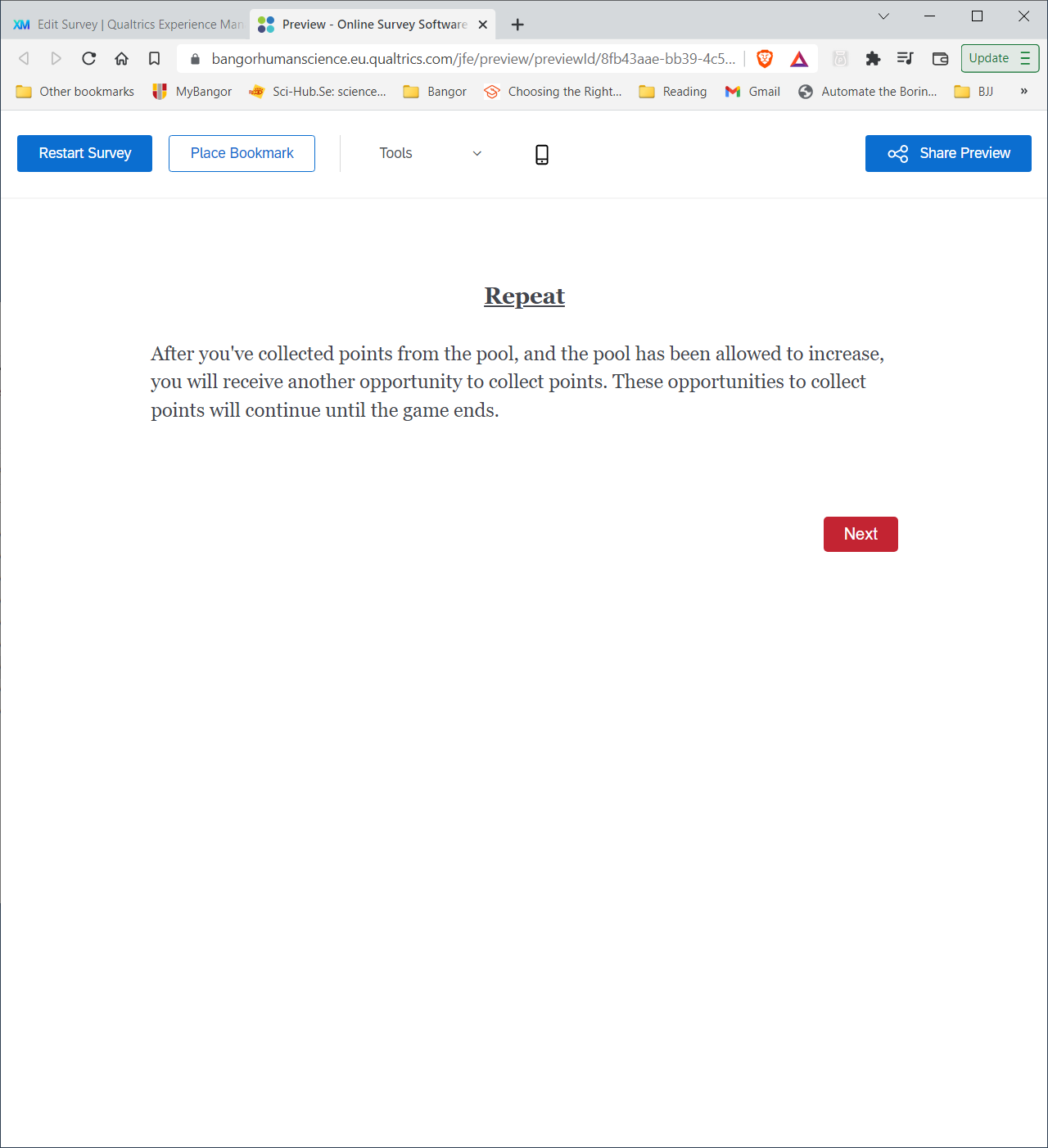


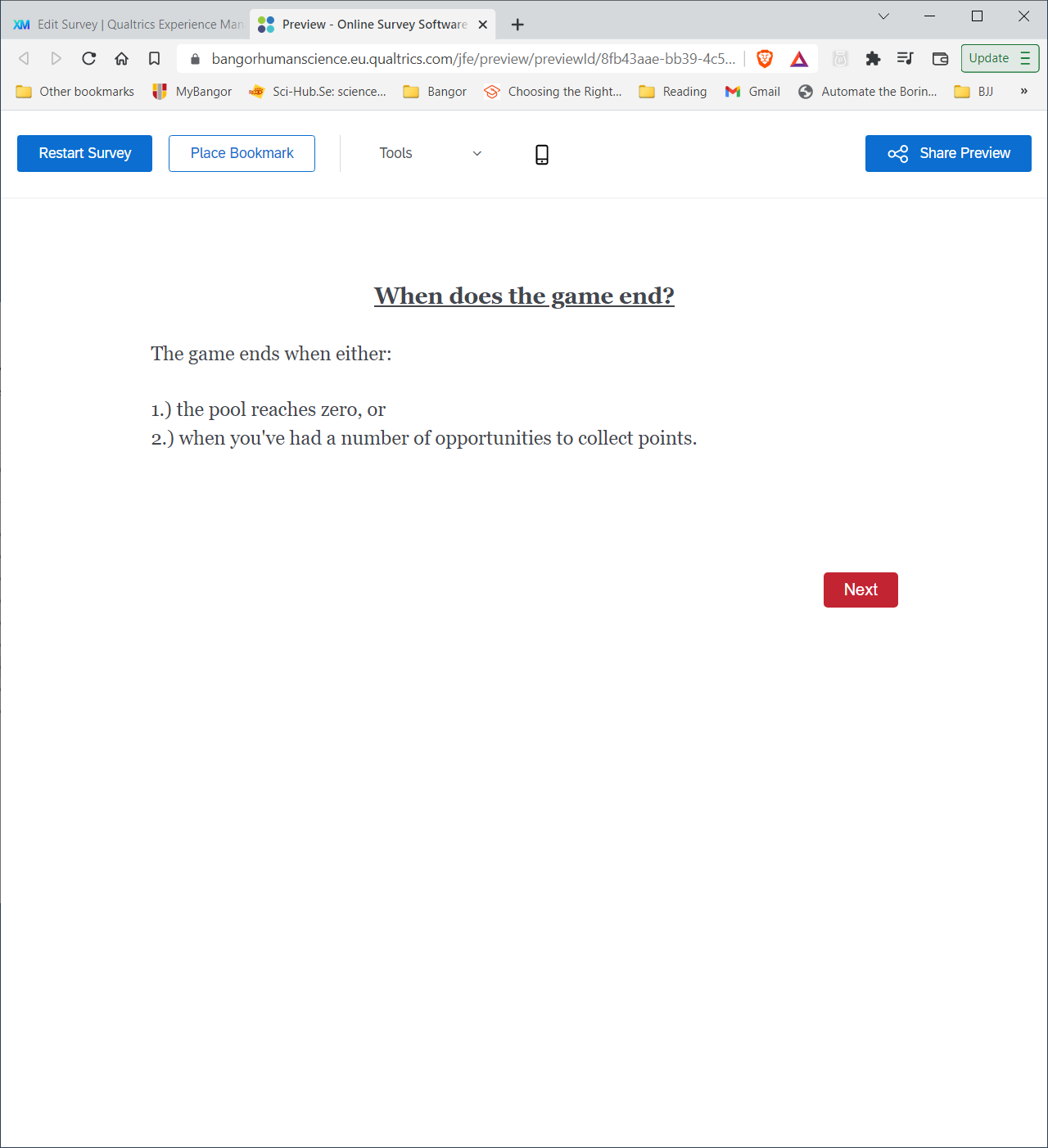


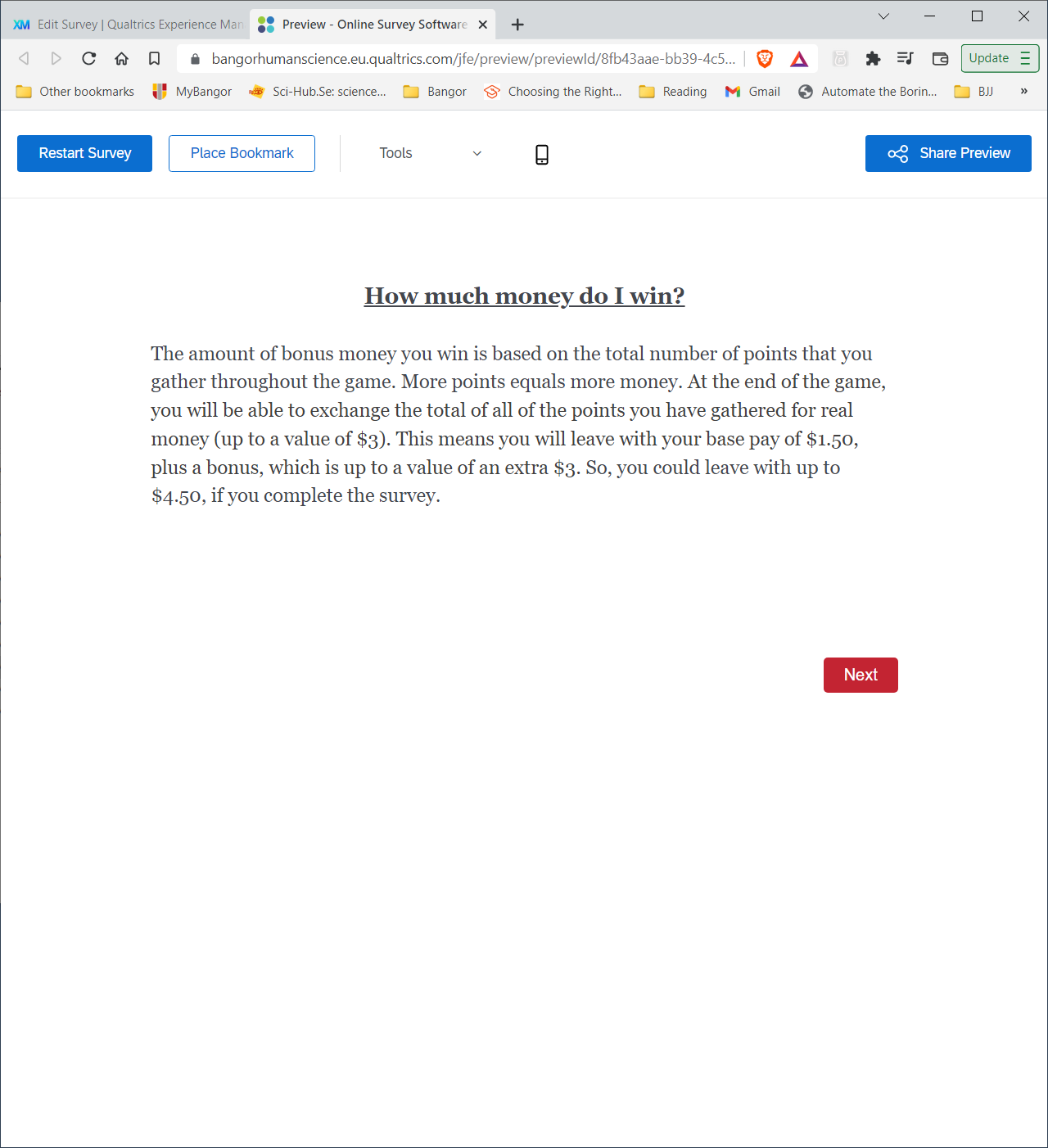


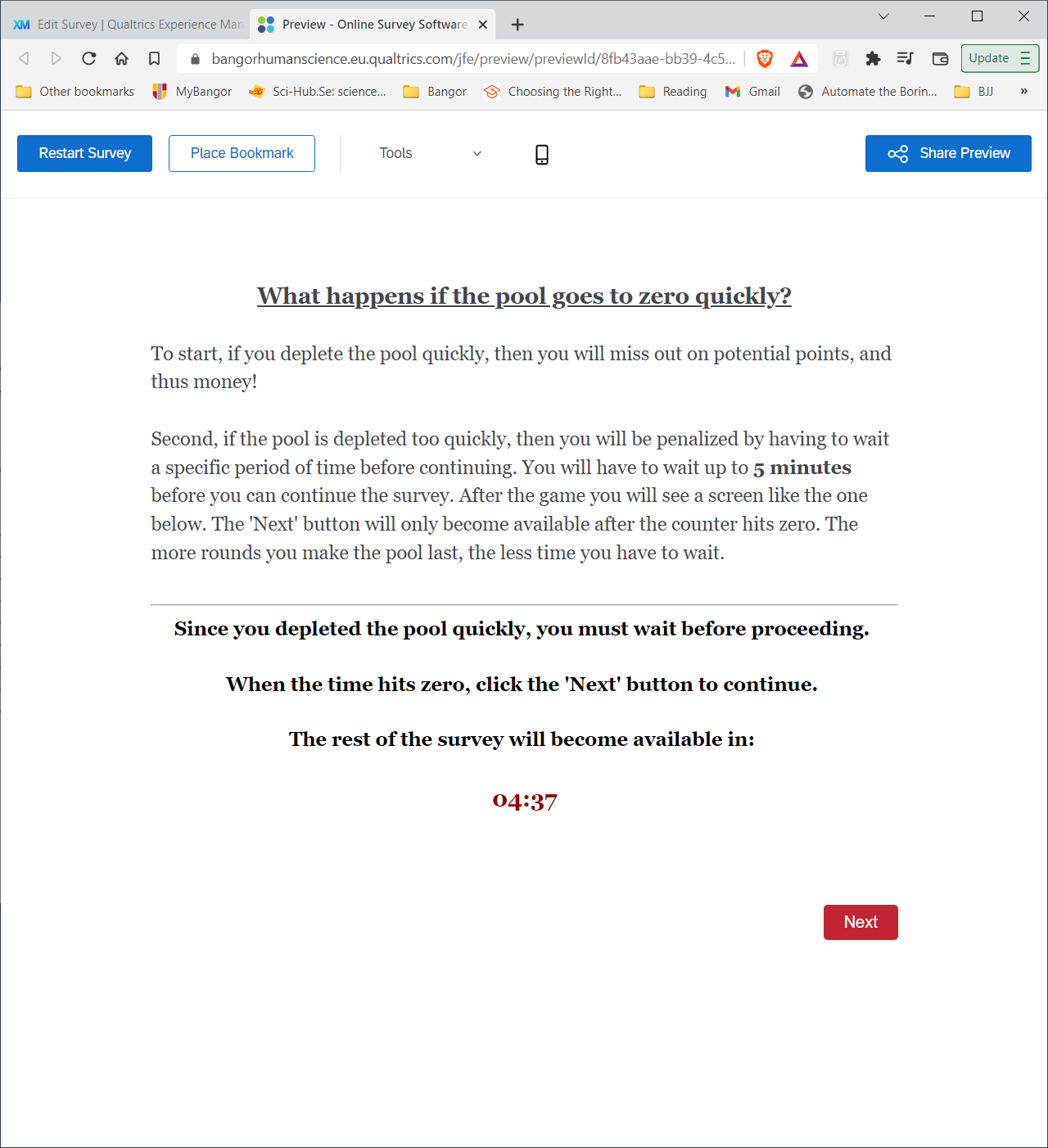


**Questions to assess participant understanding:**

Here, we asked participants three multiple-choice questions to assess their understanding of the game. Participants could not continue past this page until they had selected the correct answer for each of these questions. This page also contained all the instructions previously given to the participant, to revise again if necessary. We haven’t included screenshots of this, as the instructions are identical to those shown above.


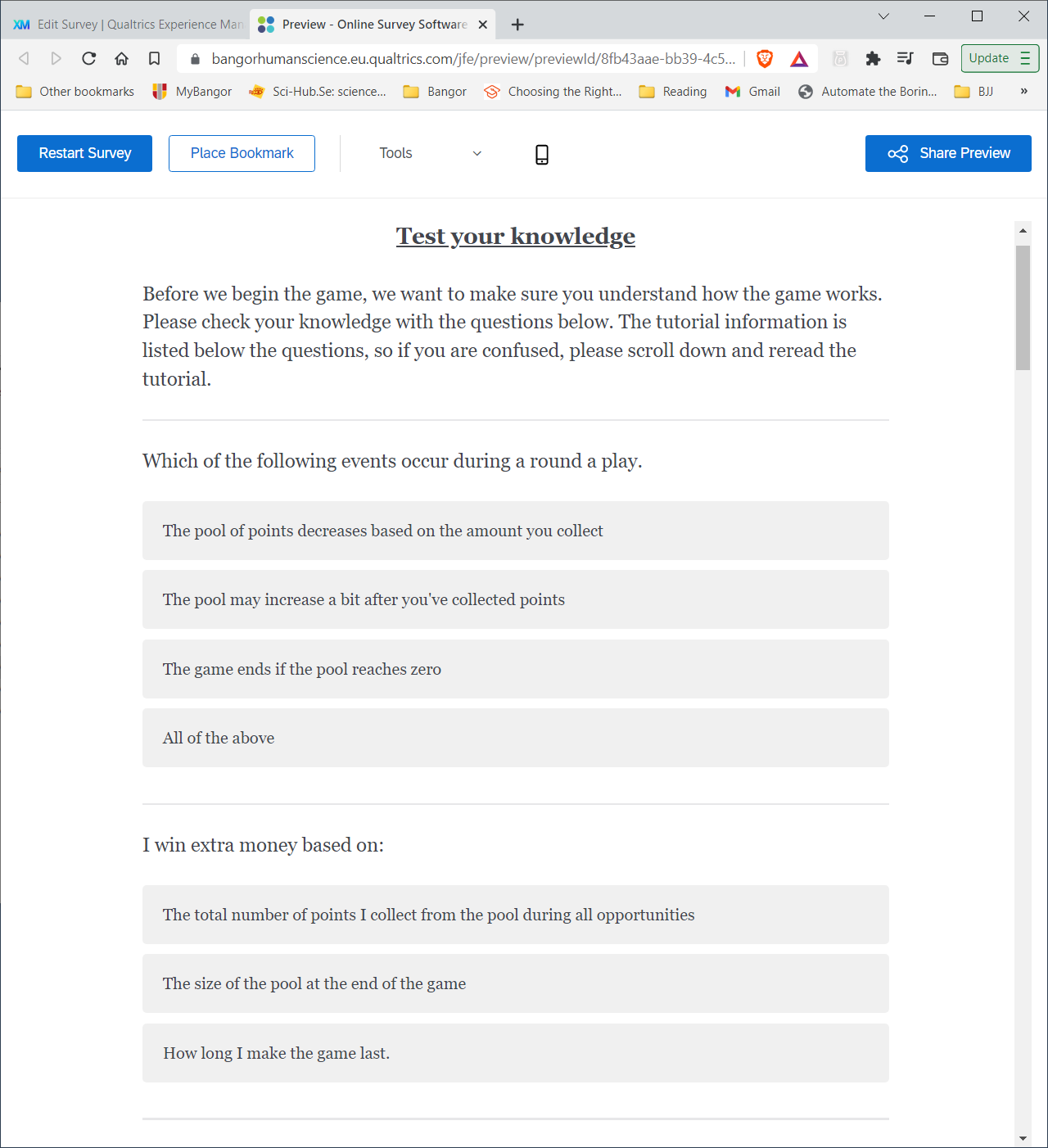


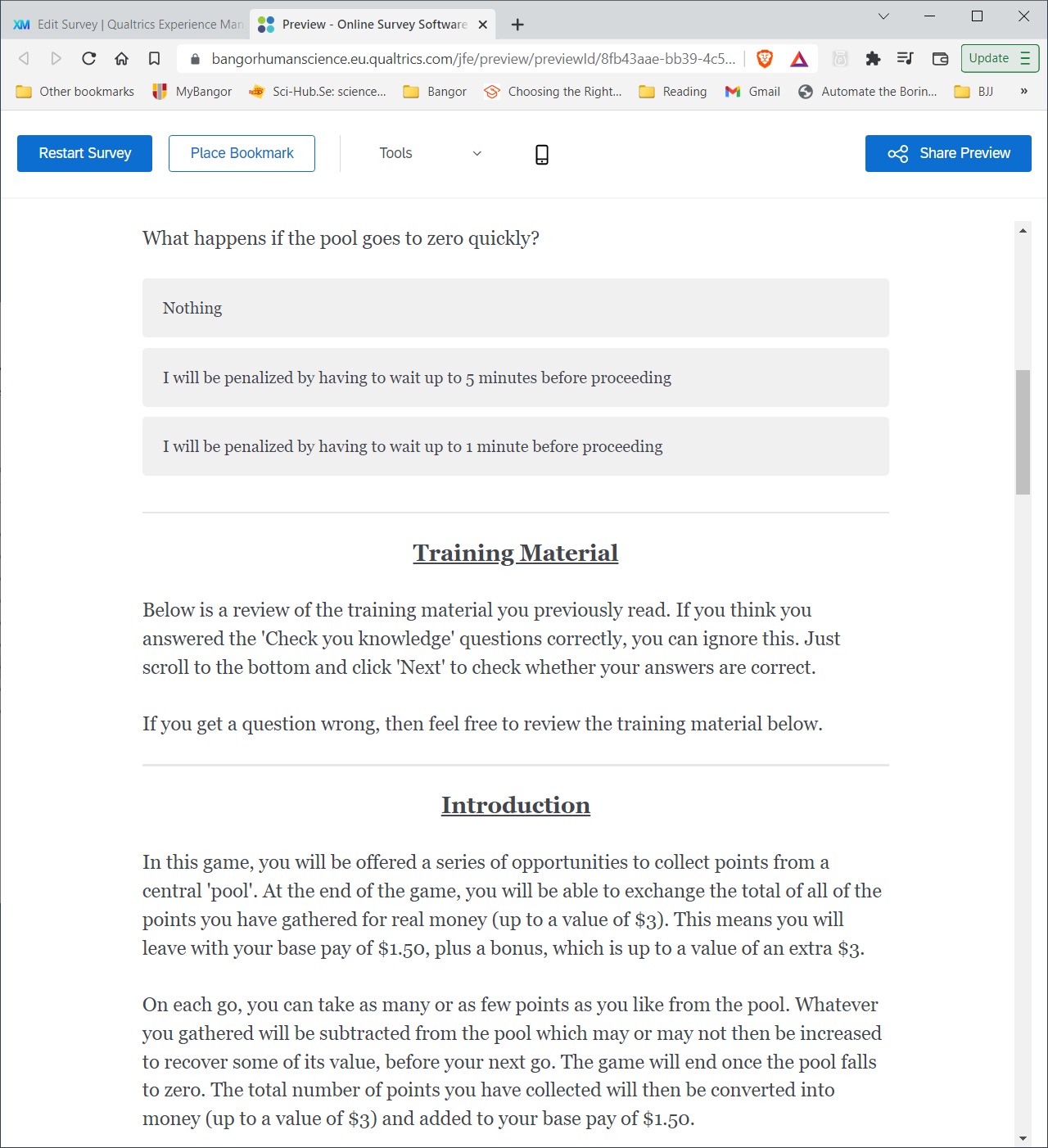


**Playing the game:**

Once they had answered the ‘Test your knowledge’ questions correctly, participants played the game. In these screenshots, we show an example of a participant who lasted two rounds in the game.


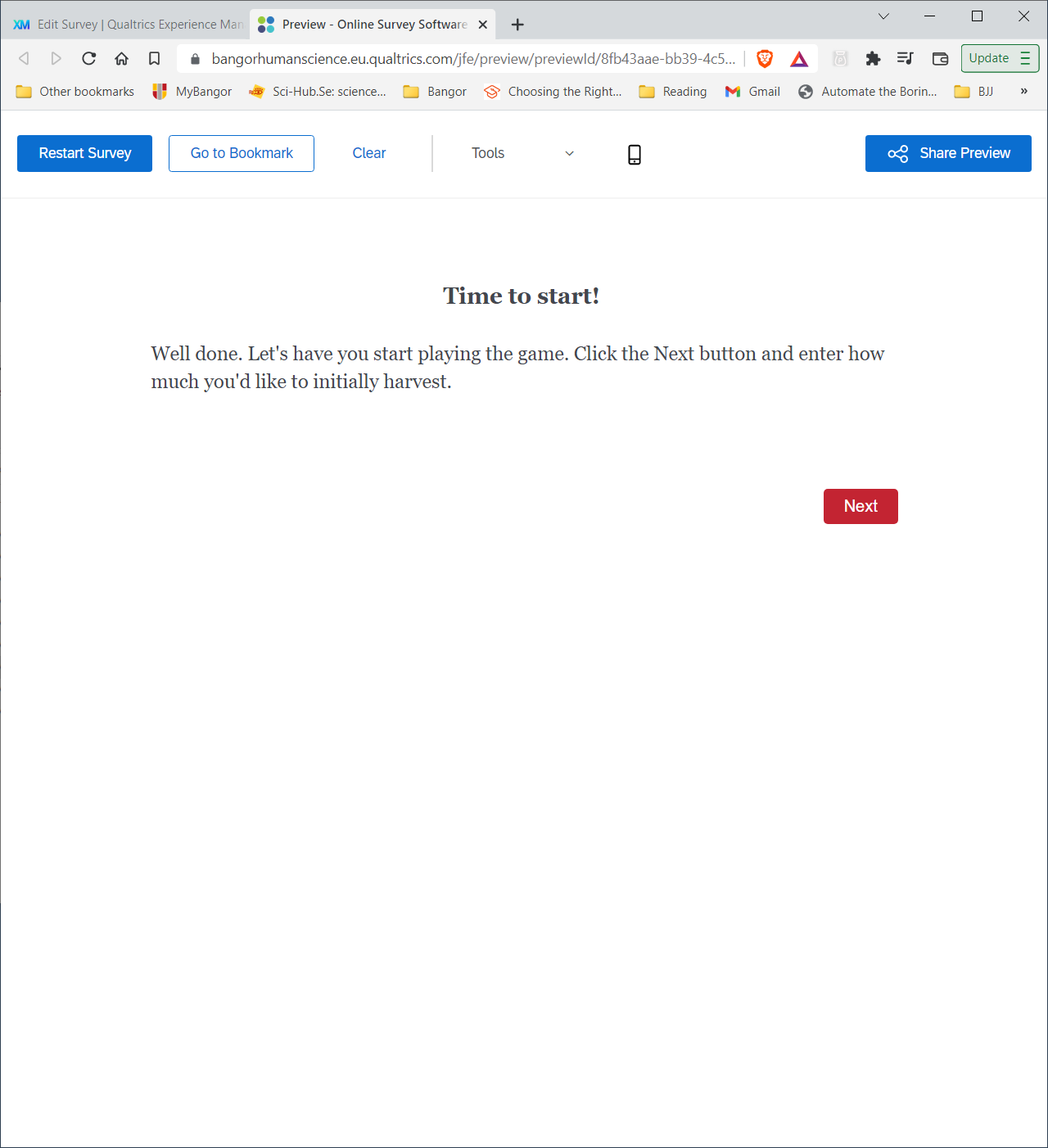


The resource always started at its maximum capacity of 60. Participants typed the number of rewards they wanted to harvest into the text box, and then clicked ‘Next’ to harvest these

rewards.


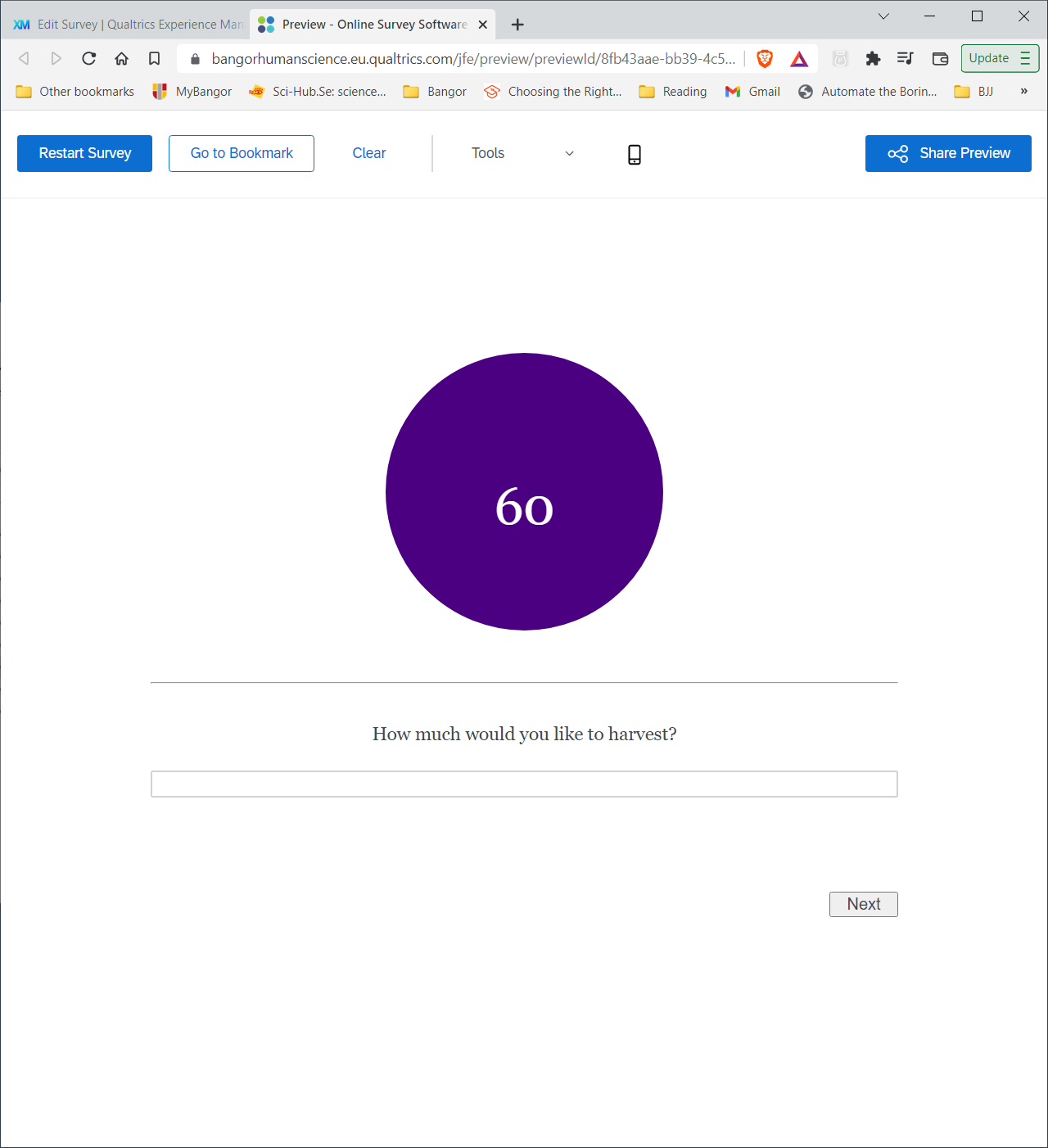


As an example, a participant might take 30 rewards from the resource in the first round.


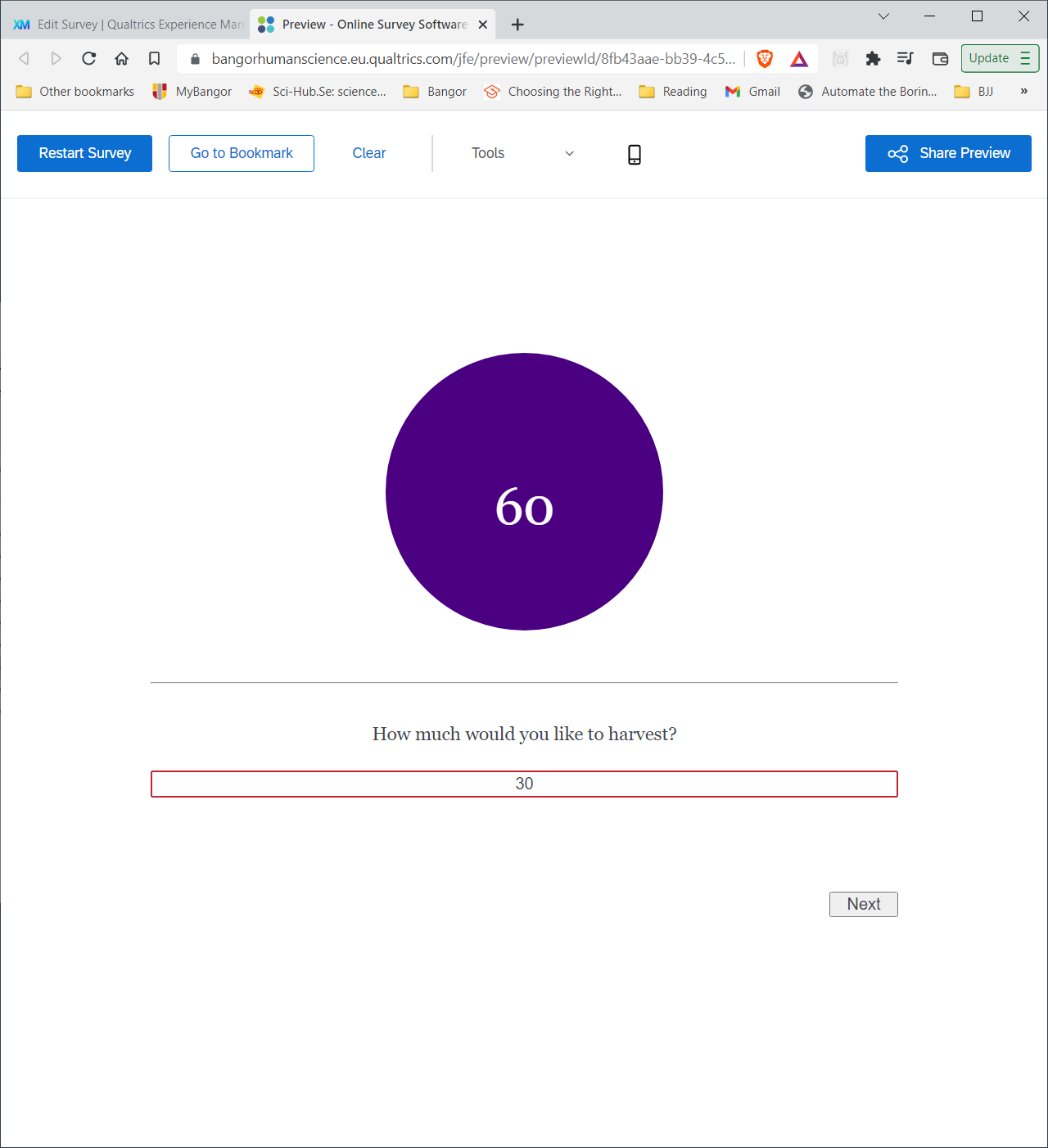


Once the participant clicked ‘Next’, they would see this page for a couple of seconds.


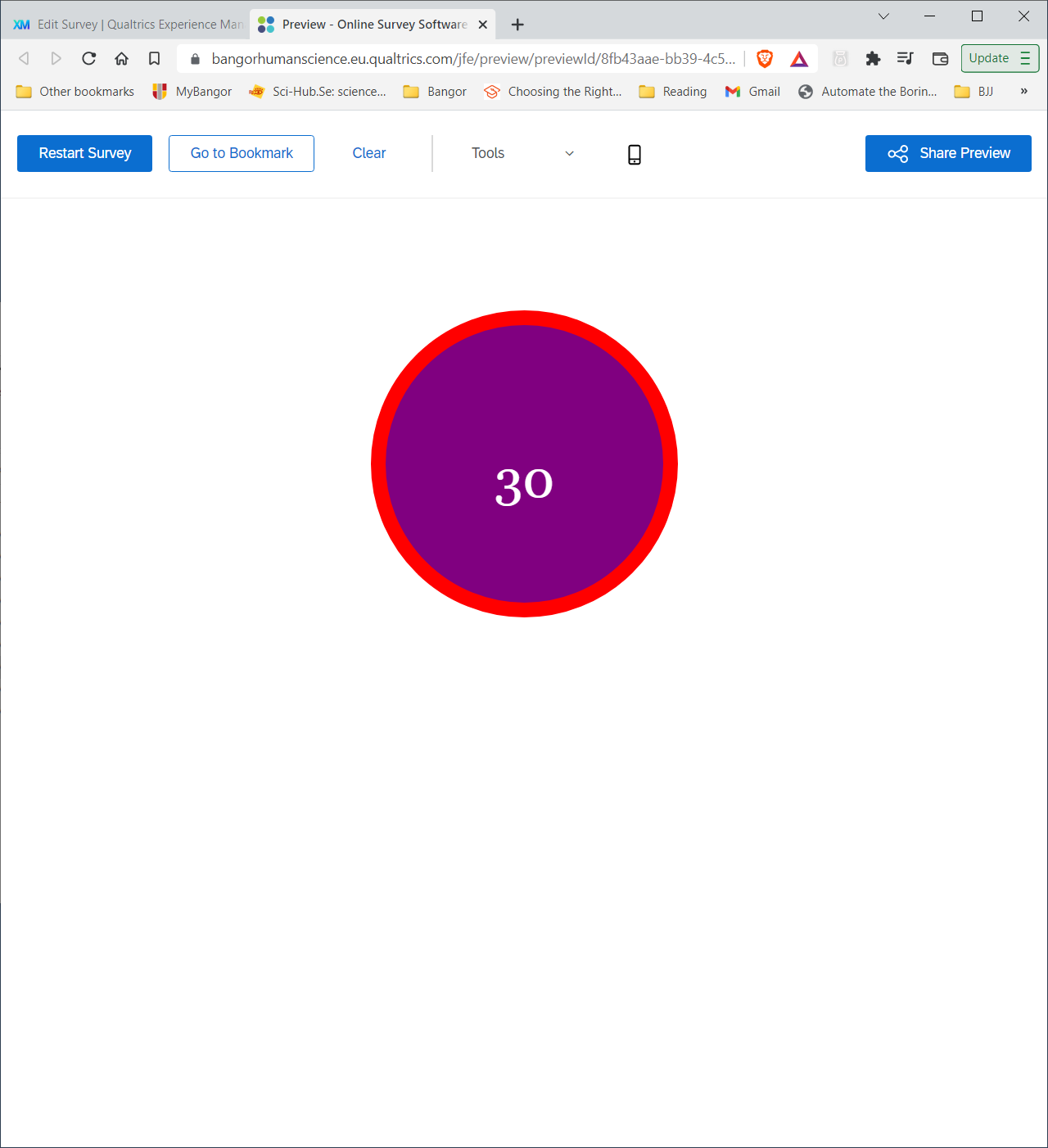


The resource would then replenish by around 15%.


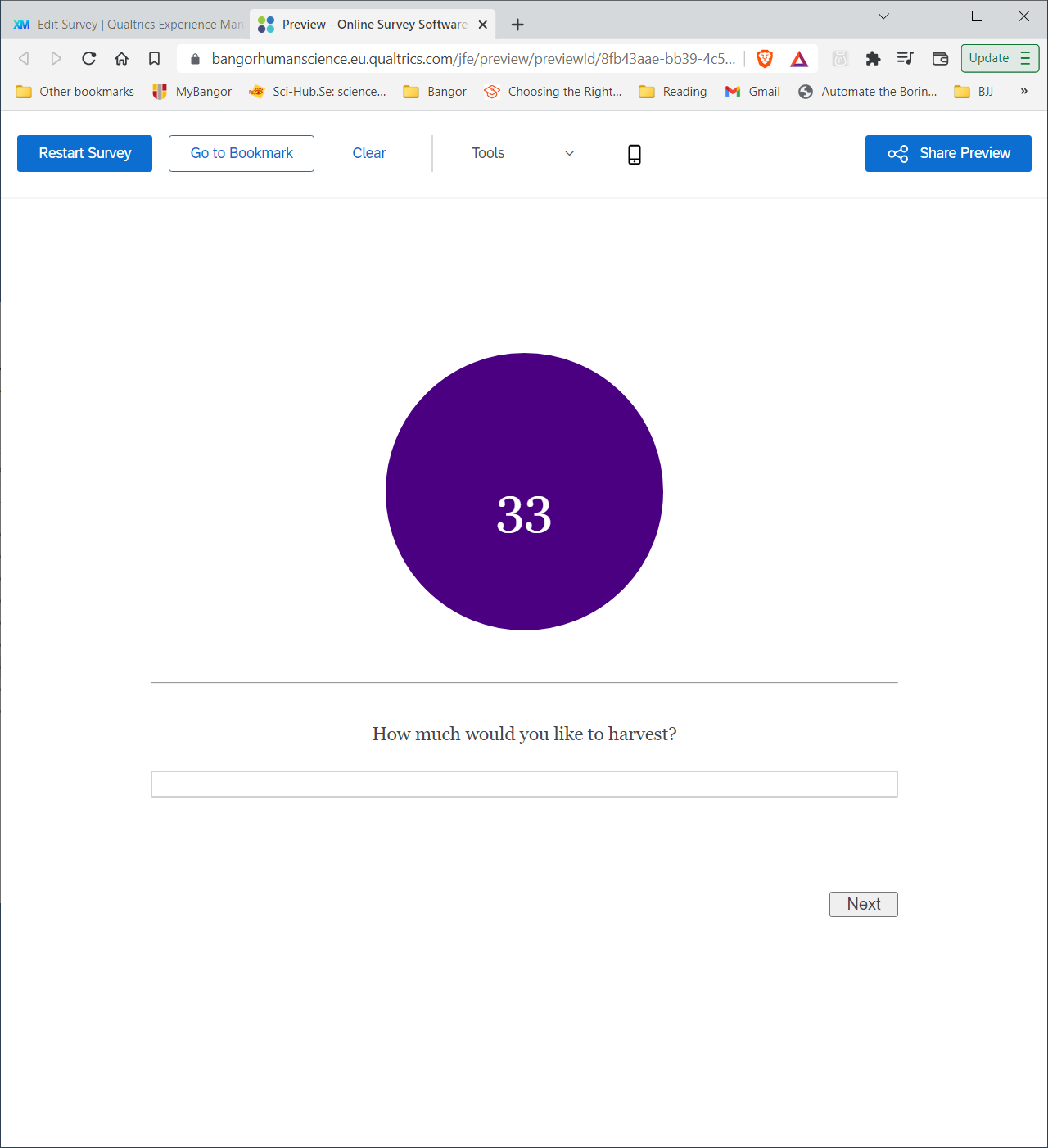


Perhaps, the participant might then choose to harvest the full amount of rewards contained in the pool.


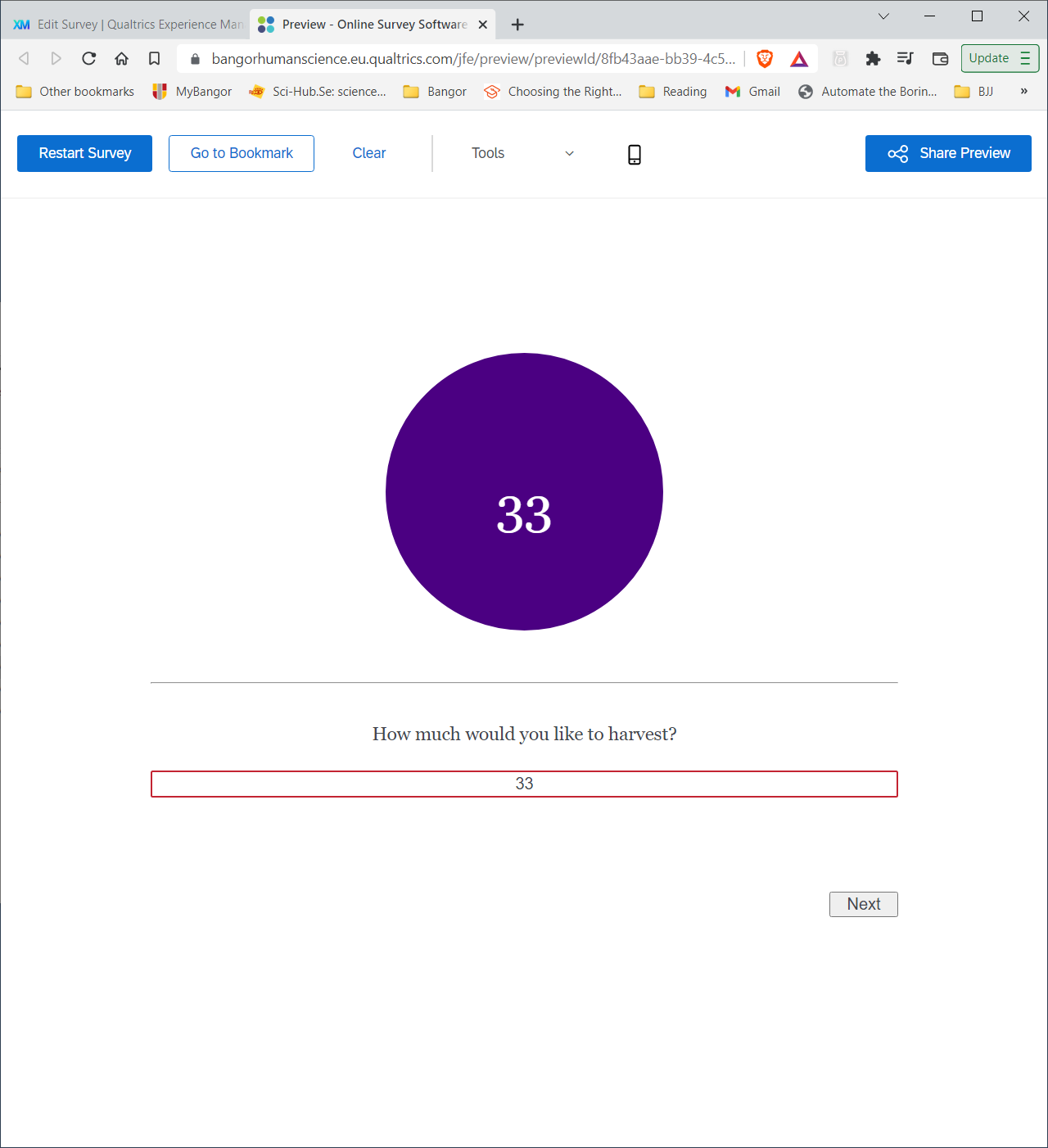


The resource would then be depleted to zero and would replenish no more.
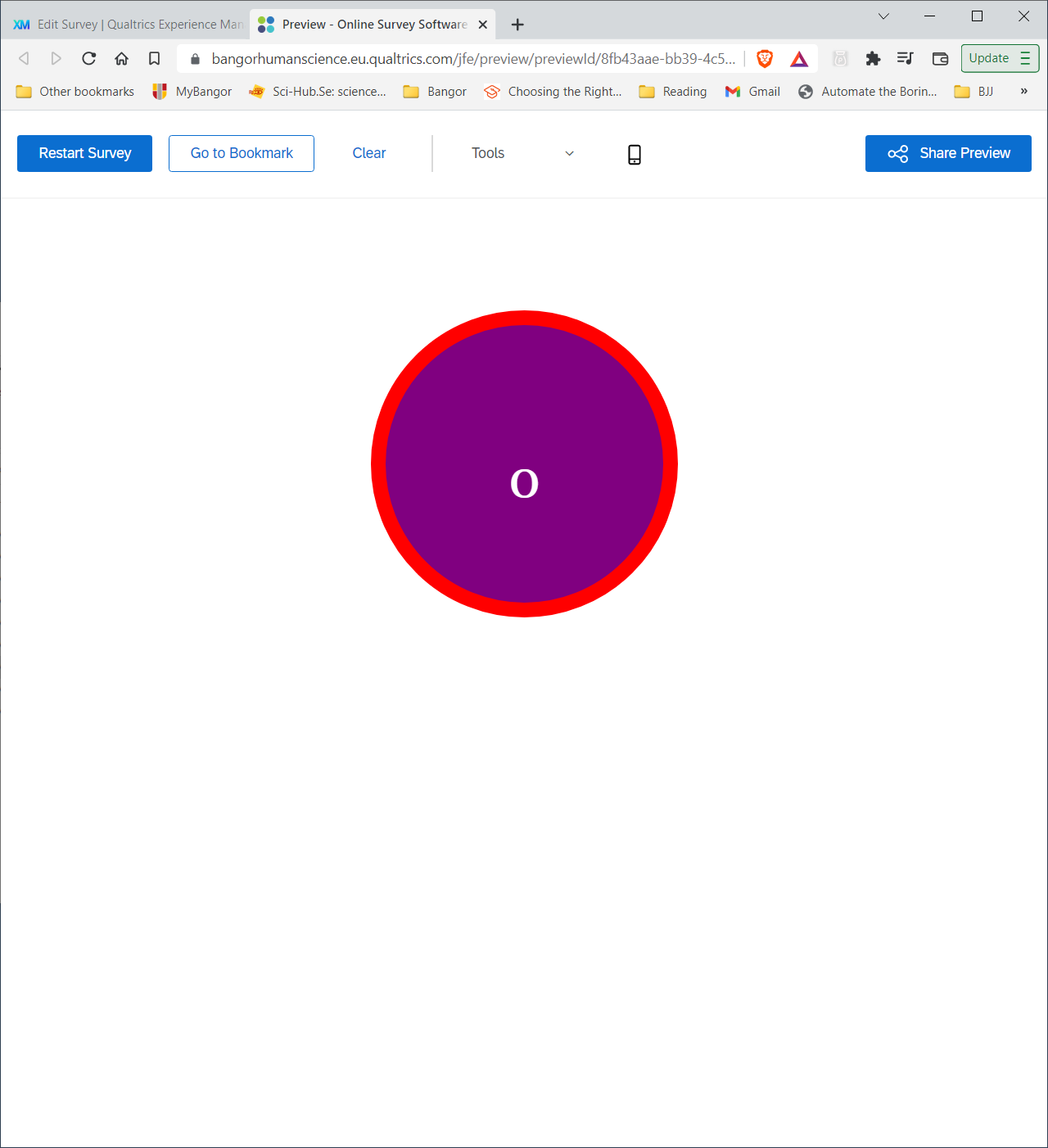


Once the game finished, participants saw this page.


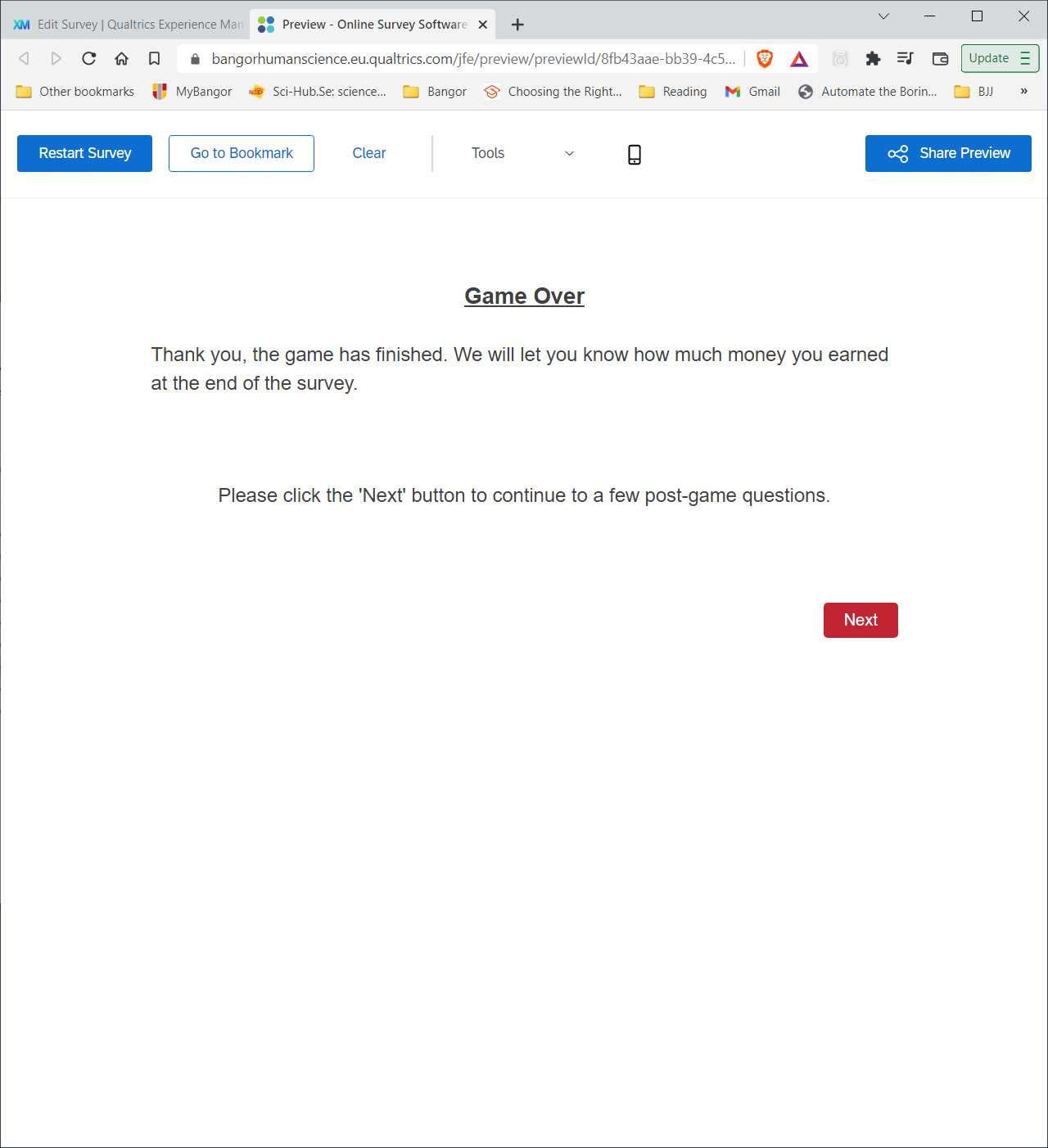


As the participant in our example finished the game before round 50, they would have to wait for a time penalty before they could continue.


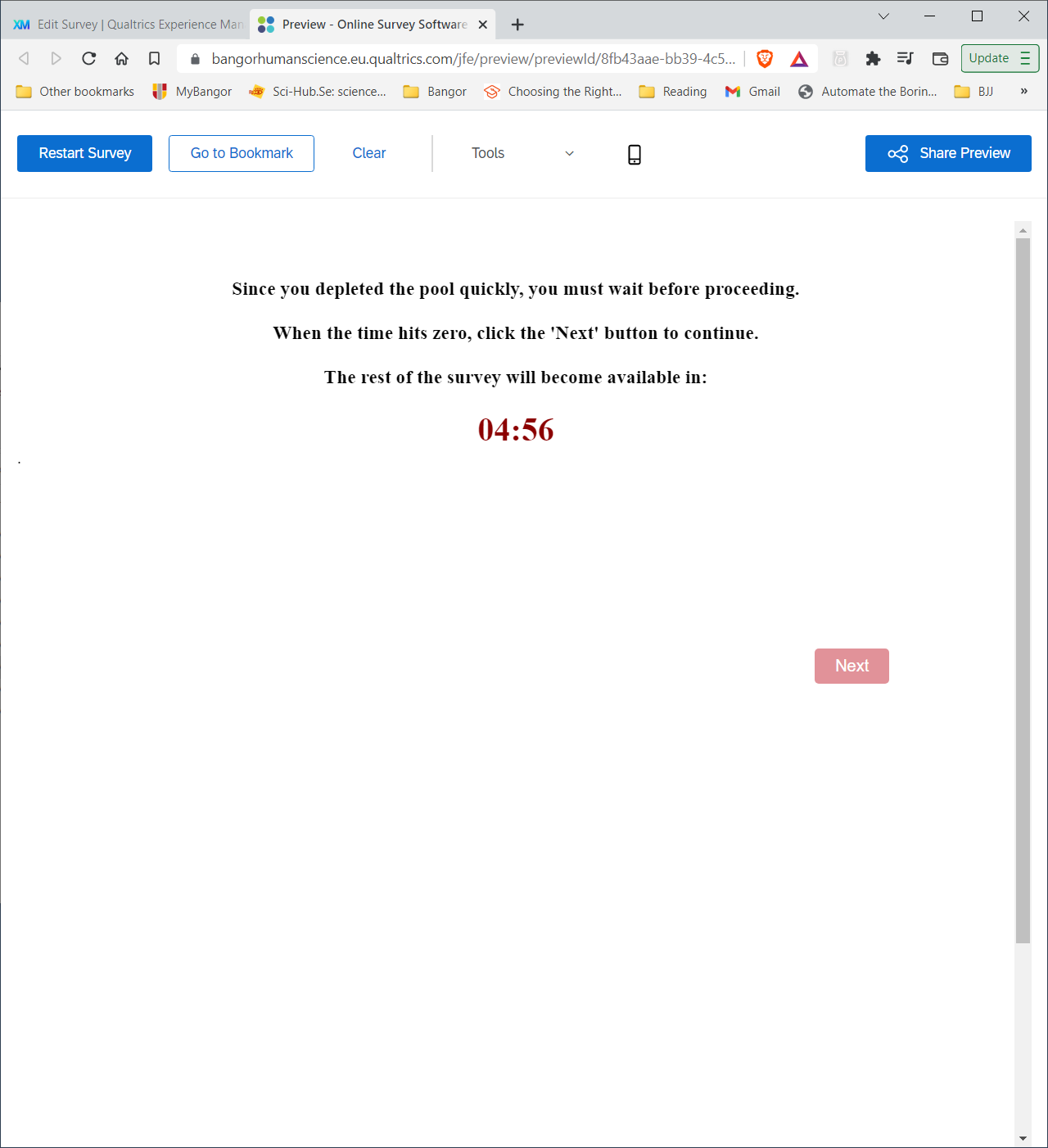


**Post-game reaction survey:**

Next, we asked participants some questions about how they felt during and after the game.
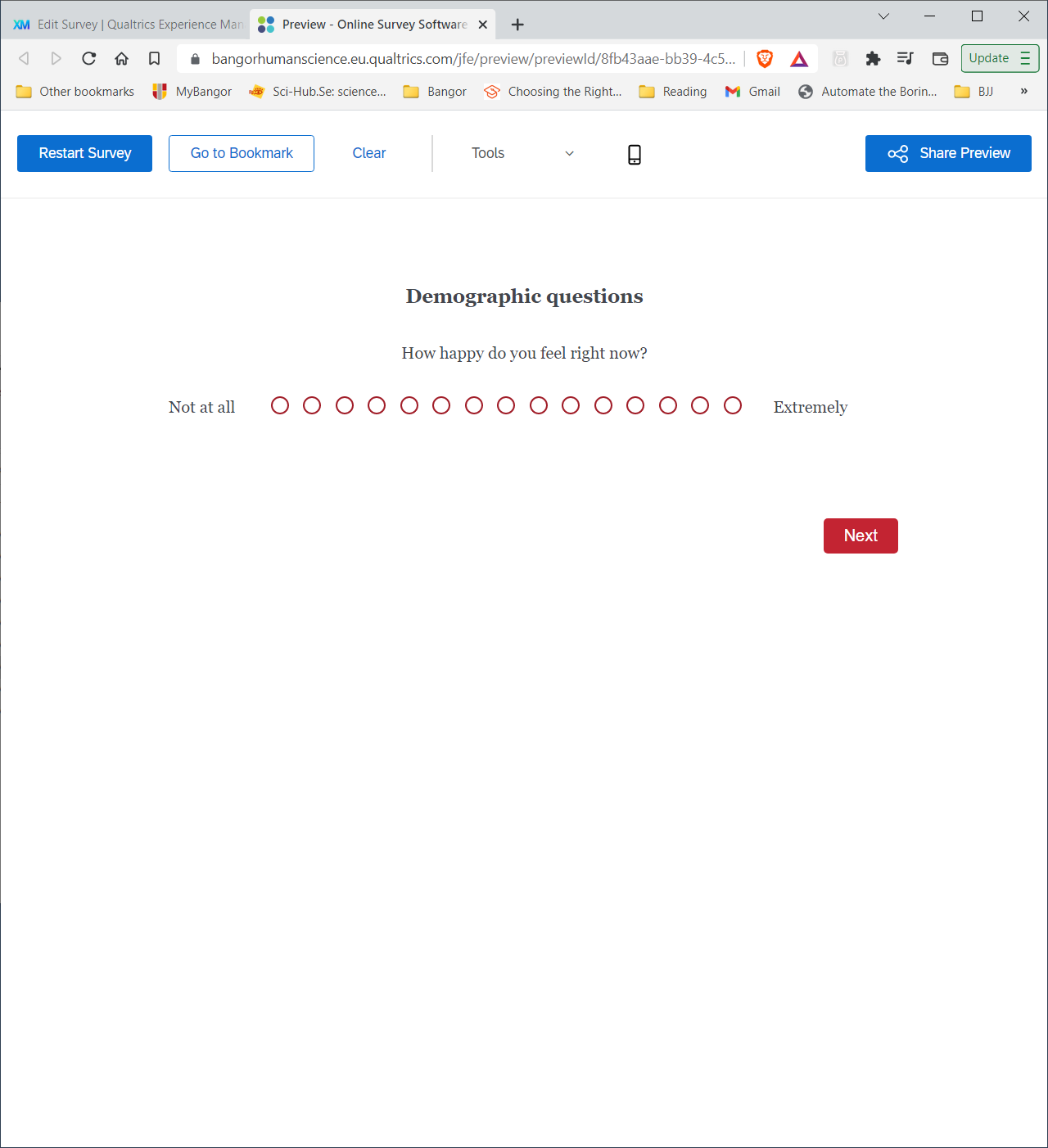


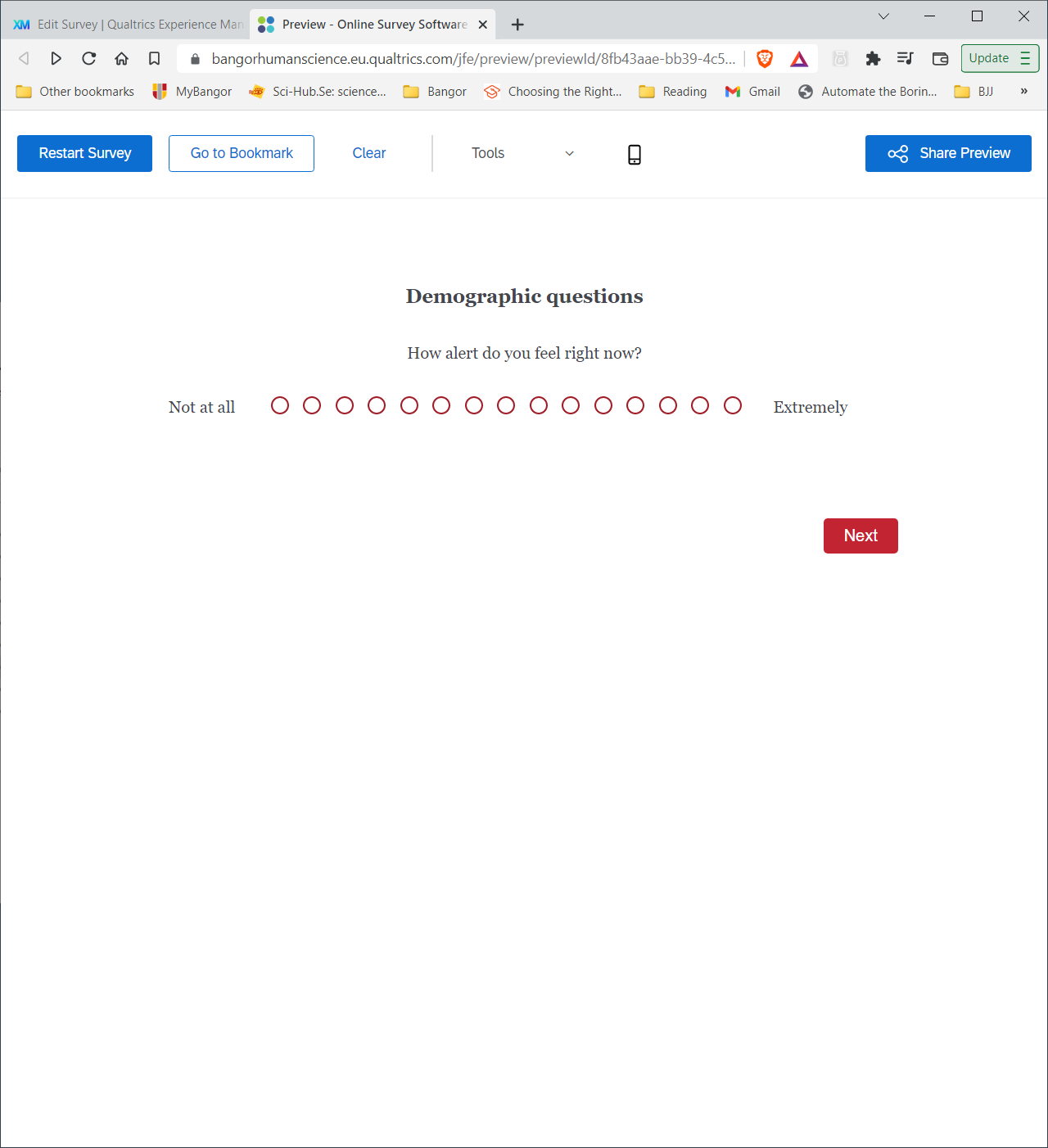


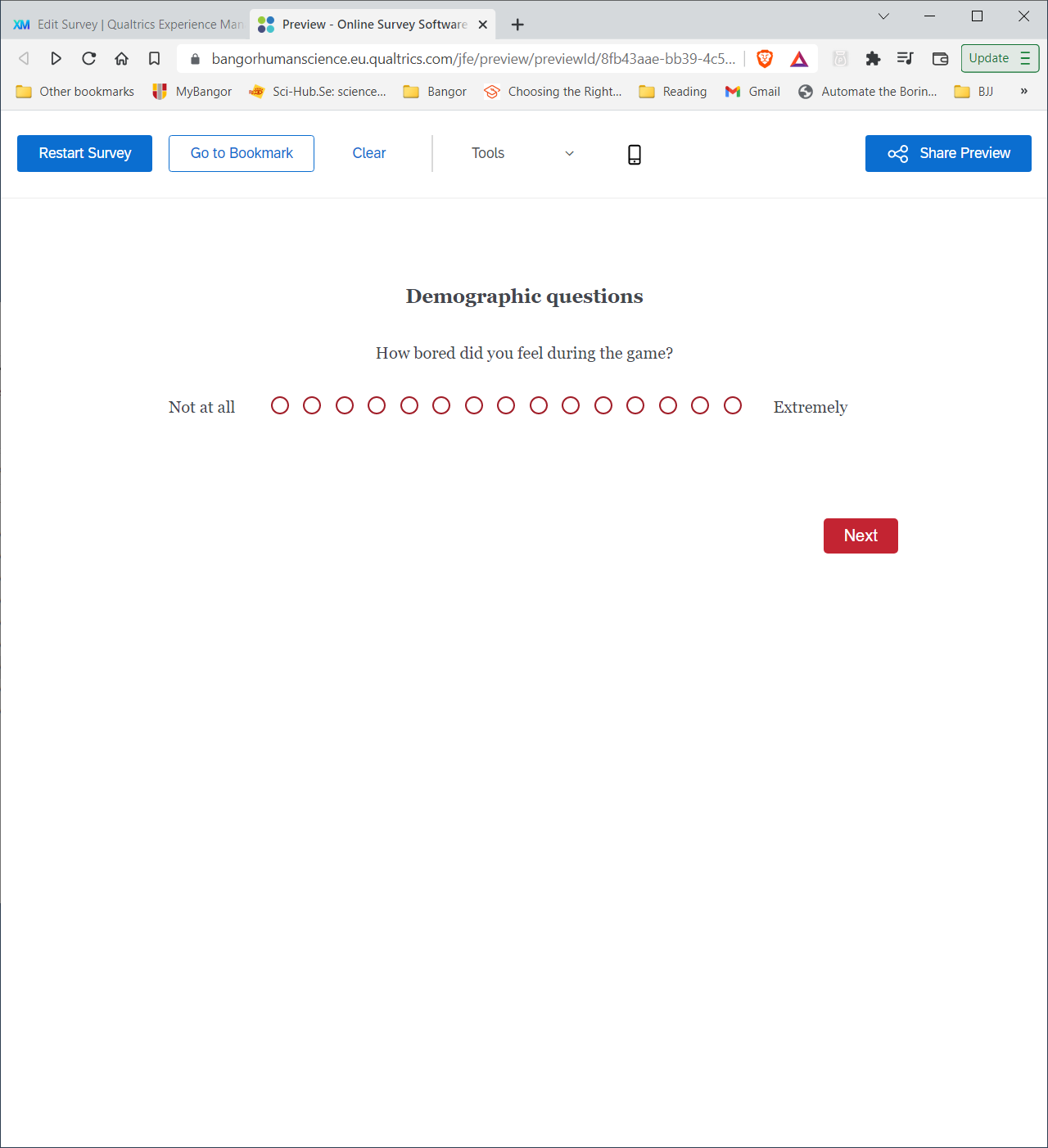


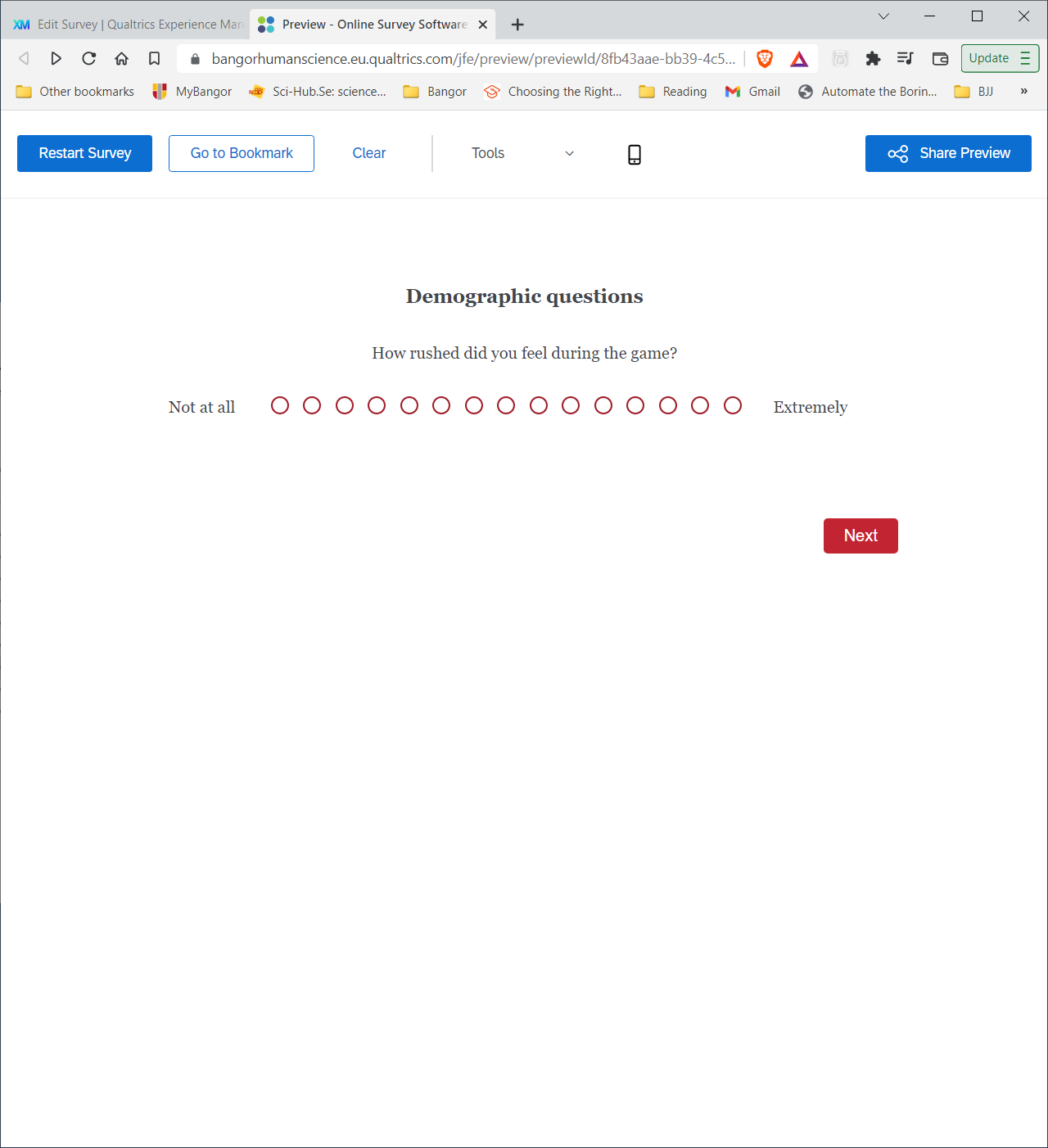


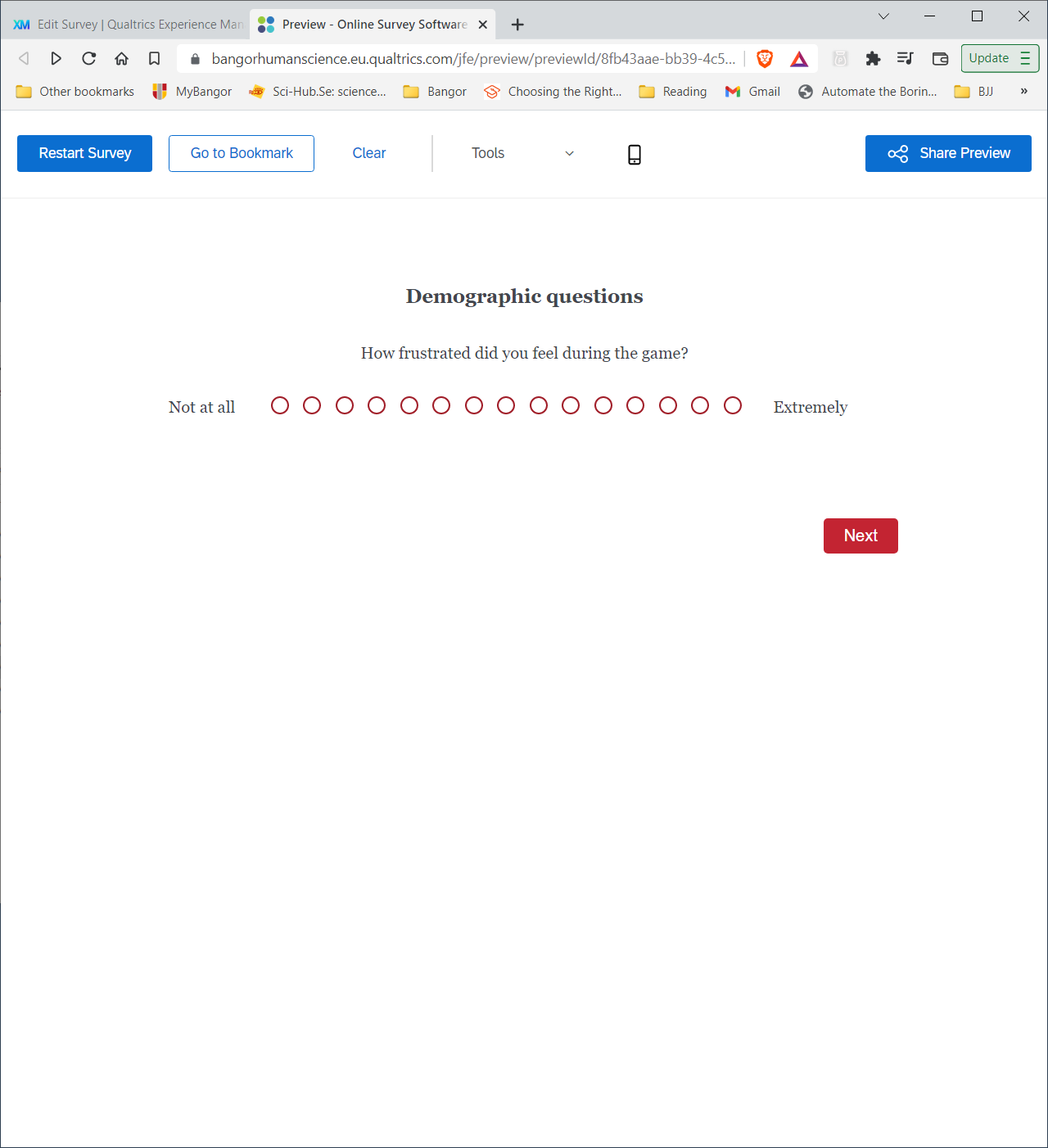


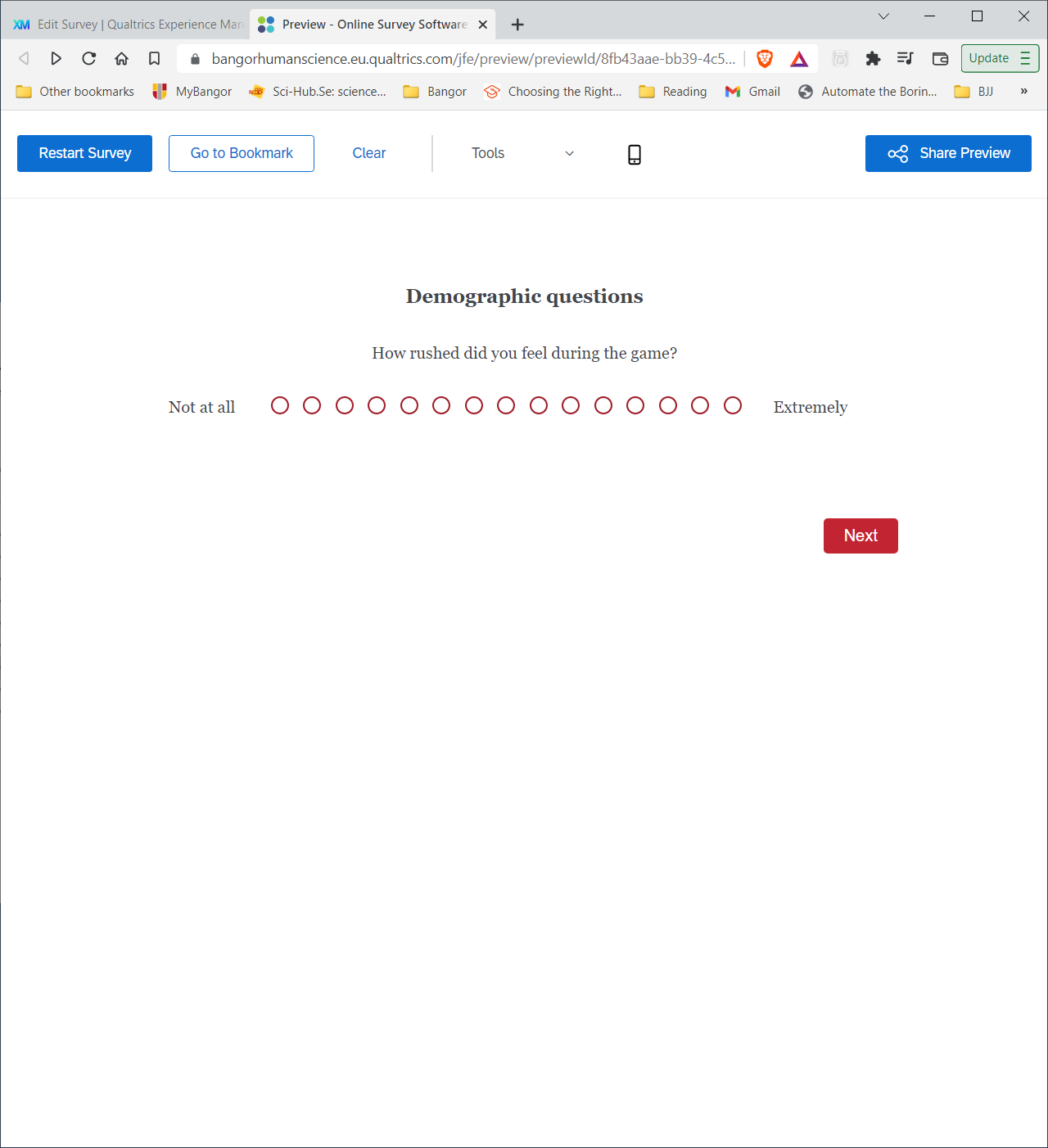


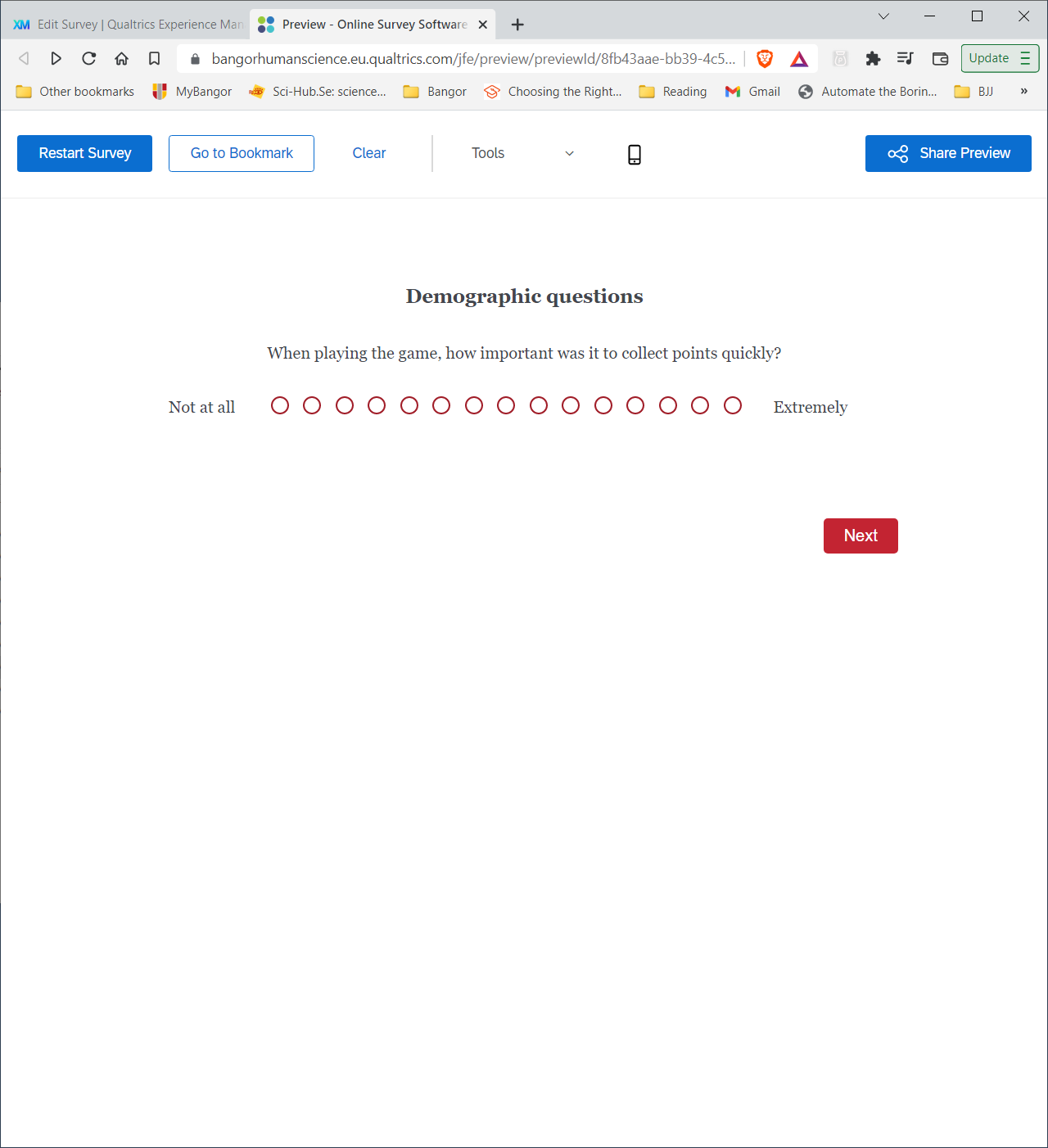


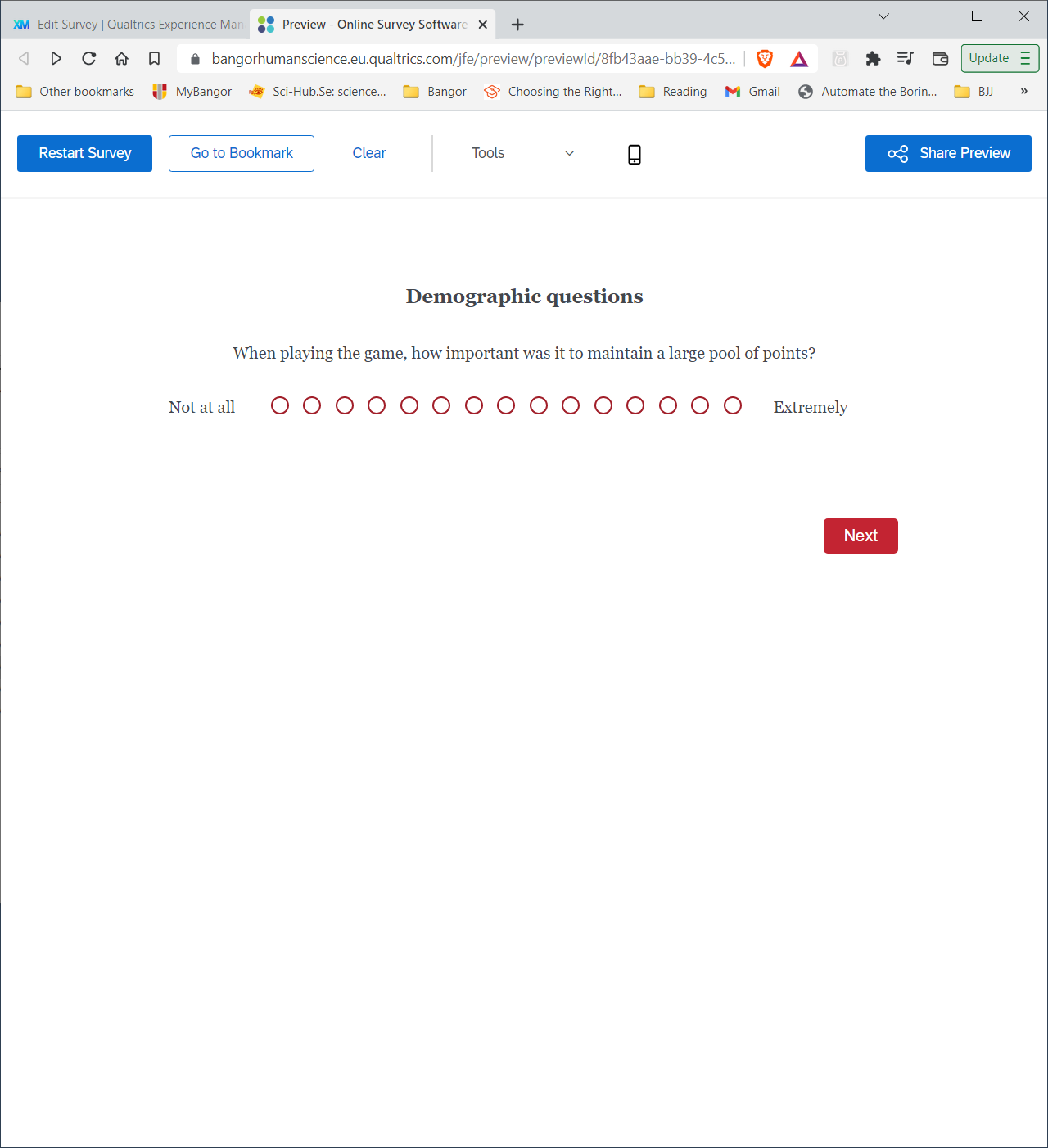


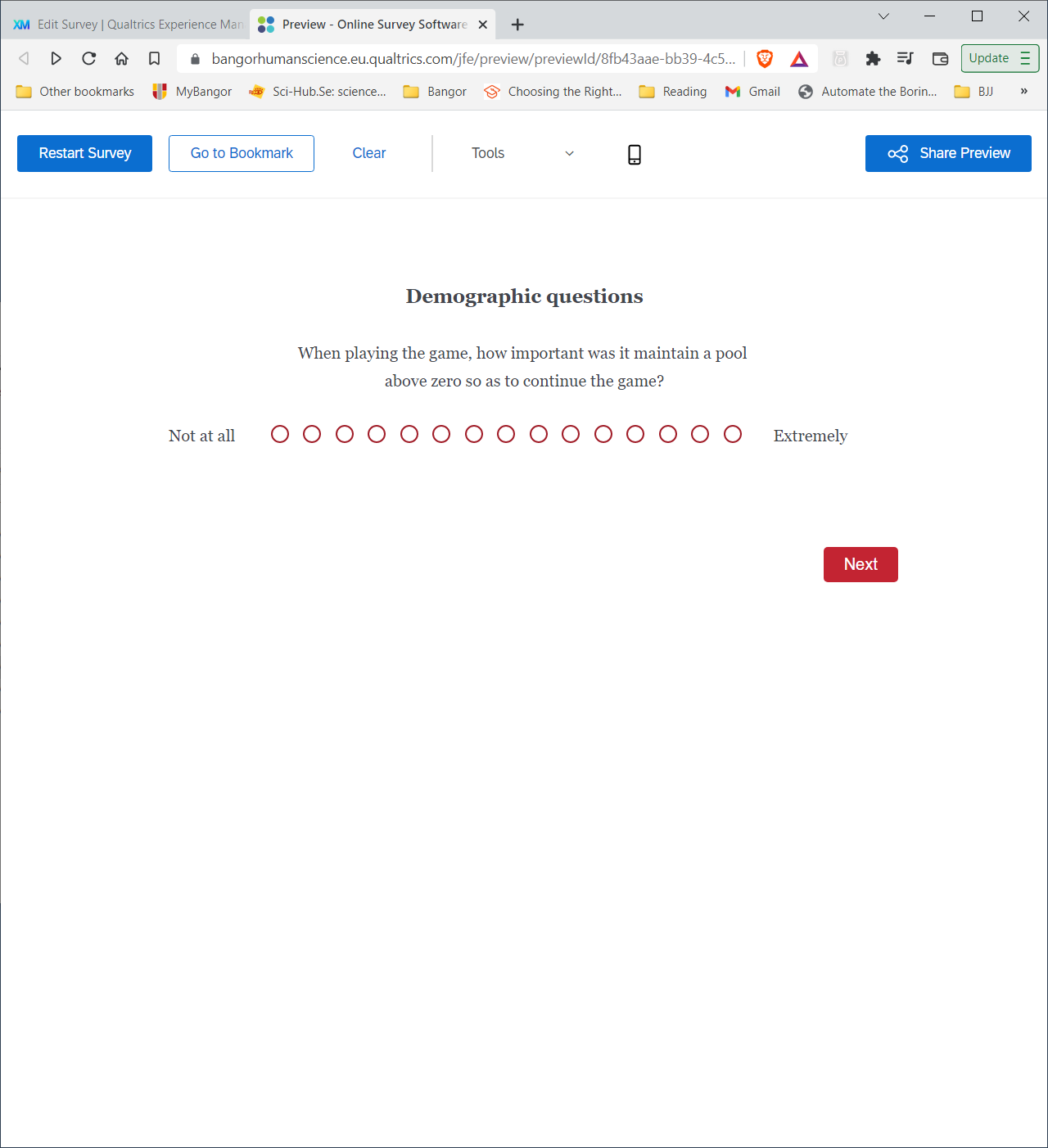


**Post-game understanding questions:**

We asked participants about their understanding of the resource replenished in the game they had just played.
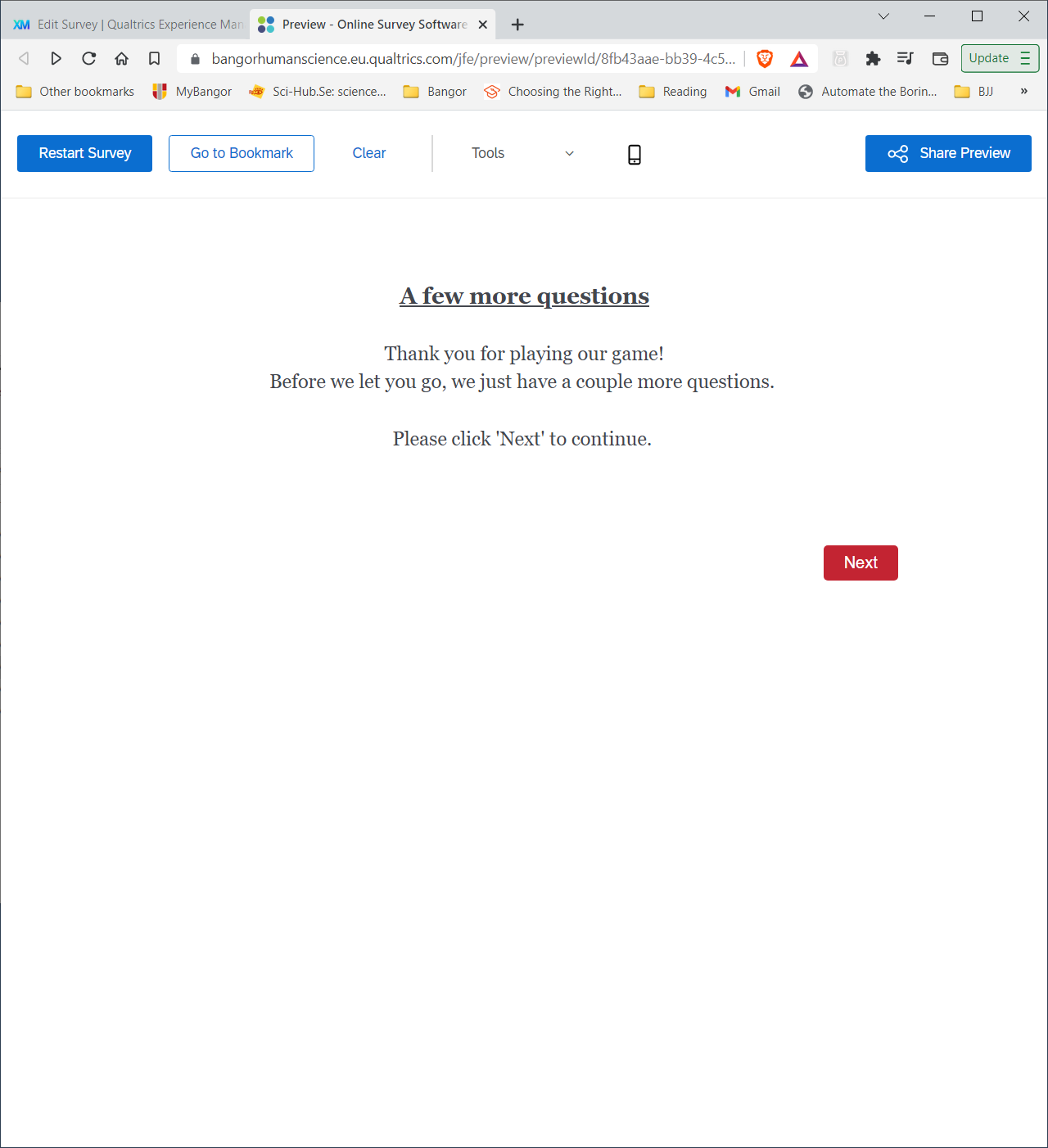


Depending on how a participant answered the question below, they may have been asked one additional question after this one.


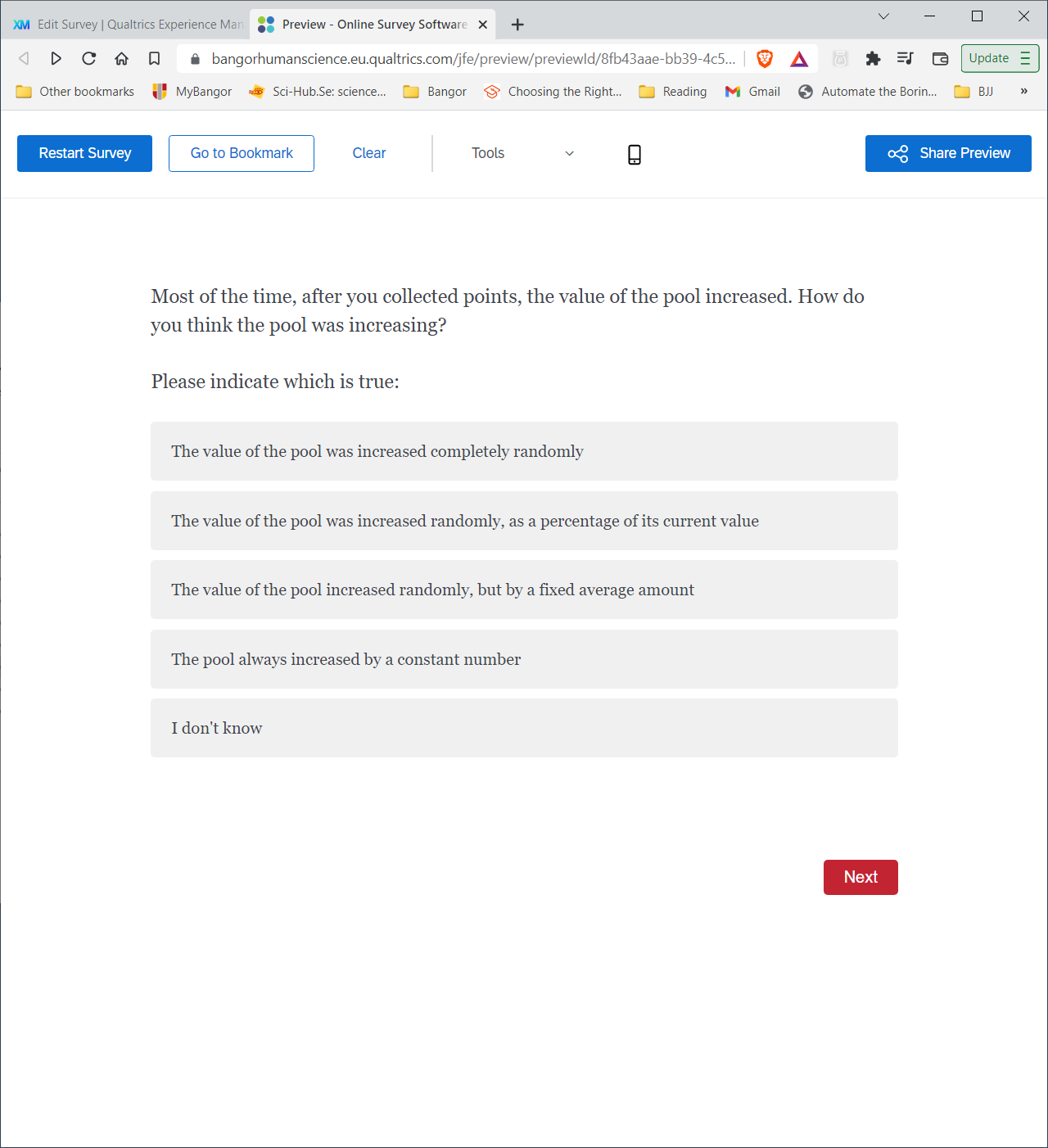


If a participant answered the first question *‘The value of the pool was increased randomly, as a percentage of its current value,’* they were then asked this question:
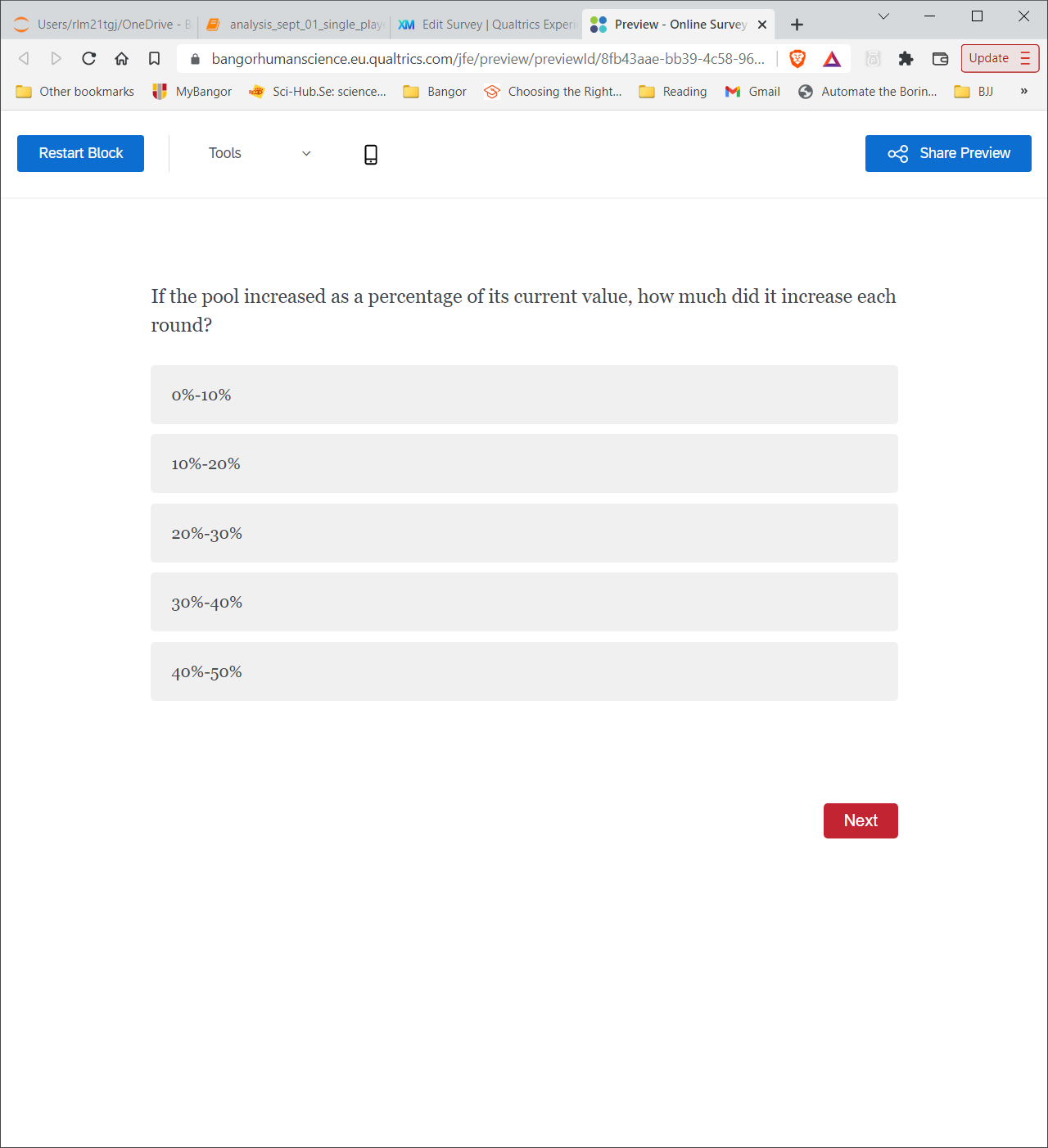


If a participant answered the first question *‘The value of the pool increased randomly, but by a fixed average amount,’* they were then asked this question:


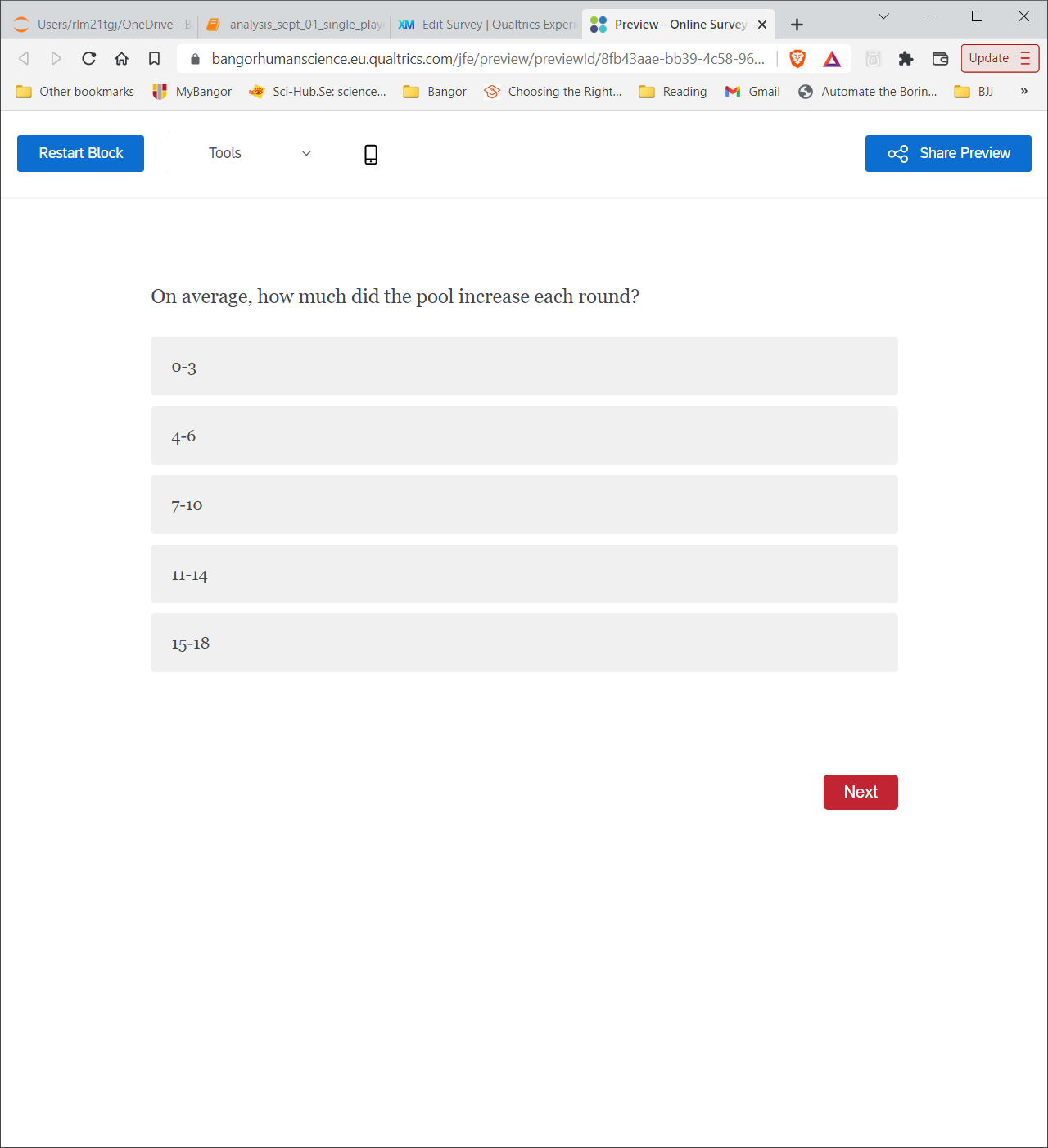


Otherwise, the participant would only be asked the first question and then move on to the next part of the survey.

**Individual characteristic measures:**

We then asked participants to complete some questionnaires to measure their individual characteristics. Data for this experiment was collected in two waves. In the first wave, 200 participants completed the (i) ED_50_ (Cox & Dallery, 2016), (ii) GHQ-12 (Goldberg et al., 1997), (iii) AUDIT (Reinert & Allen, 2002); and (iv) WHO-5 (Topp et al., 2015). In the second wave, 200 participants completed the elicitations for: (i) ED50 and (ii) financial literacy (OECD; Čonková, 2014).

Therefore, the procedure for the two waves differed a little for this part of the experiment. However, both waves started off this part of the experiment by completing the ED_50_, a measure of delay discounting.

**ED_50_ task:**

In the ED_50_ task (Koffarnus & Bickel, 2014), participants are presented with five consecutive choices, where they indicate whether they would prefer $500 now or $1000 after some delay. If the participant chooses the immediate sum of $500 in one round, the delay for the $1000 sum is decreased for the next choice. Likewise, if the participant chooses the delayed $1000 sum in one round, the delay is increased for the following round. By the fifth round, through adjusting the delay for the later reward according to a participant’s choices, we find the point at which the participant subjectively devalues the later sum by 50%. The ED_50_ score is determined by the participant’s choice in the fifth round of the task. Of note, for each question, we randomized the order of the $500 and $1000 choice.

Below, we give an example play-through of the first two choices a participant might make in the task.


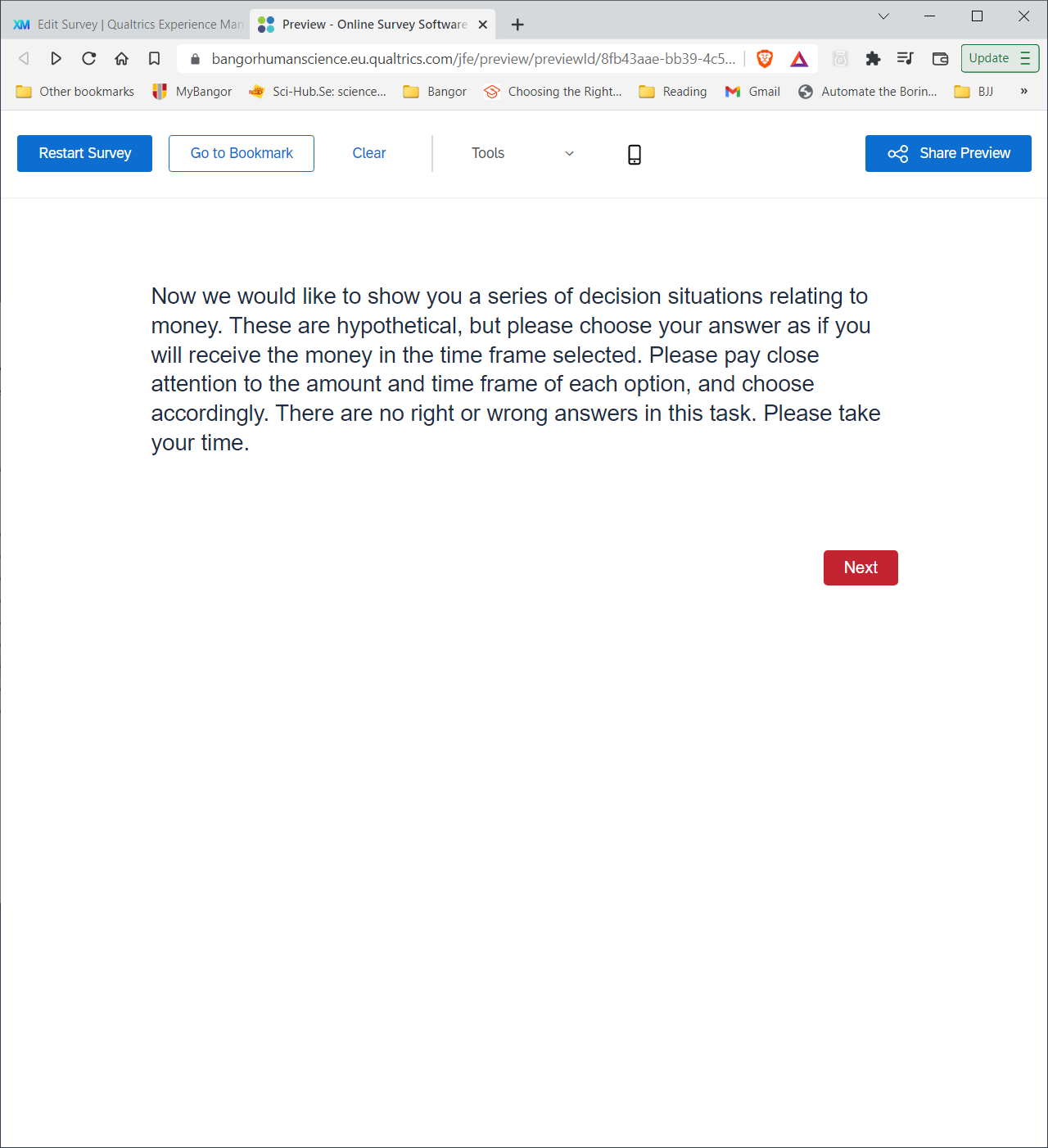


All participants started the task with the following hypothetical decision:


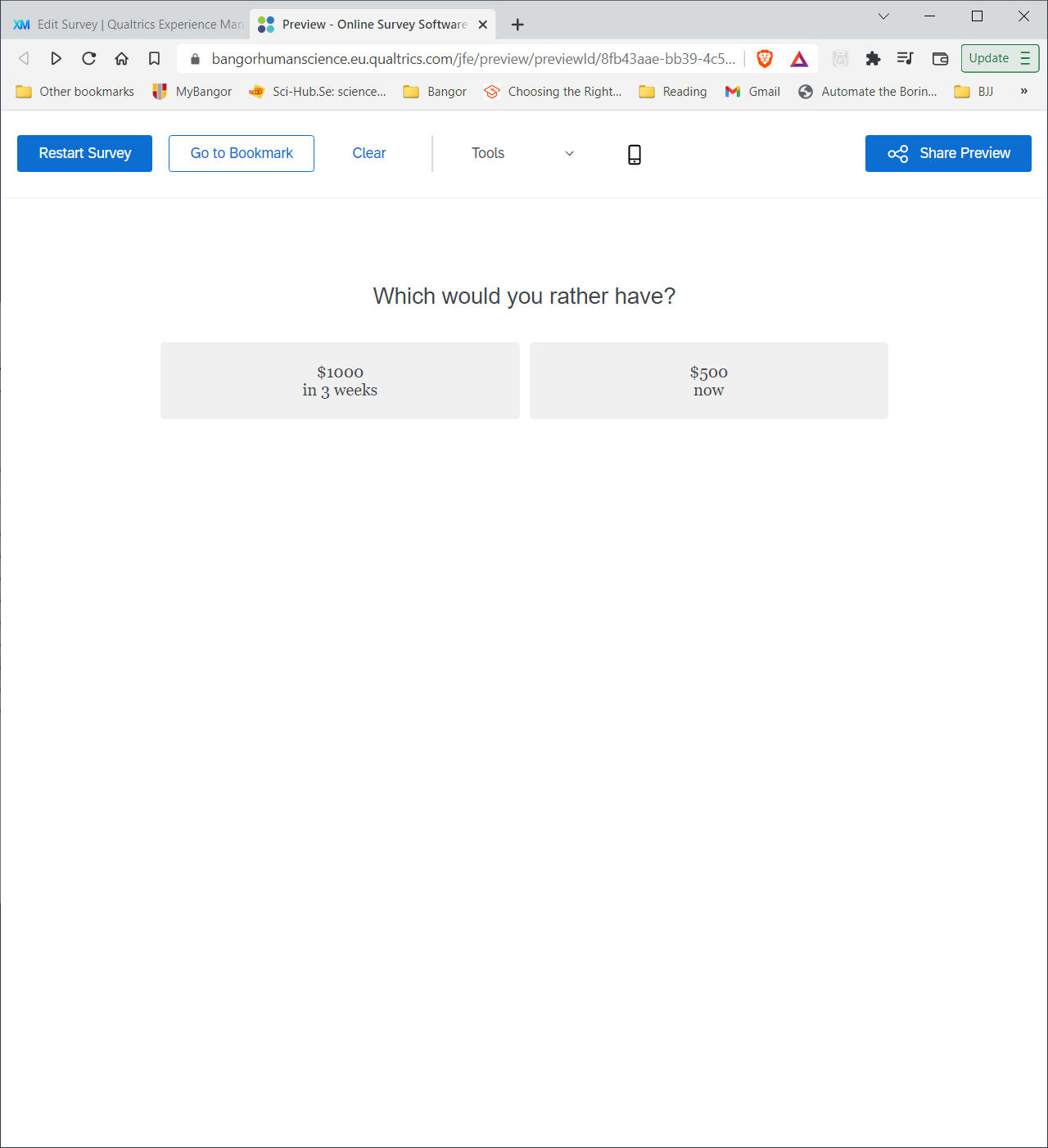


A participant may, for example, prefer the delayed sum here.


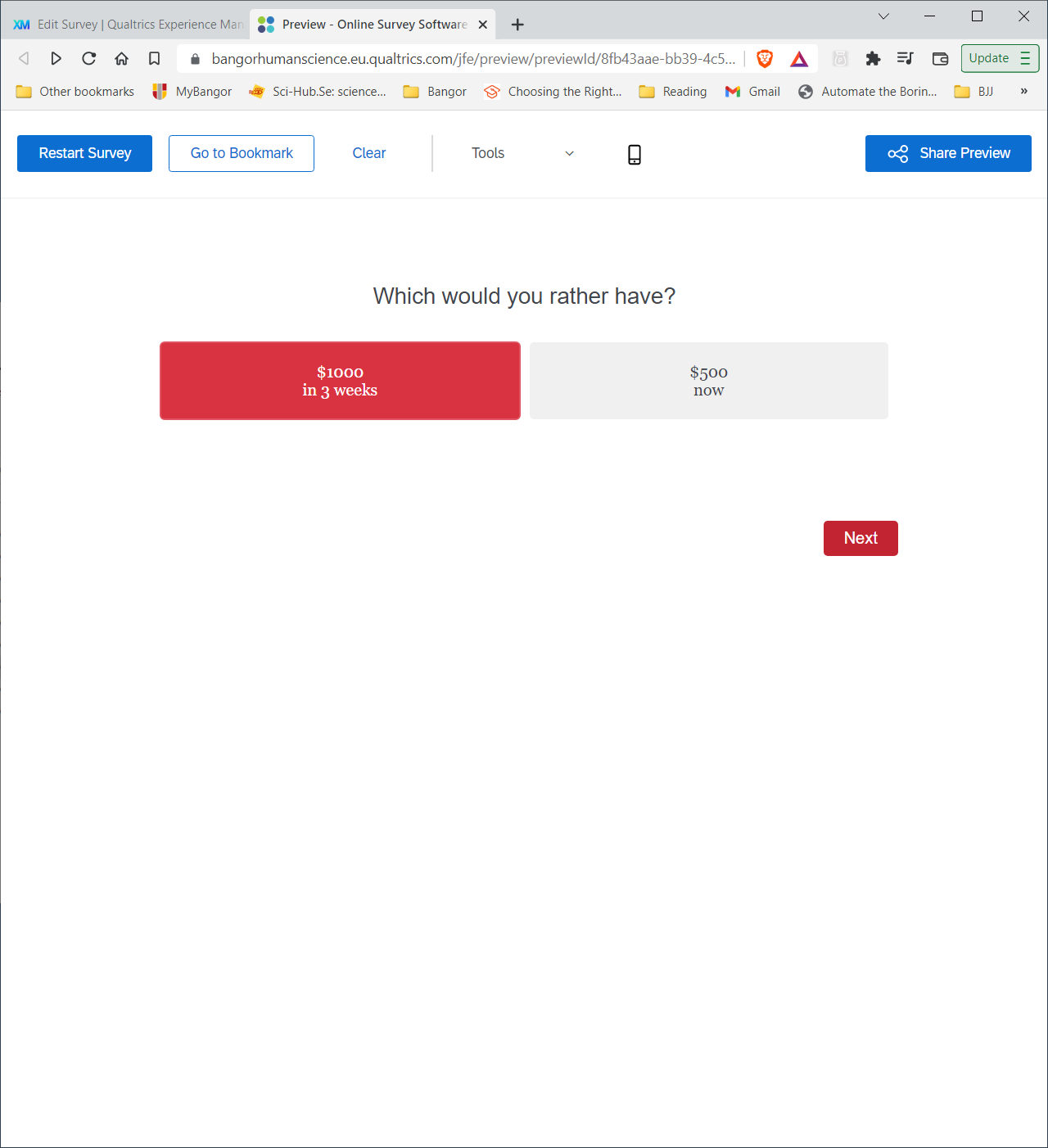


The delay for the $1000 sum is then increased for the next decision.


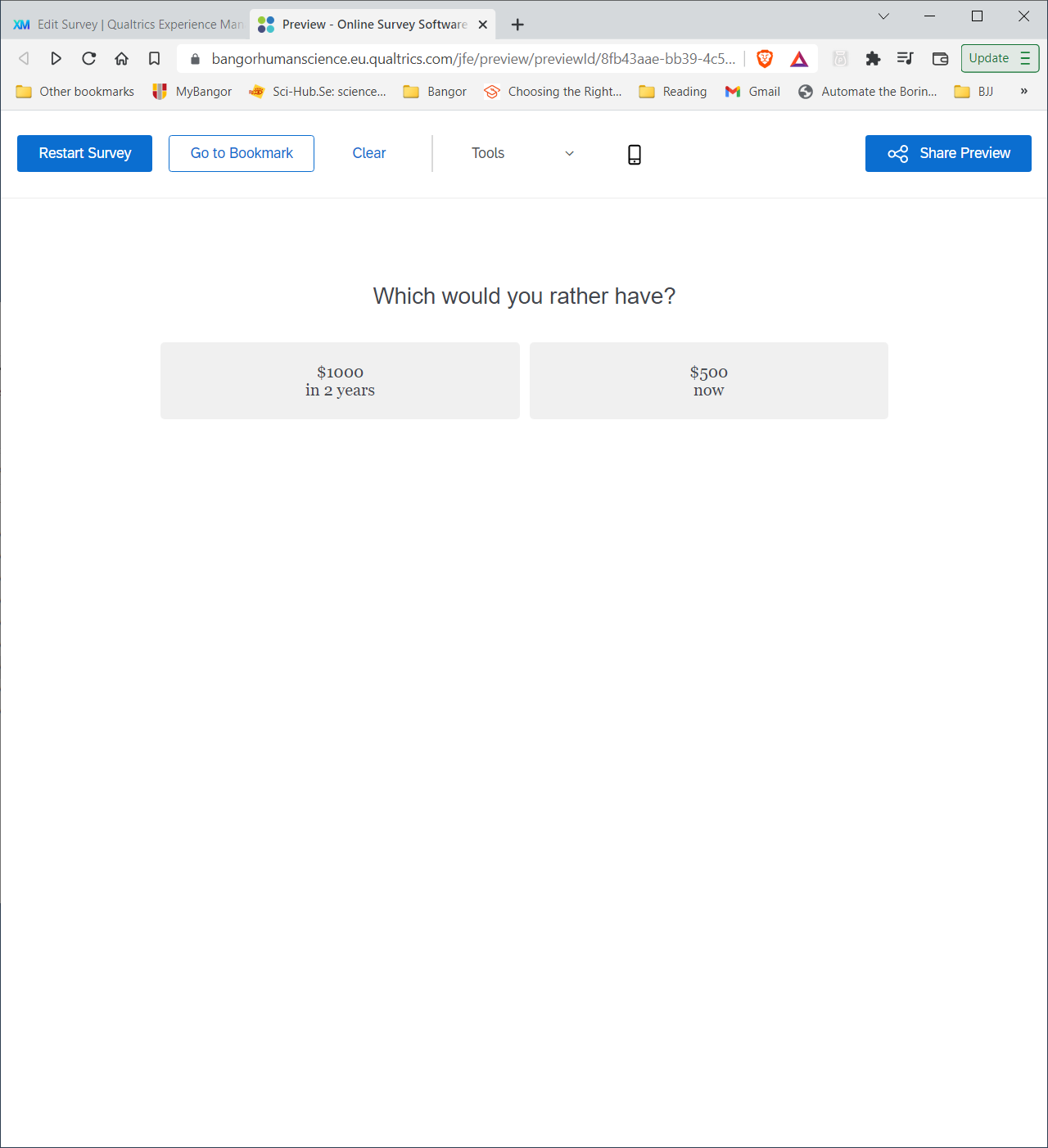


The participant may then prefer the immediate sum in this decision.


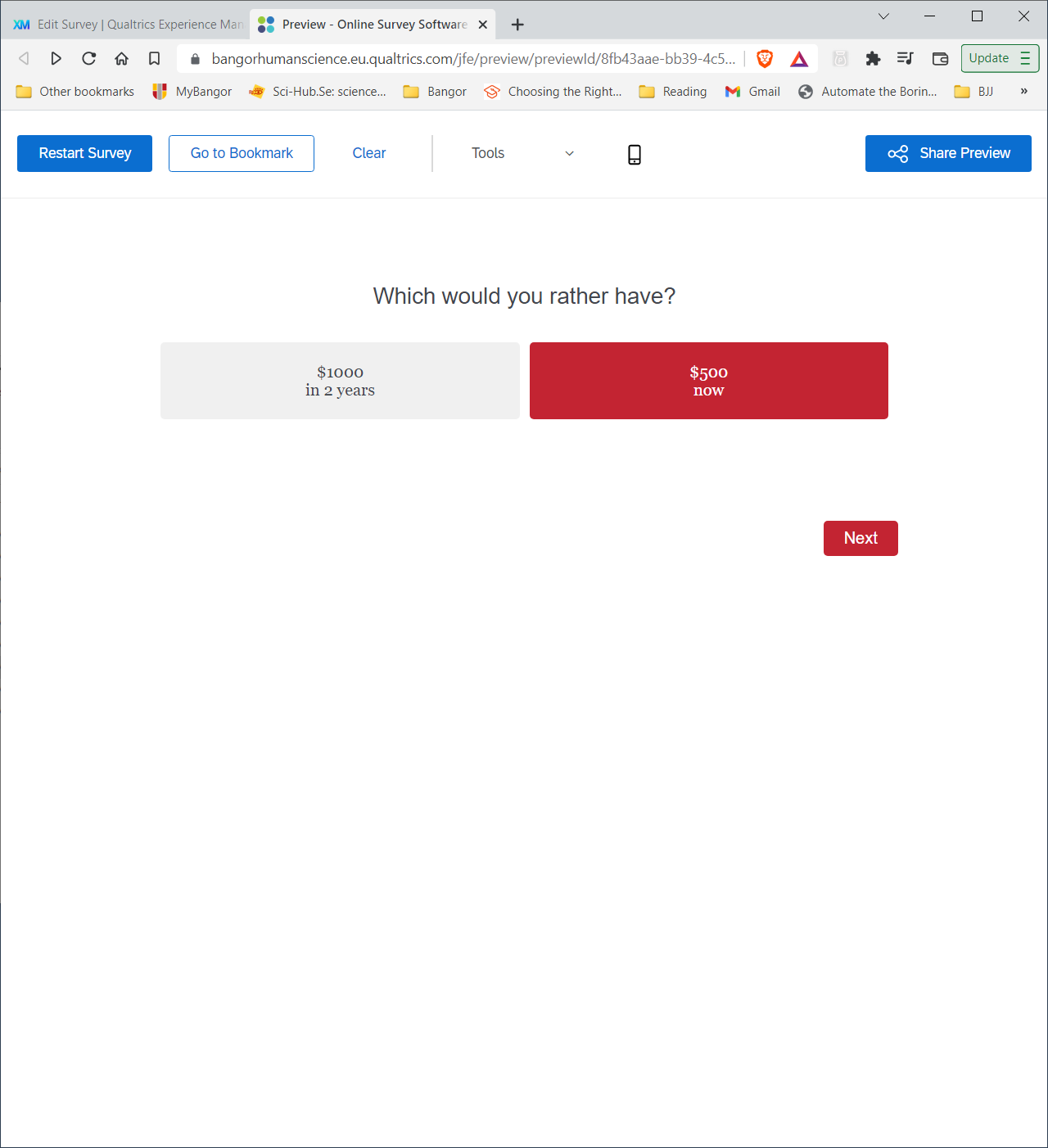


The delay for the $1000 sum is then reduced for the next round.


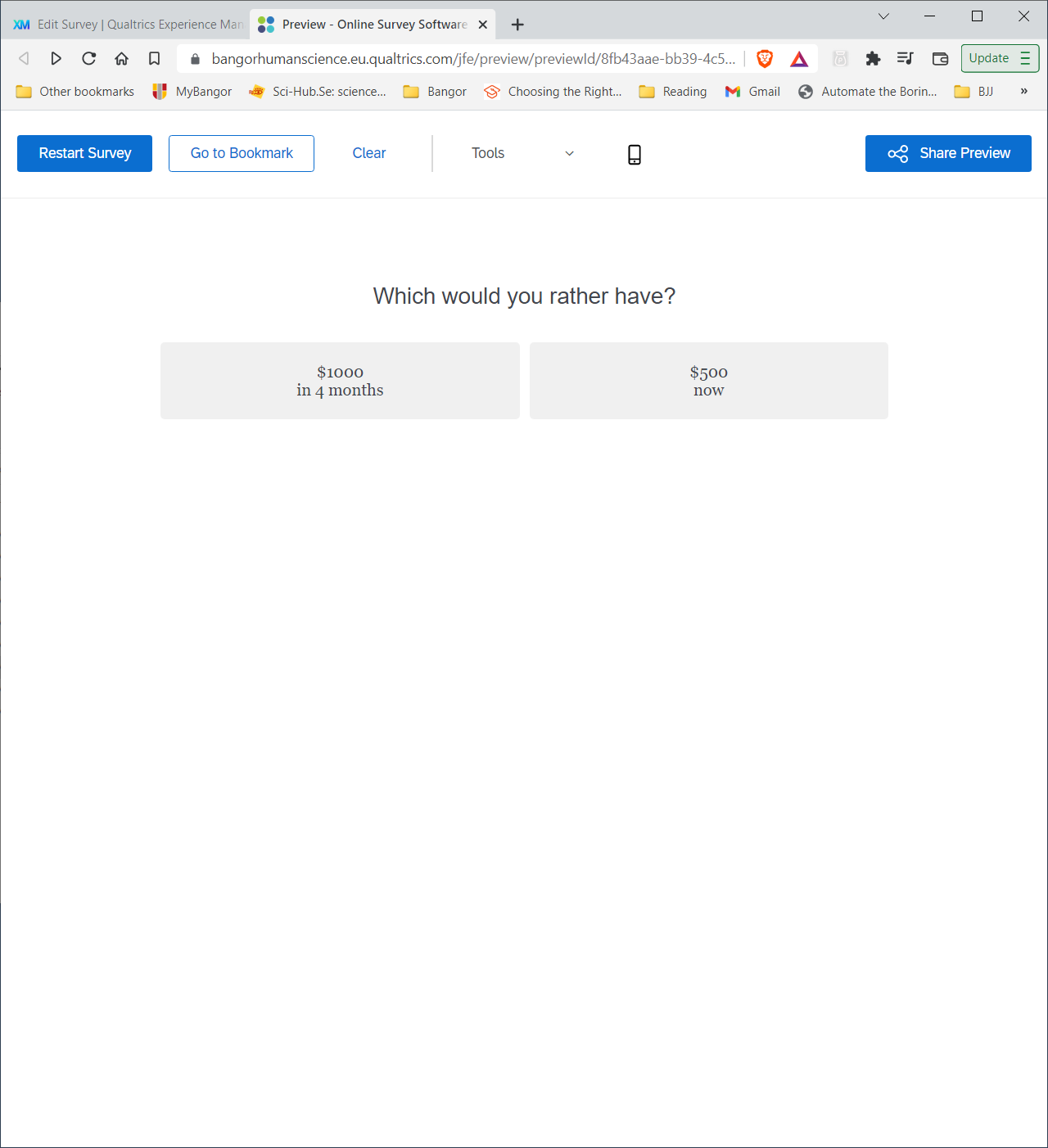


At this point, the two versions of the survey (used in the two waves of data collection) diverge. The following (up to the ‘Game Over’ end of survey message) is the procedure used for the **first** **wave** of data collection.

**Wave 1: General Health Questionnaire – short form version (GHQ-12)**

Here, participants completed the GHQ-12 (Goldberg et al., 1997), a short form, twelve-item version of the The General Health Questionnaire (GHQ; (Goldberg & Blackwell, 1970) developed to help identify psychological distress. Each item is scored from 0 (“better than usual”) to 3 (“much less than usual”).


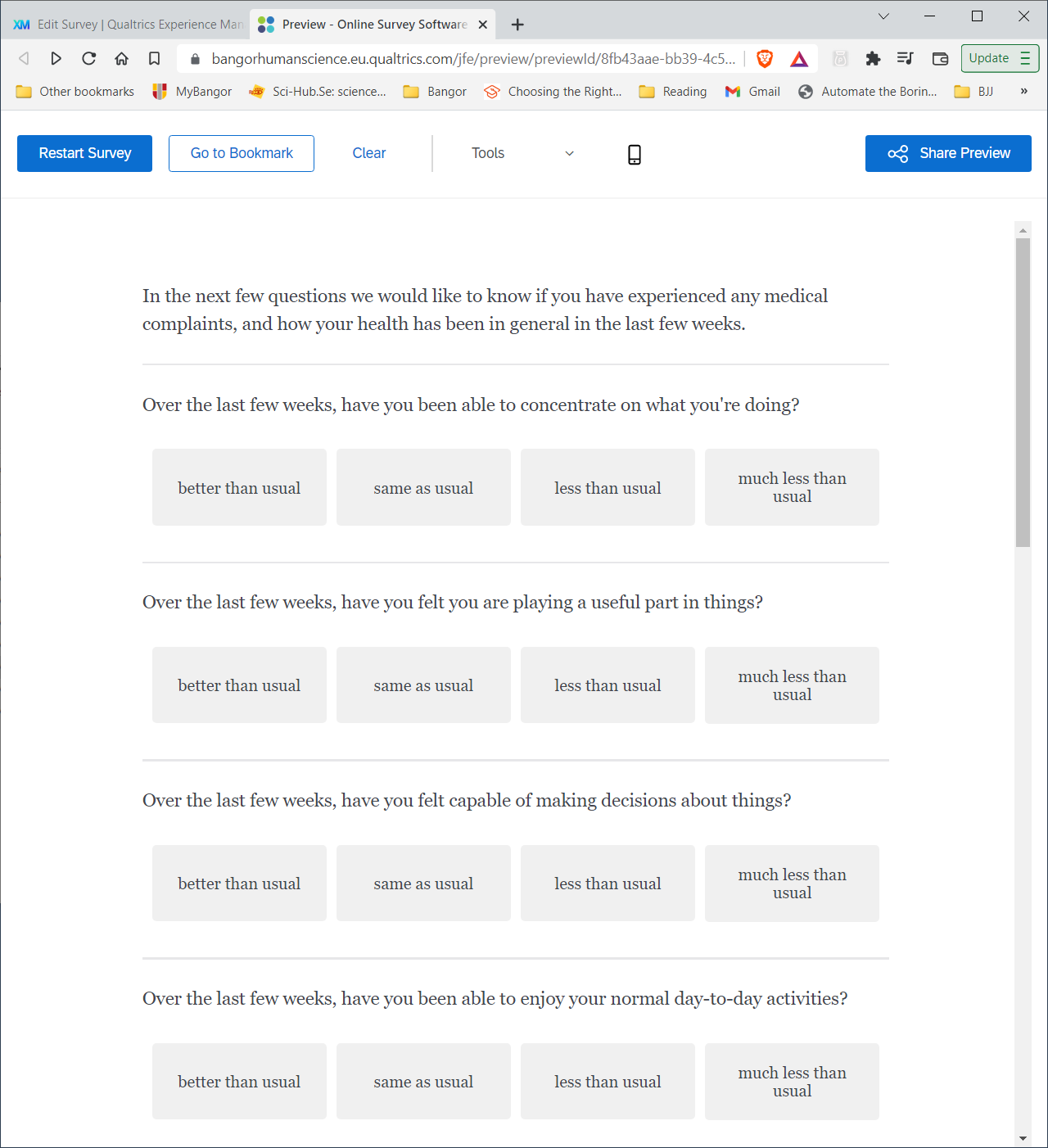


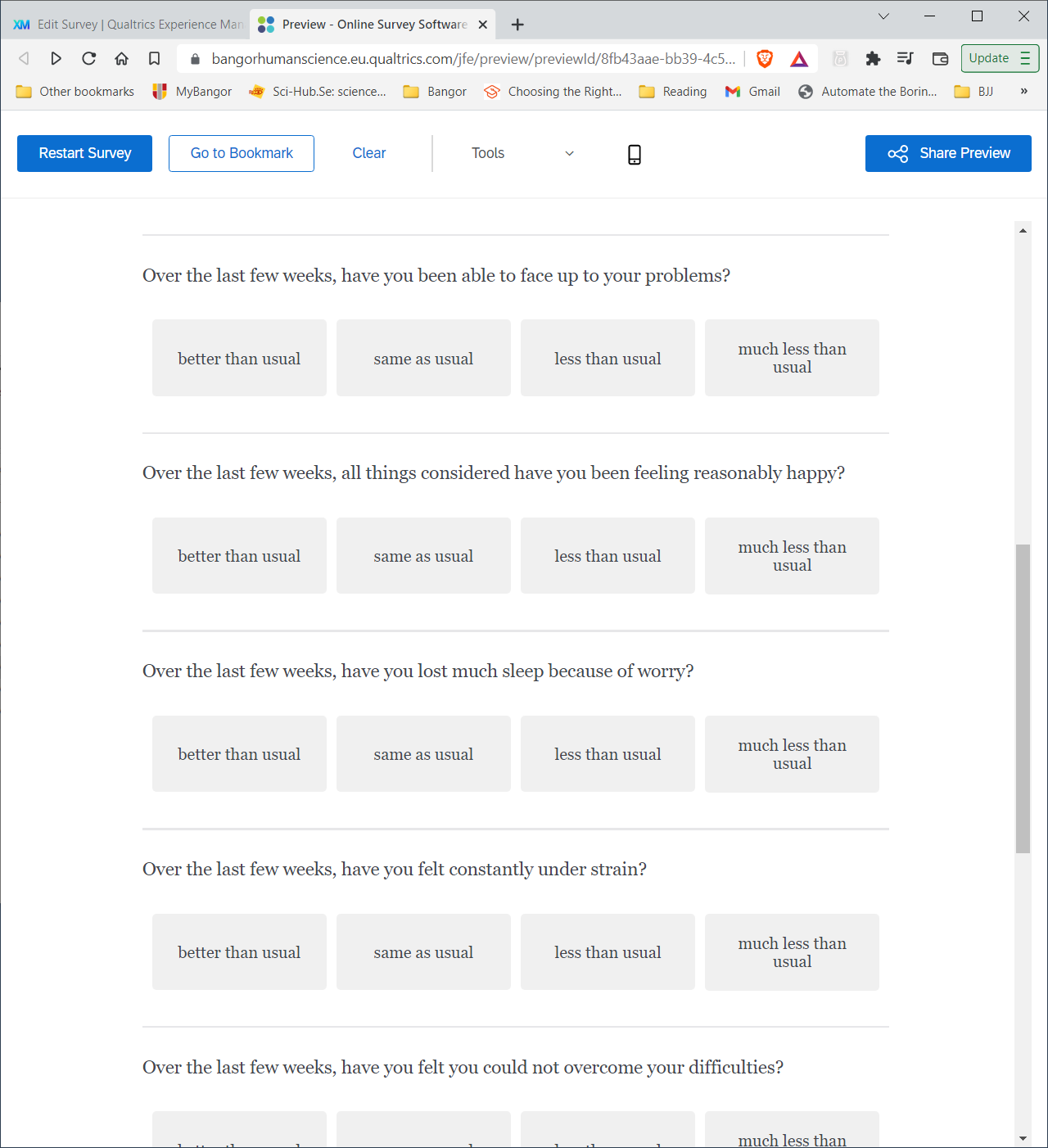

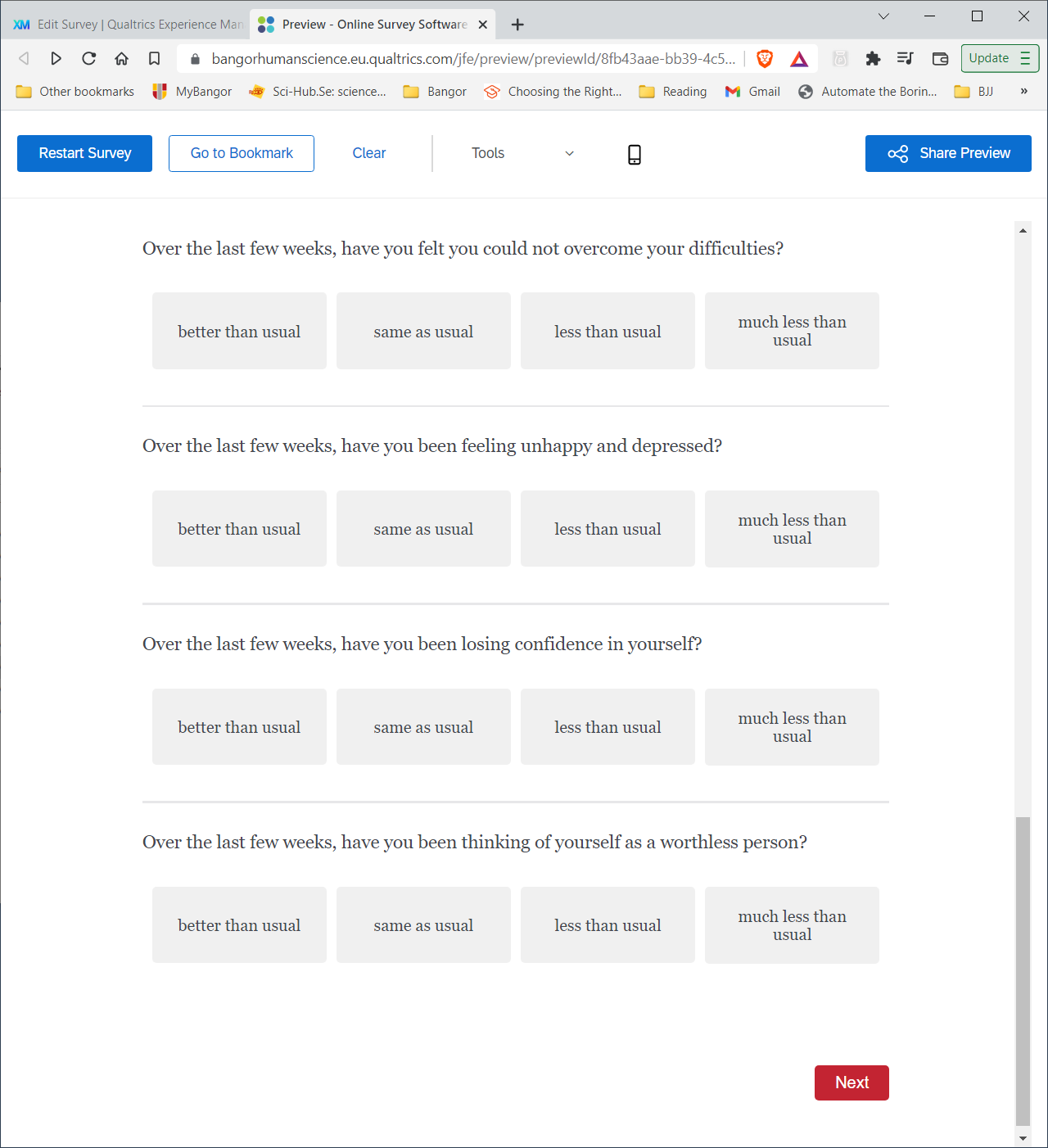


**Wave 1: Alcohol Use Disorders Identification Test (AUDIT):**

The AUDIT is a 10-item questionnaire to assess harmful alcohol use (Saunders et al., 1993). All items are scored from 0 – 4. The final two items are scored 0 for ‘No,’ 2 for ‘Yes, but not in the past year,’ and 4 for ‘Yes, during the past year.’


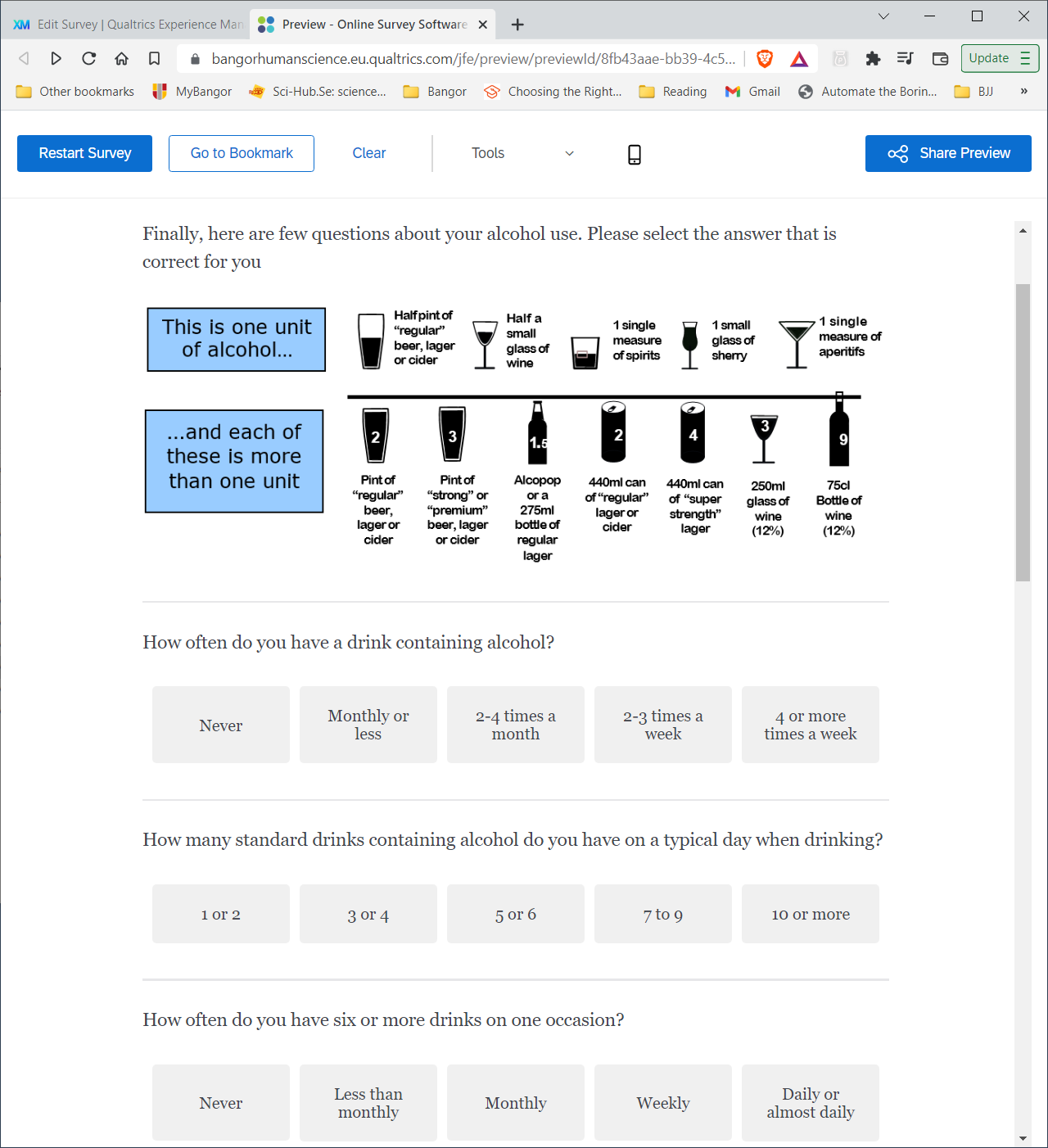


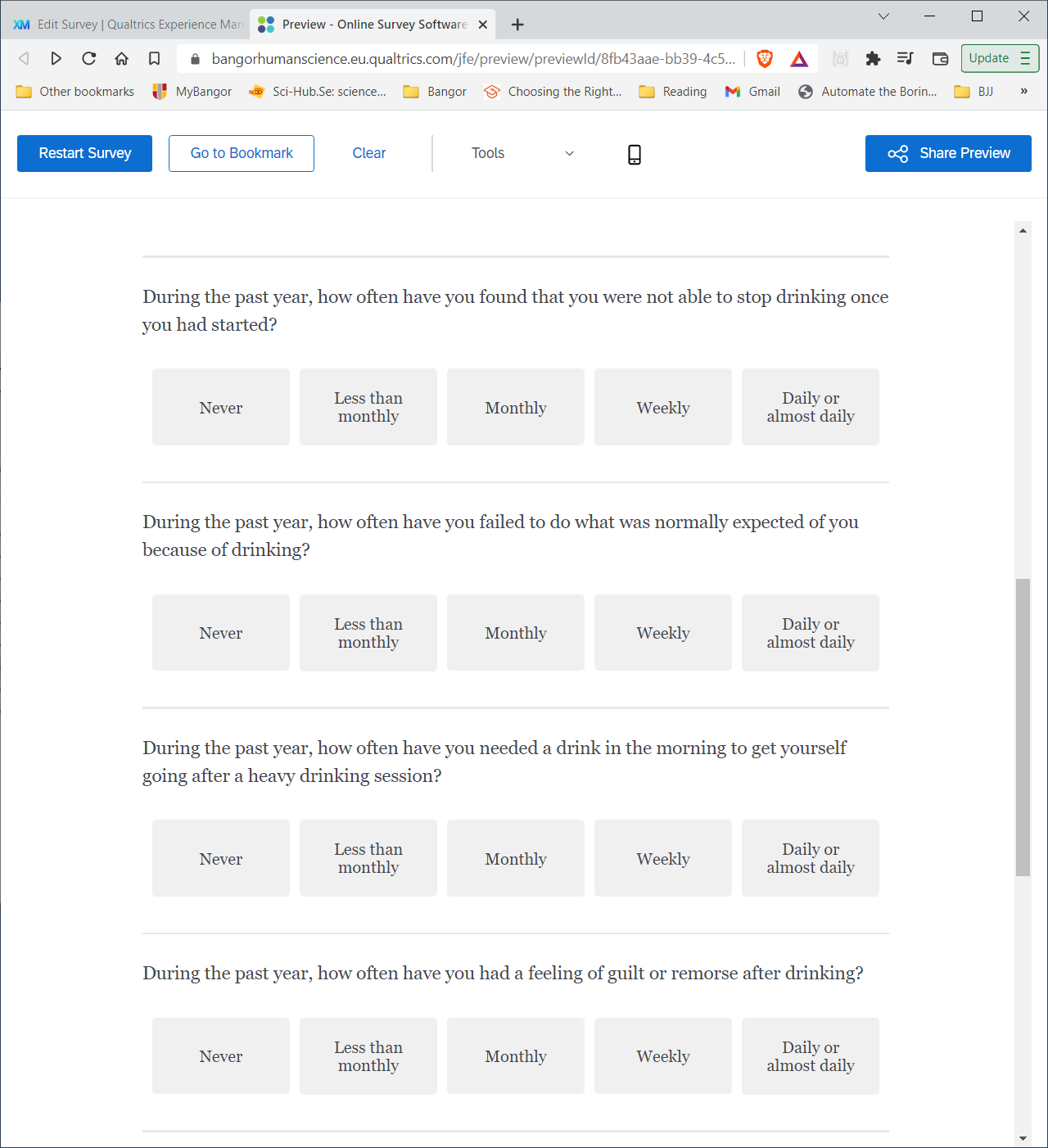

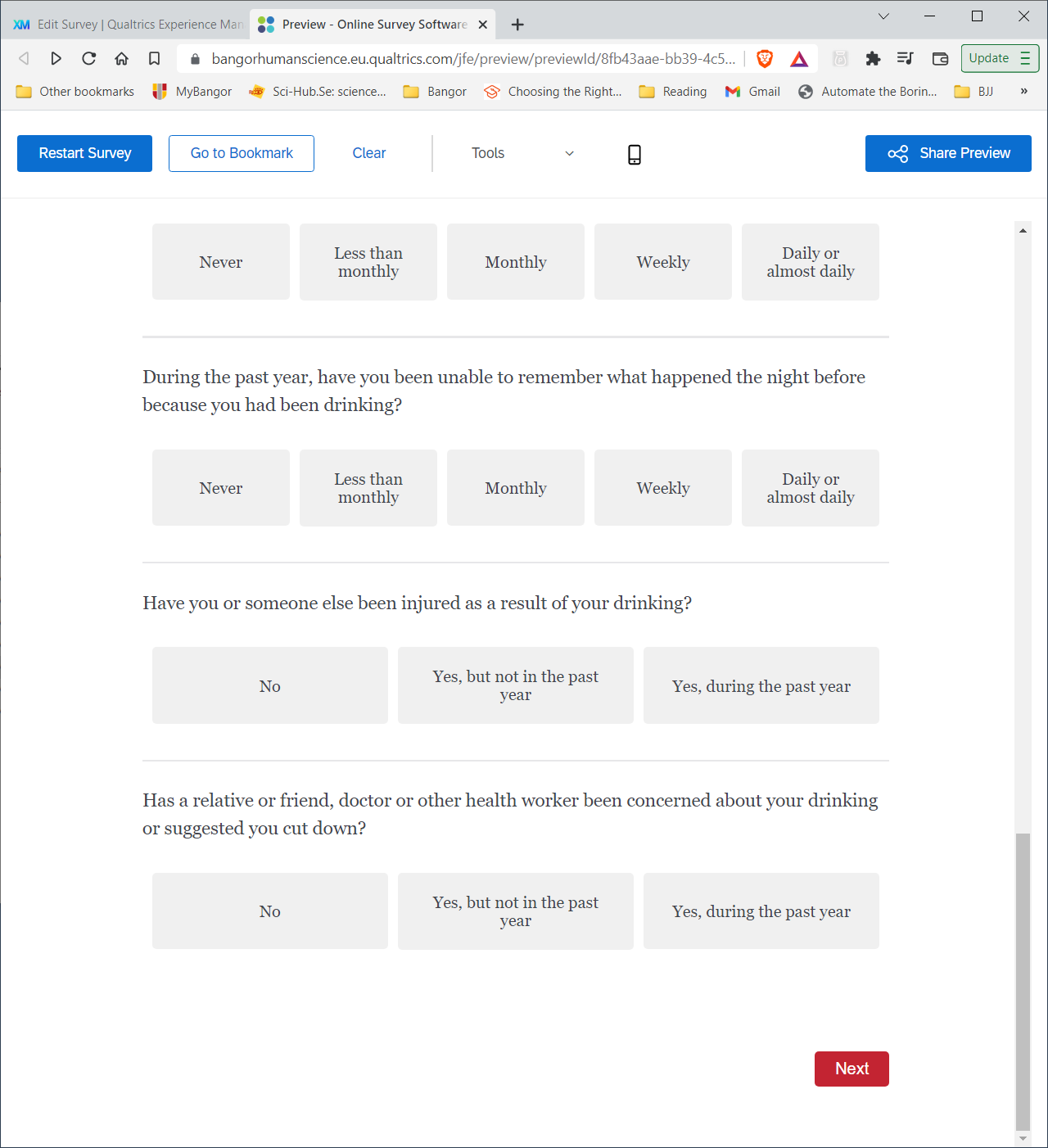


**Wave 1: World Health Organization Five Well-Being Index (WHO-5)**:

Here, participants completed the WHO-5 (Topp et al., 2015), a short-report measure of subjective well-being. Items are scored from 0 (“All of the time”) to 5 (“At no time”).


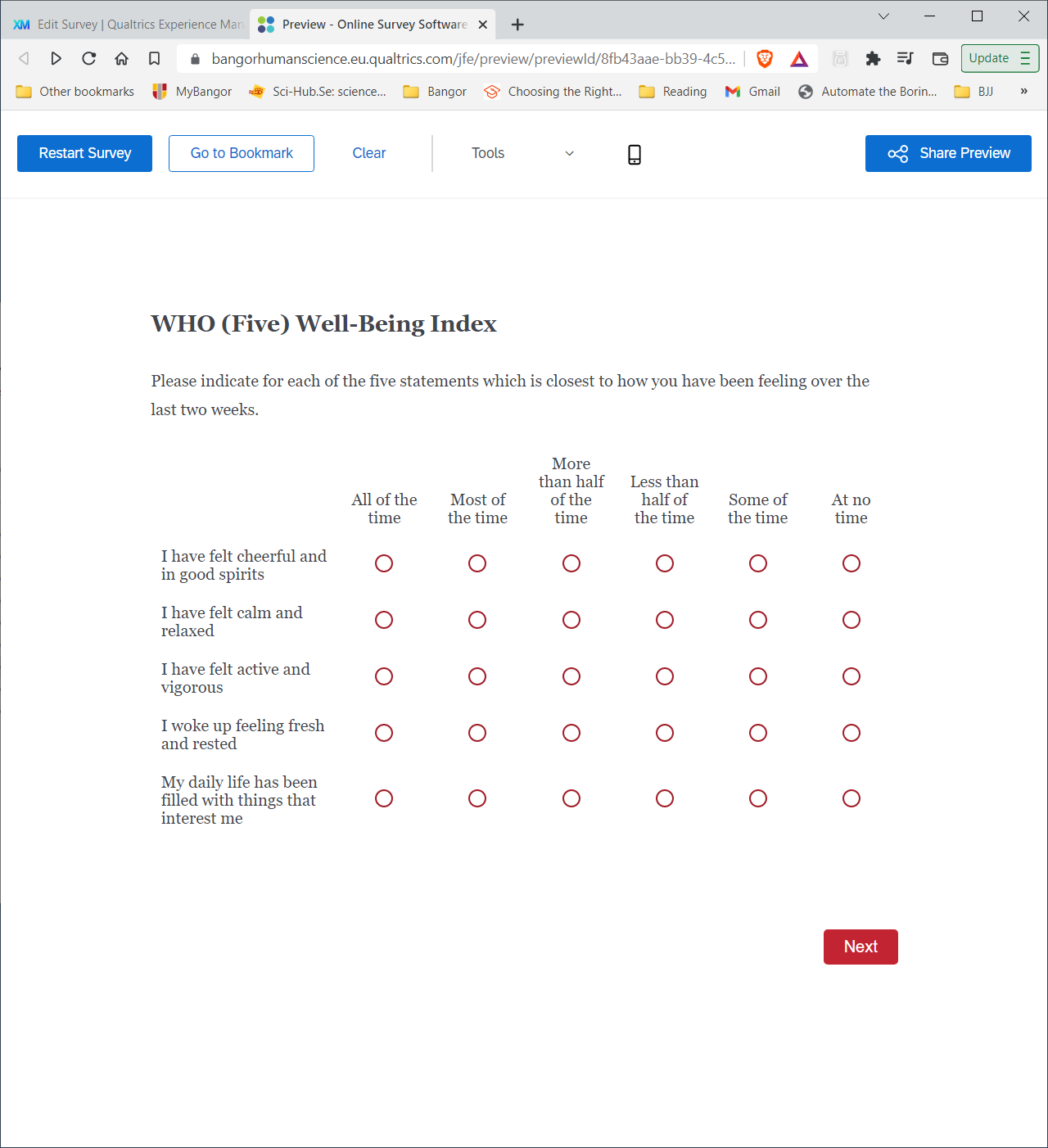


**Wave 1: End of survey page:**

Finally, the participants were thanked for taking part and shown a summary of their game stats and money earned.


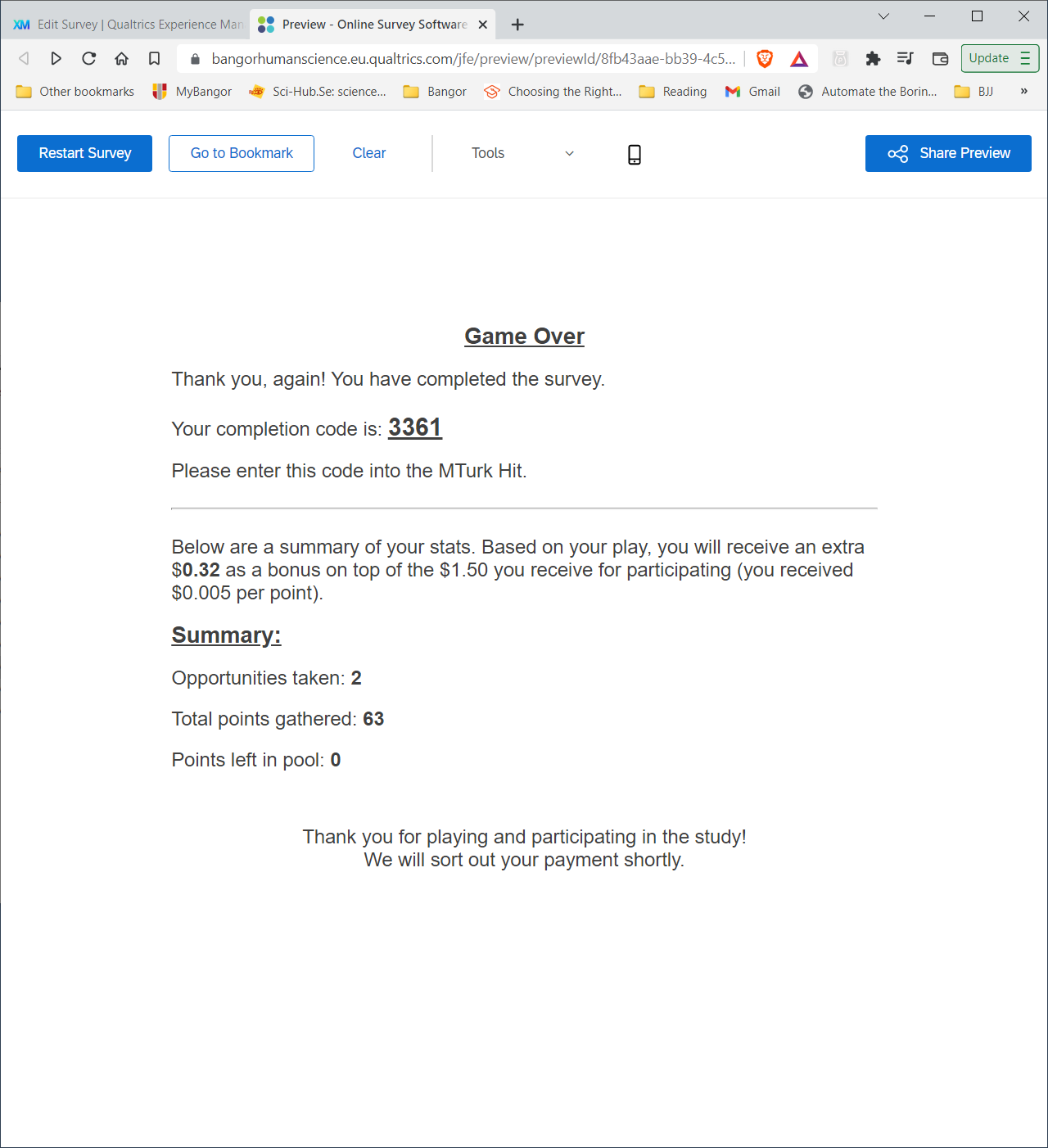


See the following pages for the procedure in wave 2.

After the ED_50_, the two versions of the survey (used in the two waves of data collection) diverge. The following is the rest of the procedure for the **second** **wave** of data collection.

**Wave 2: OECD Financial Literacy Survey:**

Instead of completing the GHQ-12, AUDIT, and WHO-5 questionnaires, participants in the second wave of data collection completed a short form version of the Organisation for Economic Co-operation and Development (OECD) financial literacy survey (Čonková, 2014). Items were scored from 1 (“disagree”) to 5 (“agree”).


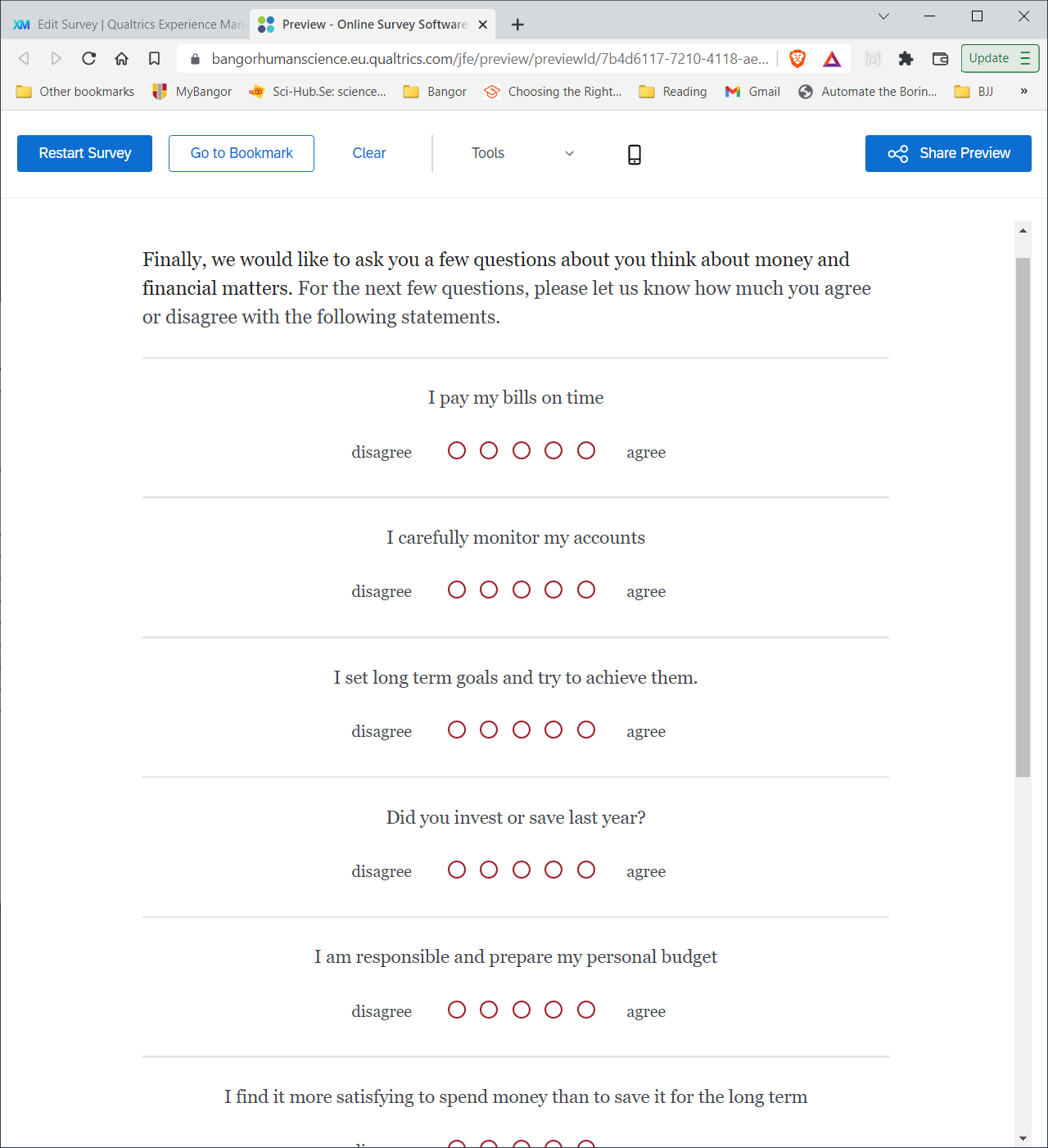


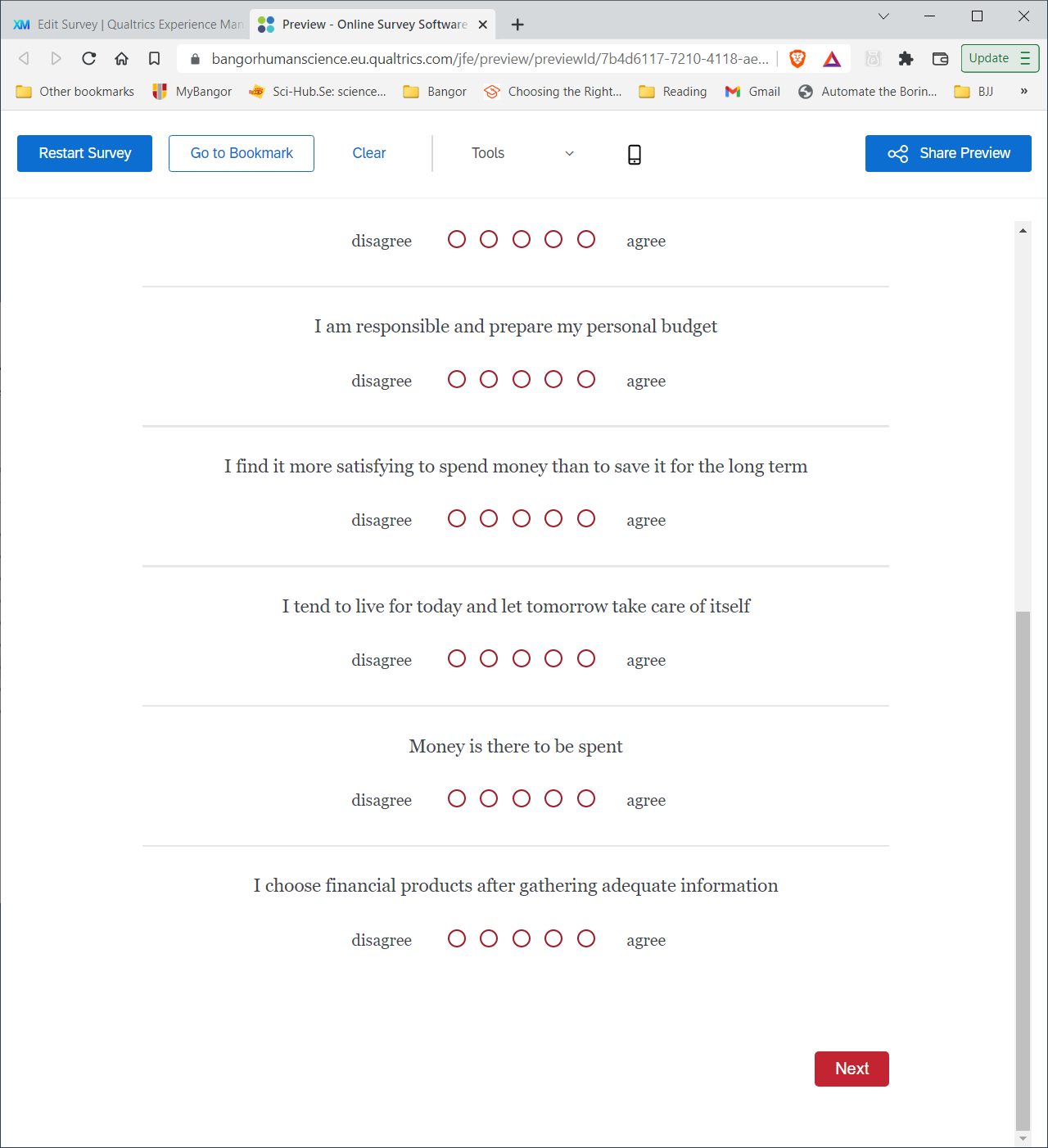


**Wave 2: End of survey page:**

Finally, just as in the first wave of data collection, the participants were thanked for taking part and shown a summary of their game stats and money earned.


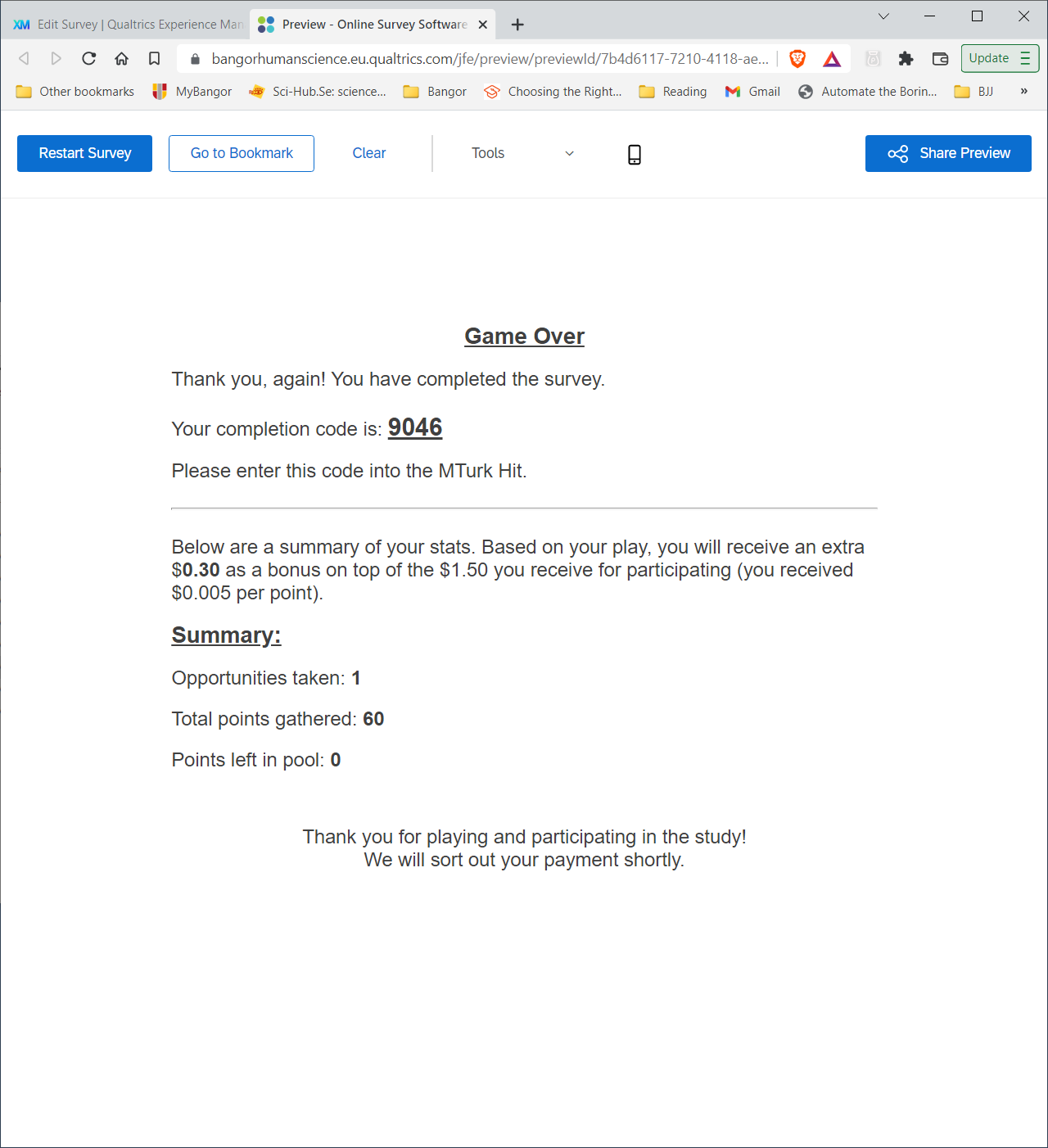


***Experiment 2:***

**Participant information page and consent form:**

Prior to completing the protocol, the participant needed to consent to participate. To do this, each participant was shown the below Information Page. At the end of the page, they agreed to several bullet points (e.g., that they knew they could quit at any point by closing the experiment). The participant agreed to the consent form by clicking ‘Accept’ at the bottom of the page.


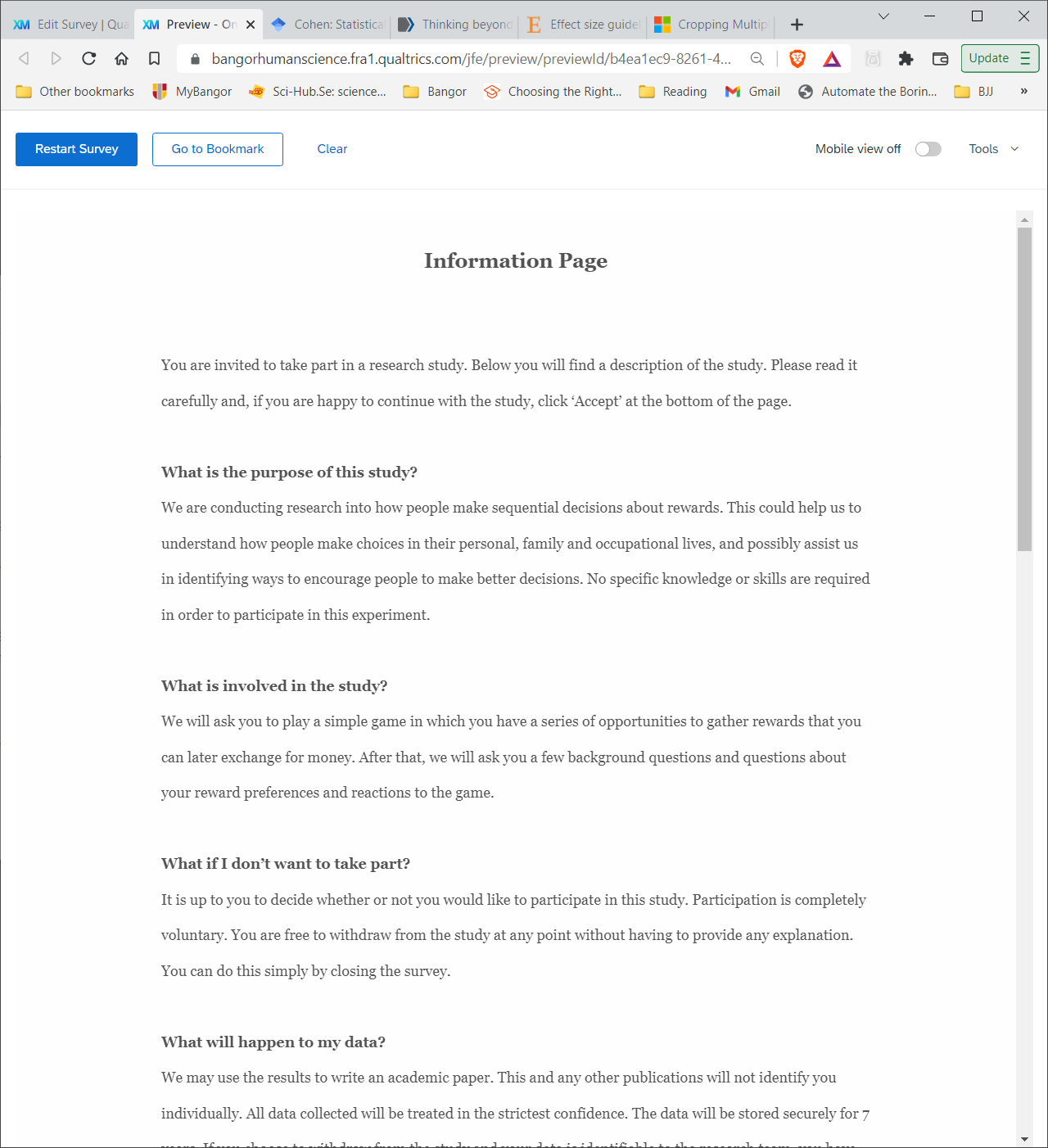


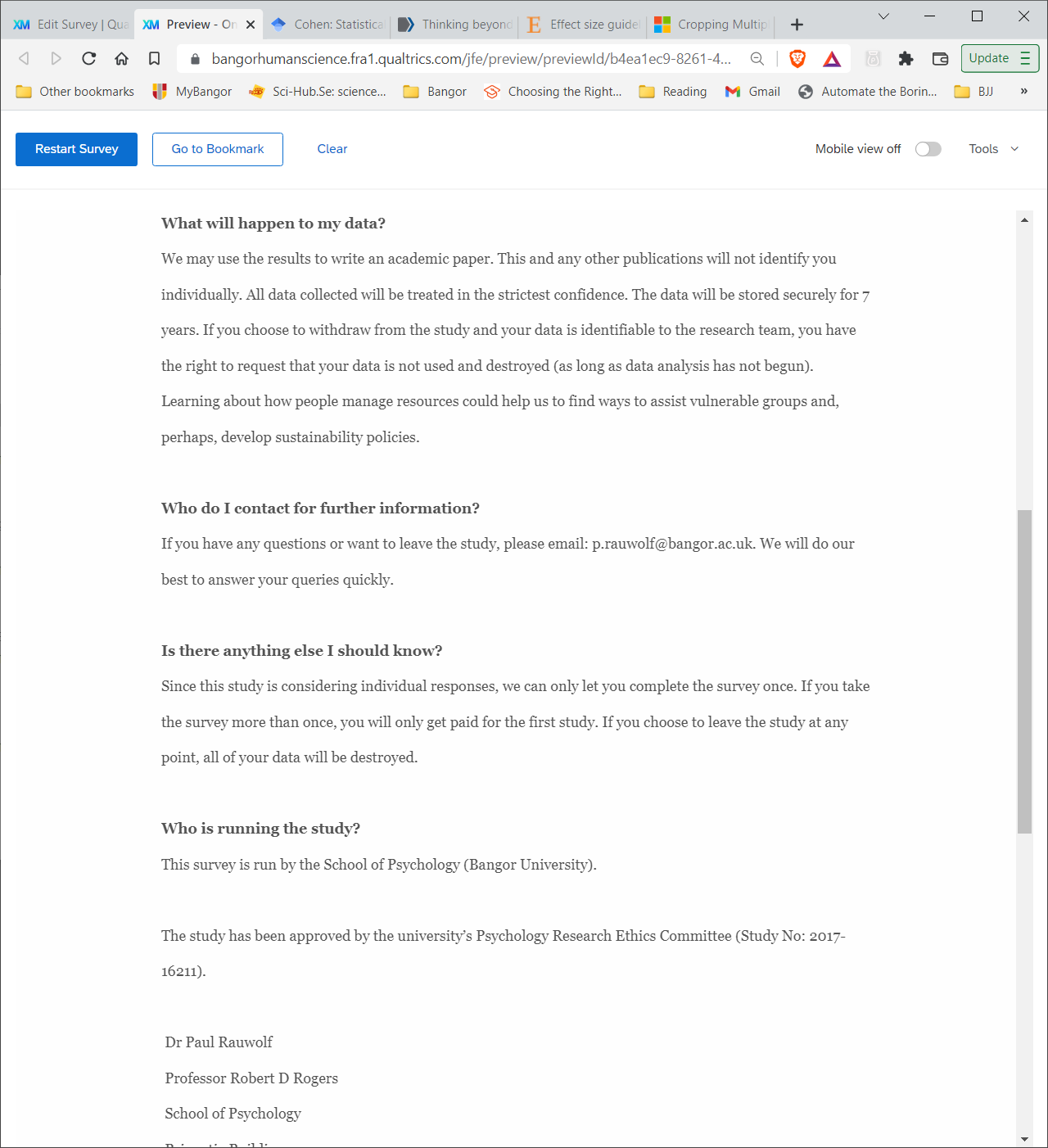


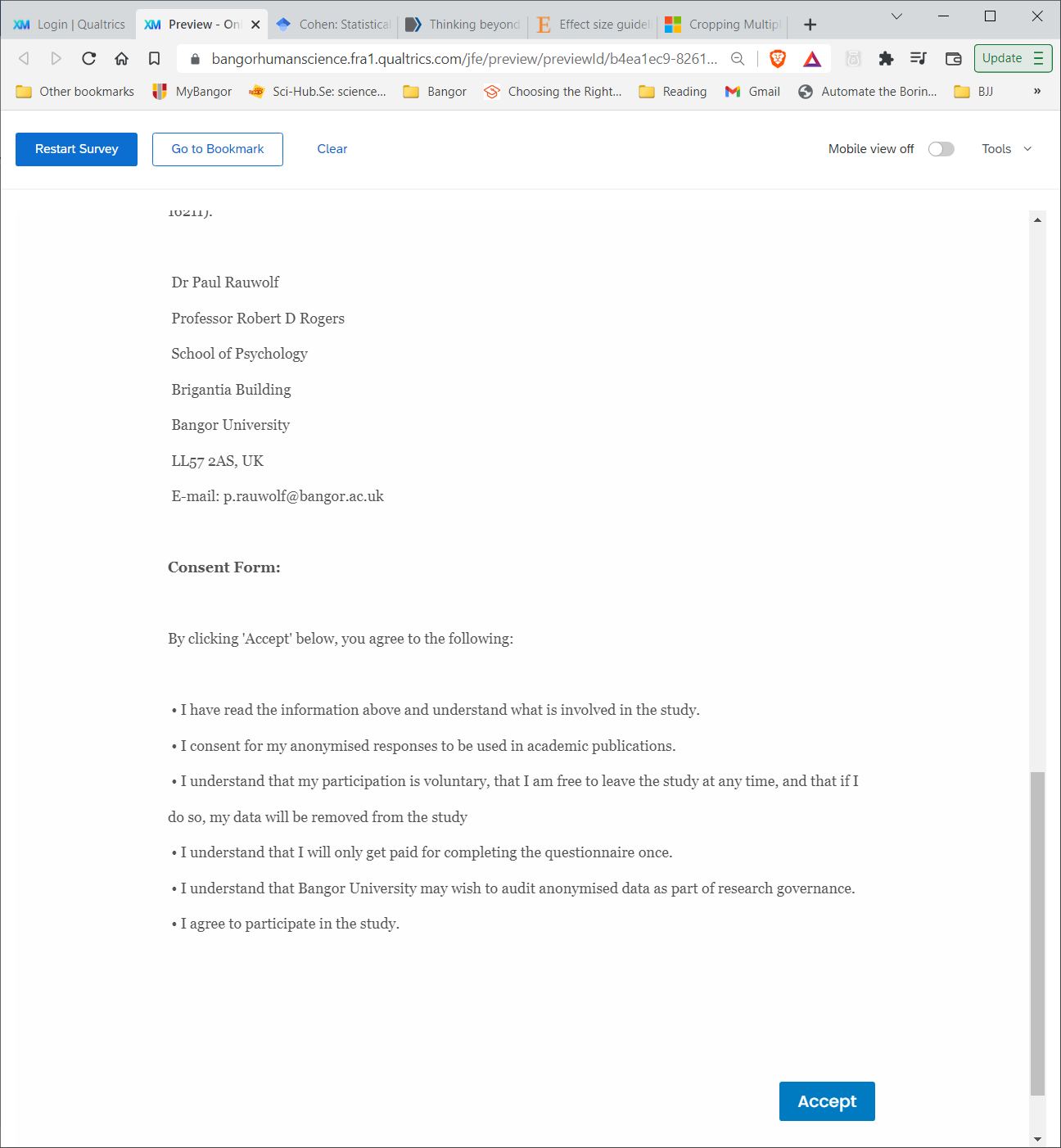


**Participant demographic information:**

Here, we asked participants a couple of demographic questions (gender and age).
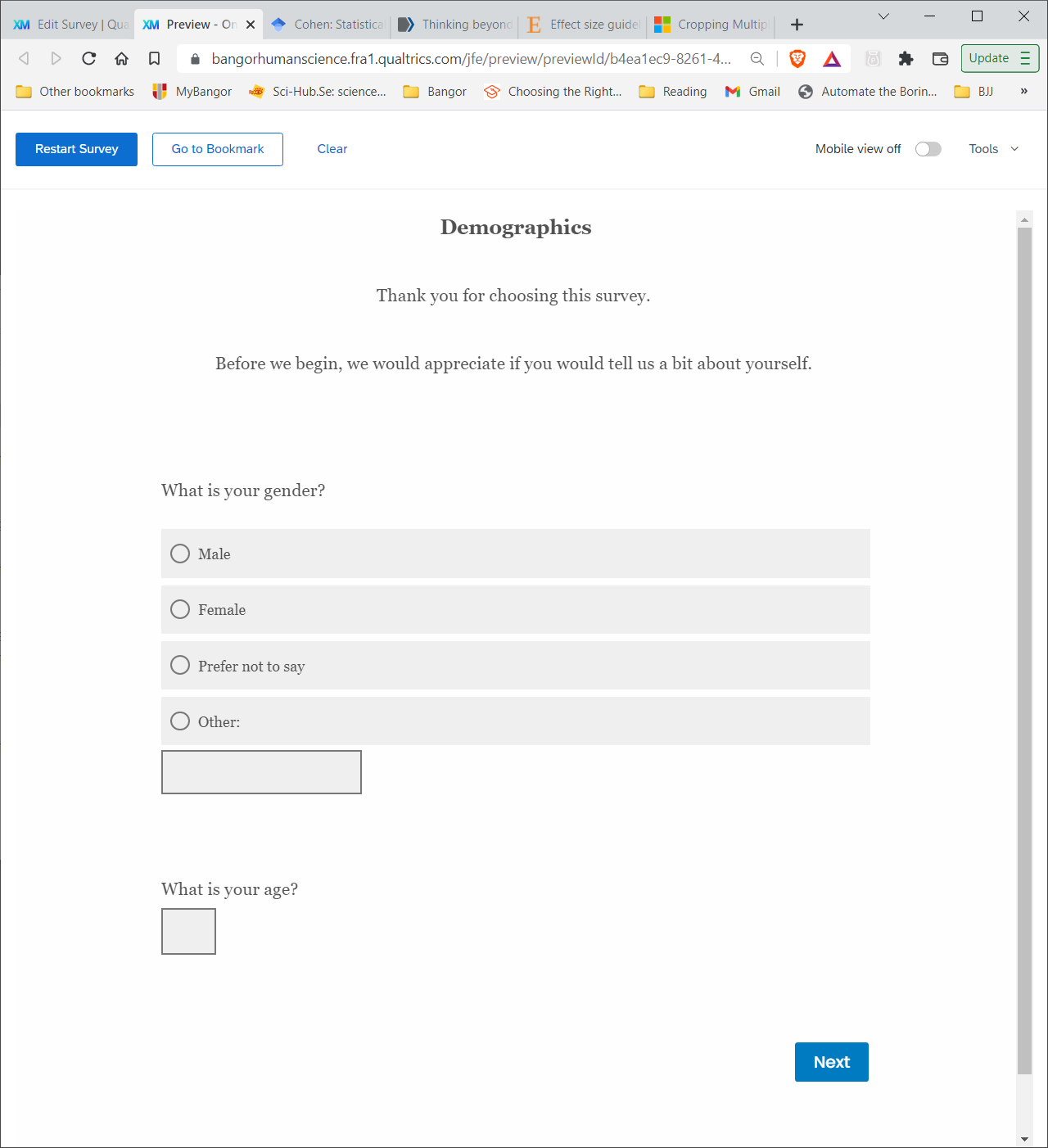


**Instructions:**

Before participants played the game, we offered a tutorial about how the game works and what they were expected to do. We did not tell the participants the replenishment rate, maximum number of rounds, or monetary value of each reward earned. Participants were only told that they could earn a maximum of $3 in each game (participants also played a multi-player version of the game, but this is beyond the scope of this paper).


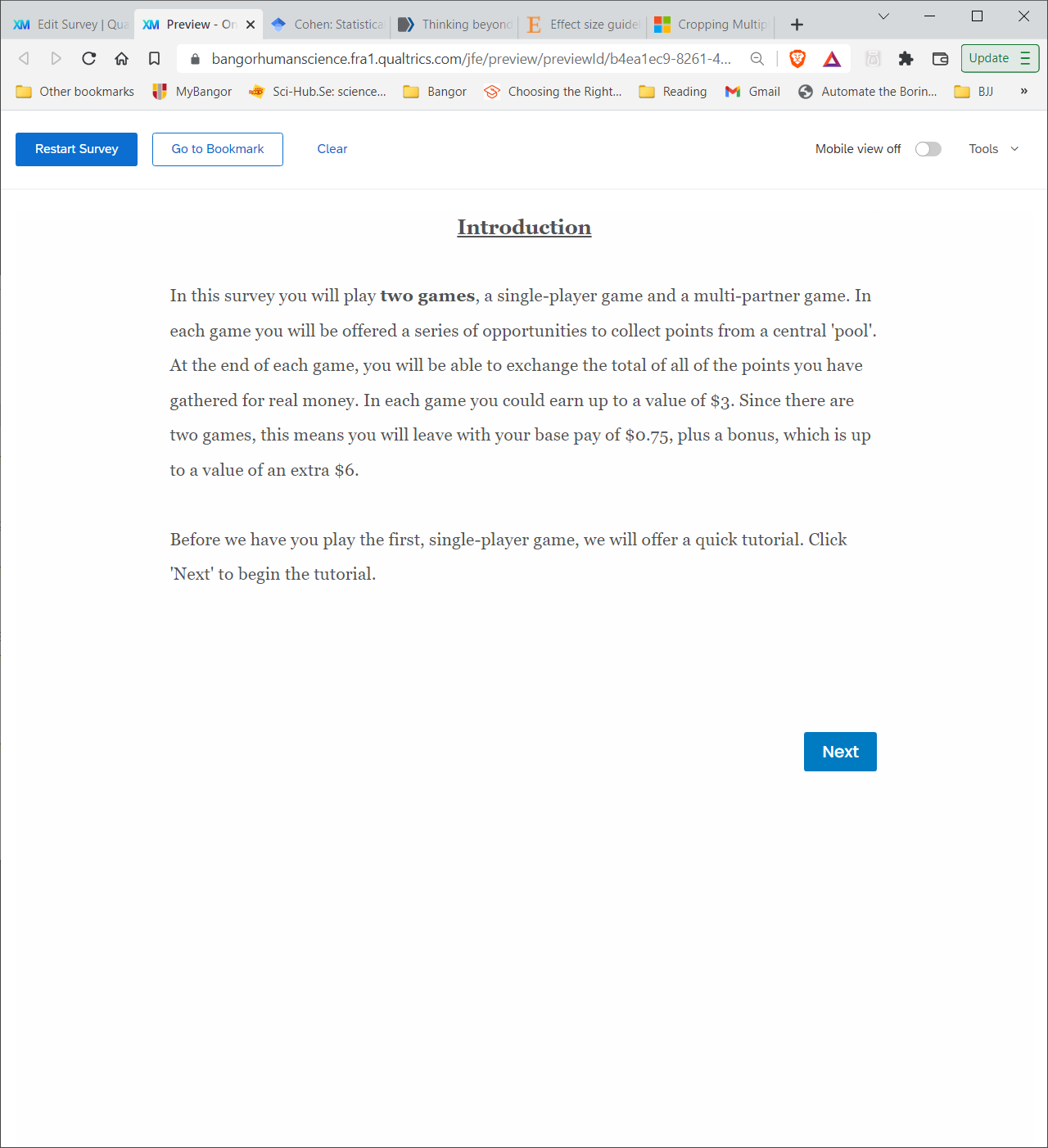


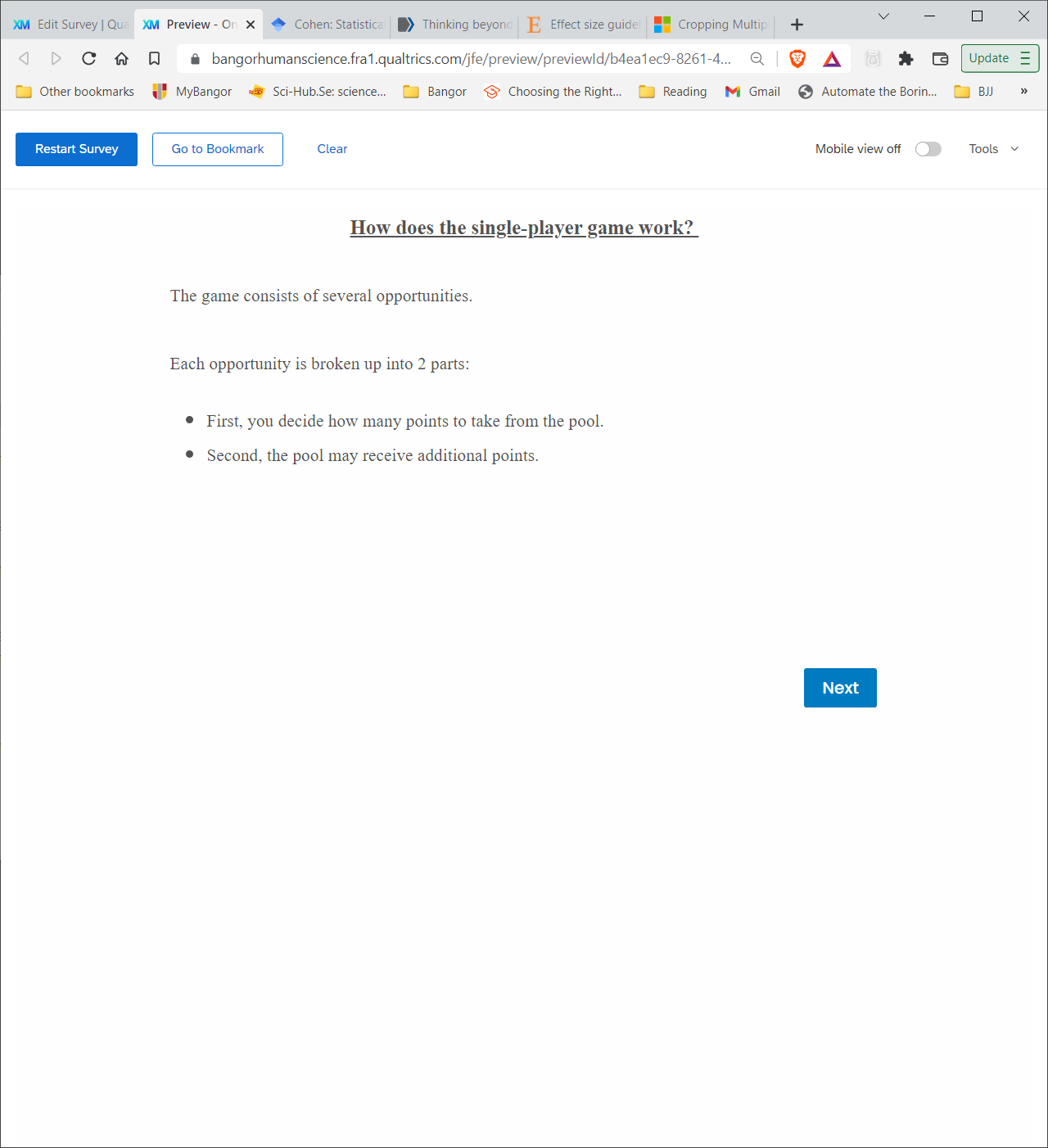


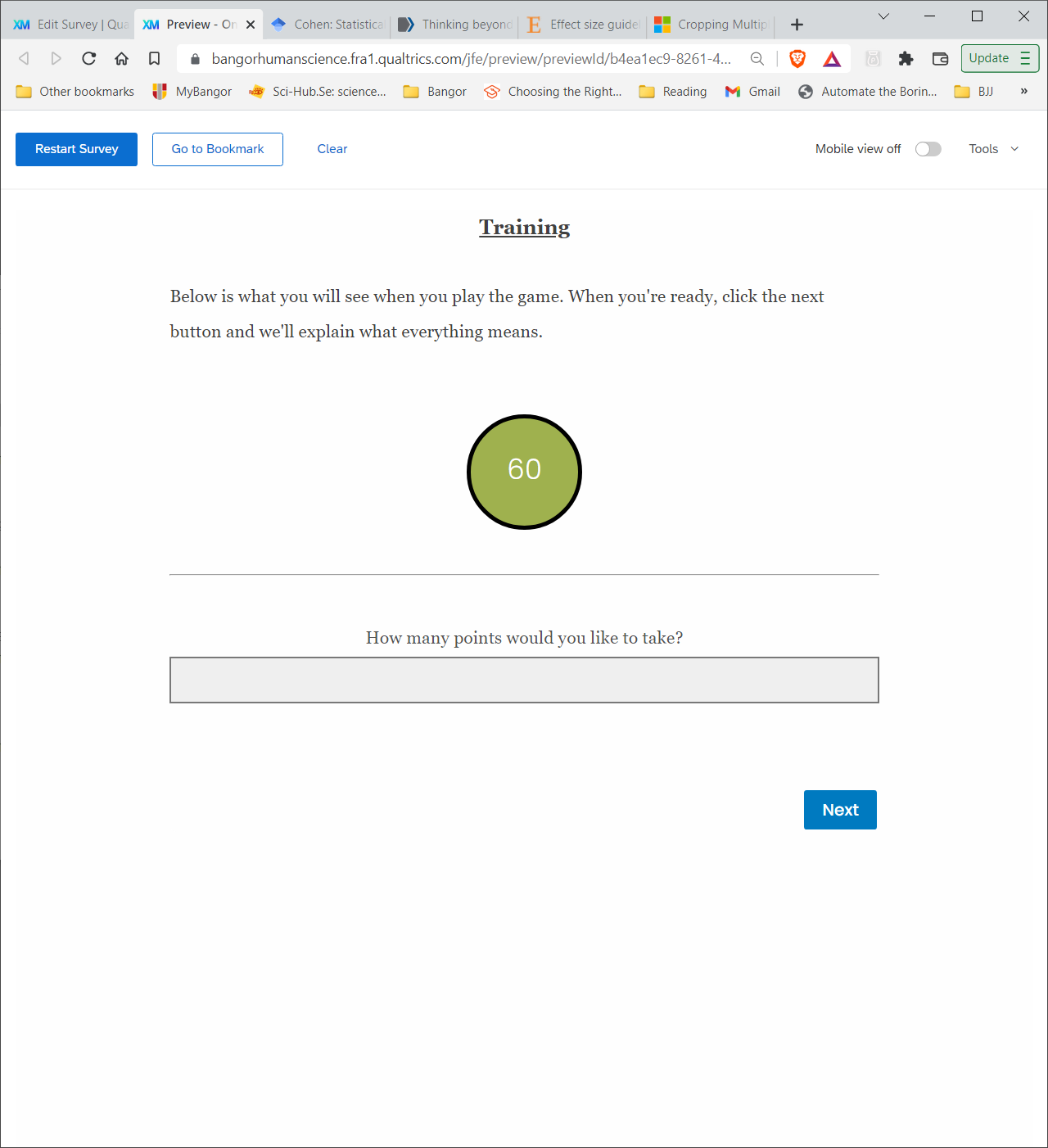


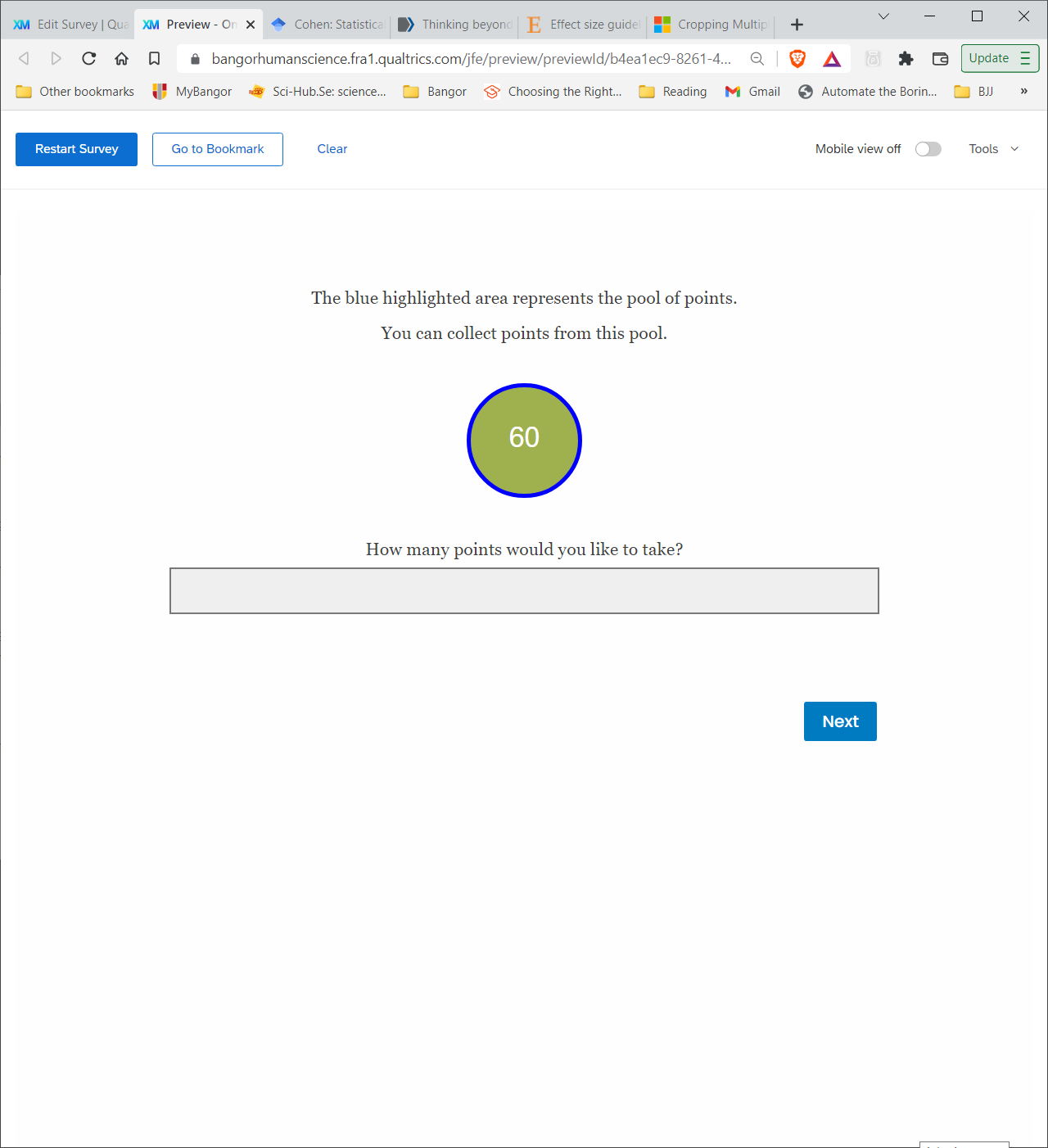


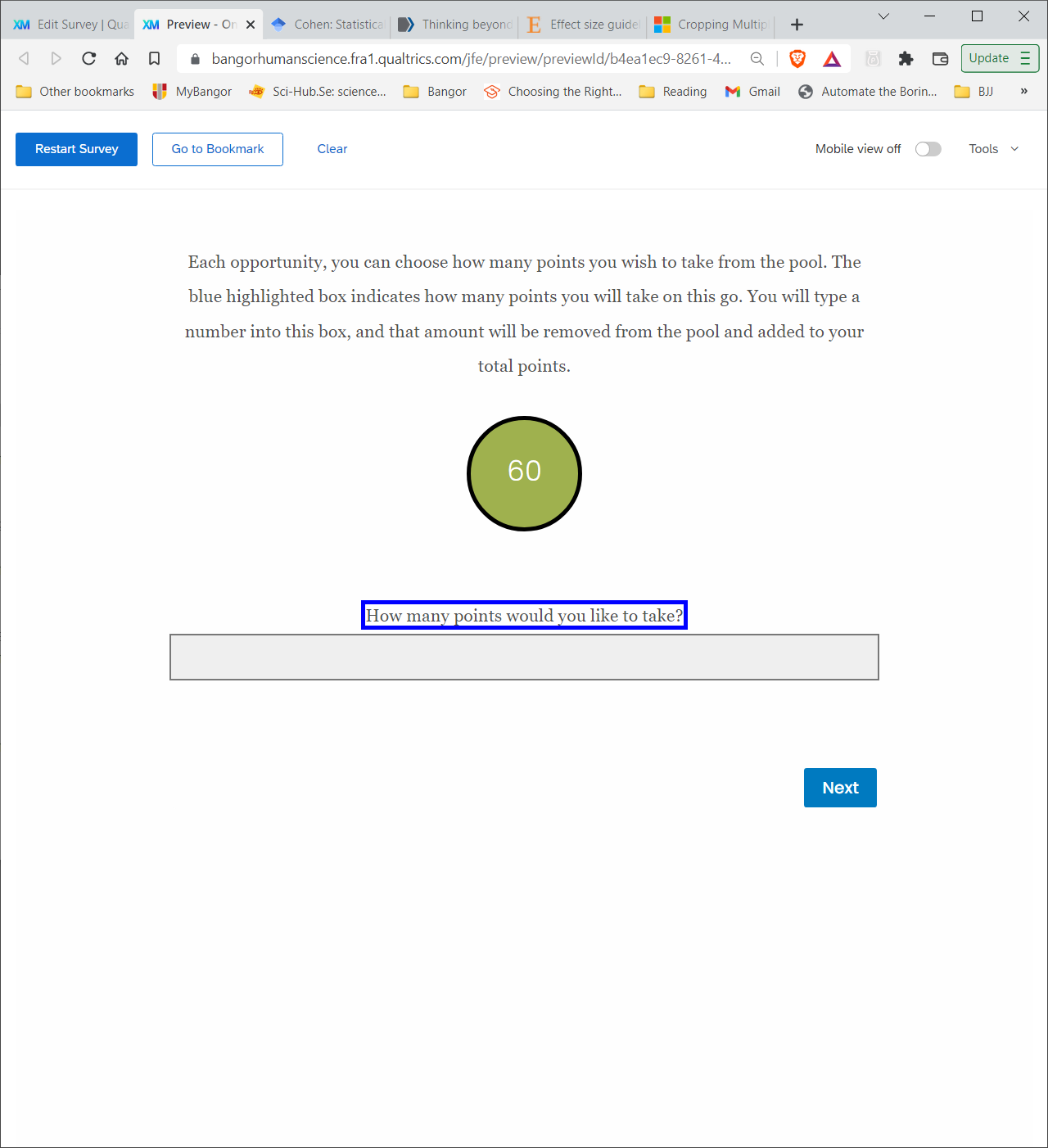


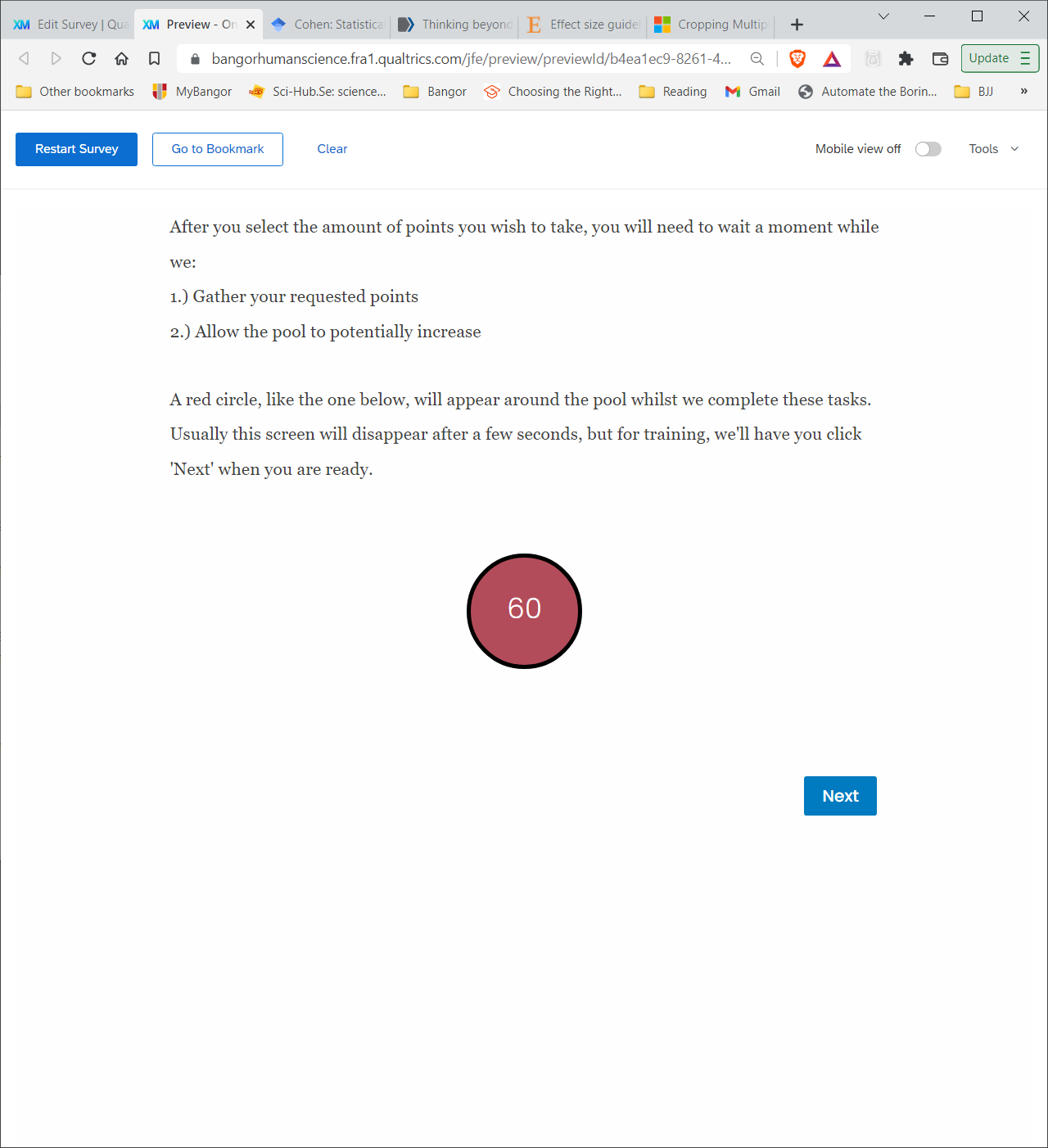


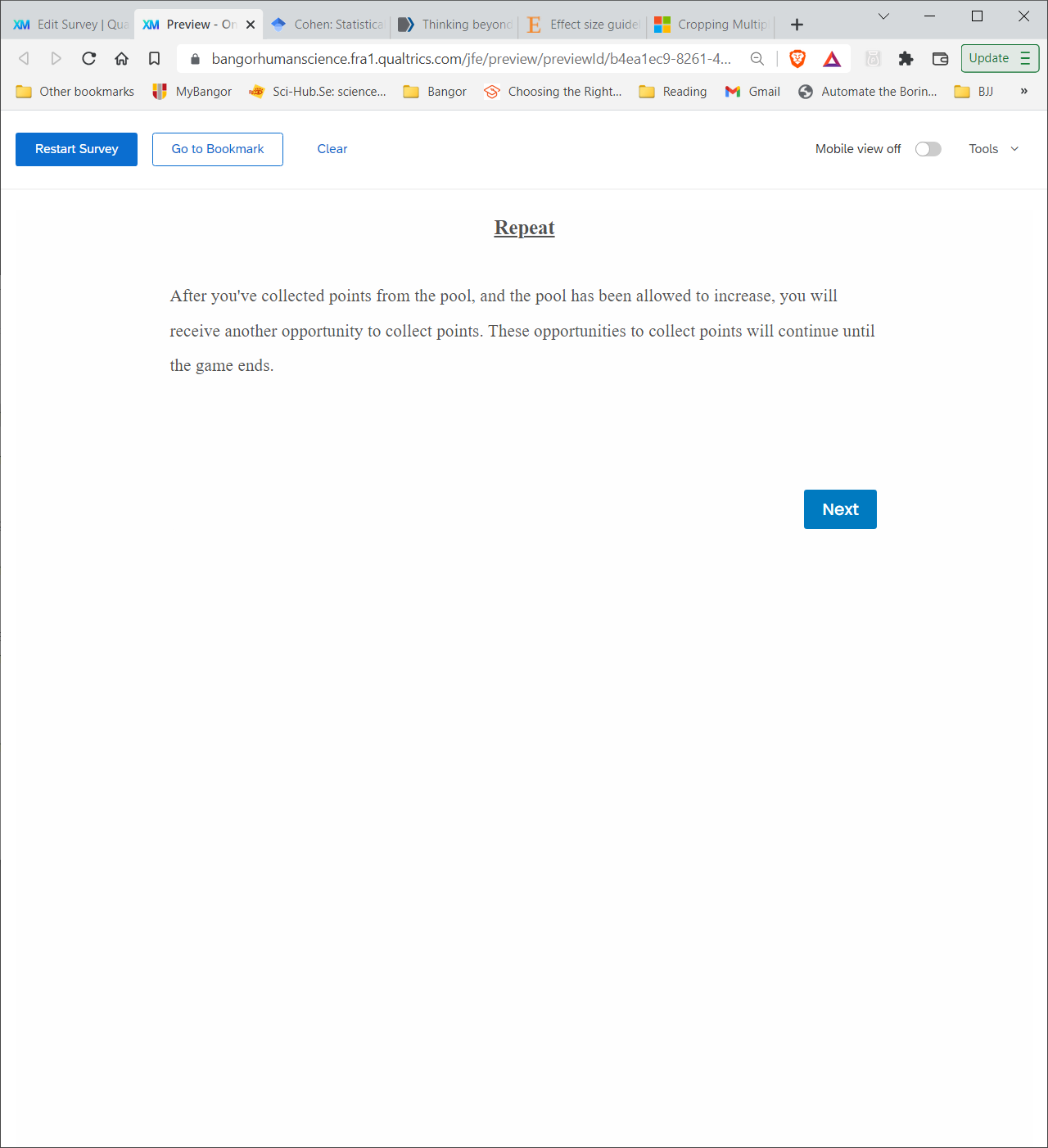


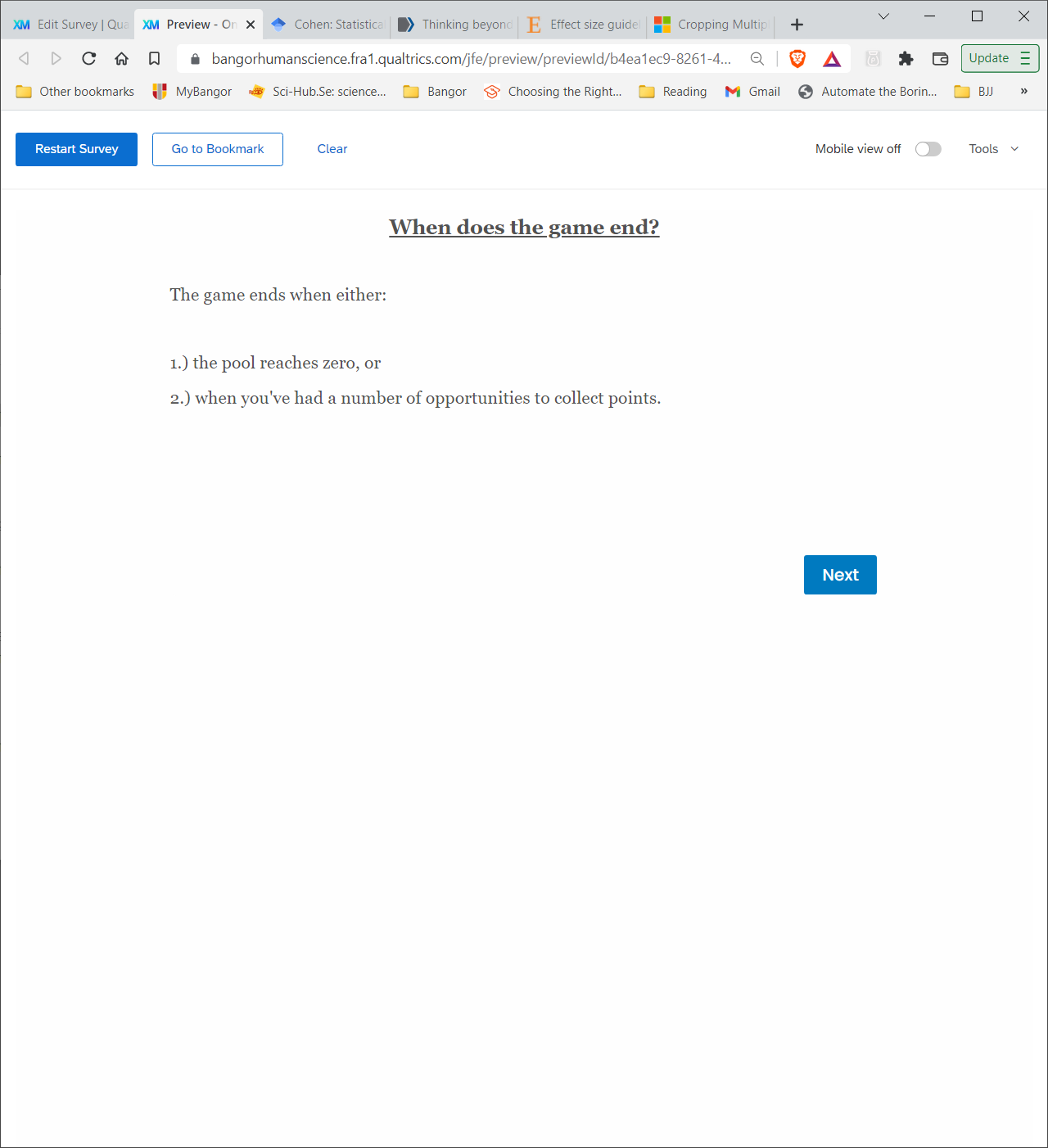


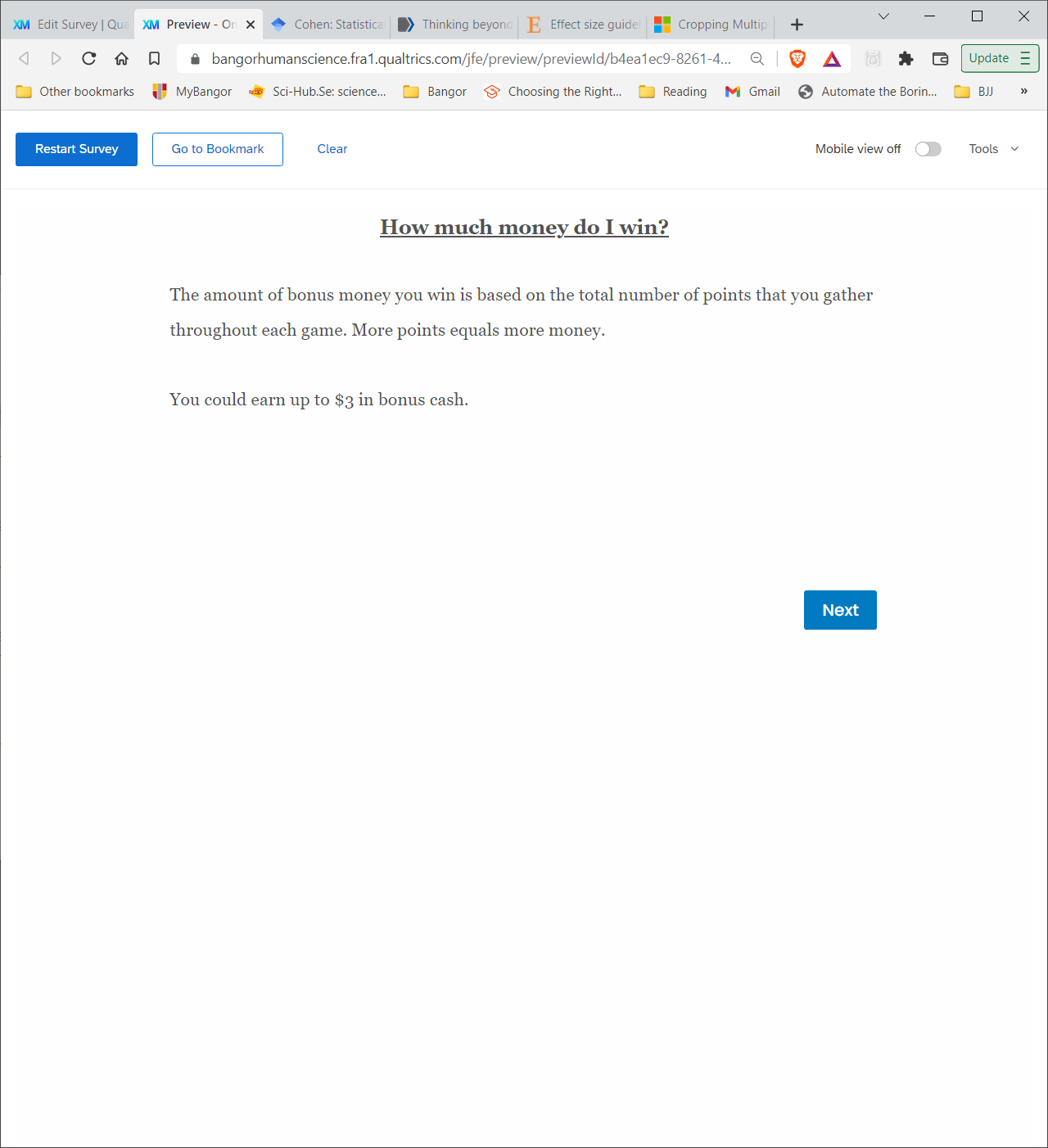


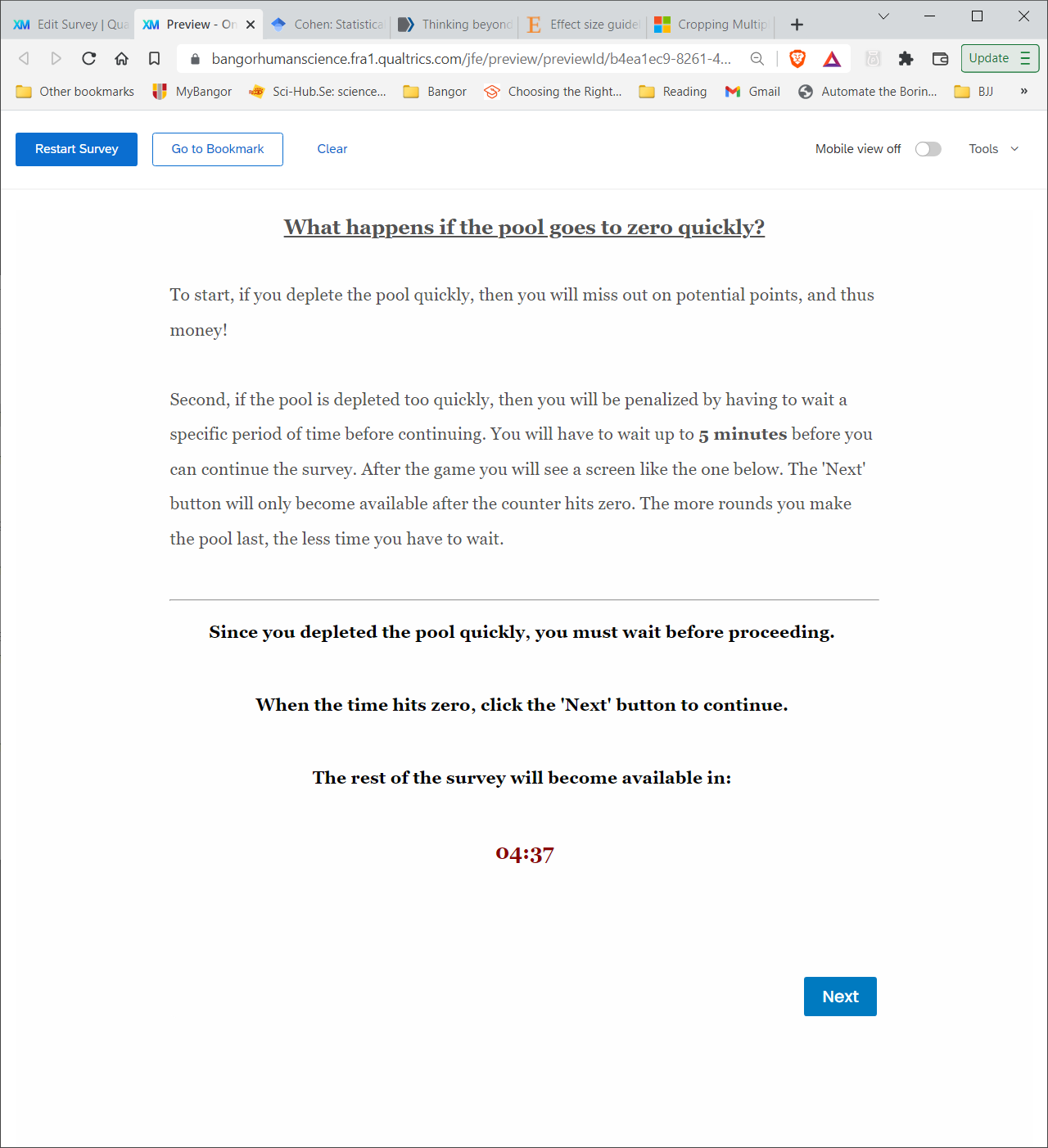


**Questions to assess participant understanding:**

Here, we asked participants four multiple-choice questions to assess their understanding of the game. Participants could not continue past this page until they had selected the correct answer for each of these questions. This page also contained all the instructions previously given to the participant, to revise again if necessary. We haven’t included screenshots of this, as the instructions are identical to those shown above.


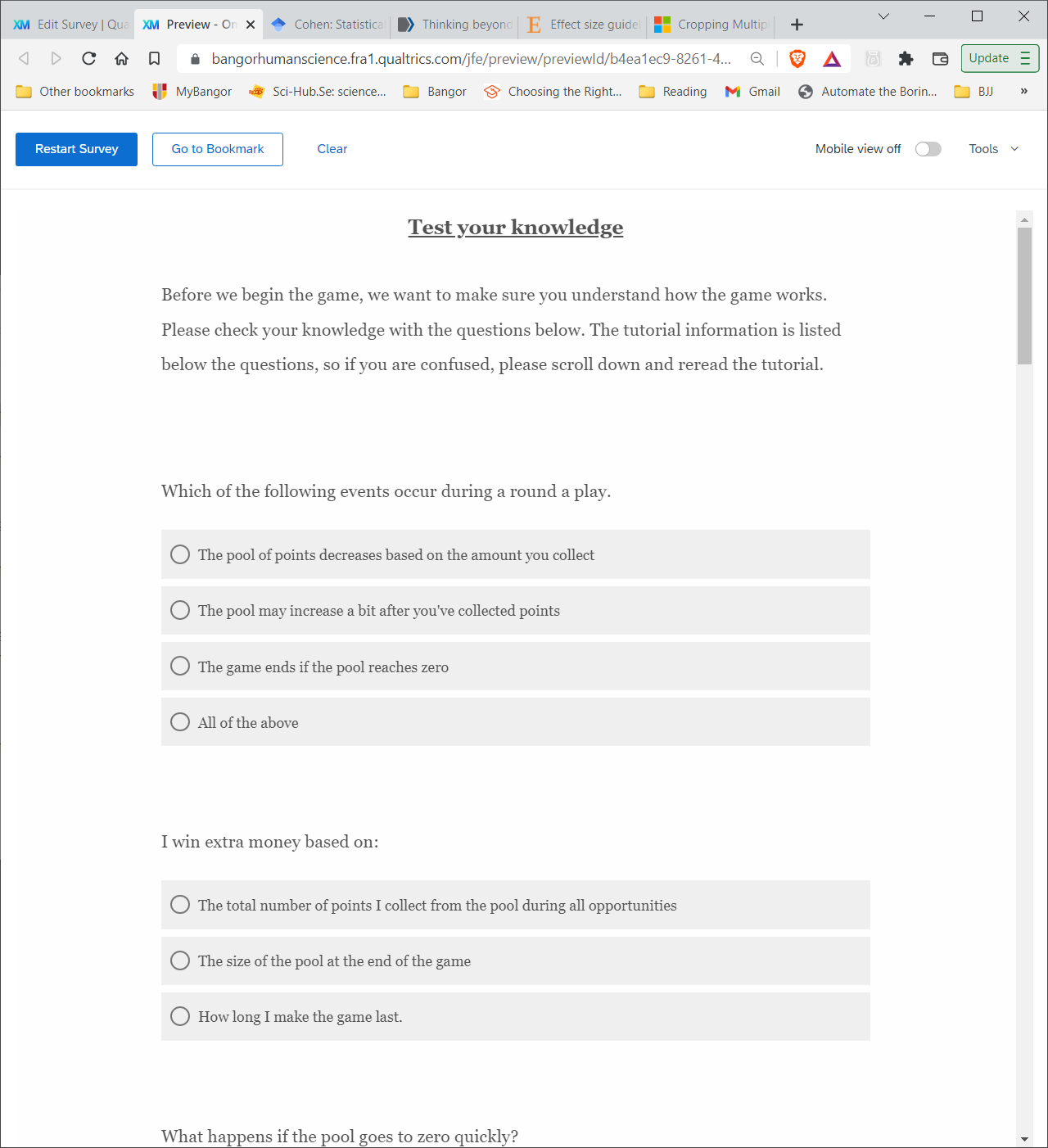


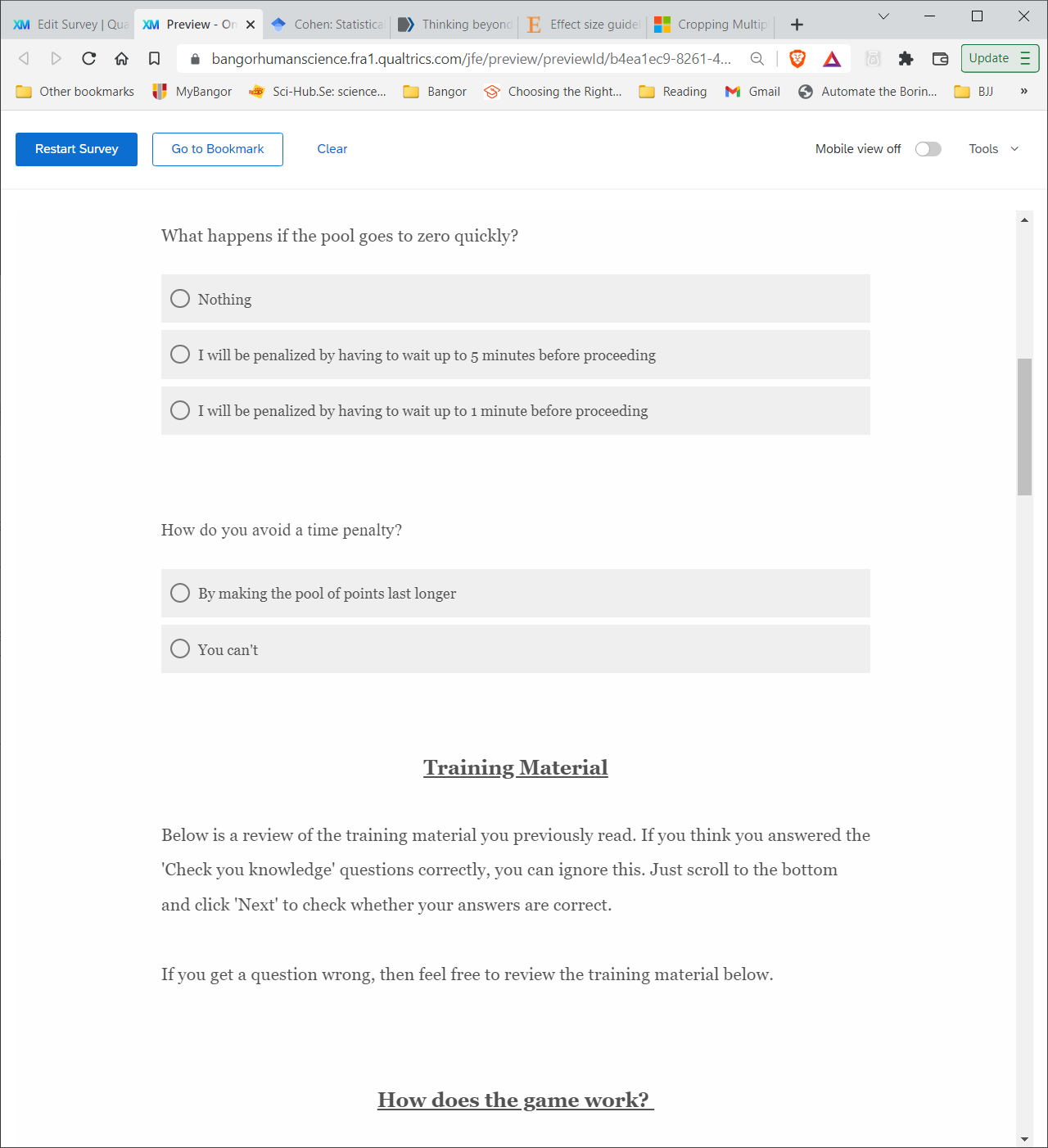


**Playing the game:**

Once they had answered the ‘Test your knowledge’ questions correctly, participants played the game. In these screenshots, we show an example of a participant who lasted two rounds in the game.


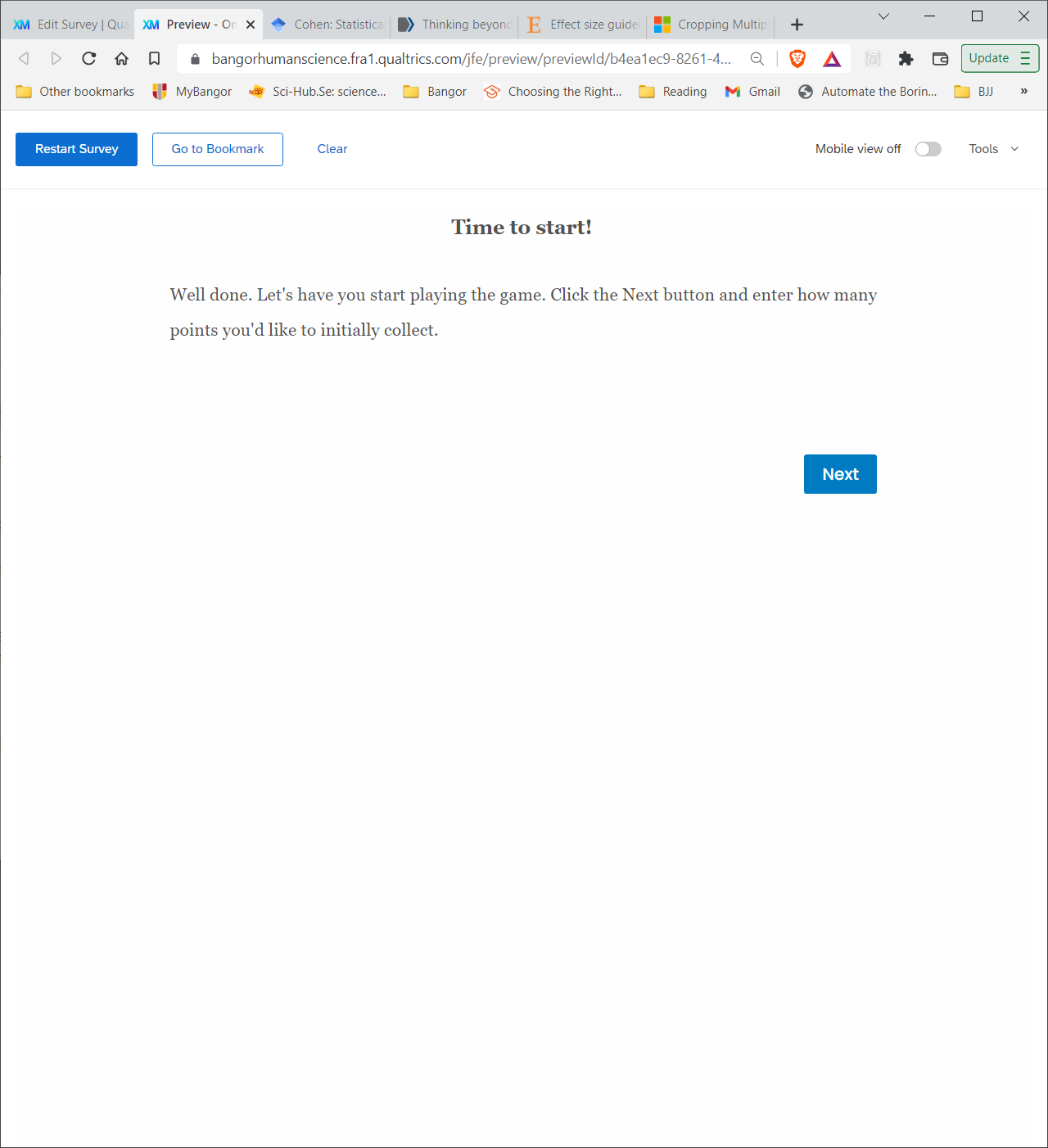


The resource always started at its maximum capacity of 60. Participants typed the number of rewards they wanted to harvest into the grey box, and then clicked ‘Next’ to harvest these rewards.


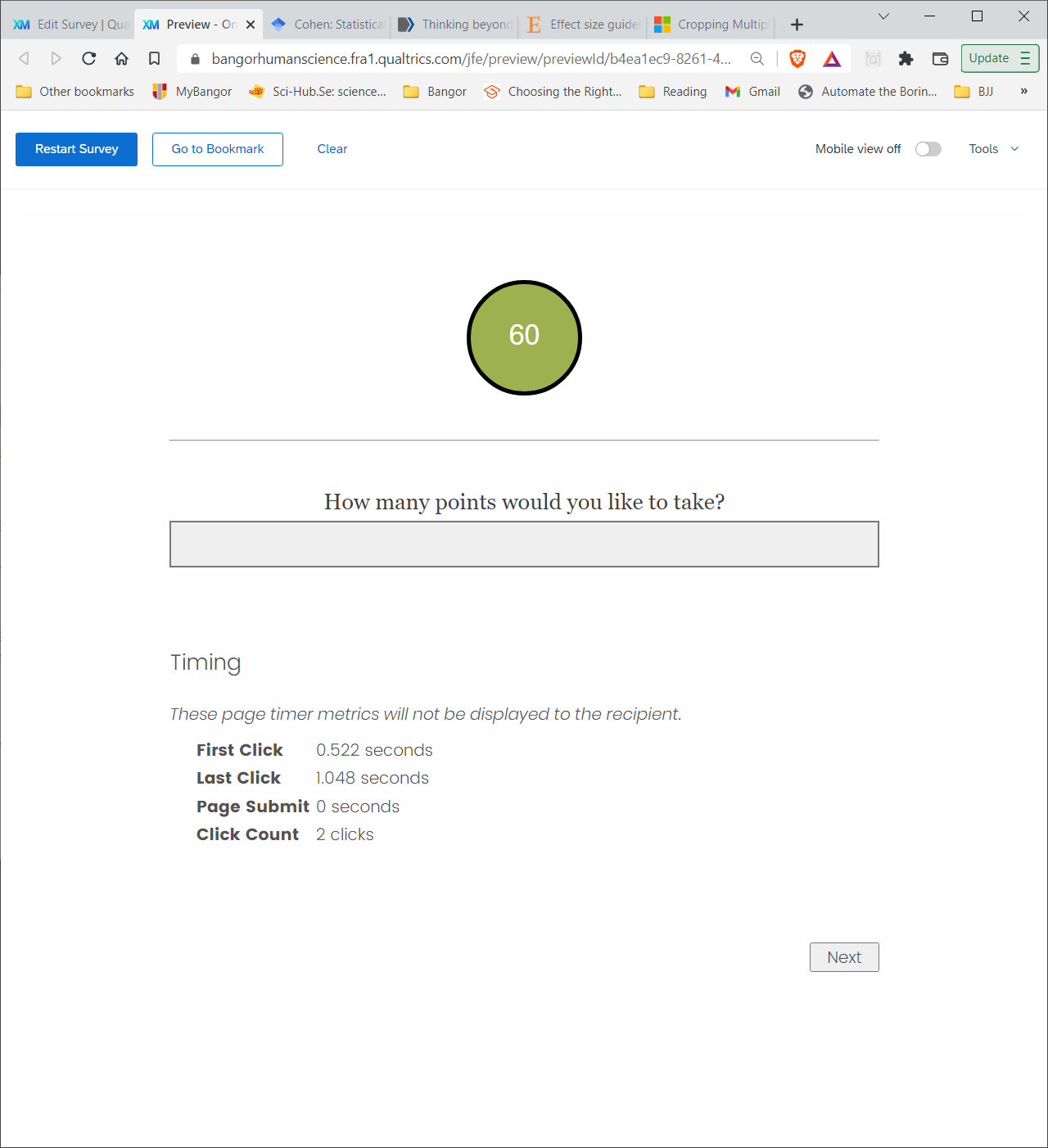


As an example, a participant might take 15 rewards from the resource in the first round.


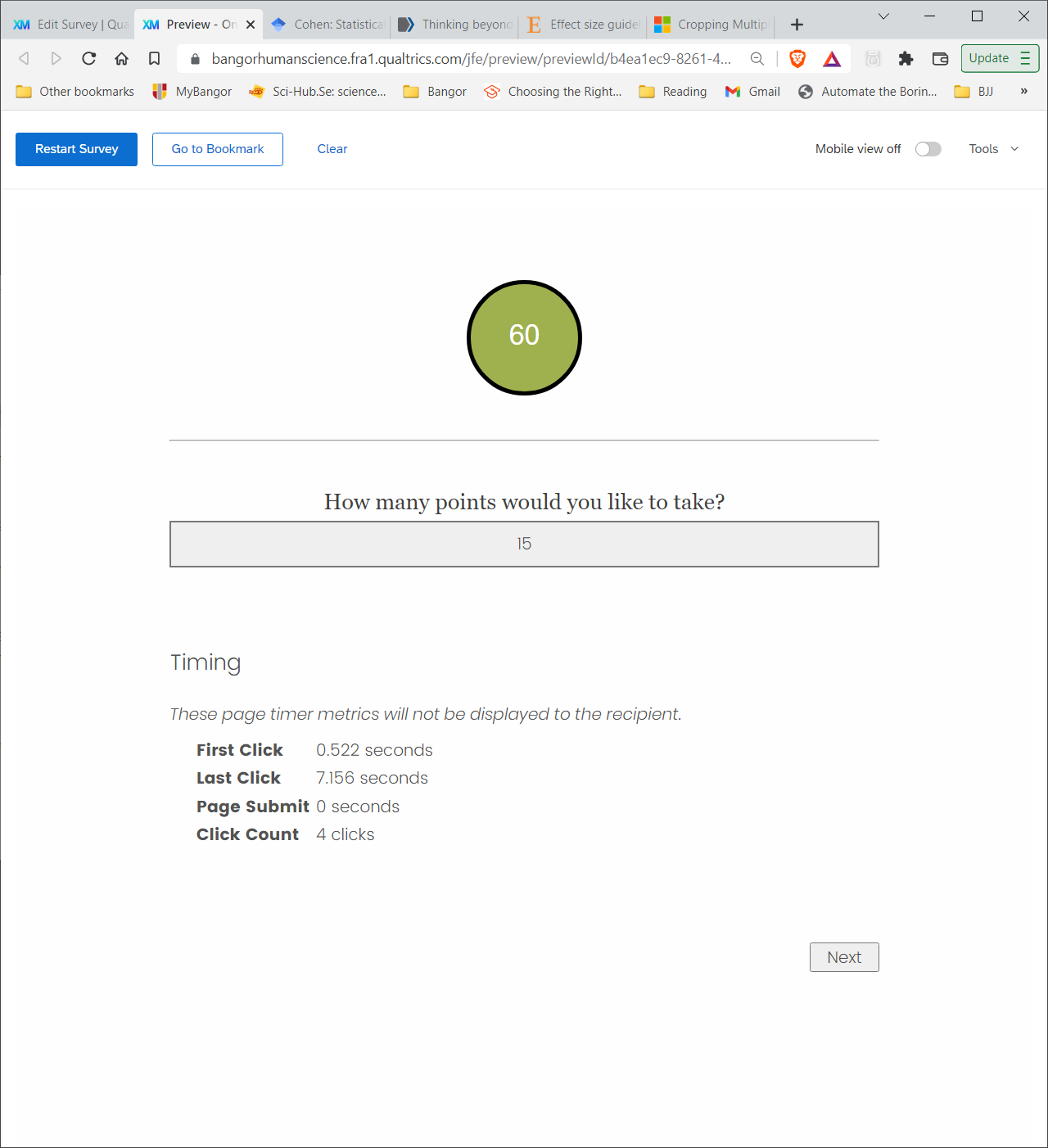


Once the participant clicked ‘Next’, they would see this page for a couple of seconds.


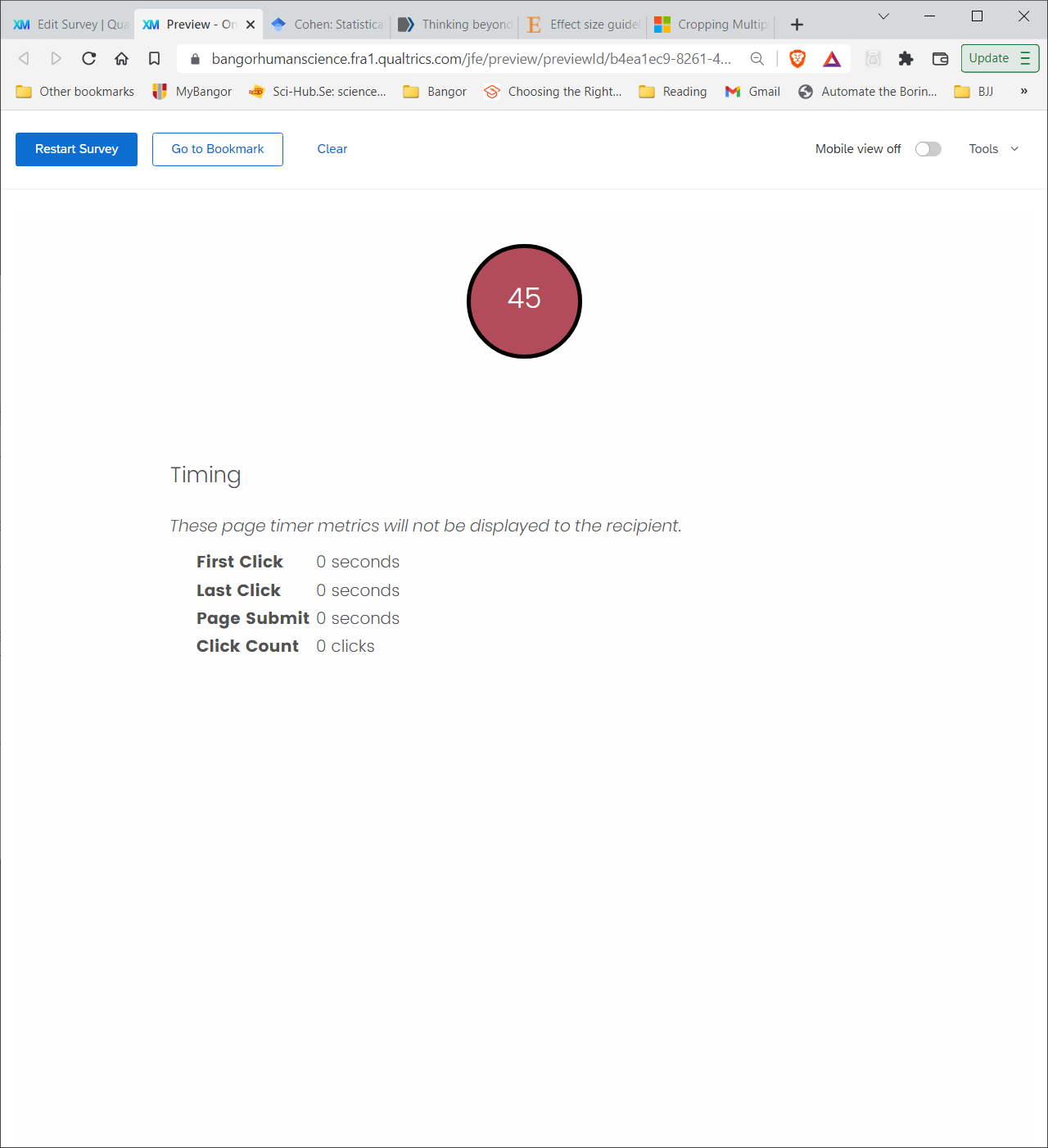


The resource would then replenish by around 15%.


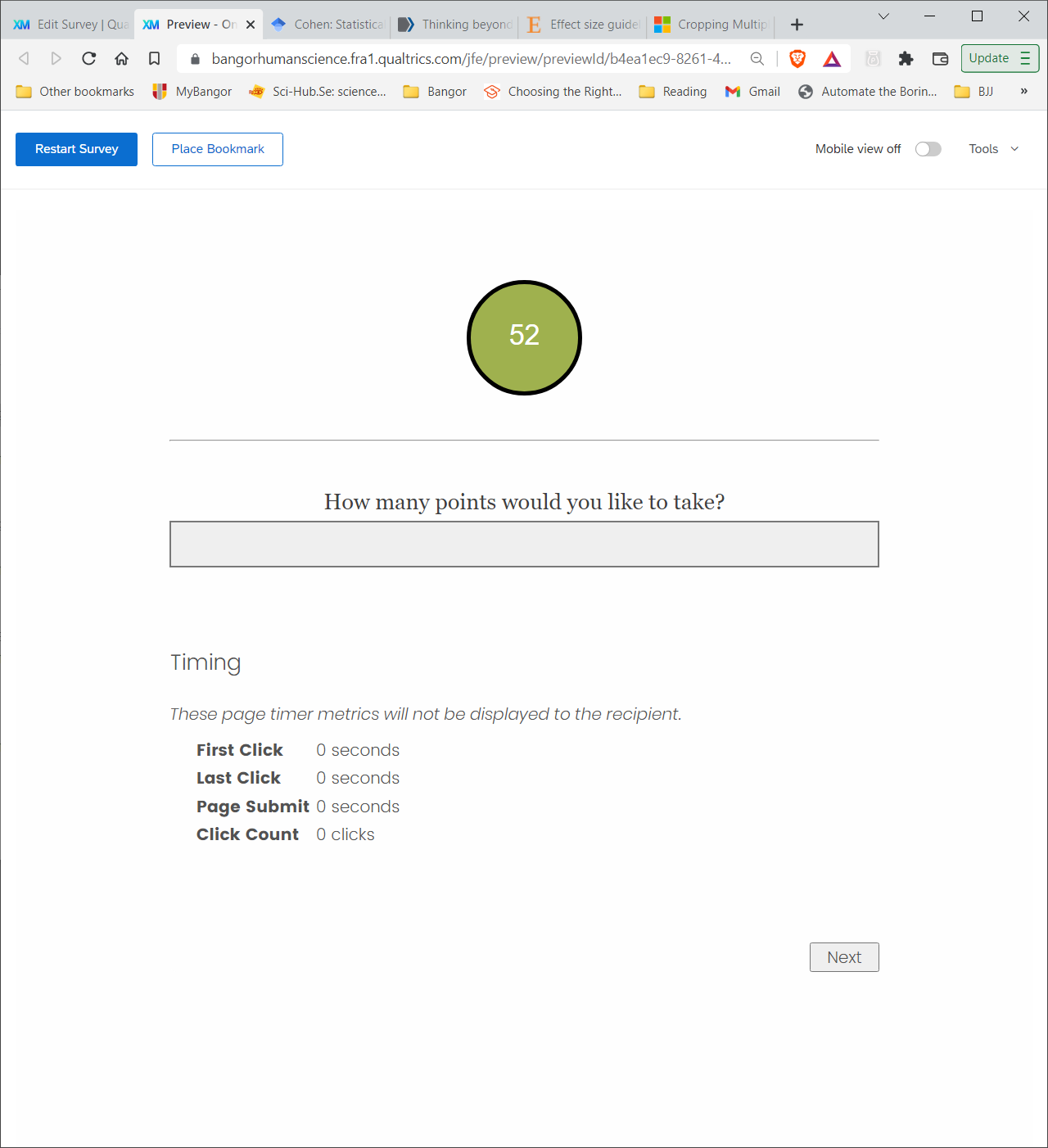


Perhaps, the participant might then choose to harvest the full amount of rewards contained in the pool.


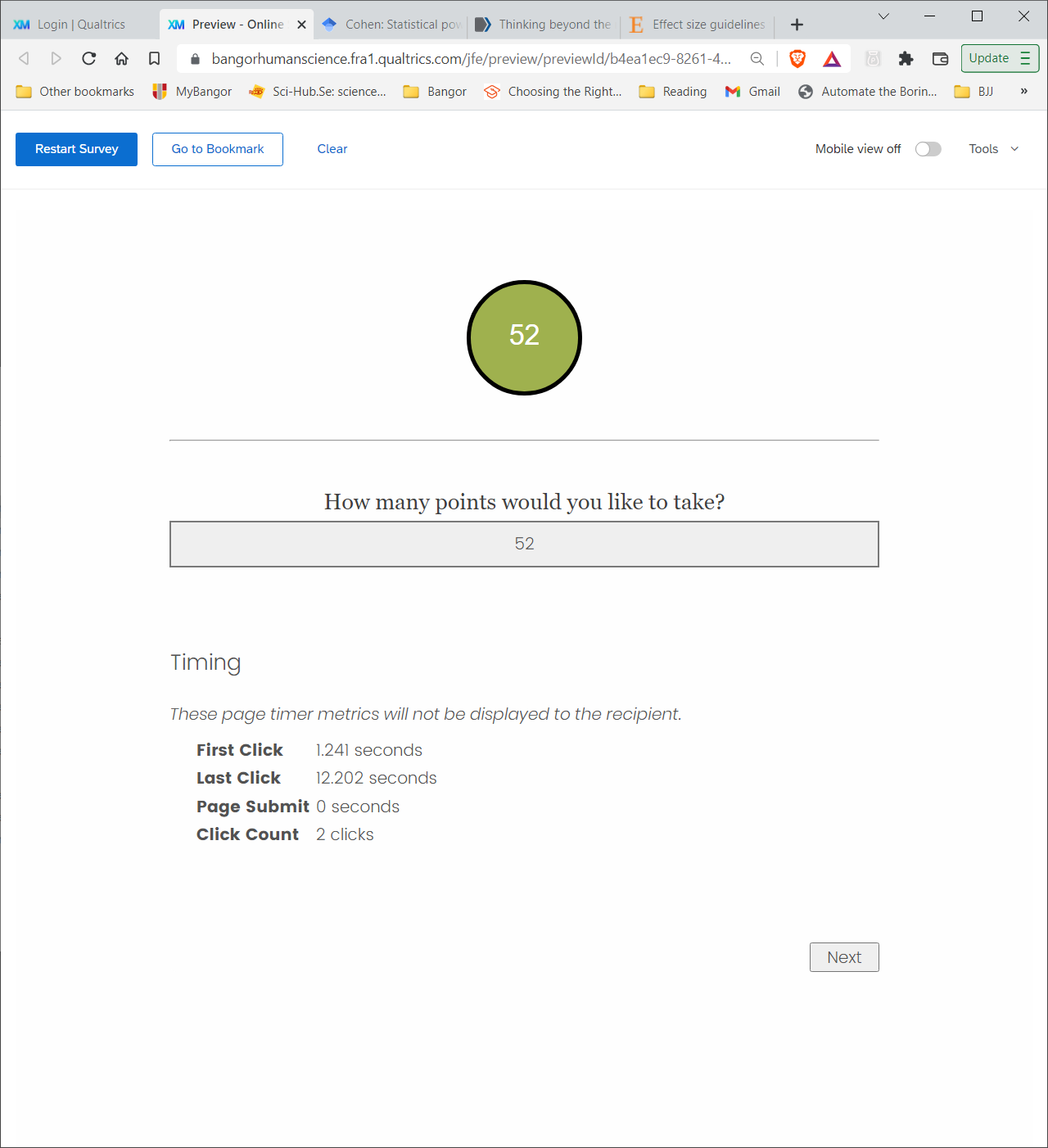


The resource would then be depleted to zero and would replenish no more.
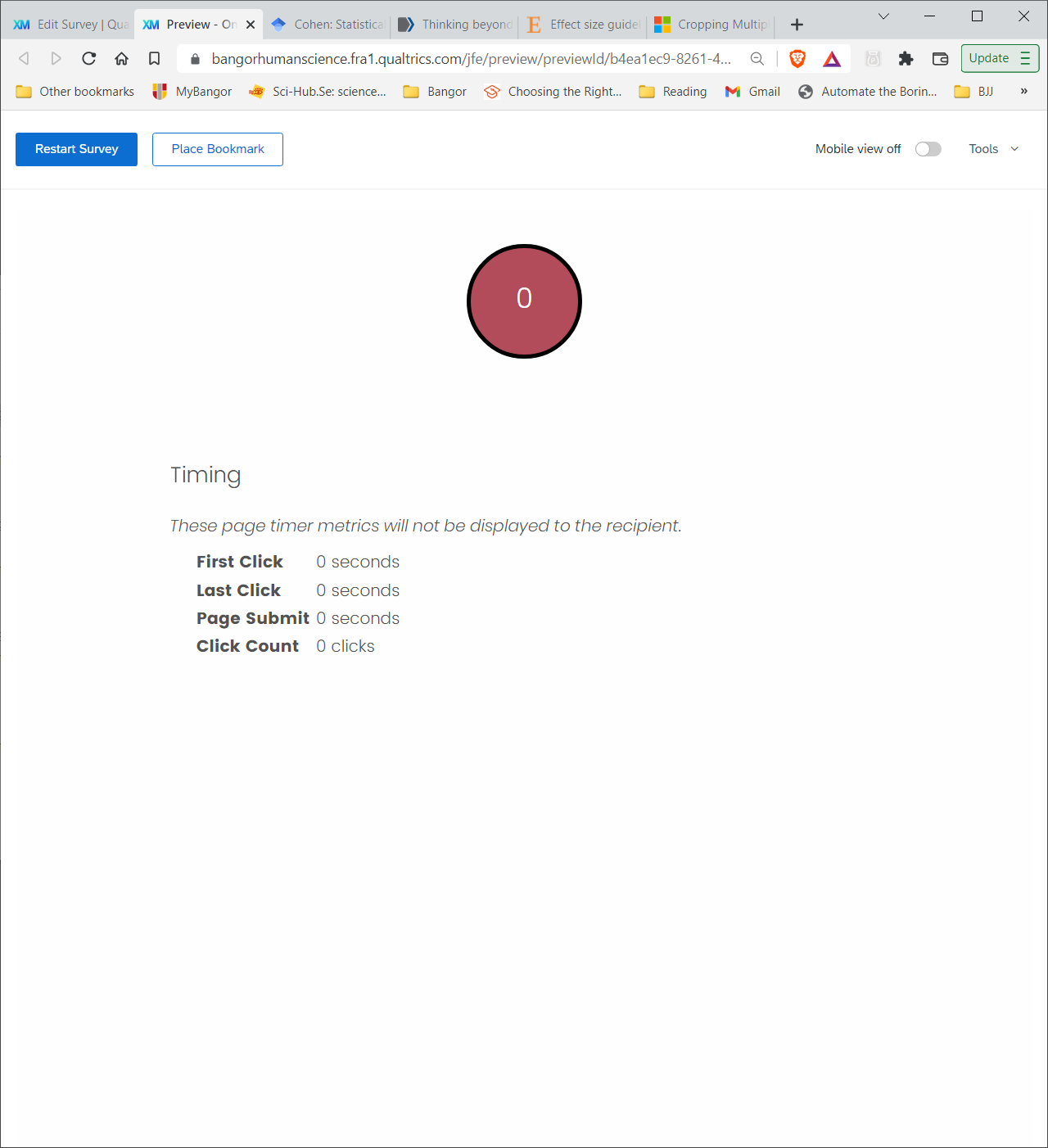


Once the game finished, participants saw this page, with a summary of how they played and how much money they earned.


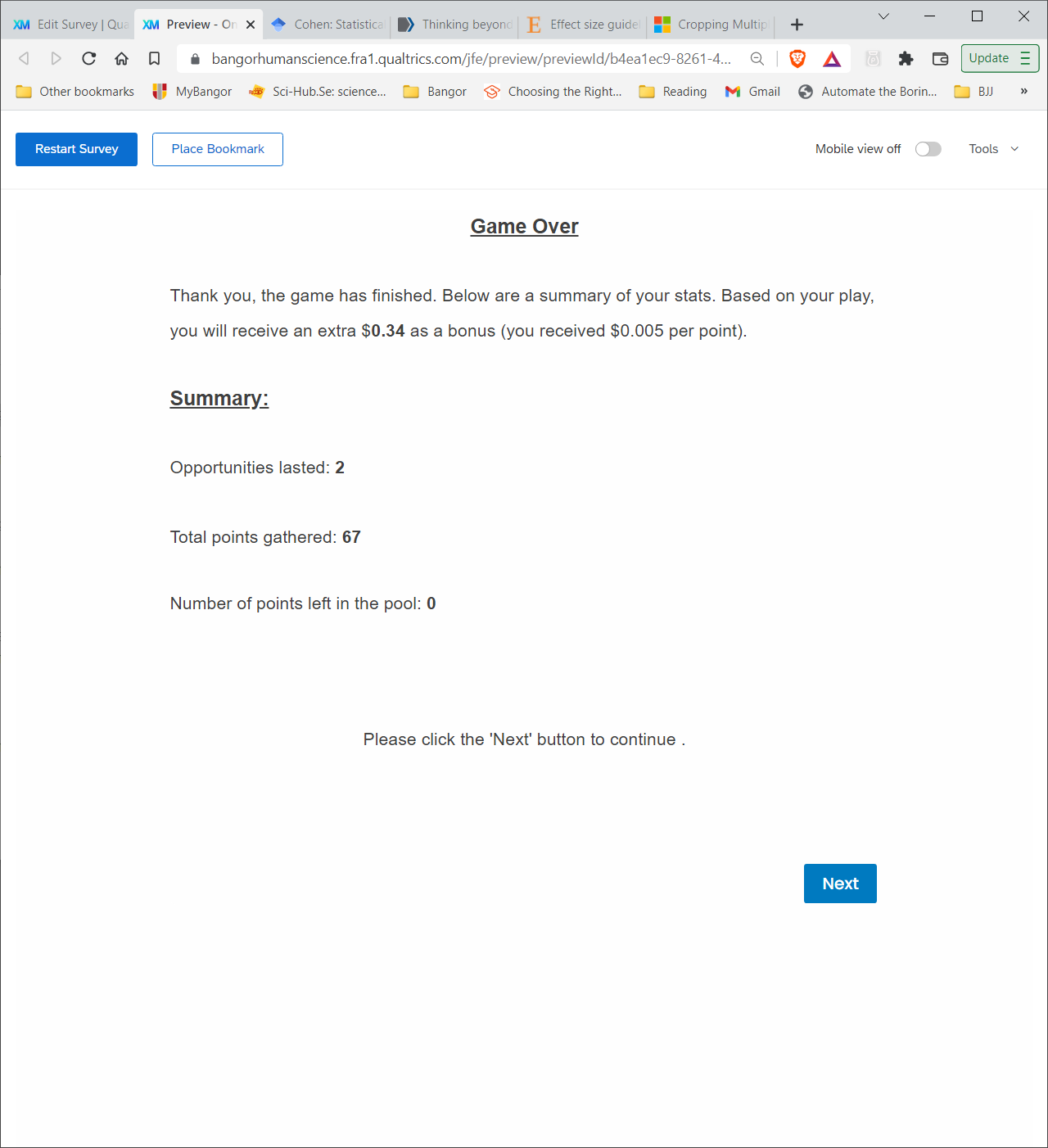


As the participant in our example finished the game before round 50, they would have to wait for a time penalty before they could continue.


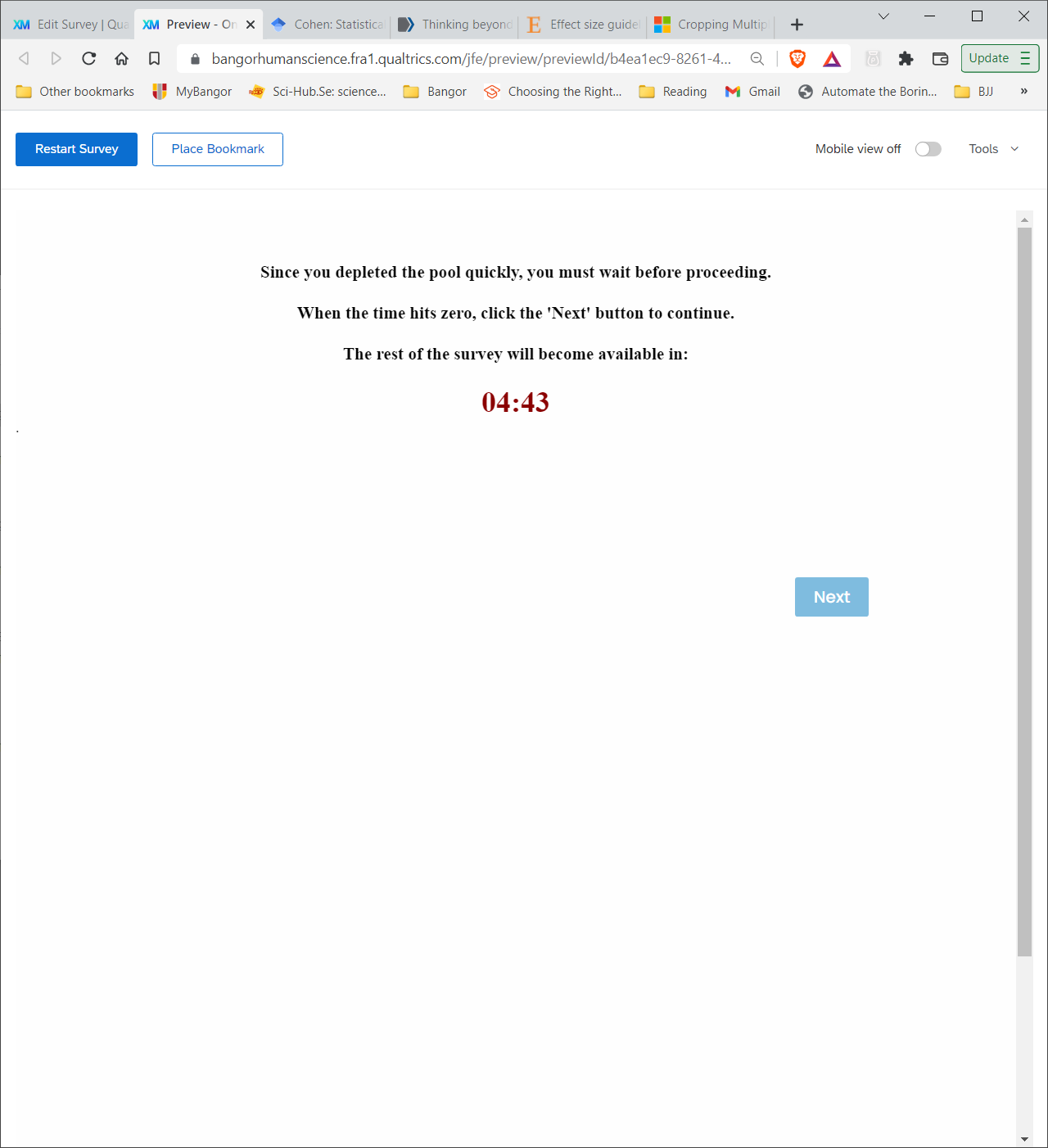


**Multi-partner game:**

In Experiment 2, participants also played a multi-player version of the game. However, this is beyond the scope of this paper, and so we do not include further screenshots of this game here.


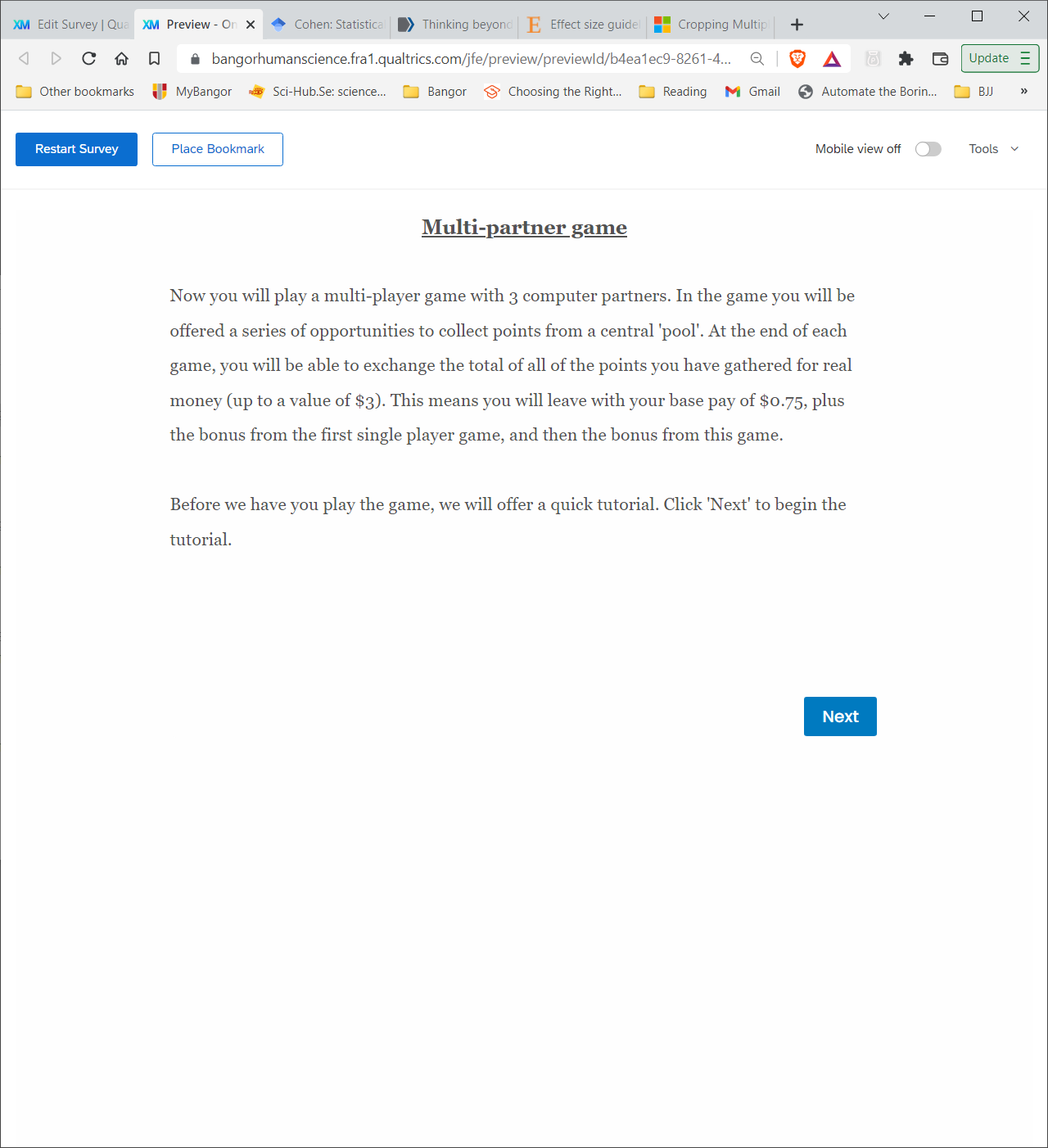


**Questionnaires:**

After finishing the multi-partner game, participants were asked to complete two short questionnaires: the ED_50_ task and the Alcohol Use Disorders Identification Test.
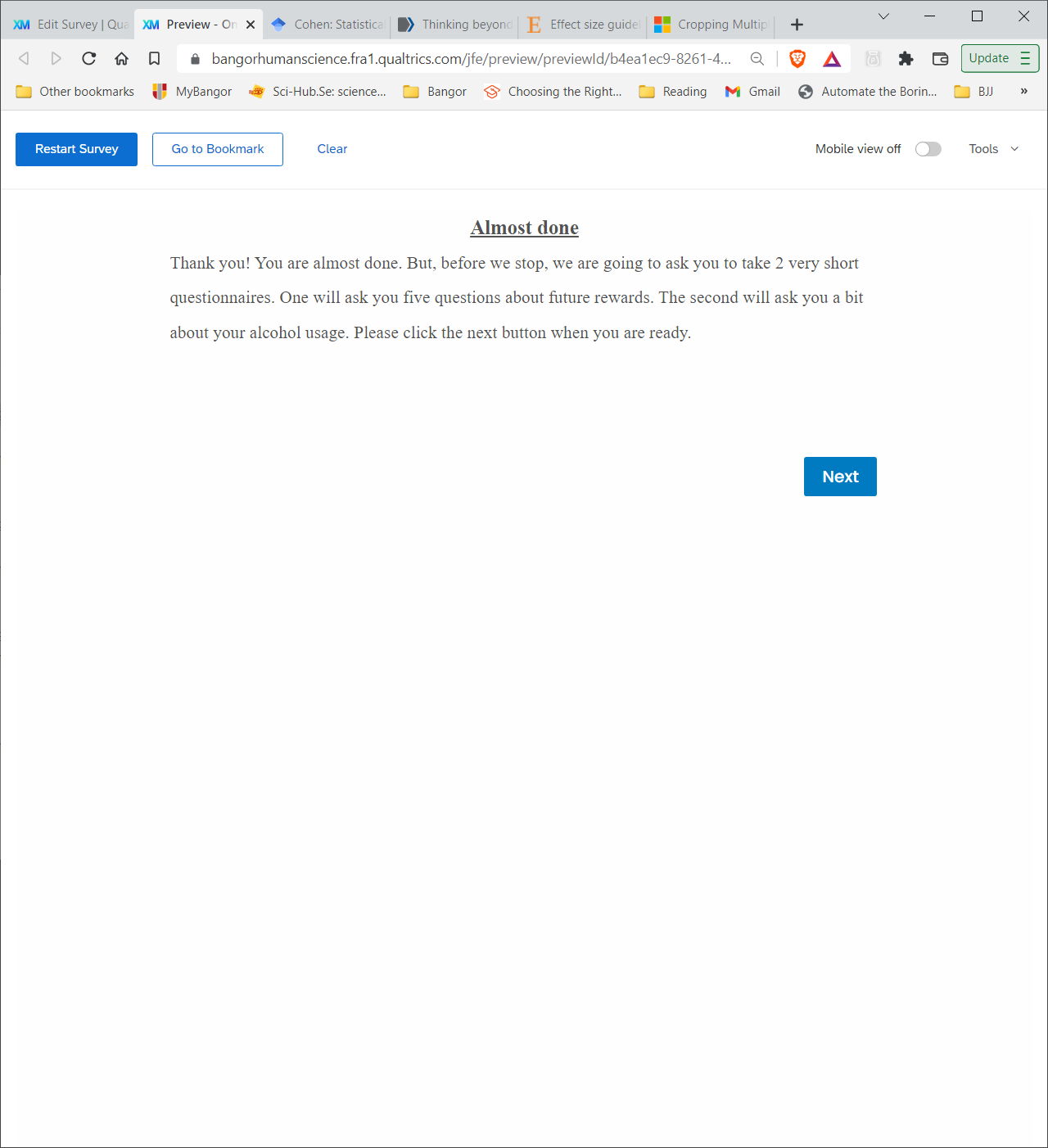


**ED_50_ task:**

In the ED_50_ task (Koffarnus & Bickel, 2014), participants are presented with five consecutive choices, where they indicate whether they would prefer $500 now or $1000 after some delay. If the participant chooses the immediate sum of $500 in one round, the delay for the $1000 sum is decreased for the next choice. Likewise, if the participant chooses the delayed $1000 sum in one round, the delay is increased for the following round. By the fifth round, through adjusting the delay for the later reward according to a participant’s choices, we find the point at which the participant subjectively devalues the later sum by 50%. The ED_50_ score is determined by the participant’s choice in the fifth round of the task. Of note, for each question, we randomized the order of the $500 and $1000 choice.

Below, we give an example play-through of the first two choices a participant might make in the task.


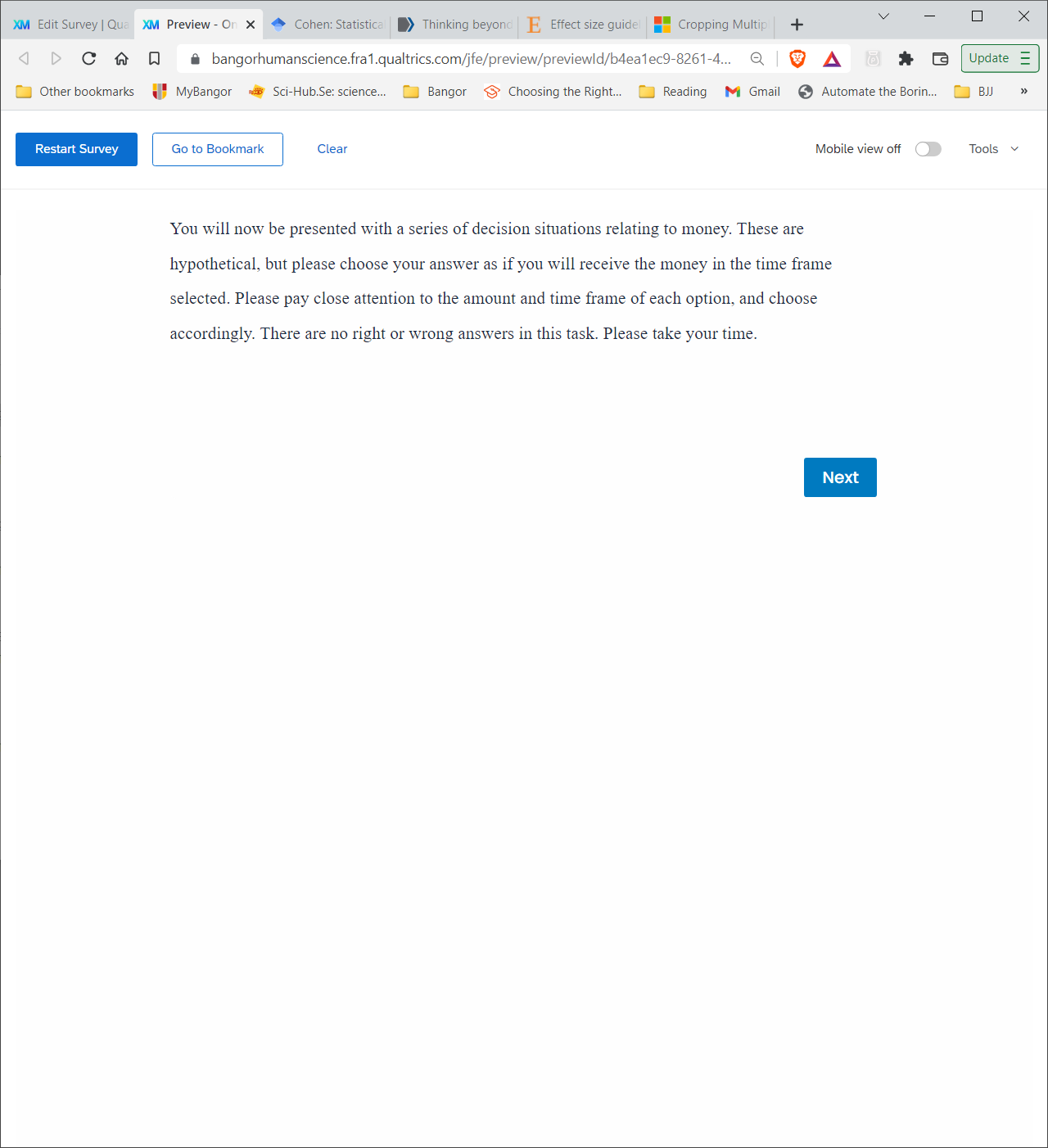


All participants started the task with the following hypothetical decision:


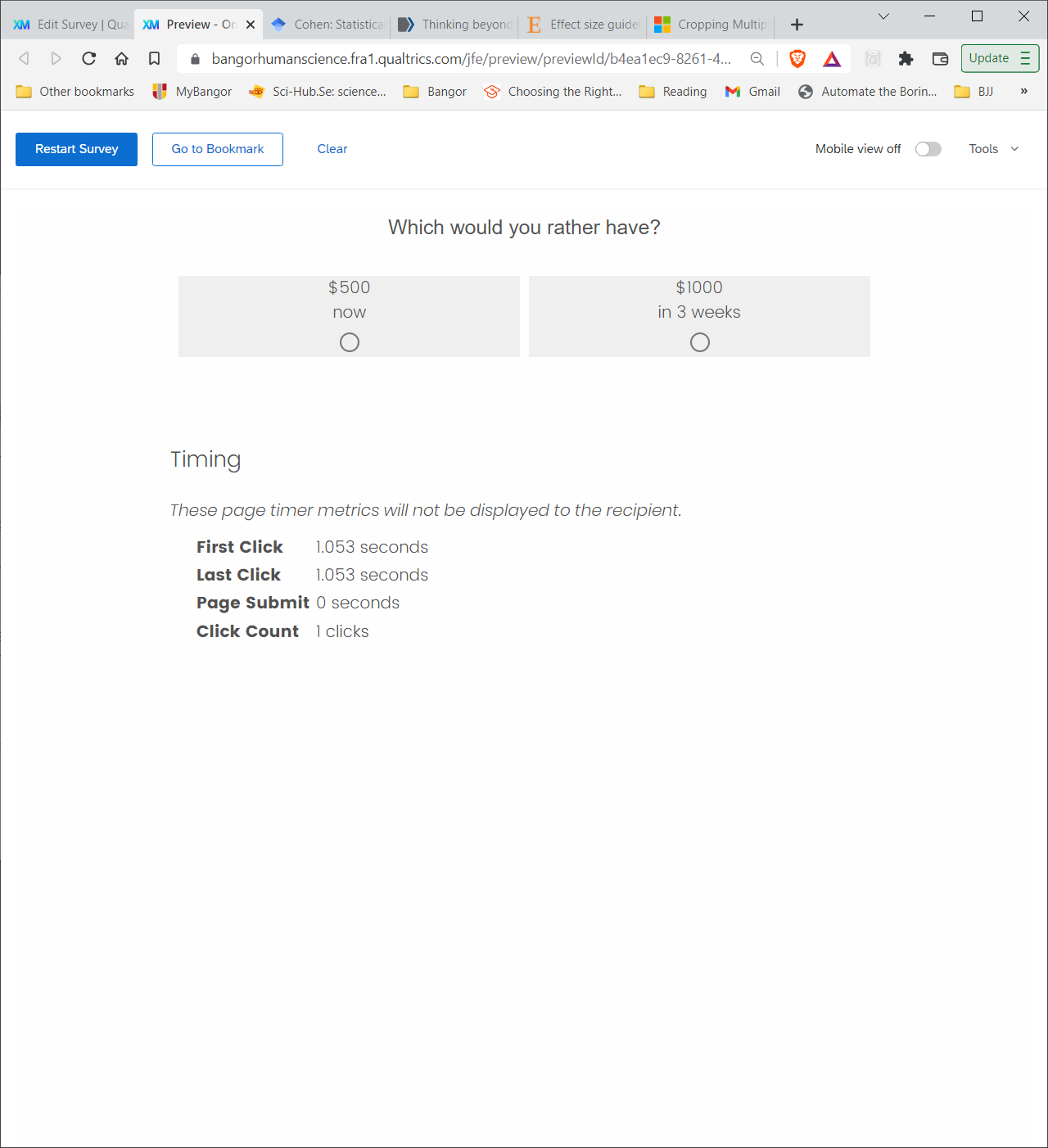


A participant may, for example, prefer the delayed sum here.


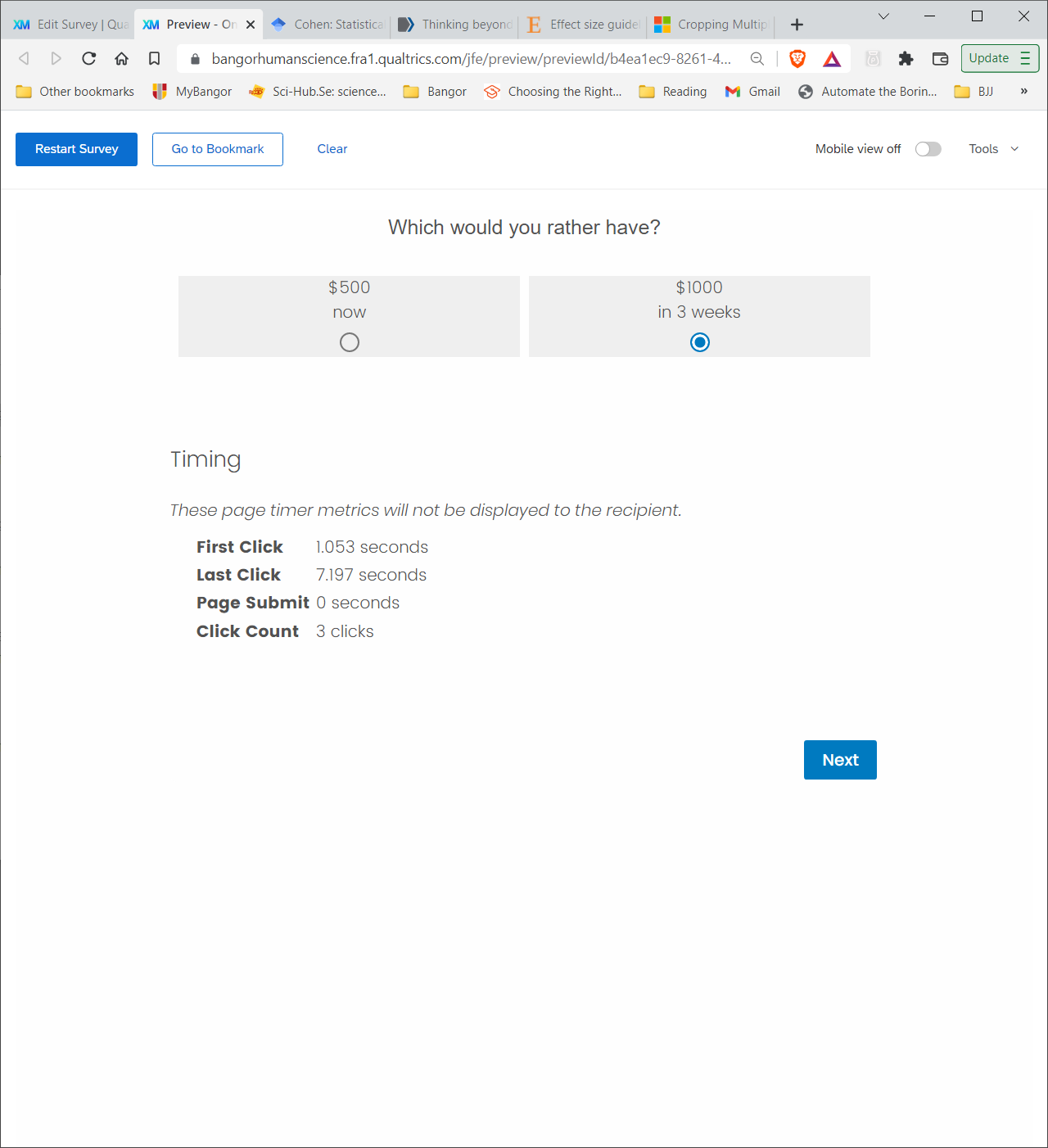


The delay for the $1000 sum is then increased for the next decision.


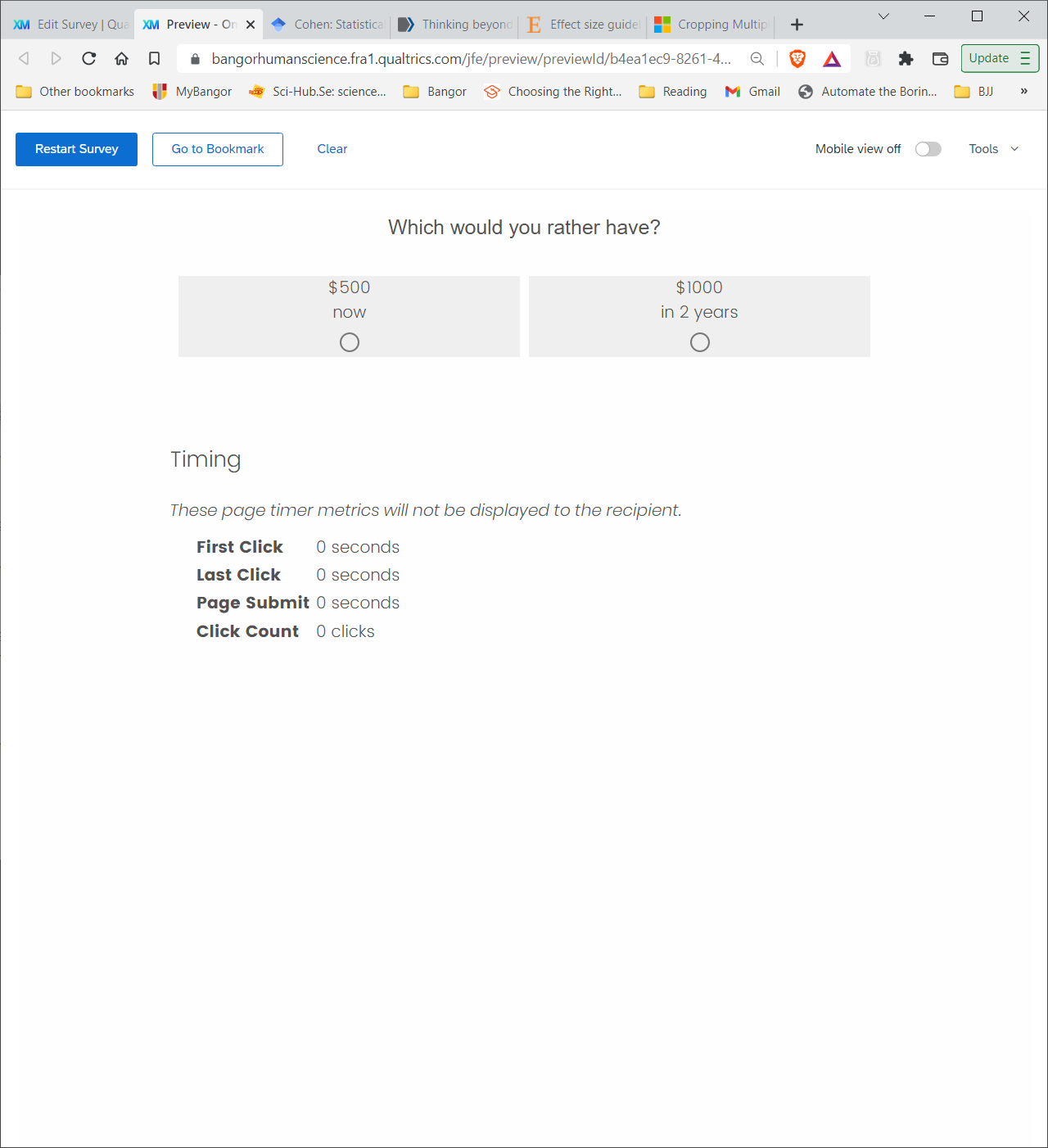


The participant may then prefer the immediate sum in this decision.


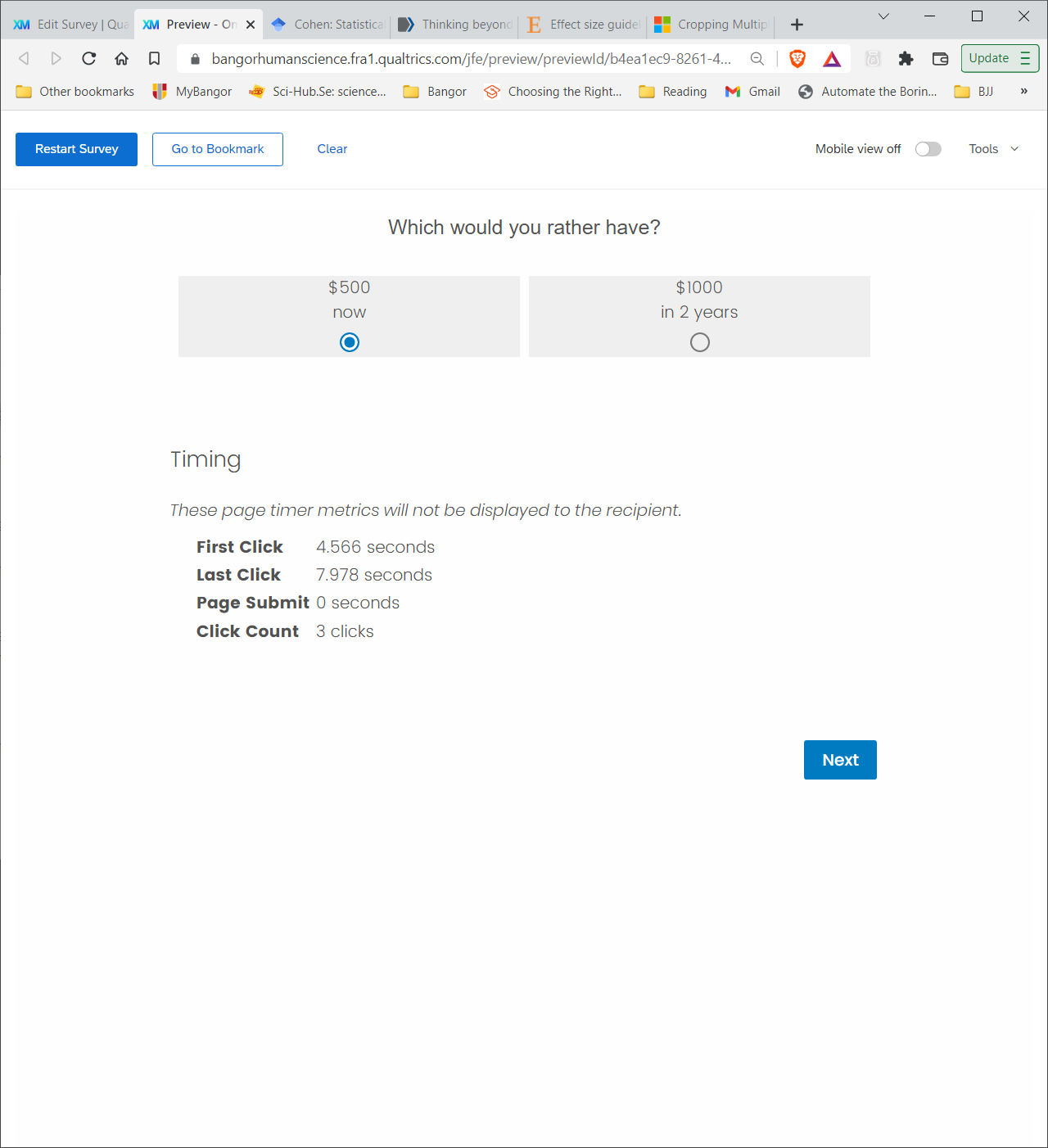


The delay for the $1000 sum is then reduced for the next round.


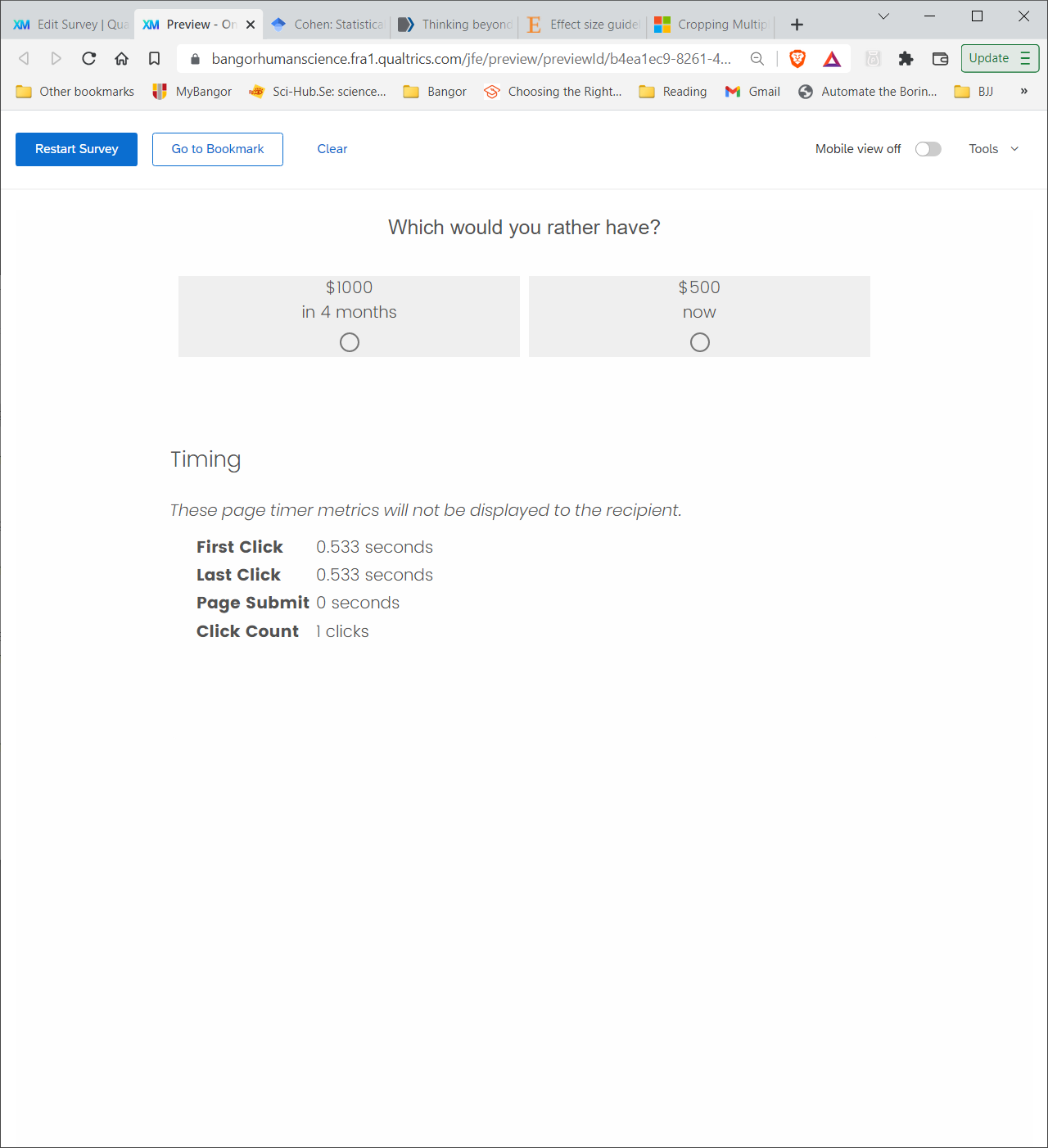


**Alcohol Use Disorders Identification Test (AUDIT):**

The AUDIT is a 10-item questionnaire to assess harmful alcohol use (Saunders et al., 1993). All items are scored from 0 – 4. The final two items are scored 0 for ‘No,’ 2 for ‘Yes, but not in the past year,’ and 4 for ‘Yes, during the past year.’


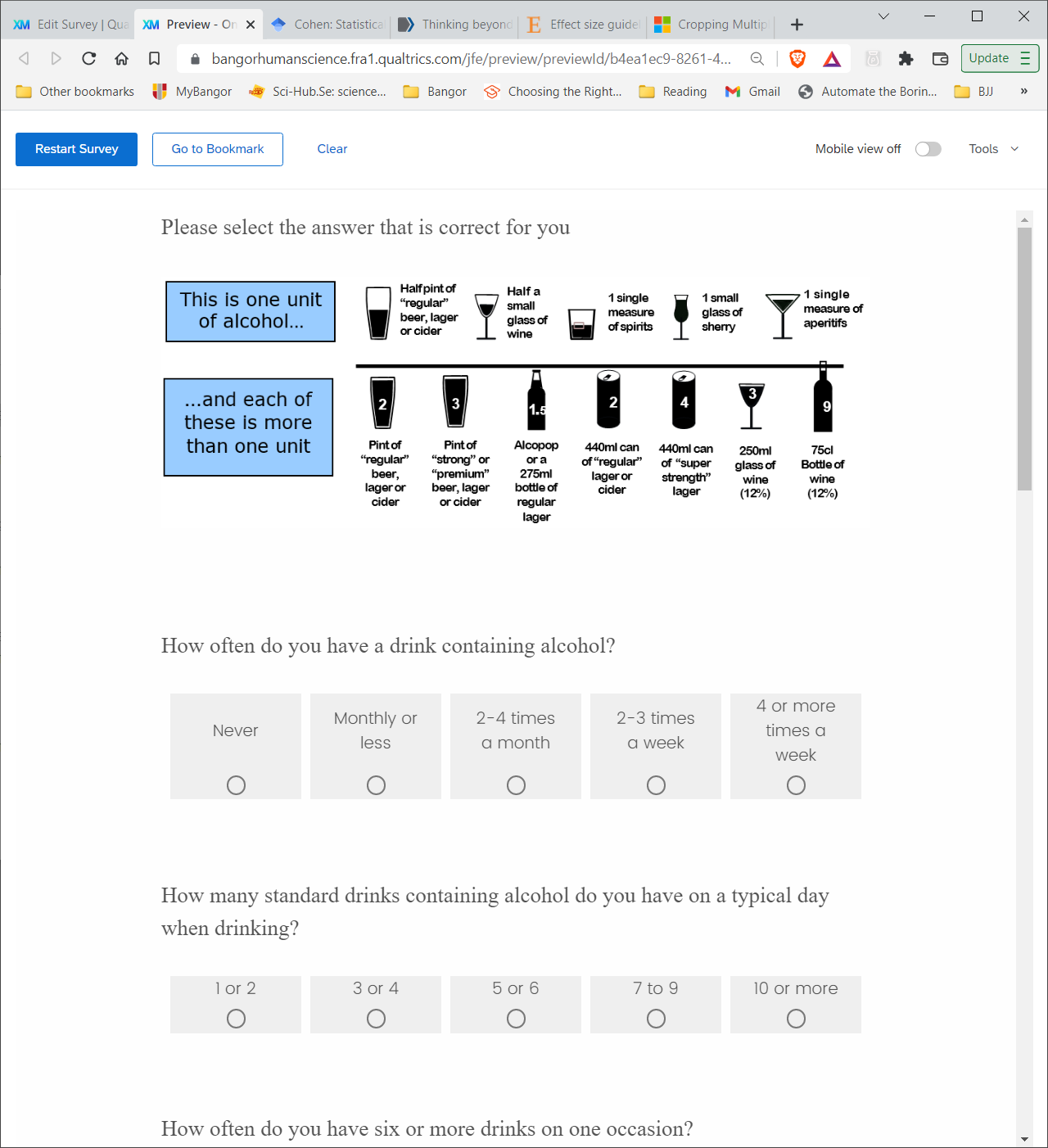


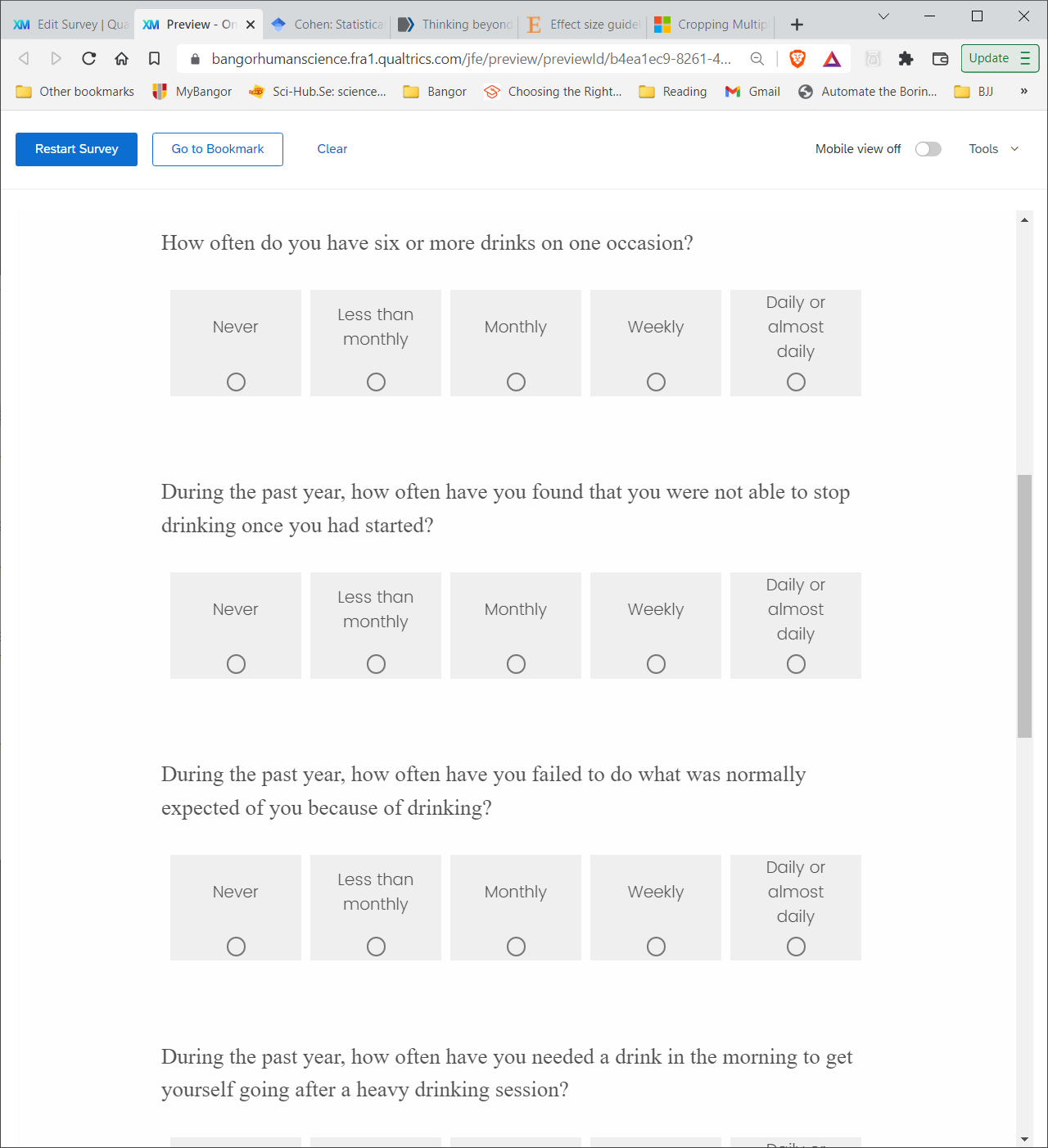

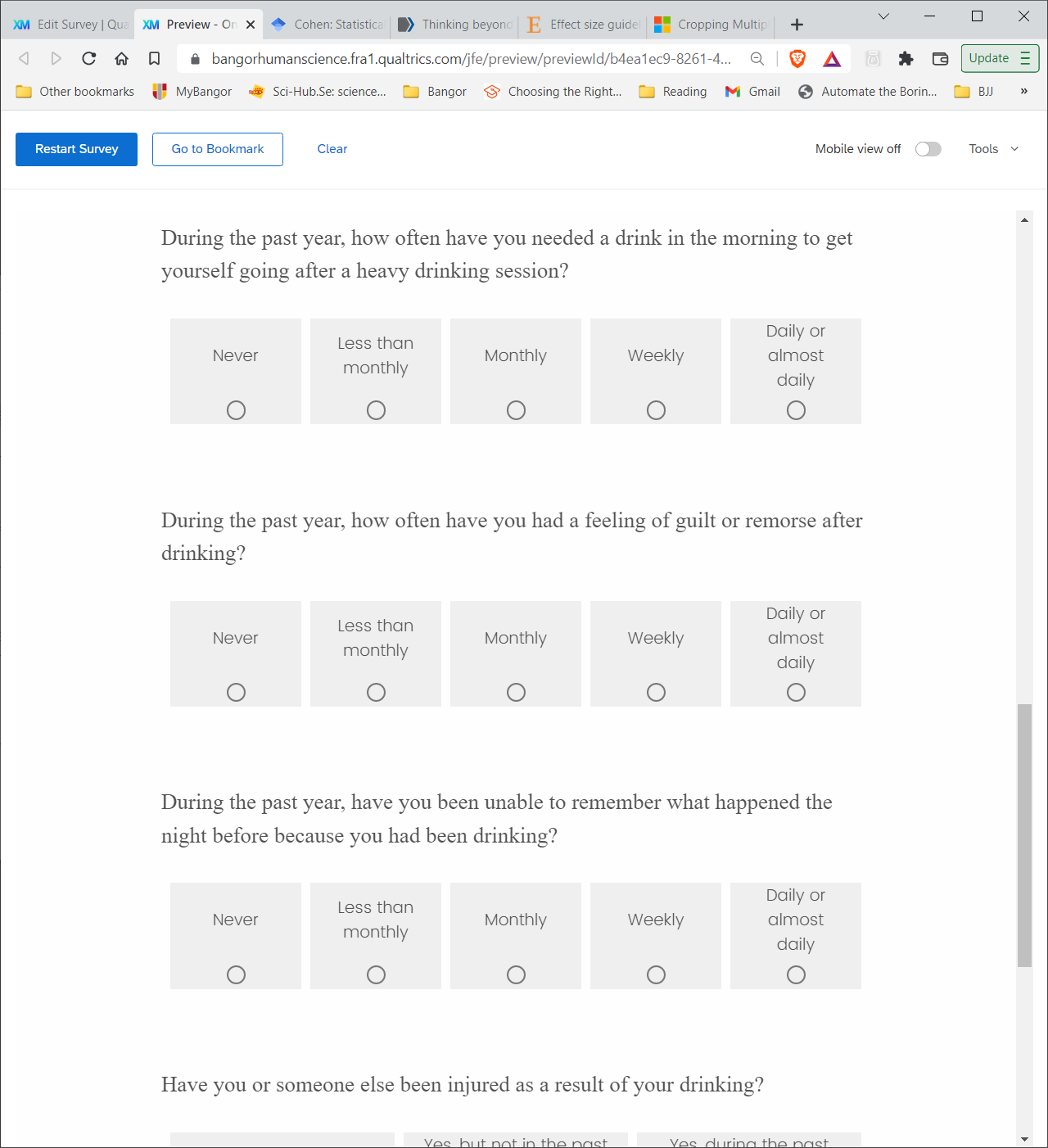

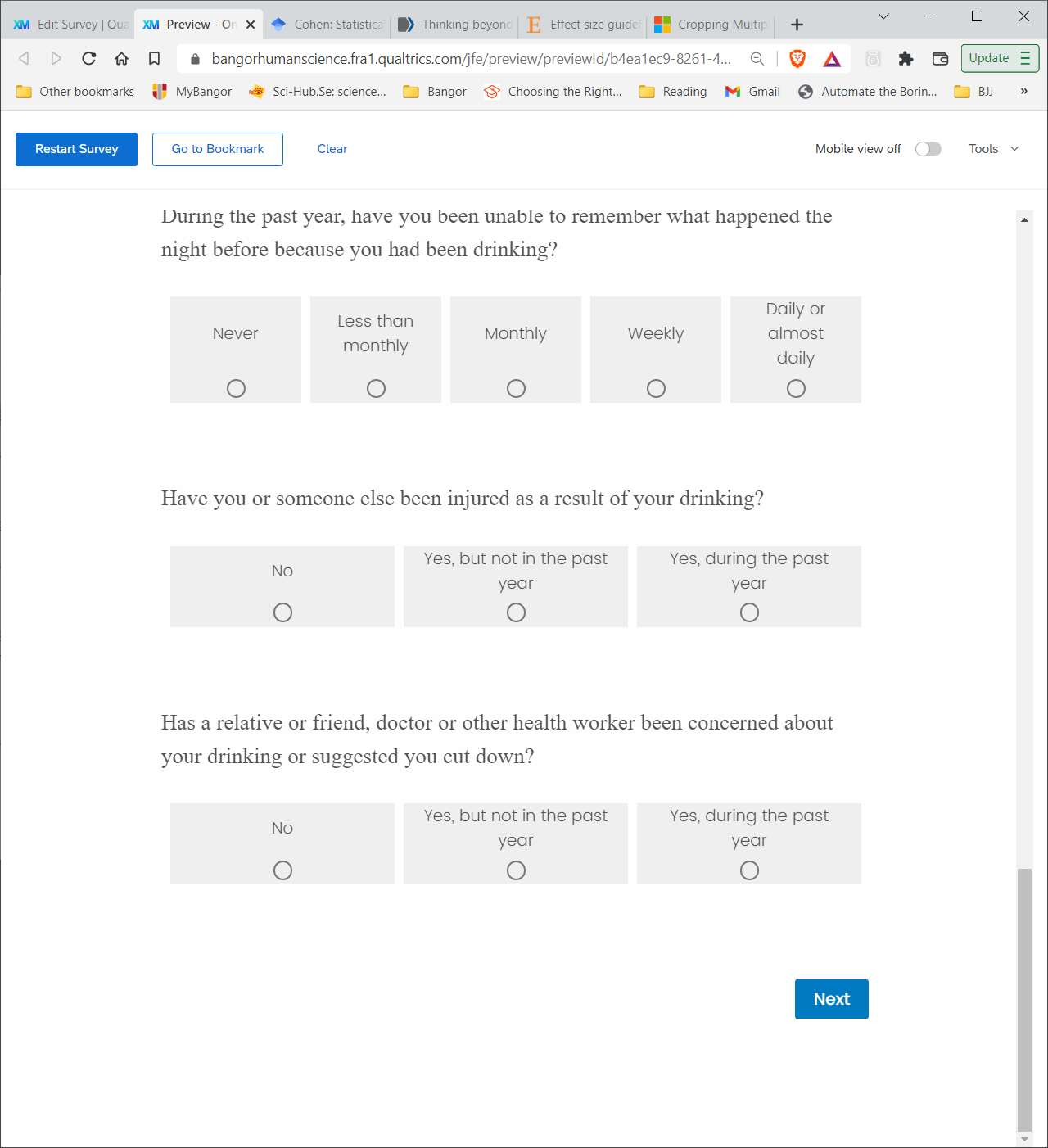


**End of survey page:**

Finally, the participants were thanked for taking part and shown a summary of their game stats and money earned.


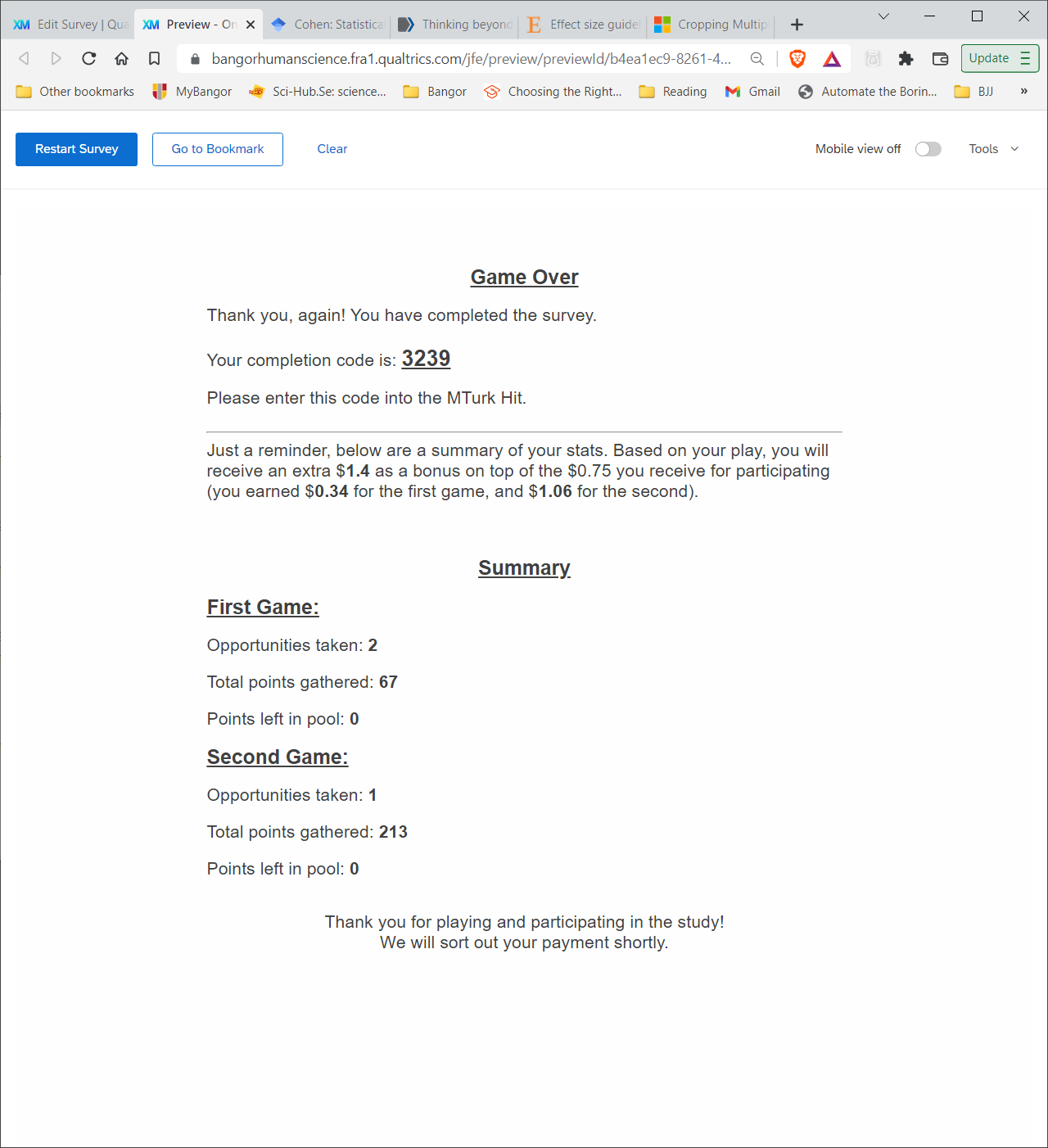


**Supplemental Material B – Calculating the optimal policy of the resource management game**

In the resource management game, the participants’ objective was to harvest as many points as possible over the duration of the game. As participants in our experiment did not know the duration of the game, they had to maximise this cumulative reward over an indefinite time period. Thus, a sensible strategy would be to take modest harvests from the resource, allowing the resource to replenish to its maximum level each round and maximising the rewards available in future rounds. However, the stochastic nature of the resource’s replenishment rate added an extra layer of complexity to participants’ harvest decisions, increasing uncertainty about potential future rewards in the game. The optimal strategy for the game is, therefore, not immediately obvious.

To determine the optimal strategy, we modelled decisions in the game using a Markov Decision Process (MDP; Bellman, 1957a; Sutton & Barto, 1998). A MDP is a mathematical framework used to model Markov sequential decision-making problems with stochastic elements, such as the resource management game. A MDP represents a game as a set of states, actions, rewards, and transition probabilities. Using this framework for the resource management game, the states correspond to the possible levels of the resource, the actions correspond to the possible harvest amounts, the rewards correspond to the points gained from each harvest, and the transition probabilities correspond to the probabilities of the resource replenishing to each possible resource level, based on its current level and the replenishment rate.

More specifically, in the case of the resource management game, there are 61 possible states, since the resource can hold a value of any integer in the range: [0, 60]. If the current value of the resource is defined as *v*, then, at any given round, the action set available to a participant is defined as an integer in the range: [0, *v*]. The participant can harvest any value of the resource between zero and the current value of the resource. The reward received for each action is defined as the amount harvested by that action. Finally, the transition probability represents the likelihood of transitioning to a specific state (*s’*) given that a participant was in a state (*s*) and took the action to harvest a certain amount (*a*): *P*(*s’* | *s , a*). For example, in one round of play, if the resource contained 60 points (its current state: *s*), and the participant harvested 20 points (the action: *a*), the participant would receive 20 points (the reward), and the resource would be depleted to 40 points. Before the next round, the resource would replenish by some amount and transition to a new state. The probability of transitioning to each of the 61 possible states is given by the transition probabilities. In this example, the probability of transitioning from 40 points to 40 points or below would be zero, as the resource in our game always replenished by at least 1 point unless it had been depleted to zero. As the replenishment rate was taken from a Gaussian distribution with mean = 15%, the most probable new state would be 46 points (40 x 1.15 = 46).

By considering all possible states, actions, and their corresponding transition probabilities, we can use dynamic programming to determine the optimal policy, which specifies the action to take in each state that maximizes cumulative rewards (Bellman, 1957b). We can do this for the resource management game whether we model the game as a finite horizon problem, with a definitive end point, or as an infinite horizon problem, with no end point. As participants in our experiment did not know the duration of the game, to accumulate a large sum of points over the 70 rounds of the game they had to use a strategy which sustained the resource indefinitely. Therefore, we first chose to model the game as an infinite horizon problem and used value iteration to find a policy optimised to maximise rewards for a duration much longer than the actual duration of our game.

***Value iteration***

When the resource management game is modelled as an infinite-horizon problem, the optimal policy can be determined using value iteration. The value iteration algorithm uses the Bellman equation to iteratively calculate and update the value of each state, considering both immediate rewards and the expected cumulative rewards of future states (Puterman, 1994). An important parameter in this calculation is the discount rate, γ, which determines the extent to which the policy is optimised for immediate versus long-term rewards. γ can be any value between 0 and 1, and as γ approaches 1, the algorithm discounts future rewards less strongly and therefore generates an optimal policy that is more focused on long-term outcomes (Sutton & Barto, 1998). We used the *mdp_value_iteration* function in the *MDPtoolbox* package in R to find the optimal harvest at each possible resource value in order to maximize the expected cumulative reward in the game. We used a discount factor of γ =.99999 so that the algorithm discounted future rewards less, encouraging it to produce a policy which was optimised for long-term outcomes.

Looking at the optimal policy produced by the value iteration algorithm, long-term cumulative rewards can be maximised by applying an *Aim for 51* strategy. If the resource level is above 51 points, long-term cumulative rewards are maximised by harvesting such that the resource is depleted to 51 points (before it is replenished ready for the following round). If the resource level has a value of 51 points or less, it is best to harvest zero resources and allow the resource to grow until it contains more than 51 points.

This strategy does make intuitive sense. As the game was played over an unknown-horizon, the most important priority was to sustain the resource close to its maximum level to maximise the expected future rewards. However, as long as this was achieved, the next most important priority was to take as many points as possible each round. Given that the resource replenished as a percentage of its current value (each round the replenishment rate was taken from a Gaussian distribution with a mean of 15% and a standard deviation of 3%), the larger the resource the more rewards would replenish (e.g. a resource of 20 would replenish 3 rewards on average, whilst a resource of 40 would replenish 6 rewards on average). However, since the resource could not grow above 60 points, it would be sub-optimal to harvest such that resource replenishment would overshoot the 60-point ceiling. For example, if the resource was at 60, harvesting 1 would only garner 1 reward and the resource would replenish to 60 with 100% likelihood. If one harvested 2, then they would receive 2 rewards, and the resource would replenish to 60, 99.994% of the time. Thus, if the resource has a value of 60, harvesting 2 is better than harvesting 1, since the participant will receive twice the reward and likely end up in the same state for the following round. In this resource management game, aiming for 51 strikes the perfect balance between harvesting as many points as possible while still allowing the resource to replenish close to its maximum level each round.

R code for creating and running the MDP can be found at: <https://osf.io/8b7av/?view_only=075259b34db4412cbf859562cf384eed>

***Backward induction***

Although the duration of our resource management game was unknown to participants, the game did have a definitive duration of 70 rounds (as long as the resource wasn’t depleted to zero before then). Therefore, it could be appropriate to treat the game as a finite horizon problem. This method determines the optimal policy for a player that knows the duration of the game and would therefore produce an optimal policy that achieves the upper limit of performance in the game.

We used backward induction (Puterman, 1994) to find this optimum policy. The backward induction algorithm starts at the final round of the game and finds the optimal action to take for each possible state at this last round. The algorithm then works backwards from there and considers the second-to-last round, finding the optimal action for each state given the previously determined optimal action for the last round given the transition probabilities. It continues this process of finding the optimal action for each round and state until it reaches the first round of the game, and the optimal action for each round has been determined.

Using the *mdp_finite_horizon* function in the *MDPtoolbox* package in R, we found that the optimal policy generated using backward induction was identical to that generated through value iteration for all rounds but round 70 – the final round of the game. From round 1 to 69, the strategy which maximised cumulative rewards was *Aim for 51*. However, the best action in round 70 would be to take all remaining points in the resource. This strategy also makes sense intuitively. In the final round, the game is going to end anyway, so the best way to maximise rewards is to take all remaining points. However, up to this point, the optimal policy would be to harvest as many points as possible while allowing the resource to replenish close to its maximum level each round. By doing this, the maximum possible number of points would be available for harvest in the final round.

It is noteworthy that, aside from the final round, the optimal policies generated by both the value iteration and backward induction algorithms were identical. It demonstrates that, unless the player knew that the current round of the game was the final round, the optimal strategy would be to harvest such that the resource was depleted to 51 points before it replenished for the next round. Participants in our experiment had no information about the duration of the game and, therefore, the most successful participants should have treated the game as an infinite horizon problem, where each round they took as many points as possible while still allowing the resource to replenish close to its maximum level.

R code for running the finite horizon MDP can be found at: <https://osf.io/8b7av/?view_only=075259b34db4412cbf859562cf384eed>

**Supplemental Material C – Evaluating parametric assumptions**

To analyse the effects of health risk factors on resource management outcomes, we wanted to evaluate whether each health risk factor predicted either a.) rounds lasted, or b.) rewards gathered. To do this, we considered running linear regressions. However, prior to this, we needed to evaluate whether our data met the assumptions of linear regression. Linear regressions assume that the residuals of a model are a.) normally distributed and b.) do not exhibit heteroskedasticity. For each potential regression, we ran a Kolmogorov-Smirnov test to see if the residuals of the model were normally distributed. Further, we ran a Breusch-Pagan test to evaluate whether the residuals exhibited heteroskedasticity. Below is the analysis for both Experiment 1 and 2. Several of the regressions did not meet the assumptions, so we decided to analyse the data with non-parametric measures.

***Experiment 1***

*Harmful alcohol use:* A regression with harmful alcohol use as the independent variable and rewards gathered as the dependent variable showed that the residuals were not normally distributed (Kolmogorov-Smirnov: D(200) = 0.61, *p* = 2.2x10^-16^) and demonstrated heteroskedasticity (Breusch-Pagan = 10.89, *p* = .0001). When a regression evaluated whether harmful alcohol use predicted rounds lasted, the residuals were not normally distributed (D(200) = 0.48, *p =* 2.2x10^-16^), though there was little evidence of heteroskedasticity (BP = 2.27, *p =* .132).

*Delay discounting:* When evaluating the effects of delay discounting on rewards gathered, the residuals were not normally distributed (D(400) = 0.66, *p =* 2.2x10^-16^) and showed heteroskedasticity (BP = 13.97, *p =* .0002). The residuals for delay discounting and rounds lasted exhibited non-normality (D(400) = 0.48, *p =* 2.2x10^-16^) and heteroskedasticity (BP *=* 20.37, *p =* 6.39x10^-6^).

*Psychological distress:* When considering the association between psychological distress and rewards gathered, the residuals were not normally distributed (D(200) = 0.65, *p =* 2.2x10^-16^), but there was little evidence of heteroskedasticity (BP = 1.80, *p =* .18). The residuals when evaluating rounds lasted exhibited non-normality (D(200) = 0.49, *p =* 2.2x10^-16^) but no heteroskedasticity (BP = 1.41, *p =* .235).

*Well-being:* The residuals for well-being and rewards gathered were not normally distributed (D(200) = 0.64, *p =* 2.2x10^-16^), but there was little evidence of heteroskedasticity (BP = 0.26, *p =* .612). When evaluating rounds lasted with well-being, the residuals exhibited non-normality (D(200) = 0.48, *p =* 2.2x10^-16^), but not heteroskedasticity (BP = 0.59, *p =* .442).

*Financial Literacy:* The residuals for financial literacy and rewards gathered were not normally distributed (D(200) = 0.65, *p =* 2.2x10^-16^) but did not display heteroskedasticity (BP = 0.15, *p =* .70). When considering rounds lasted and financial literacy, the residuals did not meet the assumption of normality (D(200) = .49, *p =* 2.2x10^-16^) and exhibited heteroskedasticity (BP = 30.12, *p =* 4.06x10^-8^).

***Experiment 2***

*Harmful alcohol use:*

A regression with harmful alcohol use as the independent variable and rewards gathered as the dependent variable showed that the residuals were not normally distributed (Kolmogorov-Smirnov: D(381) = 0.70, *p* = 2.2x10^-16^) and demonstrated heteroskedasticity (BP = 6.87, *p* = .009). When a regression evaluated whether harmful alcohol use predicted rounds lasted, the residuals were also not normally distributed (D(381) = 0.54, *p* = 2.2x10^-16^) and demonstrated heteroskedasticity (BP = 6.46, *p* = .011).

*Delay discounting:*

A regression with delay discounting as the independent variable and rewards gathered as the dependent variable showed that the residuals were not normally distributed (Kolmogorov-Smirnov: D(381) = 0.69, *p* = 2.2x10^-16^), though the test for heteroskedasticity was only marginally significant (BP = 3.58, *p* = .059). When a regression evaluated whether delay discounting predicted rounds lasted, the residuals were also not normally distributed (D(381) = 0.56, *p* = 2.2x10^-16^) and demonstrated heteroskedasticity (BP = 8.21, *p* = .004).

**Supplemental Material D – Scatterplot graphs of the association between sustainable behaviour and individual characteristics**

This section provides additional visualizations of the data. We provide a scatterplot of the association between each individual characteristic (i.e. delay discounting, well-being, etc…) and each measure of sustainable behaviour (i.e. rounds lasted and rewards gathered). On the scatterplot, we also provide the best fit quantile lines. While, in the main text, we measured 19 different quantiles (.05 - .95, in increments of .05), here we only show 10 of the quantile lines (.05 - .95 in increments of .1), since otherwise the graph would be difficult to interpret. In these graphs, the values of each individual characteristic were not normalized to a mean of zero and a standard deviation of one. This was so that values of the measures of the individual characteristics appear in their raw form. However, this means that the beta values seen in the visualizations will not directly match those discussed in the main text.


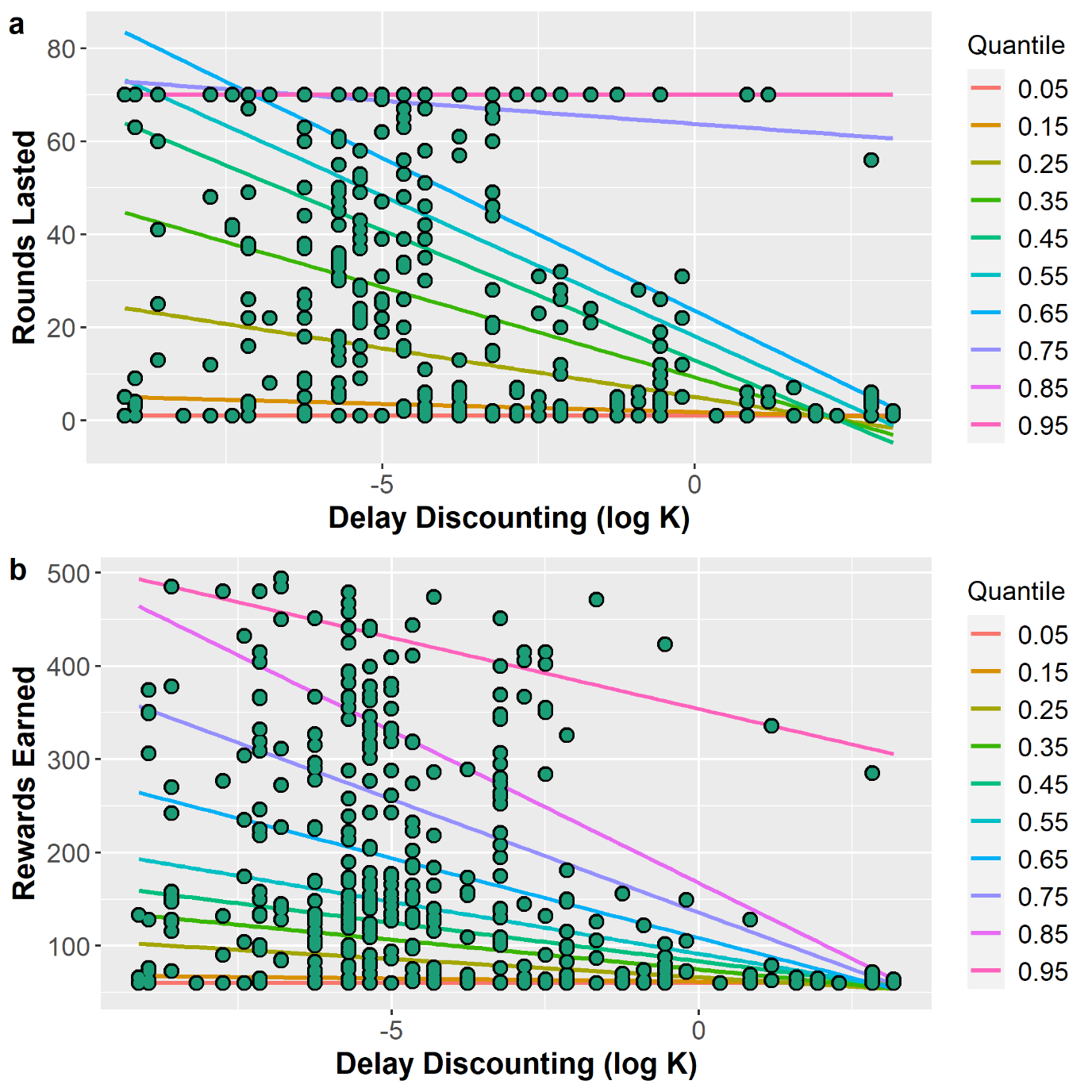


**Figure D.1**. Scatterplot of delay discounting (represented as the log of k) and **a.)** rounds lasted and **b.)** rewards earned. For each graph the best fit quantile regression lines are included for the quantiles 0.05 - 0.95 in increments of 0.1.

***Experiment 1***

*Delay discounting:* Figure D.1(a) shows there is little relationship between rounds lasted and delay discounting for low or high quantiles (e.g. the lines representing the 0.05 and 0.95 quantile have little to no slope). However, quantiles near the median exhibit a negative relationship between rounds lasted and delay discounting. Similarly, Figure D.1(b) shows that there is little association between rewards earned and delay discounting among the worst performers (i.e. the 0.05 and 0.15 quantile). However, there is a negative relationship between rewards earned and delay discounting for most of the other quantiles.


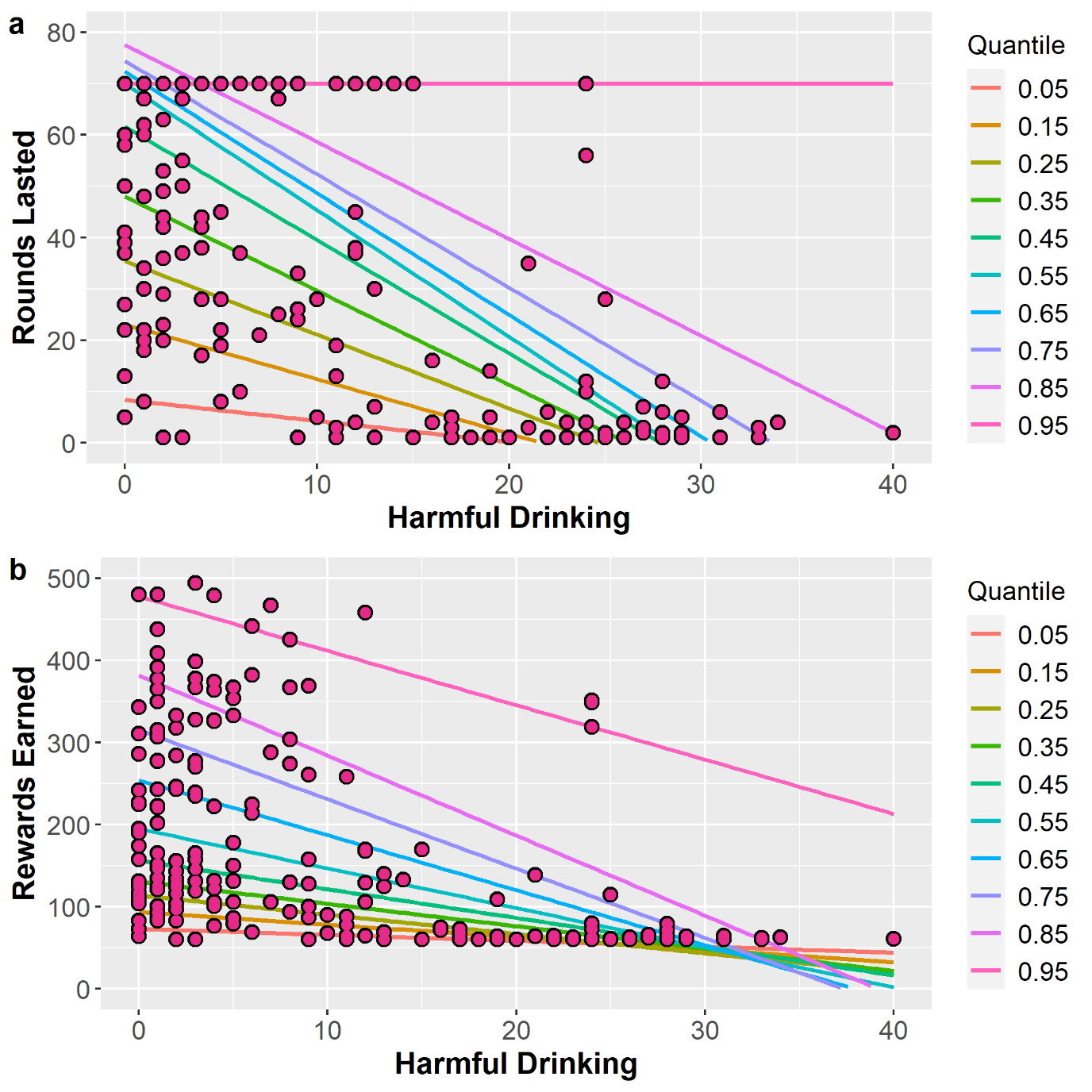


**Figure D.2** Scatterplot of harmful alcohol use (as measured using AUDIT) and **a.)** rounds lasted and **b.)** rewards earned. For each graph the best fit quantile regression lines are included for the quantiles 0.05 - 0.95 in increments of 0.1.

*Harmful alcohol use:* Figure D.2 illustrates the relationship between harmful alcohol use (as measured by AUDIT) and both rounds lasted (Figure D.2(a)) and rewards gathered (Figure D.2(b)). As shown in Figure 3 in the main text, Figure D.2(a) illustrates that there is a strong negative relationship between harmful use and rounds lasted for every quantile but the highest and lowest. Further, Figure D.2(b) shows that the effect size (i.e. slope of the line) between rewards gathered and harmful alcohol use broadly increases as the quantile increases.

**
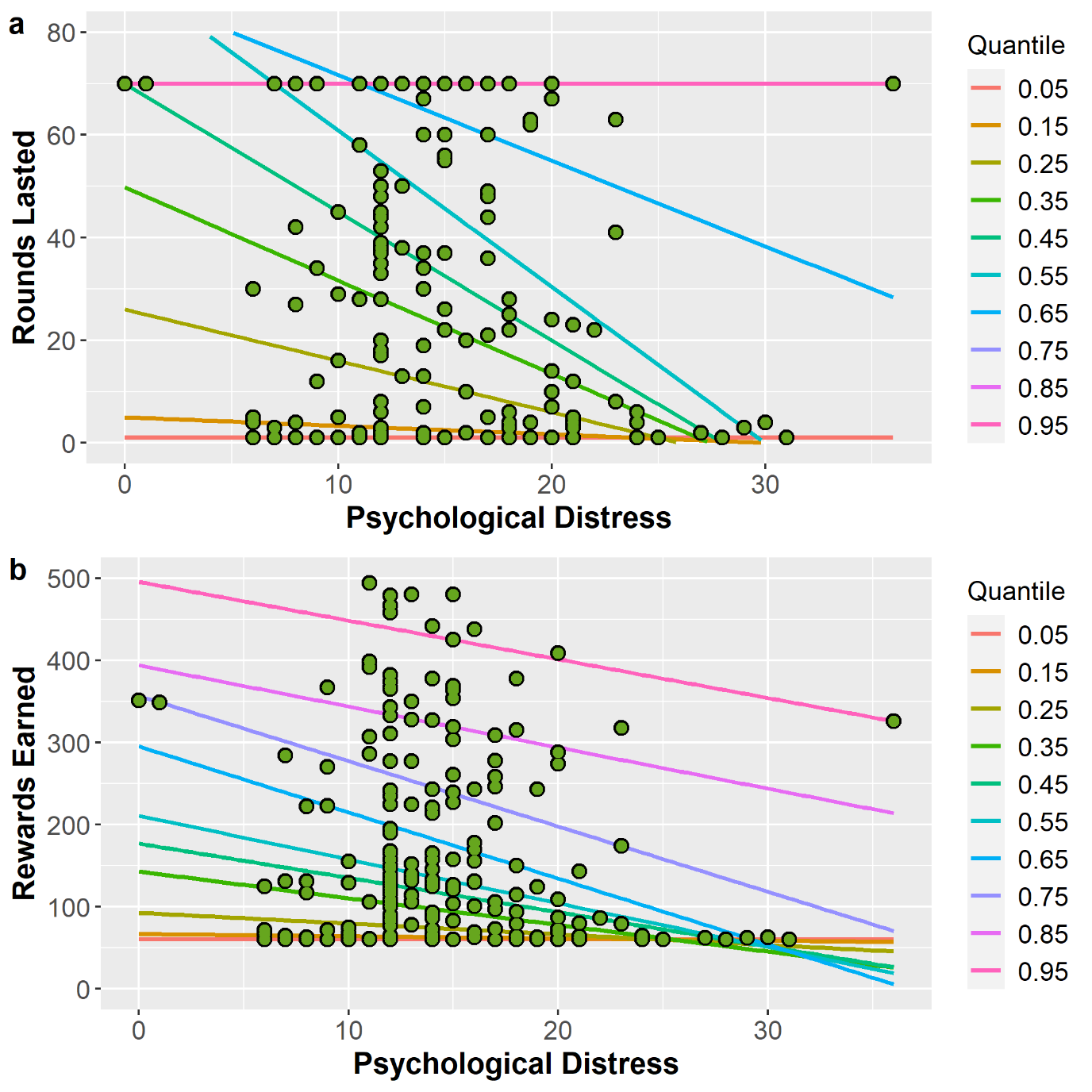
**

**Figure D.3** Scatterplot of psychological distress (as measured using GHQ-12) and a.) rounds lasted and b.) rewards earned. For each graph the best fit quantile regression lines are included for the quantiles 0.05 - 0.95 in increments of 0.1.

*Psychological Distress:* Figure D.3 illustrates the relationship between psychological distress (as measured by GHQ-12) and both rounds lasted (Figure D.3(a)) and rewards gathered (Figure D.3(b)). As shown in Figure 3 in the main text, the strongest negative relationship between psychological distress and both rounds lasted (Figure D.3(a)) and rewards gathered (Figure D.3(b)) occur near the median quantile. However, the effect sizes are weaker compared to delay discounting and harmful alcohol use.

**
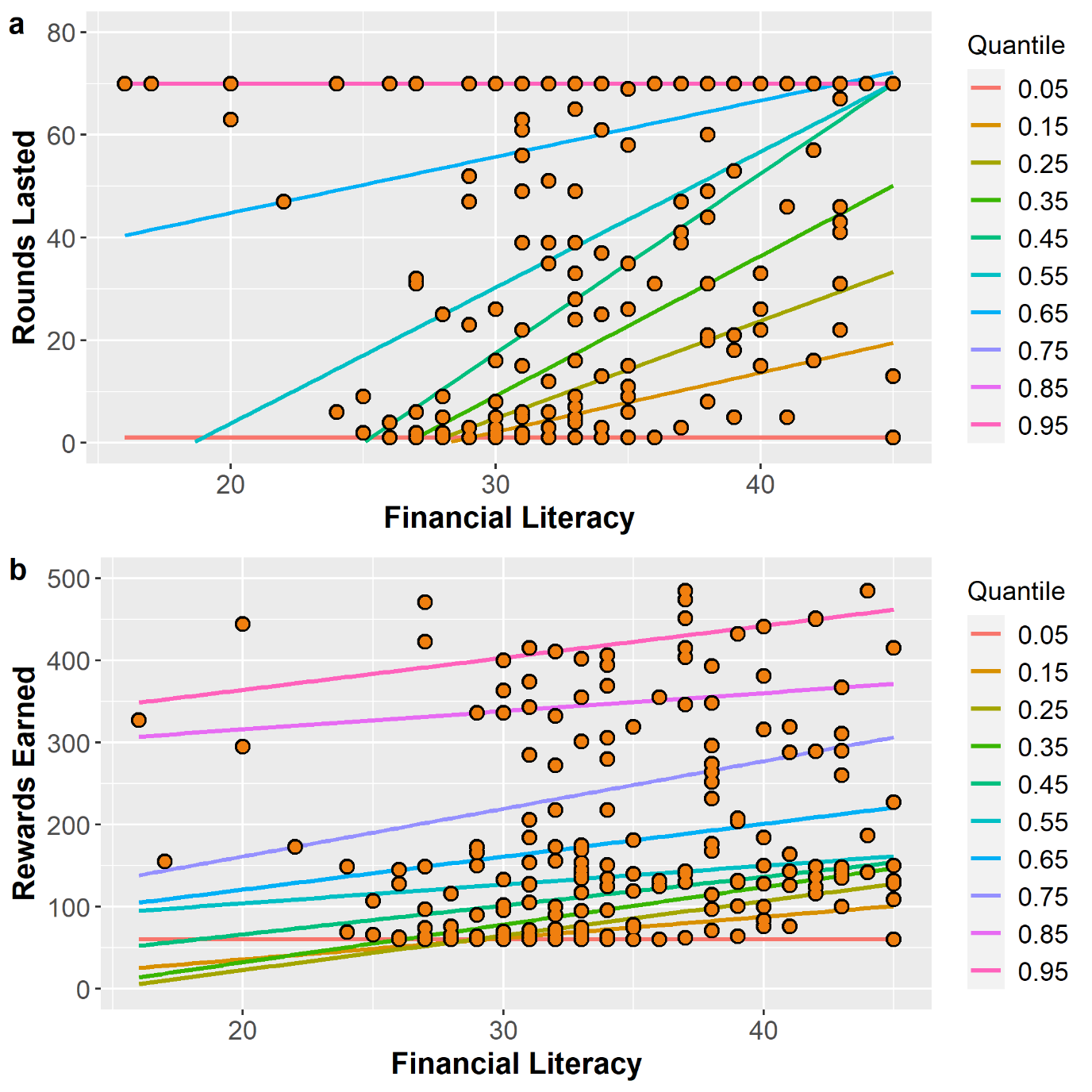
**

**Figure D.4** Scatterplot of financial literacy and **a.)** rounds lasted and **b.)** rewards earned. For each graph the best fit quantile regression lines are included for the quantiles 0.05 - 0.95 in increments of 0.1.

*Financial Literacy:* Figure D.4 illustrates the relationship between financial literacy and both rounds lasted (Figure D.4(a)) and rewards gathered (Figure D.4(b)). As shown in Figure D.4(a) (and Figure 3 in the main text), there is a positive relationship between financial literacy and rounds lasted for quantiles in the range 0.15 – 0.65. Figure D.4(b) illustrates that there is a broad positive relationship between financial literacy and rewards gathered for most of the quantiles. However, as discussed in the main text, this effect is less strong than some of the other measures, and only significant for a subset of the quantiles.

**Figure D.5** Scatterplot of well-being (as measured by WHO-5) and **a.)** rounds lasted and **b.)** rewards earned. For each graph the best fit quantile regression lines are included for the quantiles 0.05 - 0.95 in increments of 0.1.

*Well-being:* Figure D.5 illustrates the relationship between well-being (as measured by WHO-5) and both rounds lasted (Figure D.5(a)) and rewards gathered (Figure D.5(b)). Well-being is positively related to rounds lasted for several of the quantiles near the median quantile (see Figure D.5(a)). There is a positive relationship in most of the quantiles between well-being and rewards gathered (see Figure D.5(b)). However, only a few of these are significant (please the main text for a discussion).

**Figure D.6** Scatterplot of delay discounting (as measured by the log of k) and **a.)** rounds lasted and **b.)** rewards earned for Experiment 2. For each graph the best fit quantile regression lines are included for the quantiles 0.05 - 0.95 in increments of 0.1.

***Experiment 2***

*Delay discounting:* Figure D.6 illustrates the relationship between delay discounting and both rounds lasted (Figure D.6(a)) and rewards gathered (Figure D.6(b)) for Experiment 2. Broadly, there is a negative relationship between delay discounting and both rounds lasted and rewards gathered. As discussed in the main text (and illustrated in Figure 5), the largest effects of rounds lasted are associated with the quantiles between 0.45 and 0.75. For rewards gathered, Figure D.6(b) shows that the largest negative associations are found at the 0.75 and 0.85 quantile.

**Figure D.7** Scatterplot of harmful drinking (as measured by AUDIT) and **a.)** rounds lasted and **b.)** rewards earned for Experiment 2. For each graph the best fit quantile regression lines are included for the quantiles 0.05 - 0.95 in increments of 0.1

*Harmful alcohol use:* Figure D.7 illustrates the relationship between harmful alcohol use (as measured by AUDIT) and both rounds lasted (Figure D.7(a)) and rewards gathered (Figure D.7(b)) in Experiment 2. Broadly, Figure D.7 illustrates that there is a negative relationship between harmful use and both rounds lasted and rewards gathered. Figure 5 denotes which of the quantiles show a significant association.

**Supplemental Material E – Is the mean replenishment rate correlated with either points gathered, or rounds lasted?**

As the replenishment rate was stochastic, it was possible that some participants interacted with a resource that, on average, replenished by a higher or lower proportion compared to other participants. This could, potentially, influence behaviour in the game. To test whether replenishment rates were associated with resource outcomes, we calculated the mean replenishment rate over the first *x* rounds for each participant, and then ran Spearman correlations between these mean replenishment rates and each participant’s rounds lasted and rewards gathered. We calculated the mean replenishment rate and ran these correlations for the first 1, 2, 3, 5, 10, 20, 50, and 70 rounds for both experiments; the results of these correlations are summarised in Table E.1 and Table E.2, below. None of these correlations were significant. Therefore, we conclude that the variance in replenishment rates did not lead to any substantial changes in participants’ behaviour in the game in either experiment.

***Experiment 1***

**Table E.1**: Results of the Spearman correlations run to determine whether there was an association between the resource’s mean replenishment rate and resource outcomes in Experiment 1. **x**: the number of rounds used to calculate mean replenishment rate (1 = first round, 2 = first 2 rounds, 70 = all rounds, etc.); **r_s_**: Spearman’s rank correlation coefficient; **p:** p-value for the Spearman’s correlation.

| *x* | Rounds lasted | | Rewards Gathered | |
| --- | --- | --- | --- | --- |
|  | *r_s_* | *p* | *r_s_* | *p* |
| 1 | -.05 | .336 | .01 | .886 |
| 2 | -.02 | .699 | .05 | .343 |
| 3 | -.04 | .481 | .03 | .533 |
| 5 | -.04 | .384 | .02 | .639 |
| 10 | -.04 | .484 | .02 | .671 |
| 20 | -.02 | .673 | .01 | .799 |
| 50 | -.02 | .746 | .00 | .951 |
| 70 | -.02 | .752 | .02 | .674 |

***Experiment 2***

**Table E.2**: Results of the Spearman correlations run to determine whether there was an association between the resource’s mean replenishment rate and resource outcomes in Experiment 2. **x:** the number of rounds used to calculate mean replenishment rate (1 = first round, 2 = first 2 rounds, 70 = all rounds, etc.); **r_s_:** Spearman’s rank correlation coefficient; **p:** p-value for the Spearman’s correlation.

| *x* | Rounds lasted | | Rewards Gathered | |
| --- | --- | --- | --- | --- |
|  | *r_s_* | *p* | *r_s_* | *p* |
| 1 | -.06 | .268 | -.03 | .621 |
| 2 | -.04 | .411 | -.01 | .801 |
| 3 | -.05 | .379 | -.02 | .663 |
| 5 | -.03 | .562 | -.02 | .689 |
| 10 | -.03 | .557 | -.03 | .607 |
| 20 | -.04 | .411 | -.01 | .801 |
| 50 | -.04 | .386 | -.02 | .627 |
| 70 | -.04 | .392 | -.03 | .569 |

**Supplemental Material F – The impact of knowing the termination rule on the association between delay discounting and resource management**

Recently, we found that knowing the termination rule for our resource management game improves sustainable behaviour (Rauwolf & Rogers, 2023). In the study, half the participants played the game exactly as described in the main text. The other half were given the number of remaining rounds after each harvest – the termination rule. One hundred of those who knew the termination rule also completed the ED_50_ as a measure of delay discounting. This was exploratory analysis, so it was not analysed in the paper. We present the analysis here.

Spearman’s correlations showed that delay discounting (as the log of *k*) was negatively associated with both rounds lasted (r_s_(98)= -0.47, *p* = 1.57x10^-6^) and rewards gathered (r_s_(98) = -0.44, *p=*7.47x10^-6^) for the participants who knew the termination rule. Further, the Spearmen’s rank correlation coefficient (i.e., the effect size) was larger than that found in either Experiment 1 (rounds lasted: r_s_=-0.31, rewards gathered: r_s_= -0.27) and 2 (rounds lasted: r_s_=-0.34, rewards gathered: r_s_= -0.31) in the current paper. This suggests that knowing the termination rule did not reduce (and if anything increased) the association between delay discounting and sustainable behaviour.

Figure F.1 illustrates the quantile regression for the participants who knew the termination rule. Figure F.1(a) shows that higher delay discounting was significantly associated with fewer rounds lasted from 0.05-0.50 quantiles. Further, compared to Figure 3(c) and Figure 5(c), the beta values in Figure F.1(a) (which is the effect size) were larger from the 0.05-0.40 quantiles. However, the effect diminished after the 0.50 quantile. Since the participants knew

Figure F.1 Quantile regression of rounds lasted **(a)** and rewards gathered **(b)** against normalized (mean=0; SD=1) delay discounting (ED_50_) from quantiles 0.05-0.95 in increments of 0.05. Shaded areas represent 95% CIs.

the length of the game, many more sustained the resource for all 70 rounds. Thus, there was no effect of delay discounting in the best performing half of the participants.

Figure F.1(b) illustrates that there is a significant effect of delay discounting on rewards harvested in the quantile range 0.05-0.90. Further, the effect sizes of delay discounting were generally larger in those who know the termination rule, compared to the effect sizes found in the current paper. For example, when estimating median performance (i.e., the 0.5 quantile), one standard deviation increase in delay discounting was associated with a loss of 59 rewards. This was over twice the effect compared to the 27 rewards found in Experient 1 (see Figure 3(d)) and the 23 rewards found in Experiment 2 (see Figure 5(d)).

By comparing Figure F.1 to Figure 3 and 5, it is clear that, in general, knowing the termination rule of the resource management game increased the negative association between delay discounting and sustainable behaviour. Given this, we find it unlikely that the results found in the main text are confounded by the unknown termination rule. We expect that health risk factors are associated with poor sustainable behaviour, regardless of knowledge about the termination rule; however, it would be helpful if future studies extended this exploratory analysis to the other measures used in the current paper.
